# A Plug-and-Play Platform for the Formation of Trifunctional Cysteine Bioconjugates that also Offers Control over Thiol Cleavability

**DOI:** 10.1021/acs.bioconjchem.1c00057

**Published:** 2021-03-12

**Authors:** Calise Bahou, Peter A. Szijj, Richard J. Spears, Archie Wall, Faiza Javaid, Afrah Sattikar, Elizabeth A. Love, James R. Baker, Vijay Chudasama

**Affiliations:** † Department of Chemistry, University College London, 20 Gordon Street, WC1H OAJ, London, United Kingdom; ‡ Research Institute for Medicines (iMed.ULisboa), Faculty of Pharmacy, Universidade de Lisboa, 1649-004 Lisbon, Portugal; § LifeArc, Accelerator Building, SBC Open Innovation Campus, SG1 2FX, Stevenage, United Kingdom

## Abstract

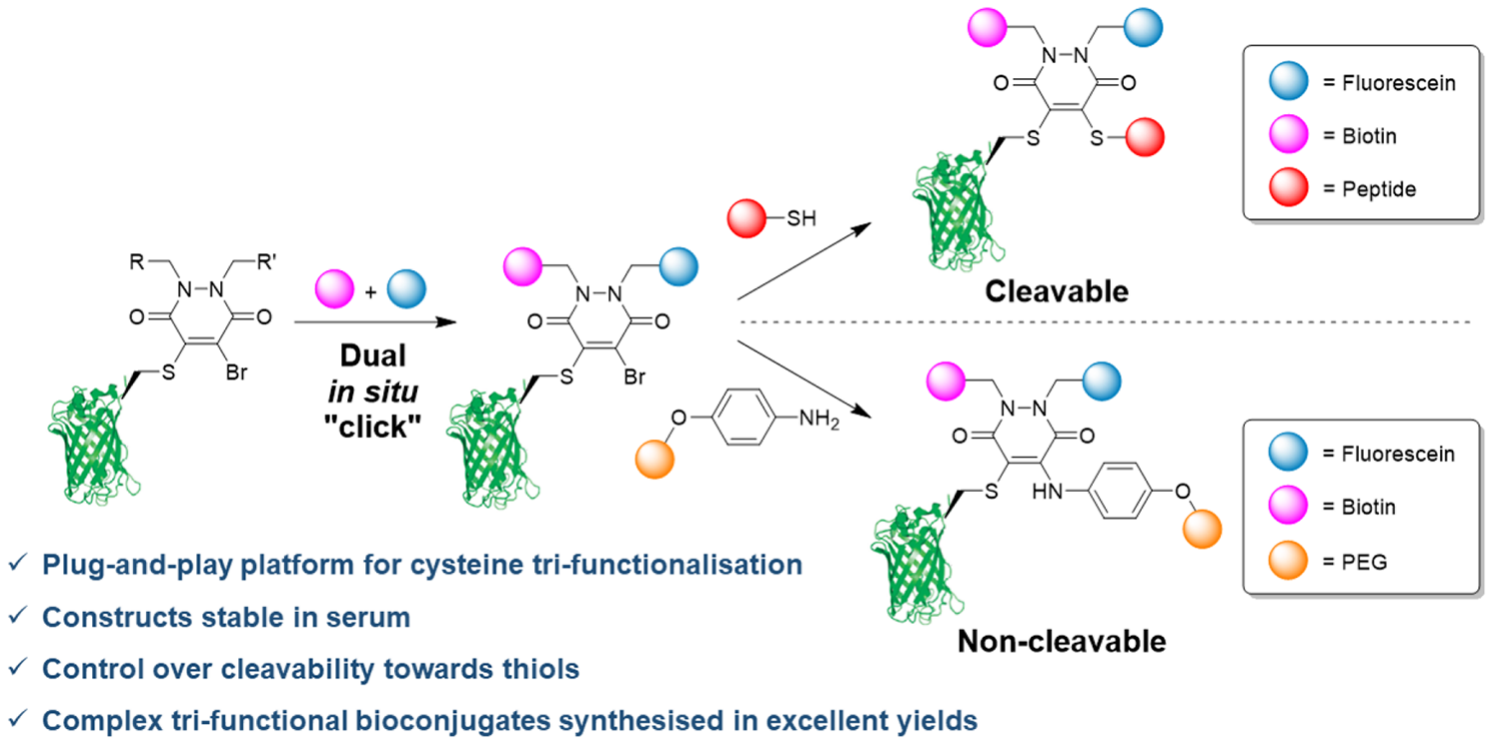

Linkers that enable the site-selective synthesis of chemically modified proteins are of great interest to the field of chemical biology. Homogenous bioconjugates often show advantageous pharmacokinetic profiles and consequently increased efficacy *in vivo*. Cysteine residues have been exploited as a route to site-selectively modify proteins, and many successfully approved therapeutics make use of cysteine directed conjugation reagents. However, commonly used linkers, including maleimide–thiol conjugates, are not stable to the low concentrations of thiol present in blood. Furthermore, only a few cysteine-targeting reagents enable the site-selective attachment of multiple functionalities: a useful tool in the fields of theranostics and therapeutic blood half-life extension. Herein, we demonstrate the application of the pyridazinedione motif to enable site-selective attachment of three functionalities to a protein bearing a single cysteine residue. Extending upon previously documented dual modification work, here we demonstrate that by exploiting a bromide leaving group as an additional reactive point on the pyridazinedione scaffold, a thiol or aniline derivative can be added to a protein, post-conjugation. Thiol cleavability appraisal of the resultant C–S and C–N linked thio-bioconjugates demonstrated C–S functionalized linkers to be cleavable and C–N functionalized linkers to be noncleavable when incubated in an excess of glutathione. The plug-and-play trifunctional platform was exemplified by attaching clinically relevant motifs: biotin, fluorescein, a polyethylene glycol chain, and a model peptide. This platform provides a rare opportunity to combine up to three functionalities on a protein in a site-selective fashion. Furthermore, by selecting the use of a thiol or an amine for functionalization, we provide unique control over linker cleavability toward thiols, allowing this novel linker to be applied in a range of physiological environments.

Modification of therapeutic proteins in a site-selective and homogeneous fashion has contributed to the synthesis of pharmacokinetically superior bioconjugates.^1,2^ Synthesis of these constructs through cysteine modification has been popular in recent years.^3−5^ The low natural abundance of cysteine (<2%) combined with a highly nucleophilic thiol side chain has resulted in many reported cases of site-selectively modified bioconjugates that display a high level of homogeneity.^6−8^ Furthermore, where a solvent-accessible cysteine residue is not available, one can be readily incorporated via site-directed mutagenesis.^9^ A variety of small molecule reagents exist for the purpose of chemoselectively modifying protein surface thiols (e.g., π-clamp, carbonylacrylic reagents, divinylpyridines (DVPs), bis-sulfones, etc.).^10−14^ Such cysteine-directed reagents have been used to synthesize therapeutic bioconjugates, including oriented attachment of proteins to nanoparticles,^15,16^ fluorescent probes,^17,18^ and antibody–drug conjugates (ADCs).^19,20^

In recent years, we have reported the extensive use of bromopyridazinediones (Br PDs) and dibromopyridazinediones (DiBr PDs) in the fields of single cysteine modification and functional disulfide rebridging, respectively.^16,21−24^ The pyridazinedione (PD) moiety has been shown to address many of the drawbacks associated with commonly employed Michael acceptors (e.g., maleimides) in the context of cysteine modification, providing a linker that is stable in serum for several days (i.e., PD-derived bioconjugates do not react with high concentrations of human serum albumin (HSA) and low concentrations of glutathione (GSH)).^25,26^ PD-protein constructs have also been shown to be cleavable in high concentrations of a reactive thiol (e.g., 2-mercaptoethanol (BME)),^21^ offering a proposed release mechanism under intracellular early endosomal conditions (i.e., a high GSH concentration between 1 and 10 mM, and a pH range 6.8–5.9).^27−32^ Furthermore, the PD scaffold can readily incorporate two functional handles for use in bio-orthogonal dual click reactions that can occur post-conjugation. This approach to protein modification has been exploited for the synthesis of antibody theranostics and chemically constructed bispecifics.^22,33^

This work aims to exploit an additional point of attachment on the PD scaffold, that may be applied for the modification of surface thiol residues on proteins. To date, Br PDs have typically been used for single cysteine modification,^16^ but it is thought that by employing DiBr PDs for this purpose, a further reactive position on the PD scaffold could be exploited for the modular synthesis of trifunctional bioconjugates (Figure 1). We have shown in previous work that the addition of a secondary nucleophile (i.e., thioglucose) to this Michael acceptor can yield *C-*functionalized bioconjugates.^21^ Here, we look to exploit this “click-like” Michael addition as a general strategy to form trifunctional bioconjugates in a similar time frame to reported click reactions and when applied under biocompatible conditions.^21,34^ Through installment of this post-conjugation *C*-linked functionality alongside previously reported click reactions, we aim to provide a first-in-class method to produce trifunctional cysteine conjugates through three successive bio-orthogonal “click-like” reactions. Furthermore, the trifunctionalized PD-based bioconjugates to be generated will be designed to investigate whether the addition of certain functional groups can induce differential cleavability toward high concentrations of thiols, potentially offering control over cleavability for distinct trifunctionalized PD-based bioconjugates under intracellular conditions.

**Figure 1:**
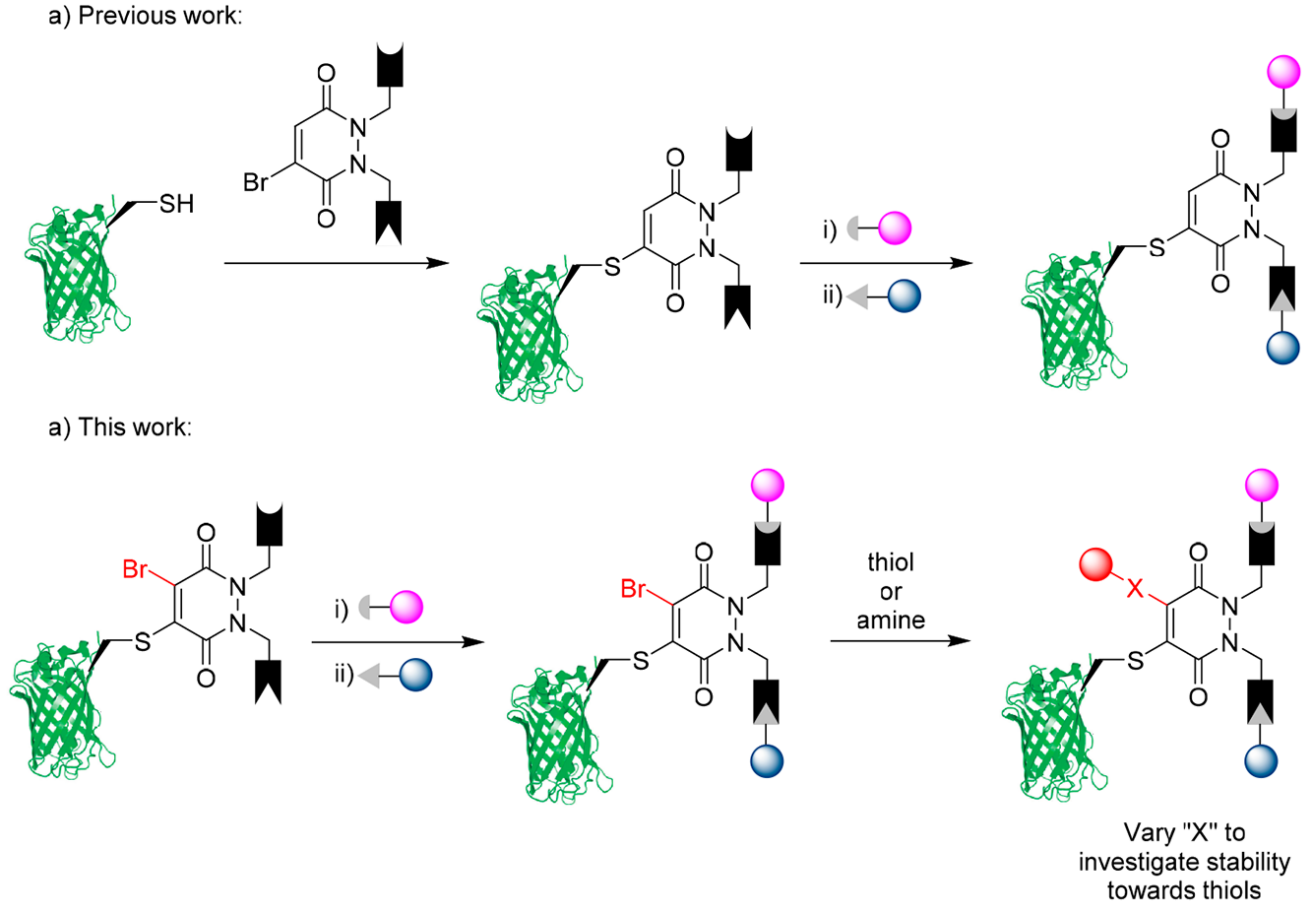
(a) Reaction of a single cysteine containing protein and subsequent dual modification, using bromopyridazinediones (Br PDs). (b) Proposed reaction between a single cysteine containing protein and dibromopyridazinediones (DiBr PDs) to afford a bromo-containing intermediate species to aid in addition of a third functionality.

## Developing a Platform for Trifunctionalization

In the initial stages of development, we sought to provide a platform for trifunctionalization through two strategies: (i) preprotein bioconjugation and (ii) postprotein bioconjugation. For both strategies, DiBr PDs were used as a convenient starting point, as this reactive Michael acceptor can act as a synthetic branching point, and an optimized synthesis of DiBr PDs has been reported.^23^ Once synthesized, small molecules were reacted with a GFP mutant GFPS147C **1** (Ser^147^ to Cys^147^ mutation). GFP, a functional fluorescent protein platform, has been extensively applied for a range of applications (e.g., Förster resonance energy transfer (FRET), gene imaging, tumor imaging, etc.) and has a well-defined reactive cysteine residue for bioconjugation.^35^ All transformations using GFPS147C were monitored using LCMS analysis. Due to the presence of a small amount of GFP homodimer species, GFPS147C **1** was reduced with tris(2-carboxyethyl)phosphine (TCEP) prior to bioconjugation reactions (see Supporting Information for details).

### Preconjugation Functionalization

Diethyl (DiEt) DiBr PD was selected as a simplistic model without the complex N,N′ functionality, and was reacted in organic solvent with various thiols (*n*-hexanethiol, 2-mercaptoethanol (BME), and 3-mercaptopropionic acid) to produce C–S functionalized PDs **2**–**4** and an amine (*n*-hexylamine) to produce a C–N functionalized PD **9**. The C–S functionalized bromo-thio PDs (**2**–**4**) were obtained in moderate yields (**2**, 60%; **3**, 71%; **4**, 58%) and required mild base (NaOAc) to facilitate the reaction (see Supporting Information for details). C–N functionalized PD **9** was afforded with a similar yield (71%) but required stronger basic conditions (NaOH) for formation (see Supporting Information for details), potentially limiting base-sensitive groups from the scope of functionality that may be added preconjugation.

**Figure 2:**
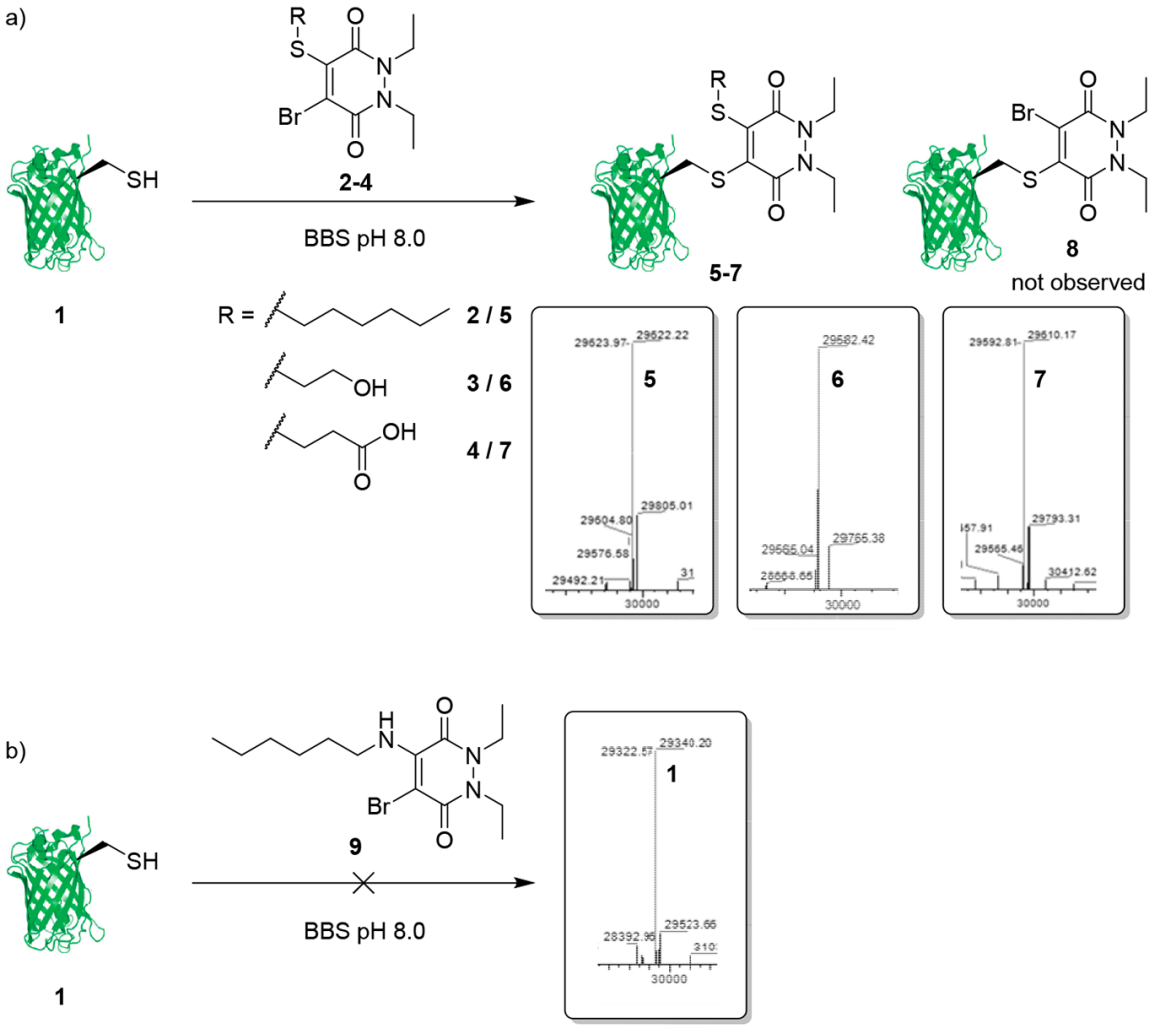
(a) Reaction between bromo-thio PD species **2**–**4** and GFPS147C **1** to form GFP-PD-thiol species **5**–**7** only. (b) Reaction between bromo-amino PD species **9** and GFPS147C **1** showing no observable reaction.

Upon reaction with GFPS147C **1** (50 μM, pH 8.0), the bromo-thio PDs **2**–**4** (1 mM final concentration) afforded the GFP-PD-thiol species **5**–**7** only (Figure 2a). This result provides evidence to suggest full thiol selectivity for the bromo position on the PD over competing thiol exchange (i.e., forming the undesired GFP-PD-Br species **8**). Interestingly, the bromo-amino PD **9** did not show any reactivity toward GFPS147C **1** (Figure 2b). This suggests that amine substitution at this position is sufficient to decrease the electrophilicity of the Michael acceptor, such that thiol reactivity is quenched. Therefore, it was envisaged that if an amine could be reacted at this position postconjugation, this transformation may confer an additional level of stability toward thiol exchange (i.e., the resultant species may be stable to high concentrations of thiol).^36^

### Postconjugation Functionalization

**Figure 3:**
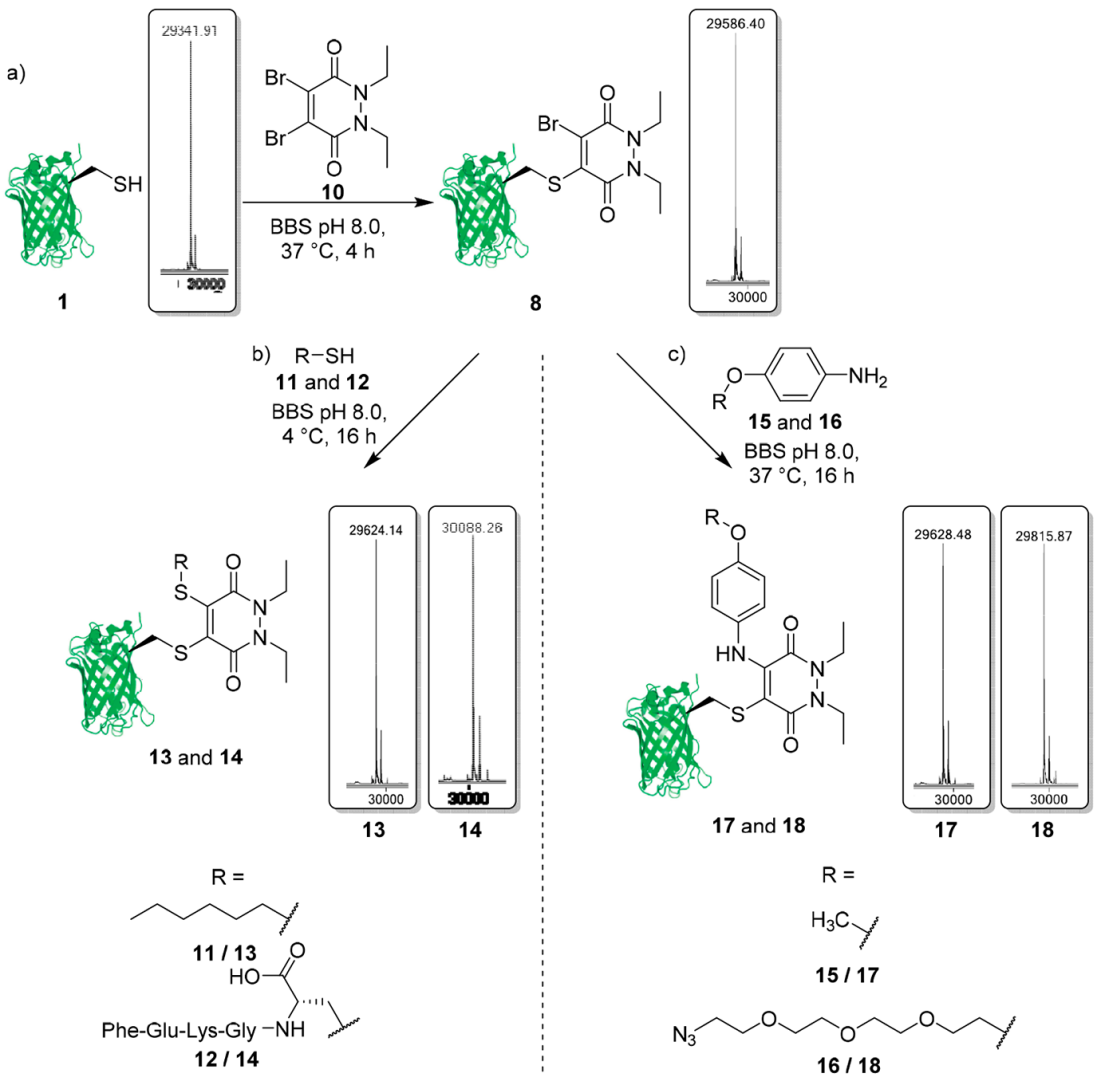
Postconjugation functionalization strategy. (a) Reaction of DiEt DiBr PD **10** with GFPS147C **1** to form GFP-PD-Br species **8**. (b) Postconjugation functionalization of GFP-PD-Br species **8** with a thiol (1-hexanethiol **11** and peptide (FEKGC) **12**) to produce C–S functionalized GFP-PD species **13** and **14**. (c) Postconjugation functionalization of GFP-PD-Br species **8** with aniline derivatives (*p-*anisidine **15** and aniline-azide **16**) to produce N-functionalized GFP-PD species **17** and **18**.

DiEt DiBr PD **10** was again selected as a simplistic model for C–S and C–N functionalization, postconjugation. By first reacting the DiEt DiBr PD **10** (1 mM final concentration) with GFPS147C **1** (50 μM, pH 8.0) at 37 °C for 4 h, quantitative formation of the GFP-PD-Br species **8** was observed (Figure 3a). We envisaged that the remaining bromine-substituted position on the PD may react further with thiol and amine groups in bio-orthogonal conditions, to produce C–S and C–N functionalized linkers.

The GFP-PD-Br species **8** (50 μM, pH 8.0) was reacted with 1-hexanethiol **11** (1 mM final concentration) at 4 °C for 16 h, to quantitatively produce the GFP-PD-thiol species **13** (Figure 3b). Once again, nucleophilic attack of the thiol was selective for the bromo-substituted position on the PD, and undesired thiol exchange/PD cleavage (to form unconjugated GFPS147C **1**) was not observed. Notably, bioconjugations that exceeded a final concentration of 1 mM 1-hexanethiol **11** began to result in cleavage of the PD moiety from GFPS147C **1** (i.e., through thiol exchange and elimination of the GFPS147C **1** from the PD). The reaction between 1-hexylamine (50 mM final concentration, pH 8.0) and the GFP-PD-Br species **8** (50 μM, pH 8.0) at 37 °C for 16 h did not yield any of the desired product, likely due to a large proportion of the aliphatic primary amine being protonated under bioconjugation conditions (i.e., p*K*_a_ of *n*-hexylamine = approximately 10.5).^37^ Following the recently documented reaction between aniline-derivatives and dibromomaleimide scaffolds, that occurs under biocompatible conditions to induce stability toward thiols and hydrolysis,^36^ the aniline derivative *p-*anisidine **15** (50 mM final concentration) was reacted with the GFP-PD-Br species **8** (50 μM, pH 8.0) at 37 °C for 16 h, which quantitatively produced the GFP-PD-amino species **17** (Figure 3c).

Finally, we sought to react the GFP-PD-Br species **8** with functional thiols and aniline derivatives using optimized conditions (pH 8.0, 1 mM thiol, 50 mM amine), to determine if complex functionality may affect amine/thiol conjugation. A cysteine containing model peptide (FEKGC) **12** was selected as a functional thiol, representing many chemical moieties in the form of amino acid side chains. The peptide **12** (1 mM final concentration) was reacted with the GFP-PD-Br species **8** (50 μM, pH 8.0) at 4 °C for 16 h, to quantitatively produce the C–S functionalized GFP-PD-peptide **14** (Figure 3b). As the number of commercially available aniline-derived functional compounds is low, an azide-harboring aniline derivative was synthesized for use as a platform to subsequently conjugate functionality through “click” chemistries. The aniline–azide **16** (50 mM final concentration) was reacted with the GFP-PD-Br species **8** (50 μM, pH 8.0) at 37 °C for 16 h, which pleasingly resulted in quantitative formation of the C–N functionalized GFP-PD-azide species **18** (Figure 3c).

## Thiol Cleavability and Serum Stability Appraisal of C–S and C–N Functionalized Linkers

Stability toward blood thiols (i.e., HSA and GSH) remains a challenge for bioconjugation reagents that target cysteine residues for modification. When using cysteine-reactive reagents (e.g., maleimides) the resultant bioconjugates are often still mildly thiol reactive, and highly abundant thiols in blood can facilitate premature release of cargo through thiol exchange. Targeted therapeutic bioconjugates with applications *in vivo* must be stable in blood to allow for optimal delivery of chemically attached cargo to the target of interest. Here, we appraise the cleavability of C–S and C–N functionalized bioconjugates in serum and in high concentrations of glutathione to investigate how these constructs may behave in certain physiological environments *in vivo*.

**Figure 4:**
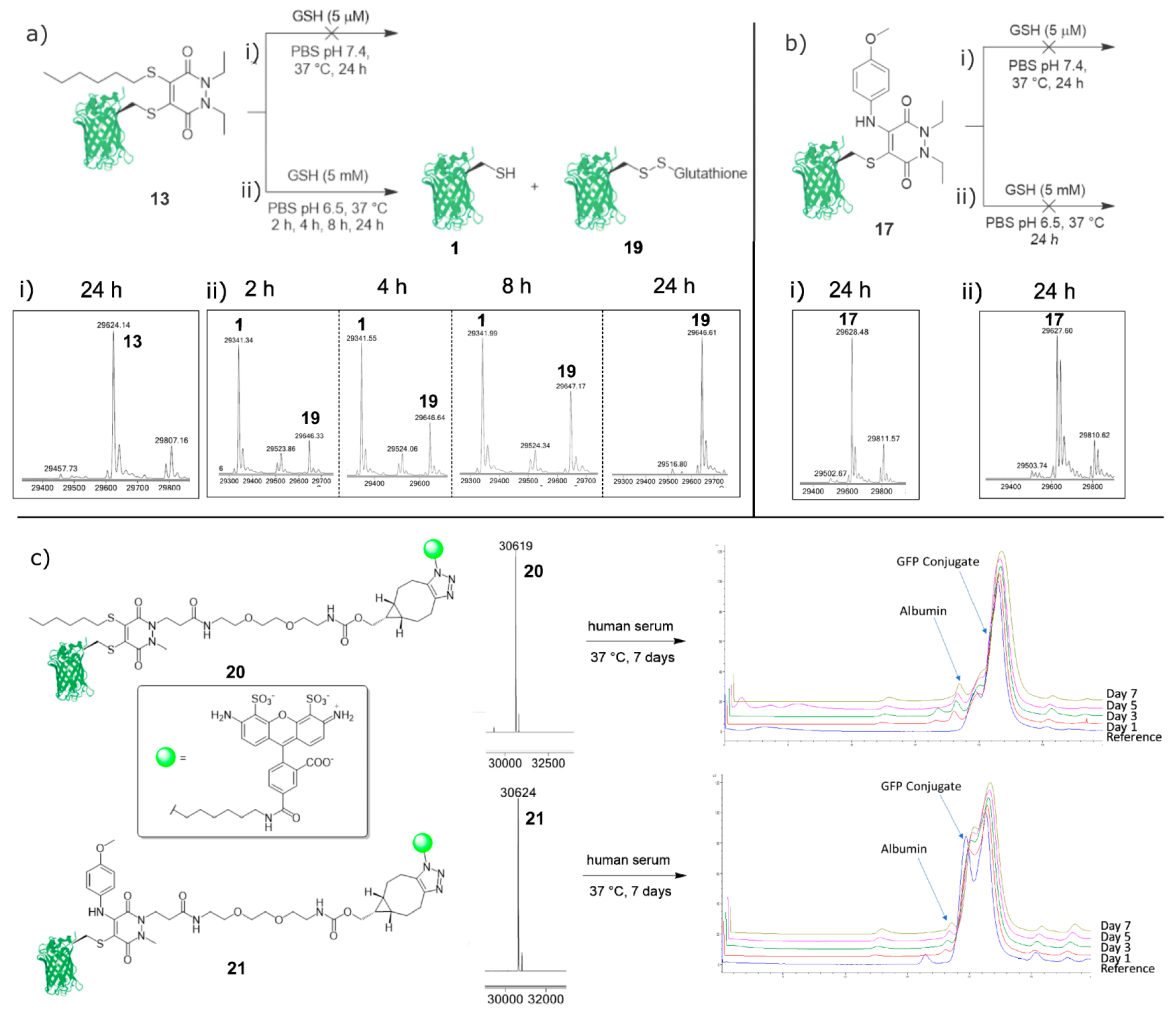
(a) (i) Incubation of C–S functionalized GFP-PD-thiol species **13** (6.7 μM, pH 7.4) with GSH (5 μM) at 37 °C for 24 h. (ii) Incubation of C–S functionalized GFP-PD-thiol species **13** (6.7 μM, pH 6.5) with GSH (5 mM) at 37 °C for 24 h (time points taken at 2, 4, 8, and 24 h). (b) Incubation of C–N functionalized GFP-PD-aniline species **17** (6.7 μM, pH 7.4) with GSH (5 μM) at 37 °C for 24 h. (ii) Incubation of C–N functionalized GFP-PD-aniline species **17** (6.7 μM, pH 6.5) with GSH (5 mM) at 37 °C for 24 h. (c) Incubation of C–S functionalized GFP-PD(AF-488)-thiol species **20** (3.3 μM, pH 7.4) and C–N functionalized GFP-PD(AF-488)-aniline species **21** (3.3 μM, pH 7.4) in human serum for 7 days (see Supporting Information for further details).

The model DiEt C–S and C–N functionalized GFP-PD species **13** and **17** were incubated with low (5 μM, pH 7.4) and high (5 mM, pH 6.5) concentrations of GSH to mimic the thiol concentrations found in blood and in the early endosome, respectively (reactions were monitored using LCMS analysis).^27^ The GFP-PD-thiol species **13** showed no significant reaction with blood concentrations of GSH (5 μM pH 7.4), and the construct remained unaffected for the 24 h incubation (Figure 4ai). When exposed to high concentrations of GSH (pH 6.5, 5 mM GSH), full cleavage of the PD was observed in just 2 h (Figure 4aii). GFPS147C **1**, once liberated from the PD, began to oxidize with GSH to form the GFP-GSH species **19** (Figure 4aii). Isolation of only the GFP-GSH species **19** after 24 h through thiol oxidation is promising evidence that the cysteine thiol was unaffected by the PD conjugation/cleavage. Interestingly, after incubating the GFP-PD-amino species **17** for 24 h in blood concentrations (5 μM) and early endosomal concentrations (pH 6.5, 5 mM) of GSH, no PD cleavage was observed (Figure 4b). These observations suggest that amine conjugation at this position results in a high degree of electron density being donated into the pyridazinedione ring, which consequently decreases the electrophilicity of the resultant product as well as decreasing reactivity toward nucleophiles (e.g., thiols). Therefore, a high degree of thiol stability can indeed be achieved though amine substitution/functionalization on the PD scaffold.

Finally, we looked to appraise the stability of the C–S and C–N functionalized GFP-PD conjugates in human serum. A serum stability study was conducted to assess whether the conjugated PDs would participate in thiol exchange with the abundant blood thiol HSA. For this assay, we required fluorophore-functionalized PDs in order to trace potential transfer to HSA. An AlexaFluor-488 (AF-488) functionalized GFP-PD(AF-488)-thiol species **20** and GFP-PD(AF-488)-amino species **21** were successfully synthesized through use of an AF-488 azide and a strained alkyne (BCN) DiBr PD derivative (see Supporting Information for details) (Figure 4c). Notably, when GFP and GFP-based bioconjugates were analyzed using SEC-HPLC, two species can be observed in the reference sample despite being confirmed as a single species by LCMS. This observation is likely a result of the native (i.e., nondenaturing) conditions employed when running the SEC-HPLC. The GFP-PD(AF-488)-thiol species **20** and GFP-PD(AF-488)-amino species **21** were then incubated in human serum for 7 days and analyzed by SEC-HPLC (Figure 4c). Both C–S and C–N functionalized PDs were shown to be stable for the 7-day incubation period, showing no significant transfer of the AF-488 labeled PD to HSA during this time. Stability of these species was confirmed by comparing relative fluorescence intensities between the GFP species and HSA over time. As the ratio between HSA and GFP species **20** and **21** did not increase over the 7-day study, we can conclude that these novel constructs are likely stable to serum concentrations of HSA. Furthermore, this data confirms that the synthesized constructs were stable to aggregation for at least 7 days in serum (i.e., minimal GFP-derived aggregate was detected by SEC-HPLC).

## Installment of Clinically Relevant Features through Trifunctionalization

**Figure 5:**
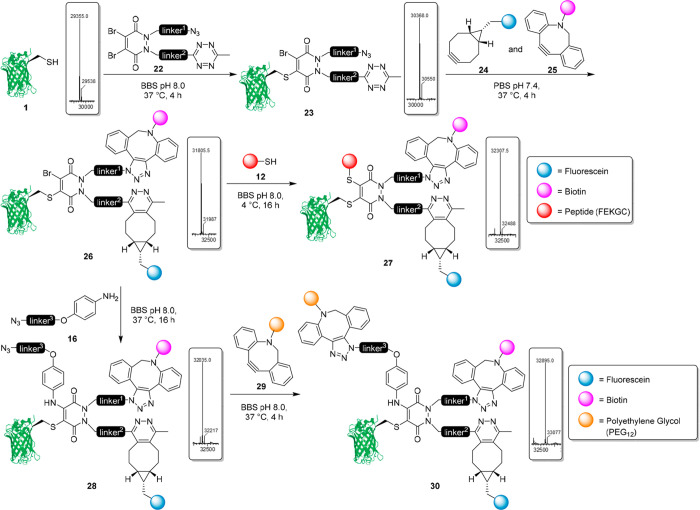
Synthesis of trifunctional C–S derived GFP-PD (biotin-fluorescein-peptide) species **27** and trifunctional C–N derived (biotin-fluorescein-PEG) species **30**.

Through design and synthesis of a dual-clickable DiBr PD with N,N′ tetrazine and azide functionality, we looked to expand this platform to allow for the attachment of three functionalities postconjugation. GFPS147C **1** (50 μM, pH 8.0) was reacted with DiBr tetrazine azide PD **22** (1 mM final concentration) at 37 °C for 4 h to produce the GFP-PD(tetrazine-azide)-Br species **23** (Figure 5). Dibenzocyclooctyl (DBCO)-biotin **24** and bicyclo[6.1.0]nonyne (BCN)-fluorescein **25** were selected as reagents to react with PD-linked azide and tetrazine moieties, respectively (i.e., through SPAAC and SPIEDDAC chemistries). By exploiting the selectivity of DBCO-azide, and BCN-tetrazine click reactions (i.e., the electron-deficient tetrazine reacts preferentially with electron-rich strained alkynes),^38^ the dual modification was achieved in a one-pot fashion to reduce the total time required for complete bioconjugate synthesis. BCN-fluorescein **25** (100 μM final concentration) and DBCO-biotin **24** (500 μM final concentration) were added *in situ* to the GFP-PD(tetrazine-azide)-Br species **23** (50 μM, pH 8.0) and were incubated for 4 h at 37 °C to produce the dual-clicked GFP-PD-Br species **26** only (Figure 5). The presence of GFP-PD(biotin-biotin)-Br or GFP-PD(fluorescein-fluorescein)-Br was not detected, suggesting that the *in situ* addition of BCN and DBCO was successful in selectively modifying tetrazine and azide groups, respectively.

To synthesize the trifunctional C–S species, we once again employed a model peptide. The model peptide (FEKGC) **12** (1 mM final concentration) was reacted with the dual-modified GFP-PD-Br species **26** (50 μM, pH 8.0) at 4 °C for 16 h, to successfully produce the trifunctional C–S modified GFP-PD (biotin-fluorescein-peptide) species **27** with excellent conversion (>90%) (Figure 5). As previously mentioned, few commercially available aniline-derived regents exist for bioconjugation, so an aniline-azide linker was incorporated. The aniline-azide **16** (50 mM final concentration) was reacted with the dual-modified GFP-PD-Br species **26** (50 μM, pH 8.0) at 37 °C for 16 h to produce the trifunctional C–N modified GFP-PD (biotin-fluorescein-azide) species **28**, once again with excellent conversion (>90%) (Figure 5). A DBCO-PEG_12_ reagent **29** (1 mM final concentration) was then added to the C–N modified GFP-PD (biotin-fluorescein-azide) species **28** (50 μM, pH 8.0) at 37 °C and left for 4 h to produce the trifunctional C–N modified GFP-PD (biotin-fluorescein-PEG) species **30**. In the synthesis of both C–S and C–N trifunctionalized bioconjugates, each step was shown to quantitatively form intermediates, and final products were obtained with an excellent conversion (>90%). Only removal of excess small molecules was required for purification, which was achieved through simple desalting or dialysis techniques. This highlights the reliability and accessibility of the chemistry, which when combined with the modularity represents an important method for forming multifunctional bioconjugates with distinctive thiol cleavability achieved from a common branch point.

## Conclusion

Through the use of cysteine conjugation, and subsequent postconjugation functionalization, we demonstrate pyridazinedione derivatives as first-in-class linkers that can provide a platform for the efficient synthesis of chemically diverse bioconjugates that can host up to three functionalities. In this work, we exploit an additional reactive center on the conjugated pyridazinedione scaffold to allow postconjugation reactions with thiols and aniline derivatives. The trifunctional bioconjugates were appraised for thiol cleavability, suggesting that C–S functionalized cysteine bioconjugates were susceptible to cleavage in high concentrations of glutathione, whereas C–N functionalized cysteine bioconjugates were shown to be stable for a minimum of 24 h. We envisage that a platform for site-selective trifunctionalization may contribute to research fields where a combination of functions is required (e.g., therapeutic half-life extension and theranostics). Additionally, offering a further level of thiol cleavability control may allow this platform to be extended to intracellular applications (e.g., *ex vivo* cellular imaging through bioconjugation to cell-penetrating peptides (CPPs)).

## Supporting Information Available

General Experimental details, UV–vis Spectroscopy, Mass Spectrometry, Evaluation of compounds (PDF)
