# Supplementary material for: A Plug-and-Play Platform for the Formation of Trifunctional Cysteine Bioconjugates that also Offers Control over Thiol Cleavability
Source: Bioconjug Chem. 2021 Mar 12;32(4):672–9. doi: 10.1021/acs.bioconjchem.1c00057 (PMC8154211; doi:10.1021/acs.bioconjchem.1c00057)
Supplement: Supplementary file 1 — bc1c00057_si_001.pdf [file bc1c00057_si_001.pdf]

Supporting Information

A plug-and-play platform for the formation of tri-functional cysteine bioconjugates that also offers control over thiol cleavability

Calise Bahou[1], Peter A. Szijj[1], Richard J. Spears[1], Archie Wall[1], Faiza Javaid[1], Afrah Sattikar[3], Elizabeth A. Love[3], James R. Baker*[1] and Vijay Chudasama*[1,2]

[1]Department of Chemistry, University College London, 20 Gordon Street, WC1H OAJ, London, United Kingdom

[2]Research Institute for Medicines (iMed.ULisboa), Faculty of Pharmacy, Universidade de Lisboa, 1649-004 Lisbon, Portugal

[3]LifeArc, Accelerator Building, SBC Open Innovation Campus, SG1 2FX, Stevenage, United Kingdom

* *E-mails: j.r.baker@ucl.ac.uk, v.chudasama@ucl.ac.uk*

General Experimental

All chemical reagents were purchased from Sigma Aldrich (Merck), Fisher Scientific, Alfa Aesar and Acros. Compounds and solvents were used as received. Petrol refers to petroleum ether (b.p. 40–60 °C). All reactions were carried out under positive pressure of argon, unless stated otherwise, and were monitored using thin layer chromatography (TLC) on pre-coated silica gel plates (254 µm). Flash column chromatography was carried out with pre-loaded FlashPure™ or GraceResolv™ flash cartridges on a Biotage® Isolera Spektra One flash chromatography system. ^{1}H NMR spectra were obtained at 300 MHz, 400 MHz, 500 MHz, 600 MHz or 700 MHz. ^{13}C NMR spectra were obtained at 125 MHz, 150 MHz or 175 MHz. All results were obtained using Bruker NMR instruments, the models are as follows: Avance Neo 700, Avance III 600, DRX 500, Avance III 400, Avance 300. All samples were run at the default number of scans and at 21 °C. Chemical shifts (δ) for ^{1}H NMR and ^{13}C NMR are quoted relative to residual signals of the solvent on a parts per million (ppm) scale. Where amide rotamers are the case, and when possible, only the major rotamer has been assigned for chemical shifts, and areas underneath all rotameric peaks have been considered for integration calculations. Coupling constants (*J* values) are reported in Hertz (Hz) and are reported as $J_{H\text{-}H}$ couplings. Infrared spectra were obtained on a Perkin Elmer Spectrum 100 FTIR spectrometer operating in ATR mode. Mass spectra were obtained for synthetic products from the Swansea EPSRC NMSF facility or the UCL mass spectroscopy service on either a LTQ Orbitrap XL (Swansea), Thermo Finnigan MAT900Xp (EI and CI, UCL) or Waters LCT Premier XE (ES, UCL) mass spectrometer. Melting points were measured with Gallenkamp apparatus and are uncorrected.

UV-Vis spectroscopy

UV-Vis spectroscopy was used to determine protein concentrations and payload to protein ratios, using a Varian Cary 100 Bio UV-Visible spectrophotometer or a NanoDrop™ One microvolume UV-Vis spectrophotometer operating at 21 °C. Sample buffer was used as blank for baseline correction. Extinction coefficients for proteins (at A_{280}) and payloads (at A_{max}) are listed below. A correction factor was applied in the event that the conjugated payload had a competing absorption at A_{280}, which is listed in the table below. PAR values

were calculated by comparing concentrations of the payloads and the protein (calculated with corrected A_{280} values).

**Protein**	**Extinction Coefficient ε $_{280}$ (M^{-1} cm^{-1})**
**Green Fluorescent Protein (GFPS147C)**	20,500 (55,000 @ ε_{495})

**Payload**	**Extinction Coefficient (M^{-1} cm^{-1})**			**Correction Factor**
	ε_{280}	ε_{335}	ε_{495}	
**Pyridazinedione Scaffolds**	2,275	9,100	-	0.25
**AlexaFluor-488**	8,030	-	73,000	0.11

Corrected A_{280} = Experimental A_{280} – (A_{max} × Correction Factor)

Mass Spectrometry **LCMS analysis – Method 1**

LCMS was performed on protein samples (<50 kDa) using a Waters Acquity UPLC connected to Waters ACQUITY Single Quad Detector (SQD). All samples were diluted to a final concentration of 1 mg/mL in deionised water and run with the following parameters. Column: Hypersil Gold C4, 1.9 µm, 2.1 µm × 50 µm. Wavelength: 254 nm. Mobile Phase: 95:5 Water (0.1% Formic Acid): MeCN (0.1% Formic Acid) Gradient over 4 min (to 5:95 Water (0.1% Formic Acid): MeCN (0.1% Formic Acid). Flow Rate: 0.6 mL/min. MS Mode: ES+. Scan Range: *m/z* = 250 – 2000. Scan time: 0.25 s. Data obtained in continuum mode. The electrospray source of the MS was operated with a capillary voltage of 3.5 kV and a cone voltage of 50 V. Nitrogen was used as the nebulizer and desolvation gas at a total flow of 600 L/h. Ion series were generated by integration of the total ion chromatogram (TIC) over the appropriate range. Total mass spectra for protein samples were reconstructed from the ion series using the MaxEnt 1 algorithm pre-installed on MassLynx™ software (Waters). Reported reaction conversions were determined using LCMS analysis and are calculated by comparing intensity of the species present in the deconvoluted data.

LCMS analysis – Method 2

Molecular masses were measured using an Agilent 6510 QTOF LC-MS system (Agilent, UK). Agilent 1200 HPLC system was equipped with an Agilent PLRP-S, 1000A, 8 µM, 150 mm x 2.1

mm column. 10 µL of a protein sample (diluted to 0.2 mg/mL in d.d. H_2O) was separated on the column using mobile phase A (water-0.1% formic acid) and B (acetonitrile-0.1% formic acid) with an eluting gradient (as shown below) at a flow rate of 0.3 mL/min. The oven temperature was maintained at 60 °C.

LCMS mobile phase gradient for A/B elution:

Time (min)	Solvent A (%)	Solvent B (%)
0	85	15
2	85	15
3	68	32
4	68	32
14	65	35
18	5	95
20	5	95
22	85	15
25	85	15

Agilent 6510 QTOF mass spectrometer was operated in a positive polarity mode, coupled with an ESI ion source. The ion source parameters were set up with a VCap of 3500 V, a gas temperature at 350 °C, a dry gas flow rate at 10 L/min and a nebulizer of 30 psig. MS Tof was acquired under conditions of a fragmentor at 350 V, a skimmer at 65 V and an acquisition rate at 0.5 spectra/s in a profile mode, within a scan range between 700 and 4500 *m/z*. The data was then analysed by deconvoluting a spectrum to a zero-charge mass spectrum using a maximum entropy deconvolution algorithm within the MassHunter software version B.07.00. Deconvoluted spectra were avoided where possible in the quantification of conjugates due to differing ionisation tendencies between species with significantly different masses.

LCMS analysis – Method 3

LC-MS was performed on a Thermo Scientific Q-Exactive Plus Orbitrap mass spectrometer coupled to Vanquish Flex UHPLC system using an Agilent PLRP-S column (1,000 Å, 5 µm, S21 2.1 mm x 50 mm). Flow rate: 0.250 ml/min; Run time: 6 min. Solvent A: 0.1% formic acid in water, Solvent B: Acetonitrile; Gradient: 15-90%. Column temperature: 80 ˚C. All mass

spectrometry results were analysed using Thermo Scientific BioPharma Finder informatics platform for protein characterization.

Organic Synthesis

Di-*tert*-butyl 1,2-diethylhydrazine-1,2-dicarboxylate S1[1]

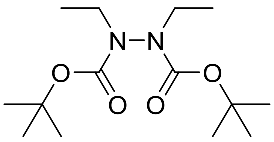

S1

To a solution of di-*tert*-butyl hydrazine-1,2-dicarboxylate (1.16 g, 5.00 mmol) in DMF (20 mL) were added caesium carbonate (3.26 g, 10.0 mmol) and bromoethane (0.70 mL, 14.7 mmol). The heterogeneous mixture was stirred at 21 °C for 24 h. After this time, DMF was removed *in vacuo* with toluene co-evaporation (3 × 50 mL as an azeotrope). The crude reaction mixture was then dissolved in diethyl ether (100 mL), and then washed with water (3 × 30 mL) and saturated aq. LiCl solution (2 × 30 mL). The organic layer was dried ($MgSO_4$) and concentrated *in vacuo* to yield di-*tert*-butyl 1,2-diethylhydrazine-1,2-dicarboxylate **S1** (1.21 g, 4.21 mmol, 81%) as a colourless oil: ^{1}H NMR (600 MHz, $CDCl_3$, rotamers) δ 3.47–3.40 (m, 4H), 1.47–1.42 (m, 18H), 1.15 (t, *J* = 7.2 Hz, 6H); ^{13}C NMR (150 MHz, $CDCl_3$, rotamers) δ 155.2 (C), 80.7 (C), 44.4 (CH_2), 28.4 (CH_3), 13.0 (CH_3); IR (thin film) 2976, 2934, 1702 cm^{-1}.

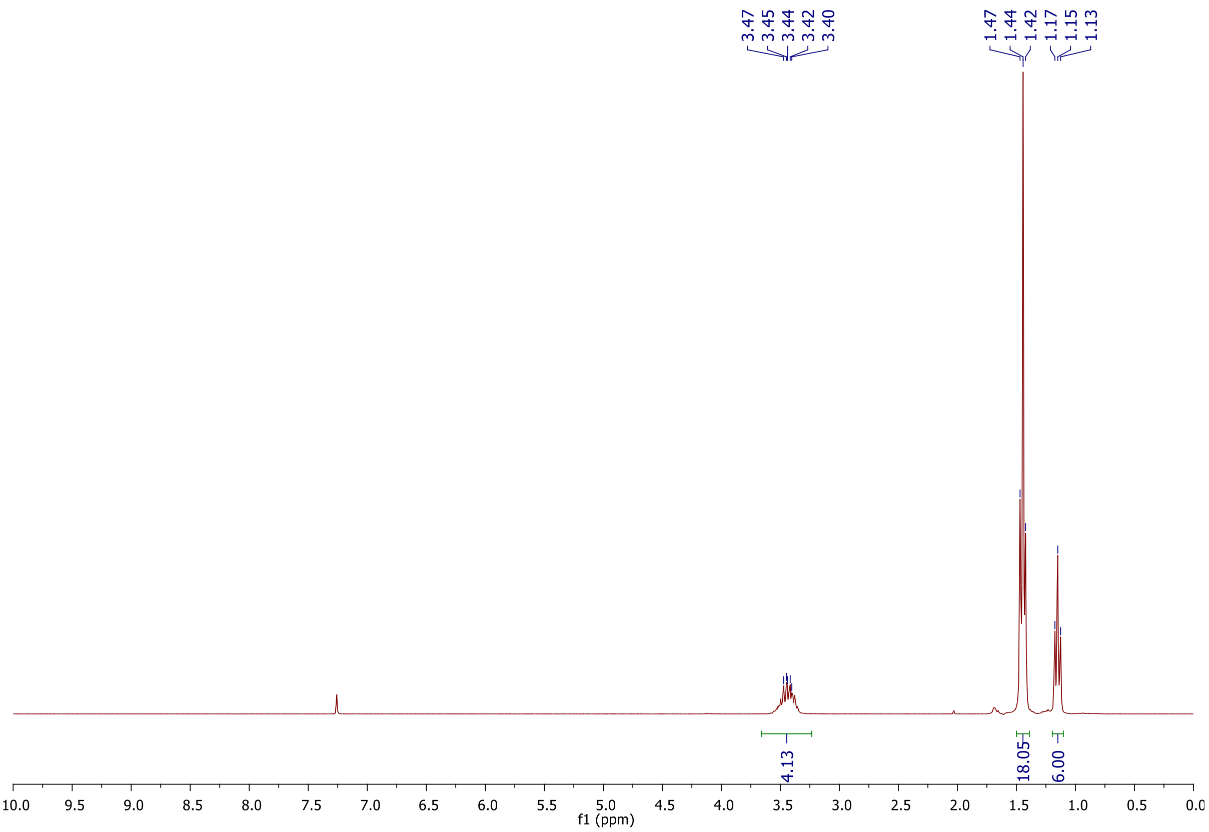
3.47
3.45
3.44
3.42
3.40
1.47
1.44
1.42
1.17
1.15
1.13
4.13
18.05
6.00
f1 (ppm)

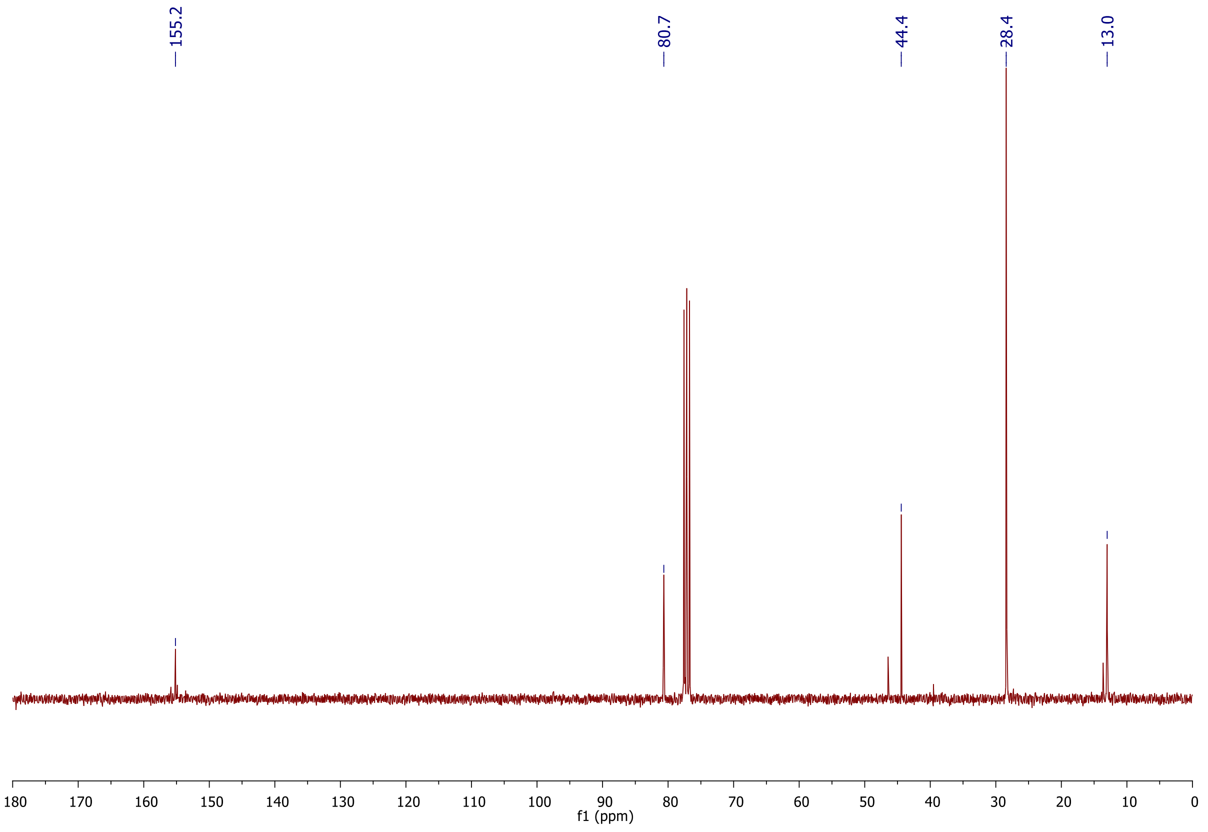
155.2
80.7
44.4
28.4
13.0
f1 (ppm)

4,5-Dibromo-1,2-diethyl-1,2-dihydropyridazine-3,6-dione 10[1]

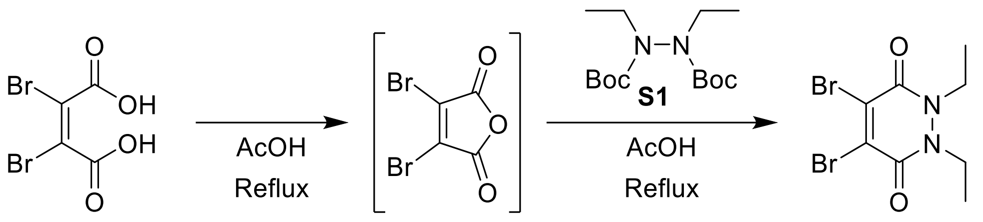

Dibromomaleic acid (274 mg, 1.00 mmol) was dissolved in AcOH (10 mL) and heated under reflux for 2 h. After this time, di-*tert*-butyl 1,2-diethylhydrazine-1,2-dicarboxylate **S1** (347 mg, 1.20 mmol) was added and the resultant mixture was heated under reflux for a further 4 h. After this time, the reaction mixture was concentrated *in vacuo* with toluene co-evaporation (3 × 20 mL, as an azeotrope). Purification of the crude residue by flash column chromatography (30% to 70% EtOAc/petrol) yielded 4,5-dibromo-1,2-diethyl-1,2-dihydropyridazine-3,6-dione **10** (267 mg, 0.819 mmol, 82%) as a yellow solid: m.p. 110–115 °C; ^{1}H NMR (600 MHz, $CDCl_3$) δ 4.17 (q, *J* = 7.1 Hz, 4H), 1.29 (t, *J* = 7.1 Hz, 6H); ^{13}C NMR (150 MHz, $CDCl_3$) δ 153.2 (C), 136.1 (C), 42.4 (CH_2), 13.1 (CH_3); IR (solid) 2979, 2937, 2873, 1629, 1574 cm^{-1}.

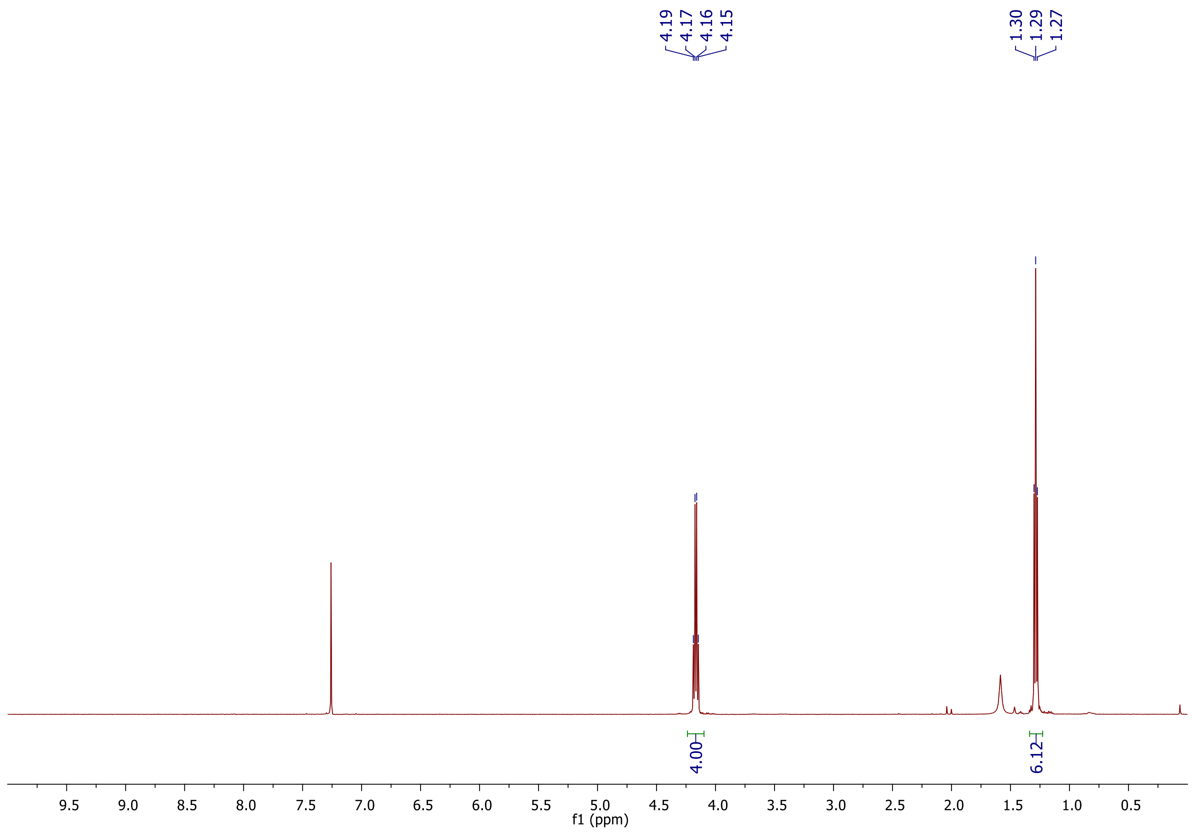
4.19
4.17
4.16
4.15
1.30
1.29
1.27
4.00
6.12
9.5 9.0 8.5 8.0 7.5 7.0 6.5 6.0 5.5 5.0 4.5 4.0 3.5 3.0 2.5 2.0 1.5 1.0 0.5
f1 (ppm)

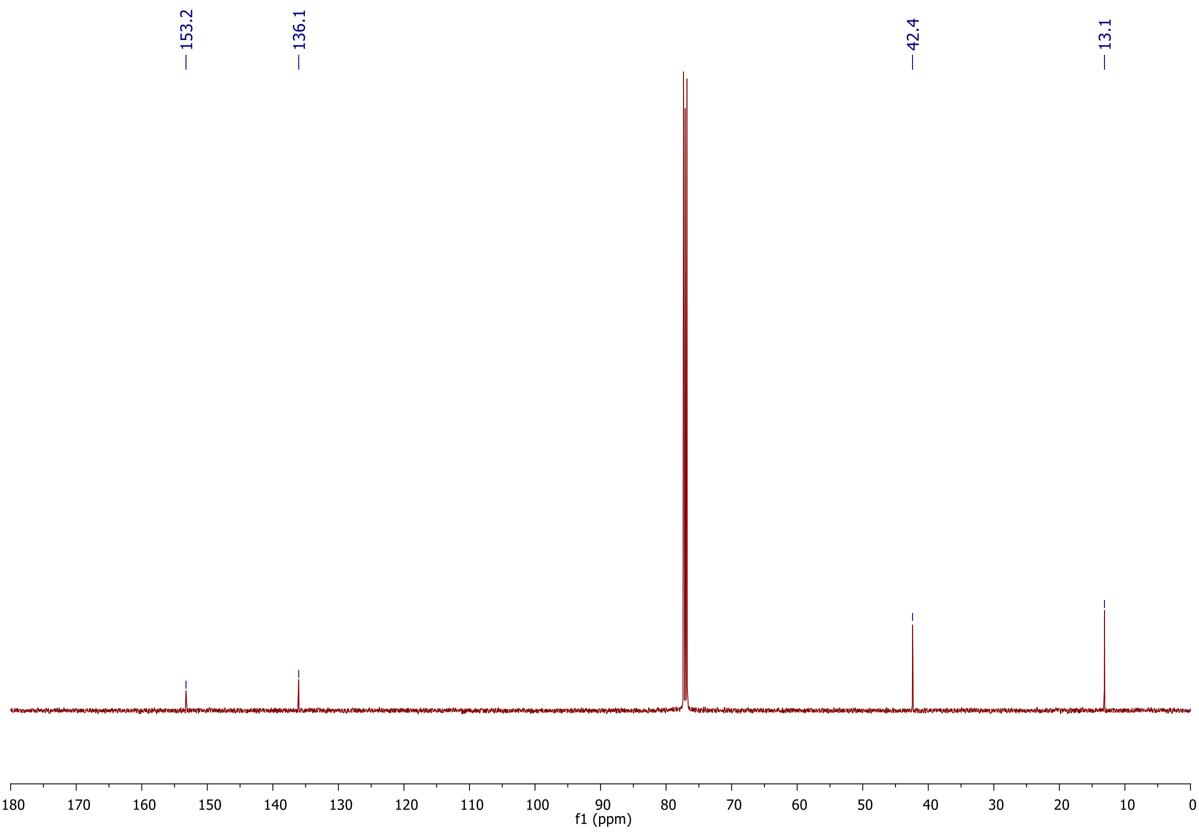
153.2
136.1
42.4
13.1
180 170 160 150 140 130 120 110 100 90 80 70 60 50 40 30 20 10 0
f1 (ppm)

4-Bromo-1,2-diethyl-5-(hexylthio)-1,2-dihydropyridazine-3,6-dione 2

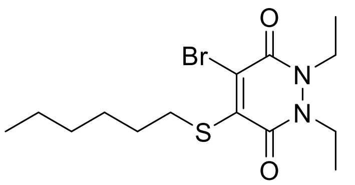

2

To a solution of NaOAc (123 mg, 1.50 mmol, pre-dissolved in MeOH (10 mL)) was added a solution of hexane-1-thiol (156 µL, 1.1 mmol) and the reaction stirred at 21 °C for 10 min. 4,5-Dibromo-1,2-diethyl-1,2-dihydropyridazine-3,6-dione **10** (326 mg, 1.00 mmol) was then added and the reaction stirred at 21 °C for a further 16 h. After this time, MeOH was removed *in vacuo,* the crude residue was dissolved in EtOAc (30 mL) and then washed with water (3 × 10 mL). The organic layer was then dried ($MgSO_4$) and concentrated *in vacuo*. Purification of the crude residue by flash column chromatography (30% to 80% EtOAc/petrol) afforded 4-bromo-1,2-diethyl-5-(hexylthio)-1,2-dihydropyridazine-3,6-dione **2** (218 mg, 0.60 mmol, 60%) as a yellow oil. ^{1}H NMR (600 MHz, $CDCl_3$) δ 4.13 (q, *J* = 7.1 Hz, 2H), 4.08 (q, *J* = 7.1 Hz, 2H), 3.38 (t, *J* = 7.5 Hz, 2H), 1.63–1.59 (m, 2H), 1.42–1.40 (m, 2H), 1.30–1.23 (m, 10H), 0.87 (t, *J* = 7.0 Hz, 3H).; ^{13}C NMR (150 MHz, $CDCl_3$) δ 154.7 (C), 154.0 (C), 147.0 (C), 129.2 (C), 42.2 (CH_2), 41.3 (CH_2), 34.4 (CH_2), 31.4 (CH_2), 30.4 (CH_2), 28.4 (CH_2), 22.6 (CH_2), 14.1 (CH_3), 13.1 (CH_3), 12.8 (CH_3); IR (thin film) 2955, 2927, 2856, 1628, 1537 cm^{-1}. LRMS (ESI) 365 (100, [$M^{81}Br+H]^+$), 363 (100, [$M^{79}Br+H]^+$). HRMS (ESI) calcd for $C_{14}H_{24}$-$Br_1N_2O_2S$ [$M^{79}Br+H]^+$ 363.0742; observed 363.0741.

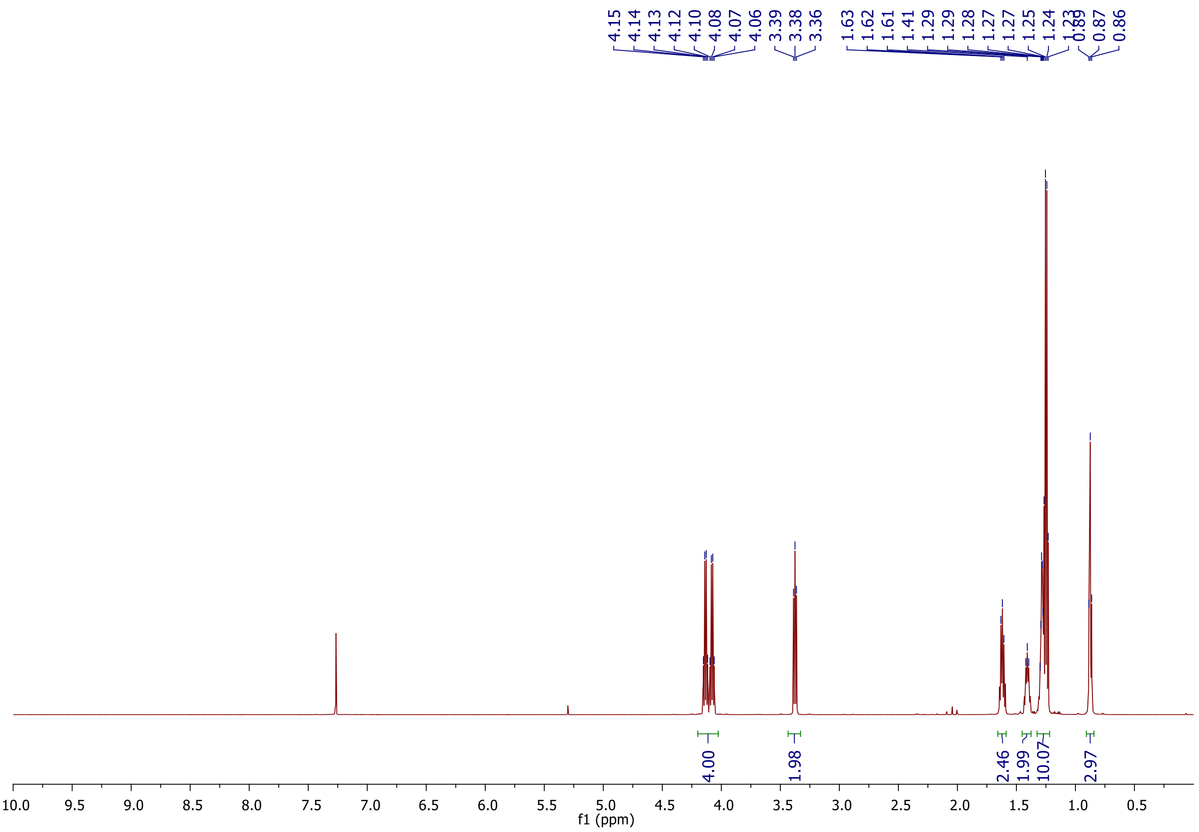
4.15
4.14
4.13
4.12
4.10
4.08
4.07
4.06
3.39
3.38
3.36
1.63
1.62
1.61
1.41
1.29
1.29
1.28
1.27
1.27
1.25
1.24
1.23
0.89
0.87
0.86
4.00
1.98
2.46
1.99
10.07
2.97
f1 (ppm)

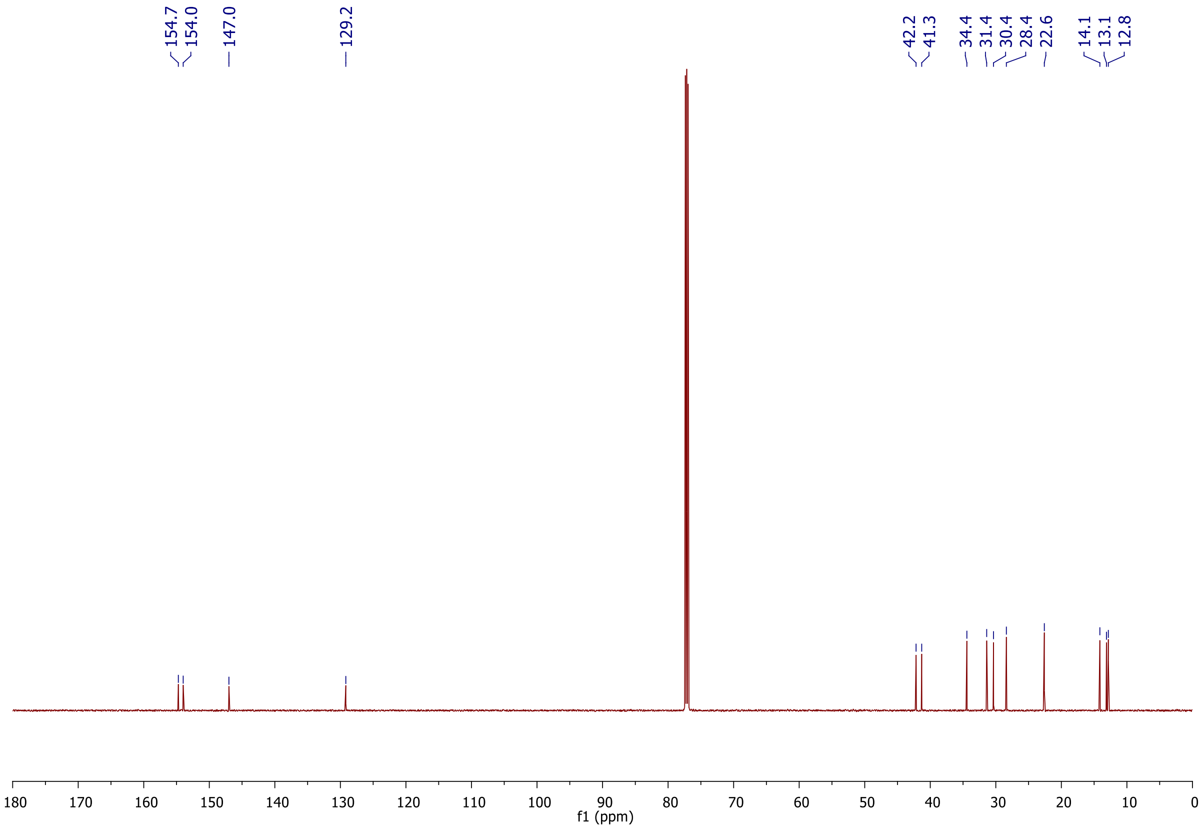
154.7
154.0
147.0
129.2
42.2
41.3
34.4
31.4
30.4
28.4
22.6
14.1
13.1
12.8
f1 (ppm)

4-Bromo-1,2-diethyl-5-((2-hydroxyethyl)thio)-1,2-dihydropyridazine-3,6-dione 3

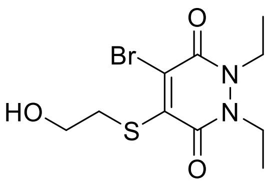

3

To a solution of NaOAc (123 mg, 1.50 mmol) pre-dissolved in MeOH (10 mL), was added 2-mercaptoethan-1-ol (77 µL, 1.1 mmol) and the reaction was stirred at 21 °C for 10 min. 4,5-Dibromo-1,2-diethyl-1,2-dihydropyridazine-3,6-dione **10** (326 mg, 1.00 mmol) was then added and the reaction stirred at 21 °C for a further 16 h. After this time, MeOH was removed *in vacuo,* the crude residue was dissolved in EtOAc (30 mL) and then washed with water (3 × 10 mL). The organic layer was then dried ($MgSO_4$) and concentrated *in vacuo*. Purification of the crude residue by flash column chromatography (30% to 80% EtOAc/petrol) afforded 4-bromo-1,2-diethyl-5-((2-hydroxyethyl)thio)-1,2-dihydropyridazine-3,6-dione **3** (229 mg, 0.71 mmol, 71%) as a yellow solid. m.p. 90–94 °C. ^1H NMR (600 MHz, $CDCl_3$) δ 4.14 (q, *J* = 7.1 Hz, 2H), 4.09 (q, *J* = 7.1 Hz, 2H), 3.84 (t, *J* = 5.6 Hz, 2H), 3.53 (t, *J* = 5.7 Hz, 2H), 1.28–1.24 (m, 6H); ^{13}C NMR (150 MHz, $CDCl_3$) δ 154.8 (C), 153.7 (C), 145.7 (C), 131.1 (C), 62.2 (CH_2), 42.3 (CH_2), 41.6 (CH_2), 37.0 (CH_2), 13.2 (CH_3), 12.9 (CH_2); IR (solid) 3418, 2976, 2935, 2873, 1608, 1536 cm^{-1}. LRMS (ESI) 325 (100, $[M^{81}Br+H]^+$), 323 (100, $[M^{79}Br+H]^+$). HRMS (ESI) calcd for $C_{10}H_{16}Br_1N_2O_3S$ $[M^{79}Br+H]^+$ 323.0065; observed 323.0066.

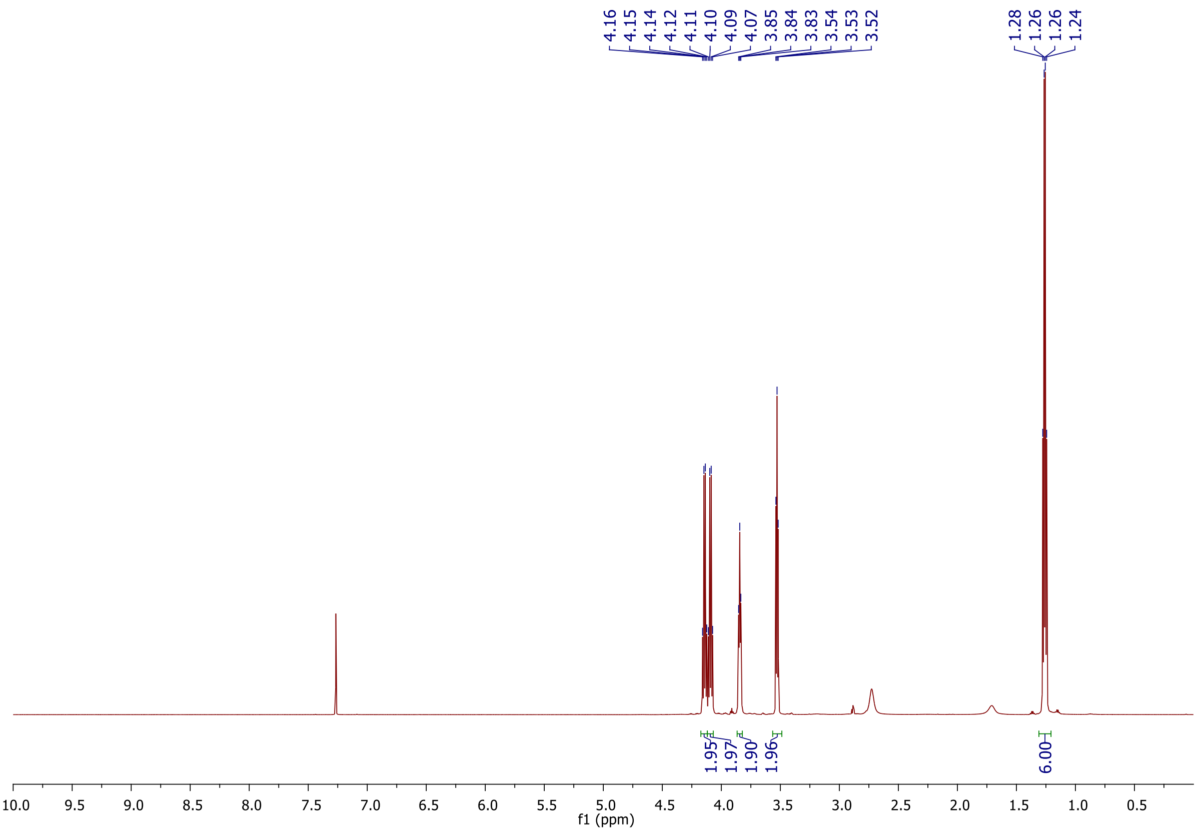
4.16 4.15 4.14 4.12 4.11 4.10 4.09 4.07 3.85 3.84 3.83 3.54 3.53 3.52
1.28 1.26 1.26 1.24
1.95 1.97 1.90 1.96
6.00
10.0 9.5 9.0 8.5 8.0 7.5 7.0 6.5 6.0 5.5 5.0 4.5 4.0 3.5 3.0 2.5 2.0 1.5 1.0 0.5
f1 (ppm)

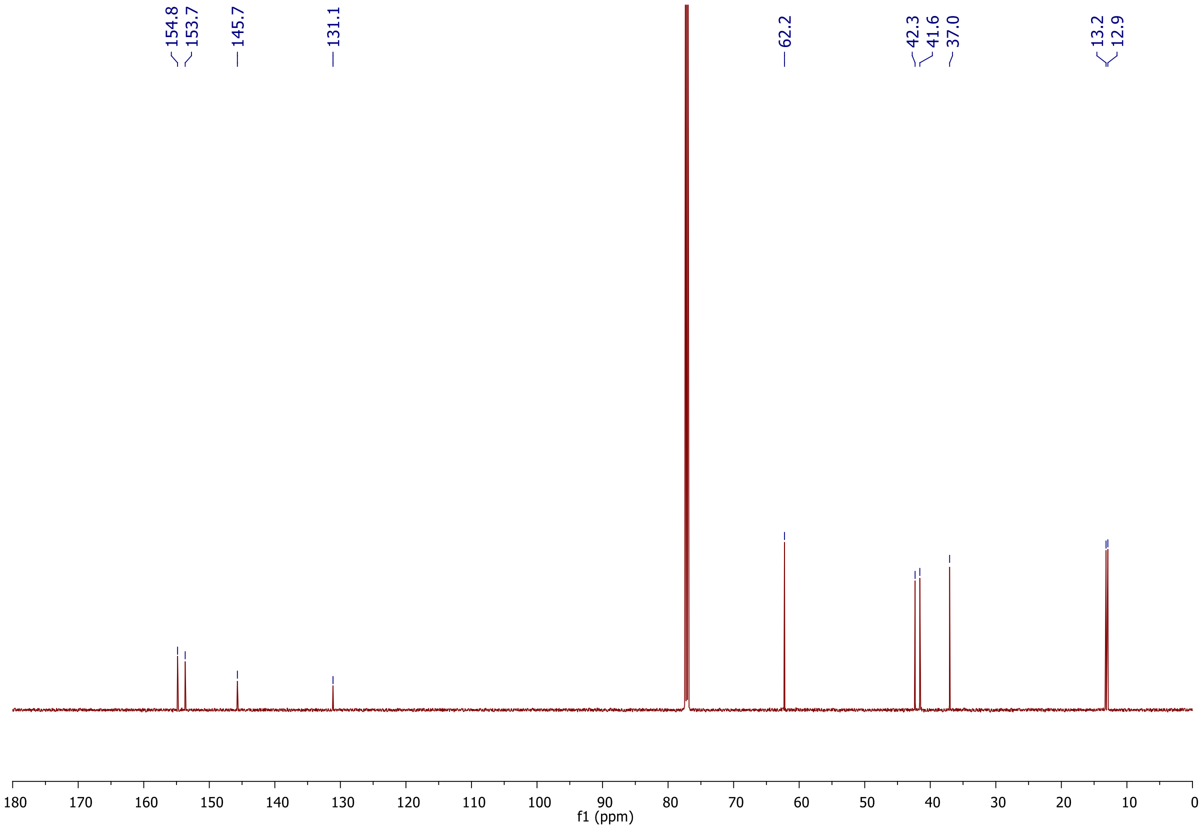
154.8 153.7 145.7 131.1
62.2
42.3 41.6 37.0
13.2 12.9
180 170 160 150 140 130 120 110 100 90 80 70 60 50 40 30 20 10 0
f1 (ppm)

3-((5-Bromo-1,2-diethyl-3,6-dioxo-1,2,3,6-tetrahydropyridazin-4-yl)thio)propanoic acid 4

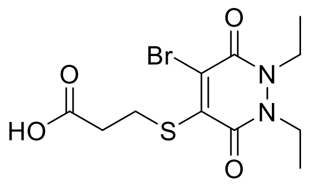

4

To a solution of NaOAc (123 mg, 1.50 mmol, pre-dissolved in MeOH (10 mL)) was added a solution of 10% 3-mercaptopropanoic acid (96 µL, 1.1 mmol) and the reaction was stirred at 21 °C for 10 min. 4,5-Dibromo-1,2-diethyl-1,2-dihydropyridazine-3,6-dione **10** (326 mg, 1.00 mmol) was then added and the reaction stirred at 21 °C for a further 16 h. After this time, MeOH was removed *in vacuo,* the crude residue was dissolved in EtOAc (30 mL) and then washed with water (3 × 10 mL). The organic layer was then dried ($MgSO_4$) and concentrated *in vacuo*. Purification of the crude residue by flash column chromatography (30% to 80% EtOAc/petrol) afforded 3-((5-bromo-1,2-diethyl-3,6-dioxo-1,2,3,6-tetrahydropyridazin-4-yl)thio)propanoic acid **4** (204 mg, 0.58 mmol, 58%) as a yellow solid. m.p. 143–145 °C; ^{1}H NMR (600 MHz, $CDCl_3$) δ 4.15 (q, *J* = 7.1 Hz, 2H), 4.10 (q, *J* = 7.1 Hz, 2H), 3.60 (t, *J* = 7.0 Hz, 2H), 2.82 (t, *J* = 7.0 Hz, 2H), 1.29–1.25 (m, 6H); ^{13}C NMR (150 MHz, $CDCl_3$) δ 176.0 (C), 154.5 (C), 153.7 (C), 145.8 (C), 129.7 (C), 42.3 (CH_2), 41.5 (CH_2), 35.4 (CH_2), 28.8 (CH_2), 13.2 (CH_3), 12.9 (CH_3); IR (solid) 3445, 2978, 2937, 1729, 1607 cm^{-1}. LRMS (ESI) 353 (100, $[M^{81}Br+H]^+$), 351 (100, $[M^{79}Br+H]^+$). HRMS (ESI) calcd for $C_{11}H_{16}Br_1N_2O_4$ $[M^{79}Br+H]^+$ 351.0014; observed 351.0013.

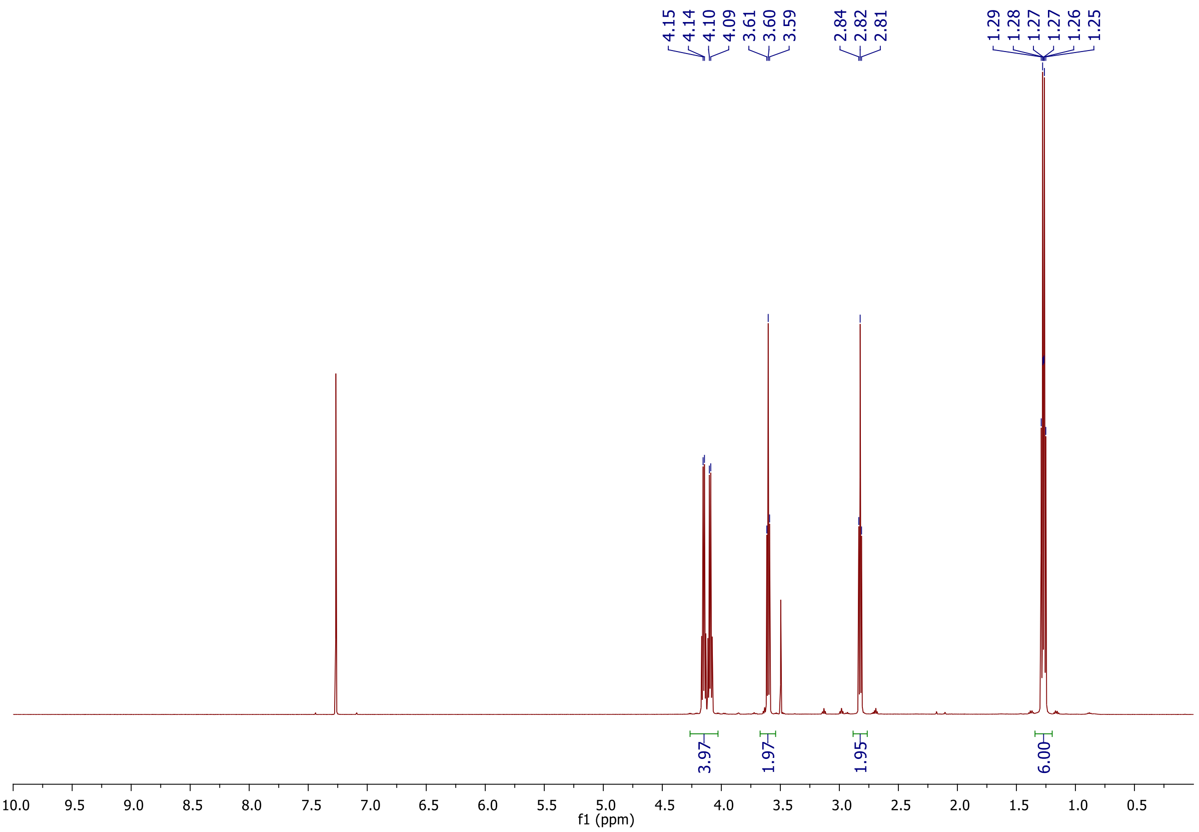
4.15
4.14
4.10
4.09
3.61
3.60
3.59
2.84
2.82
2.81
1.29
1.28
1.27
1.27
1.26
1.25
3.97
1.97
1.95
6.00
10.0 9.5 9.0 8.5 8.0 7.5 7.0 6.5 6.0 5.5 5.0 4.5 4.0 3.5 3.0 2.5 2.0 1.5 1.0 0.5
f1 (ppm)

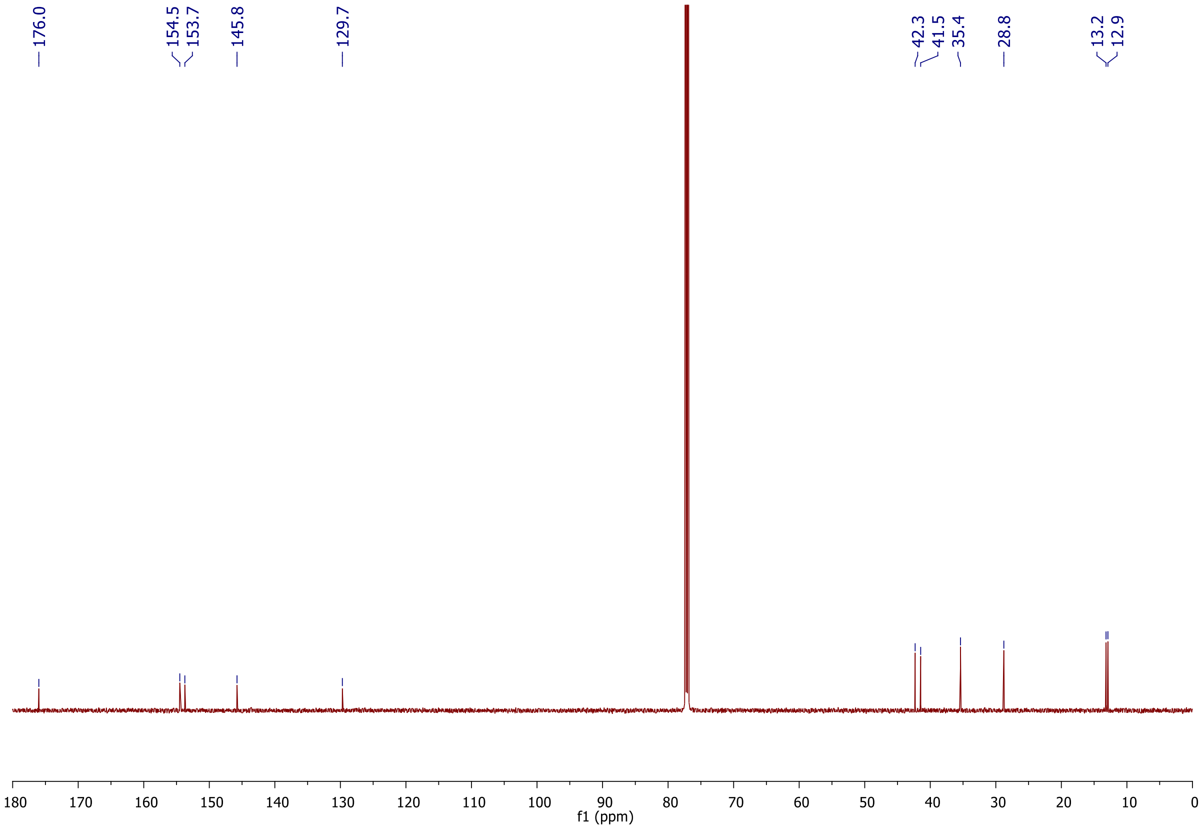
176.0
154.5
153.7
145.8
129.7
42.3
41.5
35.4
28.8
13.2
12.9
180 170 160 150 140 130 120 110 100 90 80 70 60 50 40 30 20 10 0
f1 (ppm)

4-Bromo-1,2-diethyl-5-(hexylamino)-1,2-dihydropyridazine-3,6-dione 9

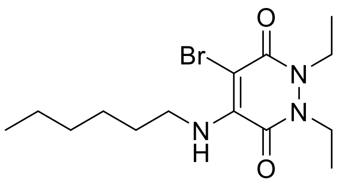

9

To a solution of NaOH (60 mg, 1.50 mmol, pre-dissolved in MeOH (10 mL)), was added a solution of hexan-1-amine (144 µL, 1.1 mmol) and the reaction stirred at 21 °C for 10 min. 4,5-Dibromo-1,2-diethyl-1,2-dihydropyridazine-3,6-dione **10** (326 mg, 1.00 mmol) was then added and the reaction stirred at 21 °C for a further 16 h. After this time, MeOH was removed *in vacuo*, the crude residue dissolved in EtOAc (30 mL) and then washed with water (3 × 10 mL). The organic layer was then dried ($MgSO_4$) and concentrated *in vacuo*. Purification of the crude residue by flash column chromatography (50% to 100% EtOAc/petrol) afforded 4-bromo-1,2-diethyl-5-(hexylamino)-1,2-dihydropyridazine-3,6-dione **9** (246 mg, 0.71 mmol, 71%) as a yellow oil. ^{1}H NMR (600 MHz, $CDCl_3$) δ 6.02 (s, 1H), 4.10–4.05 (m, 4H), 3.74–3.77 (m, 2H), 1.64–1.59 (m, 2H), 1.38–1.34 (m, 2H), 1.32–1.27 (m, 4H), 1.24 (t, *J* = 7.1 Hz, 3H), 1.15 (t, *J* = 7.1 Hz, 3H), 0.88 (t, *J* = 7.0 Hz, 3H).; ^{13}C NMR (150 MHz, $CDCl_3$) δ 158.6 (C), 154.4 (C), 142.8 (C), 44.5 (CH_2), 41.9 (CH_2), 41.5 (CH_2), 31.5 (CH_2), 31.0 (CH_2), 26.4 (CH_2), 22.7 (CH_2), 14.1 (CH_3), 12.9 (CH_3), 12.8 (CH_3); IR (thin-film) 3303, 2955, 2929, 2856, 1599, 1509 cm^{-1}. LRMS (ESI) 348 (100, $[M^{81}Br+H]^+$), 346 (100, $[M^{79}Br+H]^+$). HRMS (ESI) calcd for $C_{14}H_{25}Br_1N_3O_2$ $[M^{79}Br+H]^+$ 346.1125; observed 345.1124.

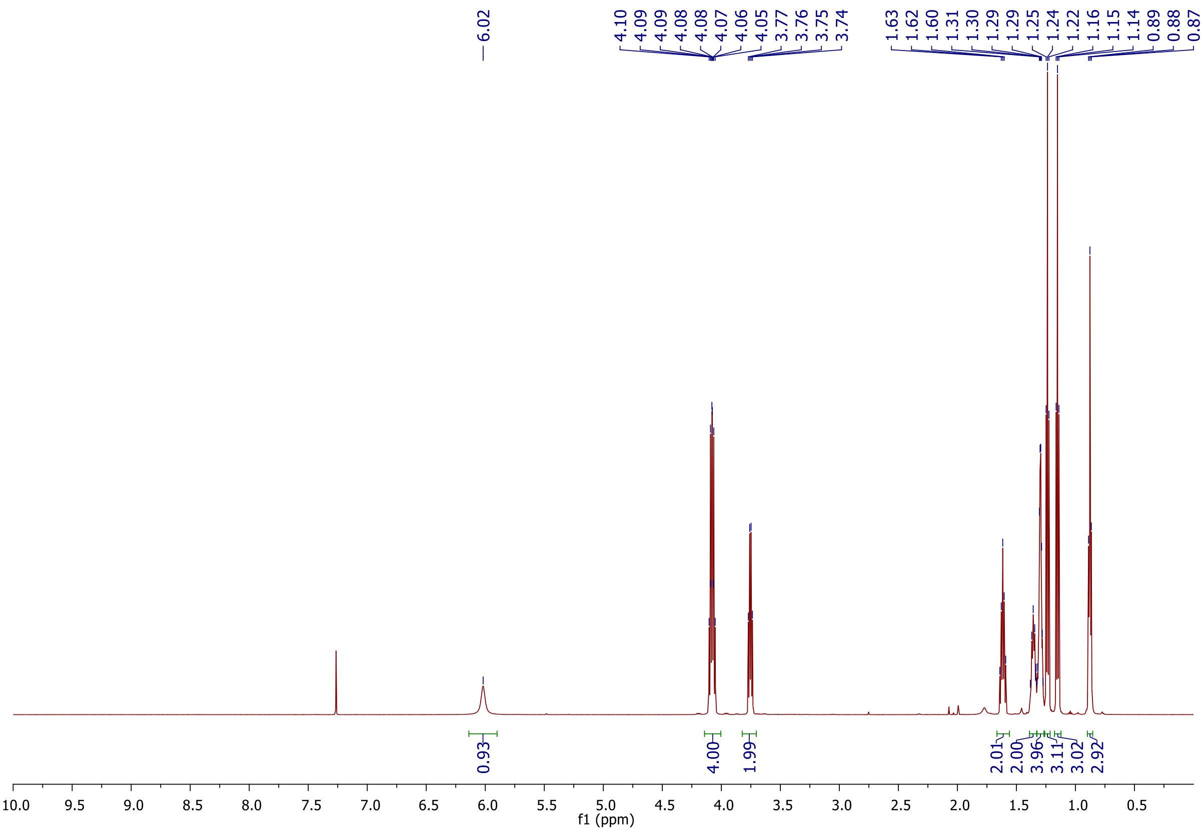
6.02
4.10
4.09
4.09
4.08
4.08
4.07
4.06
4.05
3.77
3.76
3.75
3.74
1.63
1.62
1.60
1.31
1.30
1.29
1.29
1.25
1.24
1.22
1.16
1.15
1.14
0.89
0.88
0.87
0.93
4.00
1.99
2.01
2.00
3.96
3.11
3.02
2.92
10.0 9.5 9.0 8.5 8.0 7.5 7.0 6.5 6.0 5.5 5.0 4.5 4.0 3.5 3.0 2.5 2.0 1.5 1.0 0.5
f1 (ppm)

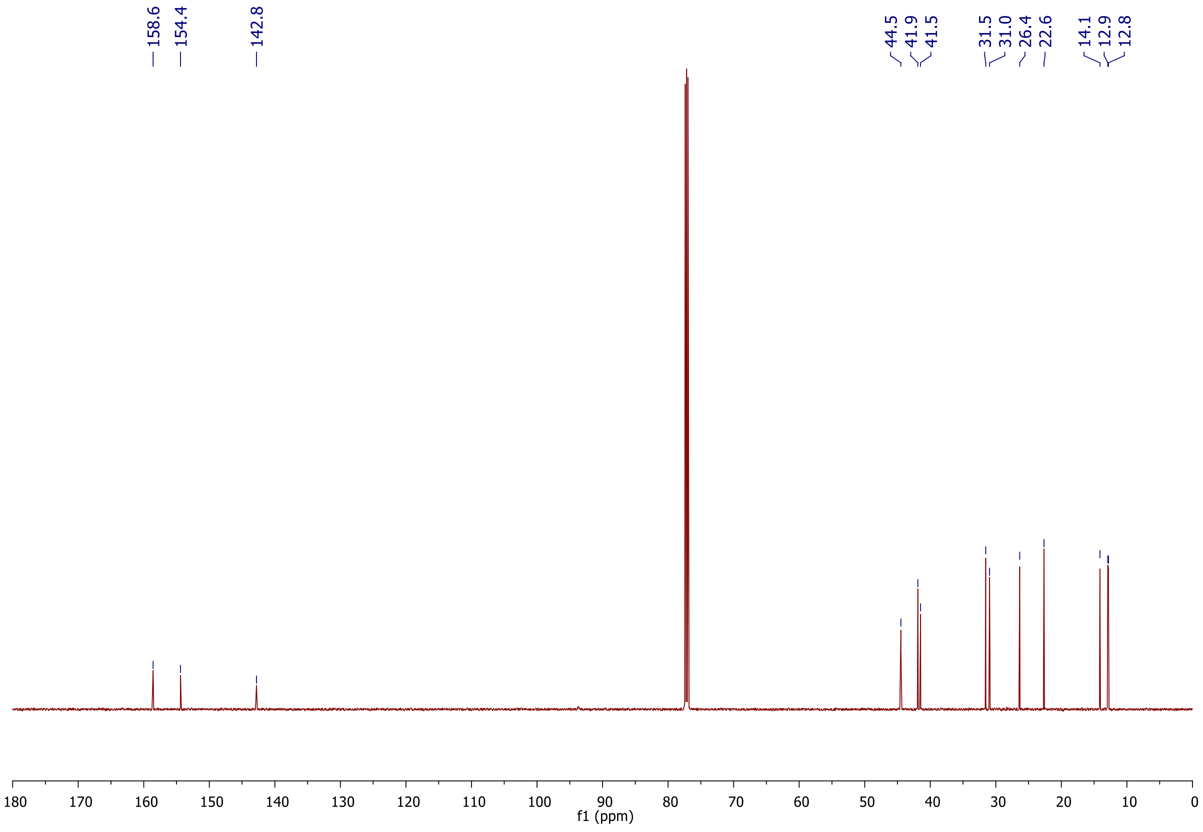
158.6
154.4
142.8
44.5
41.9
41.5
31.5
31.0
26.4
22.6
14.1
12.9
12.8
180 170 160 150 140 130 120 110 100 90 80 70 60 50 40 30 20 10 0
f1 (ppm)

Di-*tert*-butyl-1-methylhydrazine-1,2-dicarboxylate S2[2]

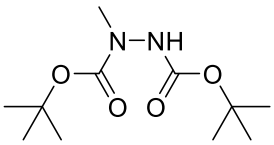

S2

To a solution of methyl hydrazine (1.14 mL, 21.7 mmol) in *i*-PrOH (16 mL), was added a solution of di-*tert*-butyl dicarbonate (11.4 g, 52.1 mmol, pre-dissolved in CH_2Cl_2 (12 mL)) drop-wise over 30 min. The reaction was then stirred at 21 °C for 16 h. After this time, the solvents were removed *in vacuo* and the crude residue purified by flash column chromatography (0% to 15% EtOAc/petrol) to afford di-*tert*-butyl-1-methylhydrazine-1,2-dicarboxylate **S2** (4.67 g, 19.1 mmol, 88%) as a white solid: m.p. 58–62 °C (*lit m.p.* 54–56 °C)[1]; ^{1}H NMR (600 MHz, $CDCl_3$, rotamers) δ 6.41–6.16 (m, 1H) 3.11 (s, 3H), 1.47–1.46 (m, 18H); ^{13}C NMR (150 MHz, $CDCl_3$, rotamers) δ 155.9 (C), 81.3 (C), 37.5 (CH_3), 28.3 (CH_3); IR (solid) 3315, 2981, 1702 cm^{-1}.

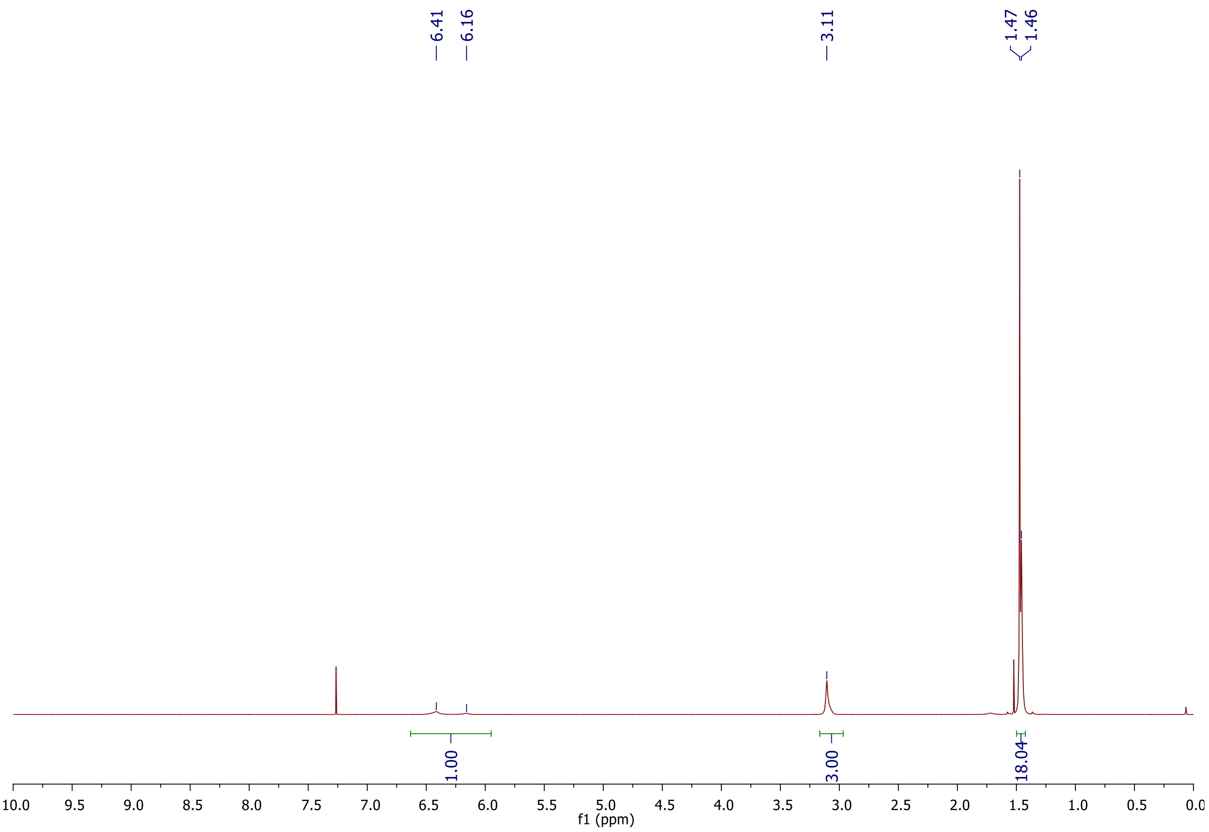

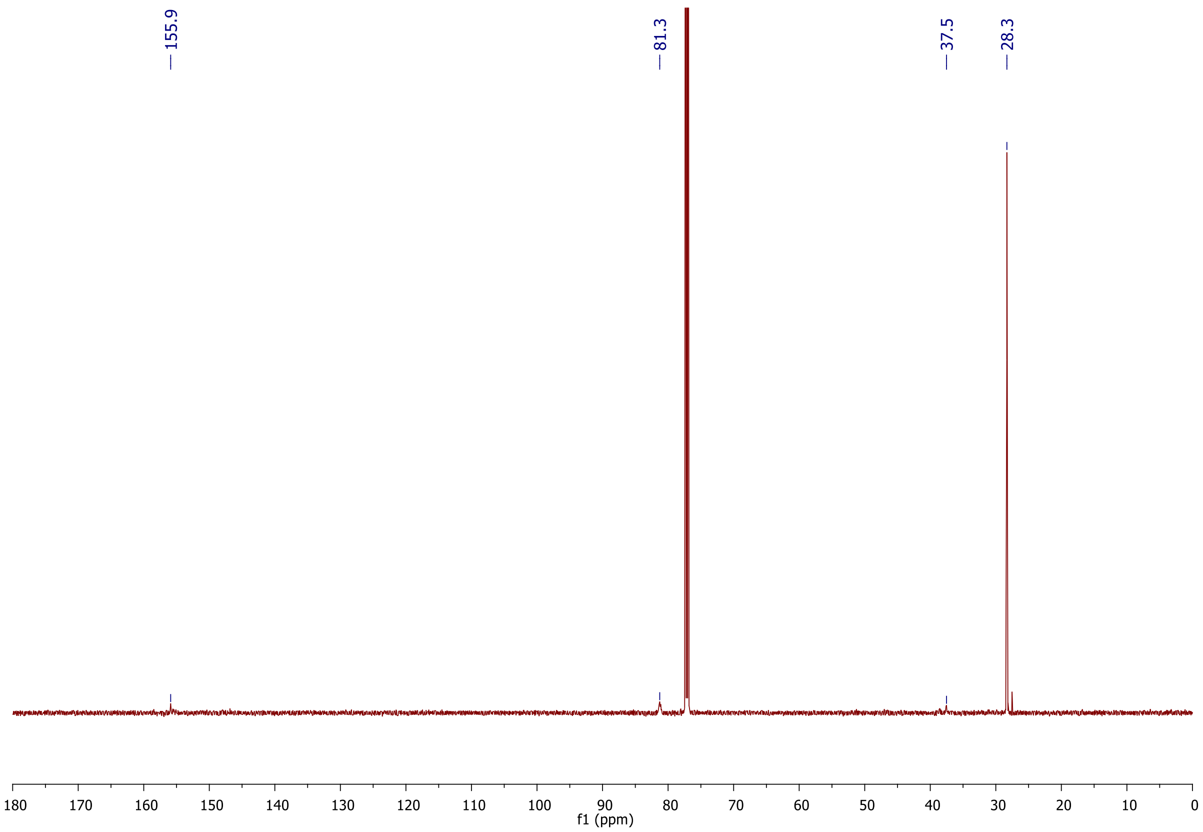
155.9
81.3
37.5
28.3
180
170
160
150
140
130
120
110
100
90
80
70
60
50
40
30
20
10
0
f1 (ppm)

Di-*tert*-butyl-1-(3-(*tert*-butoxy)-3-oxopropyl)-2-methylhydrazine-1,2-dicarboxylate S3[2]

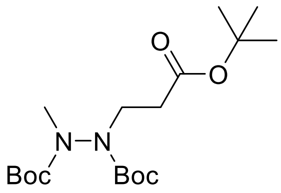

S3

To a solution of di-*tert*-butyl 1-methylhydrazine-1,2-dicarboxylate **S2** (3.00 g, 12.2 mmol) in *t*-BuOH (15 mL) was added 10% NaOH (0.5 mL) and the reaction mixture stirred at 21 °C for 10 min. After this, *tert*-butyl acrylate (5.31 mL, 36.6 mmol) was added to the solution and the reaction mixture was heated at 60 °C for 24 h. Following this, the solvent was removed *in vacuo* and the crude residue was dissolved in EtOAc (150 mL) and washed with water (3 × 50 mL). The organic layer was then dried ($MgSO_4$) and concentrated *in vacuo.* Purification of the crude residue by flash column chromatography (0% to 20% EtOAc/petrol) to afford di-*tert*-butyl-1-(3-(*tert*-butoxy)-3-oxopropyl)-2-methylhydrazine-1,2-dicarboxylate **S3** (3.33 g, 8.66 mmol, 71 %) as a clear oil: ^{1}H NMR (600 MHz, $CDCl_3$, rotamers) δ 3.85–3.52 (m, 2H), 3.06–2.99 (m, 3H), 2.51 (t, *J* = 7.2 Hz, 2H), 1.48–1.43 (m, 27H); ^{13}C NMR (150 MHz, $CDCl_3$, rotamers) δ 171.0 (C), 155.4 (C), 154.4 (C), 81.0 (C), 44.6 (CH_3), 36.6 (CH_2), 34.1 (CH_2), 28.3 (CH_3); IR (thin film) 2976, 2933, 1709 cm^{-1}; LRMS (ESI) 375 (100, $[M+H]^+$), 319 (30, $[M-C_4H_9+2H]^+$ HRMS (ESI) calcd for $C_{18}H_{35}N_2O_6$ $[M+H]^+$ 376.2524; observed 376.2516.

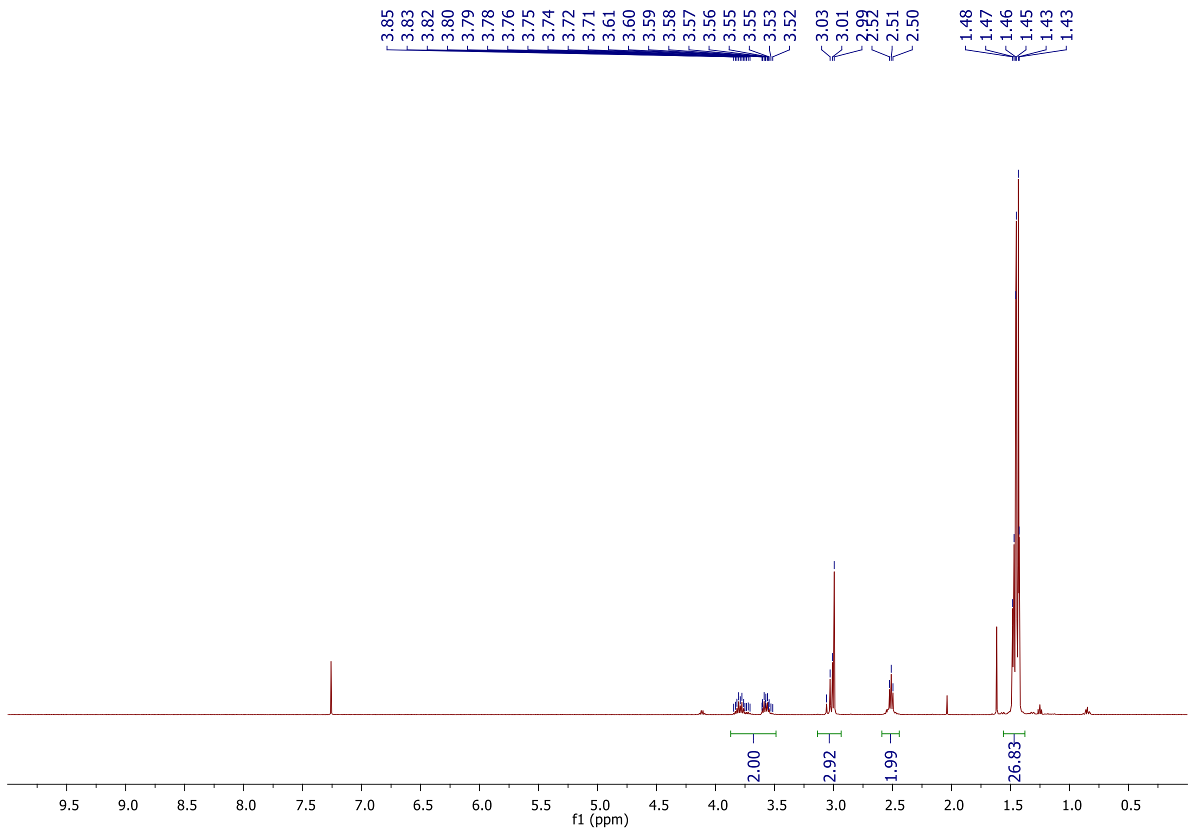
3.85 3.83 3.82 3.80 3.79 3.78 3.76 3.75 3.74 3.72 3.71 3.61 3.60 3.59 3.58 3.57 3.56 3.55 3.55 3.53 3.52
3.03 3.01 2.99 2.52 2.51 2.50
1.48 1.47 1.46 1.45 1.43 1.43
2.00
2.92
1.99
26.83
f1 (ppm)

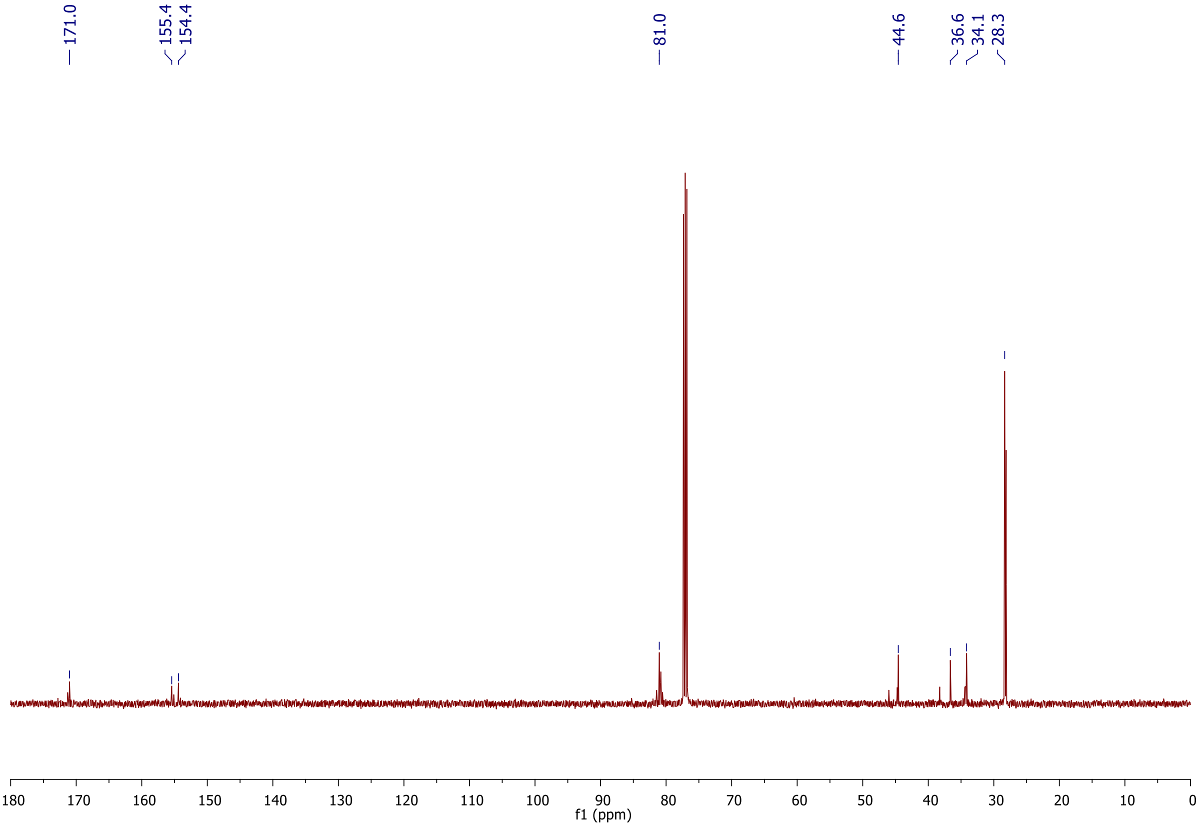
171.0
155.4
154.4
81.0
44.6
36.6
34.1
28.3
f1 (ppm)

3-(4,5-Dibromo-2-methyl-3,6-dioxo-3,6-dihydropyridazin-1(2*H*)-yl) propanoic acid S4[2]

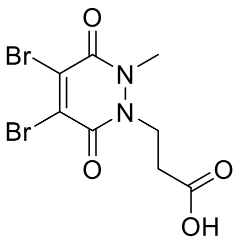

S4

Dibromomaleic acid (880 mg, 3.06 mmol) was dissolved in AcOH (25 mL) and heated under reflux for 30 min. To this solution, was added di-*tert*-butyl-1-(3-(*tert*-butoxy)-3-oxopropyl)-2-methylhydrazine-1,2-dicarboxylate **S3** (1.00 g, 2.67 mmol) and the reaction heated under reflux for a further 4 h. After this time, the reaction mixture was concentrated *in vacuo* with toluene co-evaporation (3 × 30 mL, as an azeotrope) and the crude residue purified by flash column chromatography (50% to 100% EtOAc/petrol (1% AcOH)) to afford 3-(4,5-dibromo-2-methyl-3,6-dioxo-3,6-dihydropyridazin-1(2*H*)-yl) propanoic acid **S4** (801 mg, 2.25 mmol, 84%) as a yellow solid: m.p. 140–144 °C; ^{1}H NMR (600 MHz, DMSO-d$_6$) δ 4.28 (t, *J* = 7.3 Hz, 2H), 3.56 (s, 3H), 2.63 (t, *J* = 7.3 Hz, 2H); ^{13}C NMR (150 MHz, DMSO-d$_6$) δ 171.9 (C), 152.7 (C), 152.4 (C), 135.3 (C), 135.0 (C), 43.1 (CH_3), 34.7 (CH_2), 31.7 (CH_2); IR (solid) 3044, 1725, 1606, 1570 cm^{-1} LRMS (ESI). 359 (50, [M^{81}Br^{81}Br+H]$^+$) 357 (100, [M^{79}Br^{81}Br+H]$^+$), 355 (50, [M^{79}Br^{79}Br+H]$^+$). HRMS (ESI) calcd for $C_8H_9Br_2N_2O_4$ [M^{81}Br^{81}Br+H]$^+$ 358.8883; observed 358.8882.

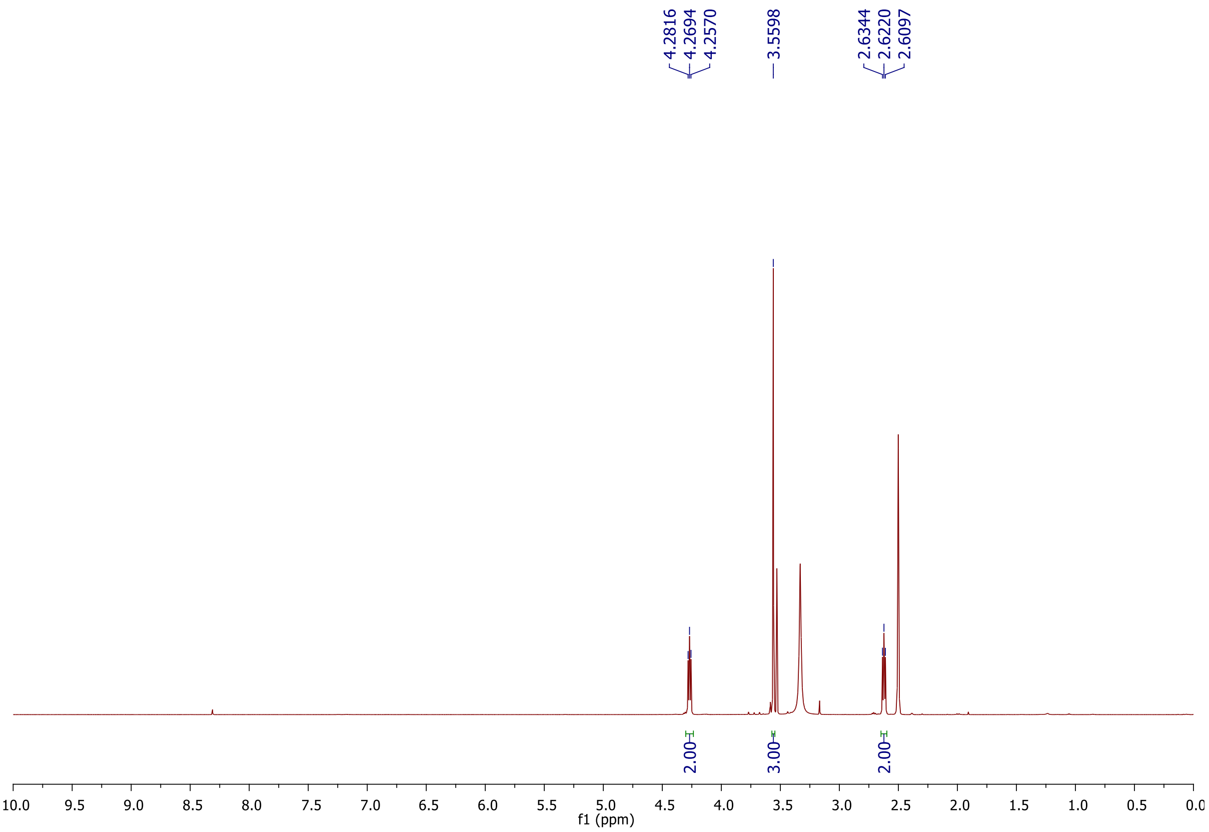
4.2816
4.2694
4.2570
3.5598
2.6344
2.6220
2.6097
2.00
3.00
2.00
10.0 9.5 9.0 8.5 8.0 7.5 7.0 6.5 6.0 5.5 5.0 4.5 4.0 3.5 3.0 2.5 2.0 1.5 1.0 0.5
f1 (ppm)

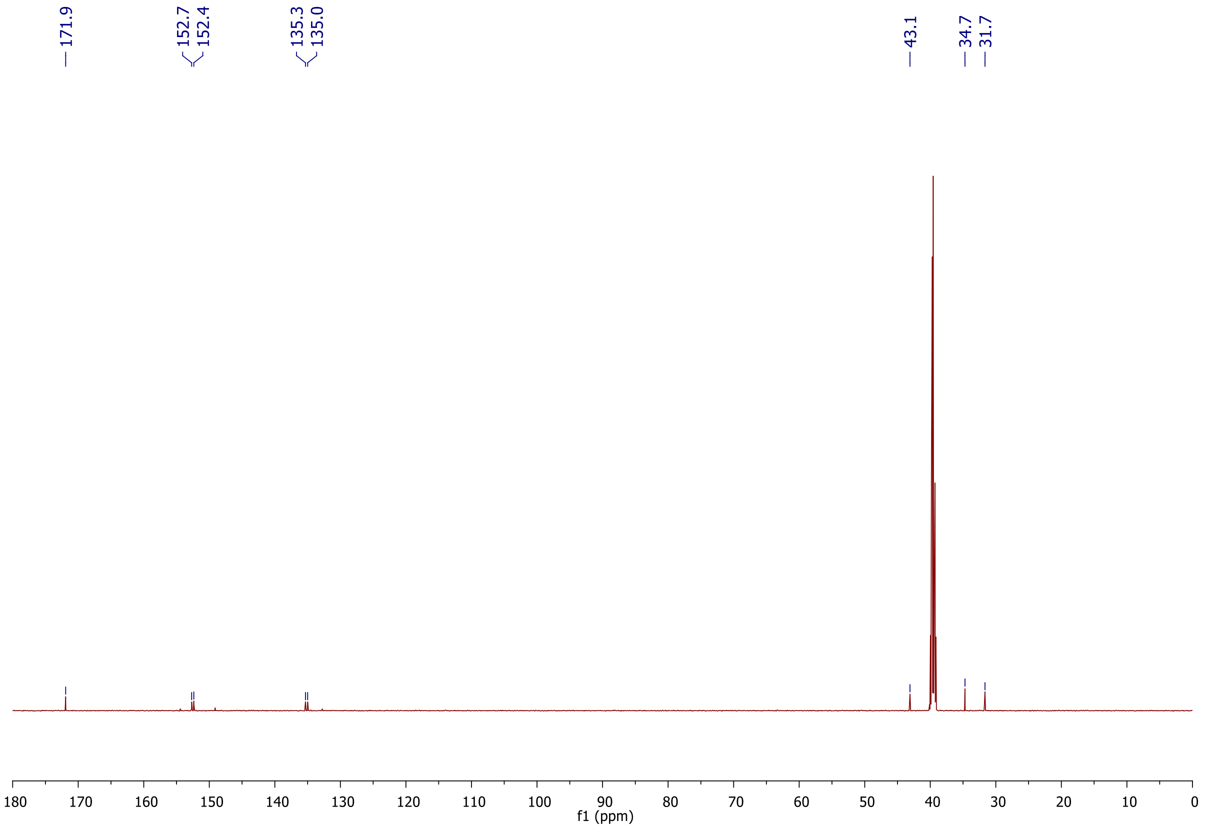
171.9
152.7
152.4
135.3
135.0
43.1
34.7
31.7
180 170 160 150 140 130 120 110 100 90 80 70 60 50 40 30 20 10 0
f1 (ppm)

2,5-Dioxopyrrolidin-1-yl 3-(4,5-dibromo-2-methyl-3,6-dioxo-3,6-dihydropyridazin-1(2*H*)-yl) propanoate S5[2]

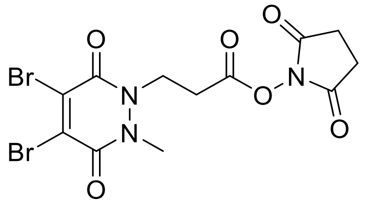

S5

To a solution of 3-(4,5-dibromo-2-methyl-3,6-dioxo-3,6-dihydropyridazin-1(2*H*)-yl) propanoic acid **S4** (250 mg, 0.702 mmol) in THF (10 mL) cooled to 0 °C, was added *N,N'*-dicyclohexylcarbodiimide (160 mg, 0.774 mmol). The homogenous solution was then stirred at 0 °C for 30 min. After this time, was added *N*-hydroxysuccinimide (89.0 mg, 0.78 mmol) and the reaction stirred at 21 °C for a further 16 h. The newly formed heterogeneous mixture was then filtered and the filtrate concentrated *in vacuo*. Purification of the crude residue by flash column chromatography (20% to 100% EtOAc/petrol) afforded 2,5-dioxopyrrolidin-1-yl 3-(4,5-dibromo-2-methyl-3,6-dioxo-3,6-dihydropyridazin-1(2*H*)-yl) propanoate **S5** (230 mg, 0.507 mmol, 72%) as a yellow solid: m.p. 100–104 °C; ^{1}H NMR (600 MHz, $CDCl_3$) δ 4.48 (t, *J* = 6.9 Hz, 2H), 3.68 (s, 3H), 3.11 (t, *J* = 6.9 Hz, 2H), 2.85 (s, 4H); ^{13}C NMR (150 MHz, $CDCl_3$) δ 168.7 (C), 166.0 (C), 153.3 (C), 153.1 (C), 136.9 (C), 135.3 (C), 43.0 (CH_2), 35.3 (CH_3), 29.1 (CH_2), 25.7 (CH_2); IR (solid) 2992, 1814, 1782, 1735, 1634, 1576 cm^{-1}. LRMS (ESI) 358 (50, [M^{81}Br^{81}Br+H-succinimide]$^{+}$), 356 (100, [M^{81}Br^{79}Br+H-succinimide]$^{+}$), 354 (50, [M^{79}Br^{79}Br+H-succinimide]$^{+}$). Unable to obtain HRMS data due to decomposition of the NHS ester under mass spec conditions.

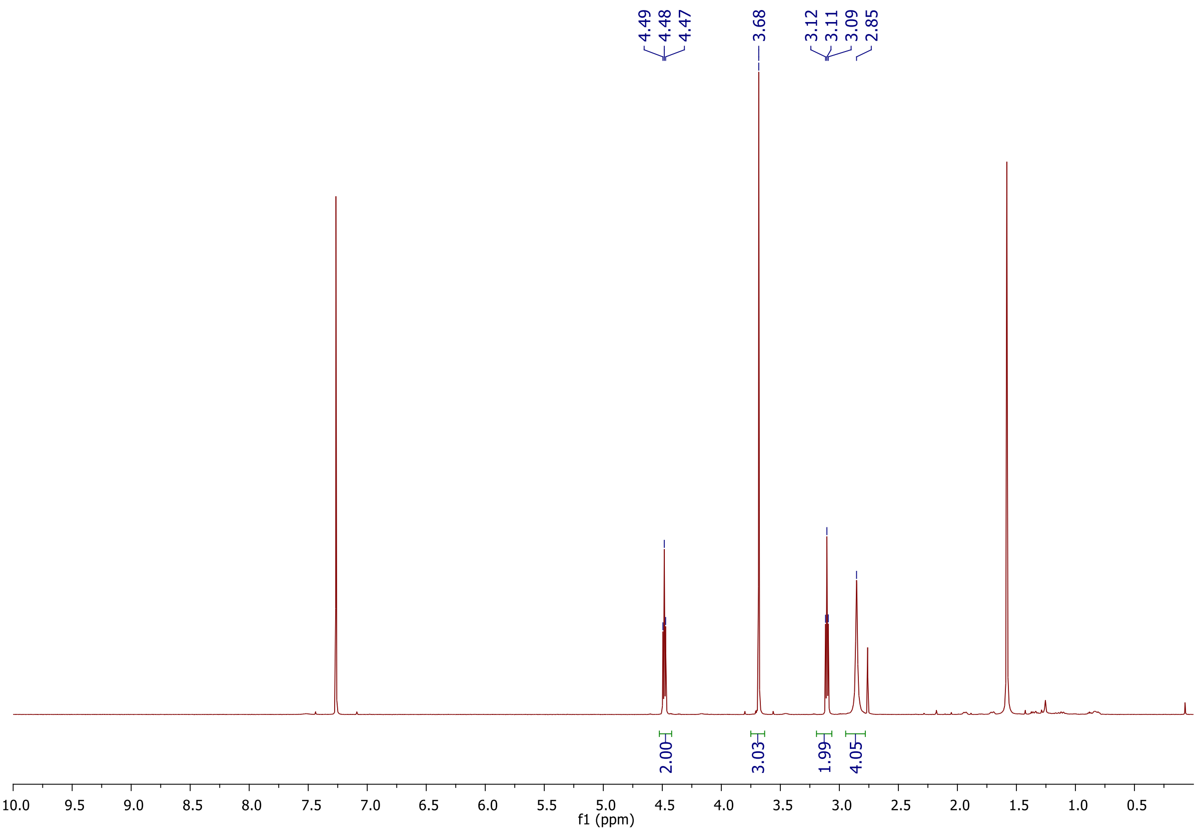
4.49
4.48
4.47
3.68
3.12
3.11
3.09
2.85
2.00
3.03
1.99
4.05
10.0 9.5 9.0 8.5 8.0 7.5 7.0 6.5 6.0 5.5 5.0 4.5 4.0 3.5 3.0 2.5 2.0 1.5 1.0 0.5
f1 (ppm)

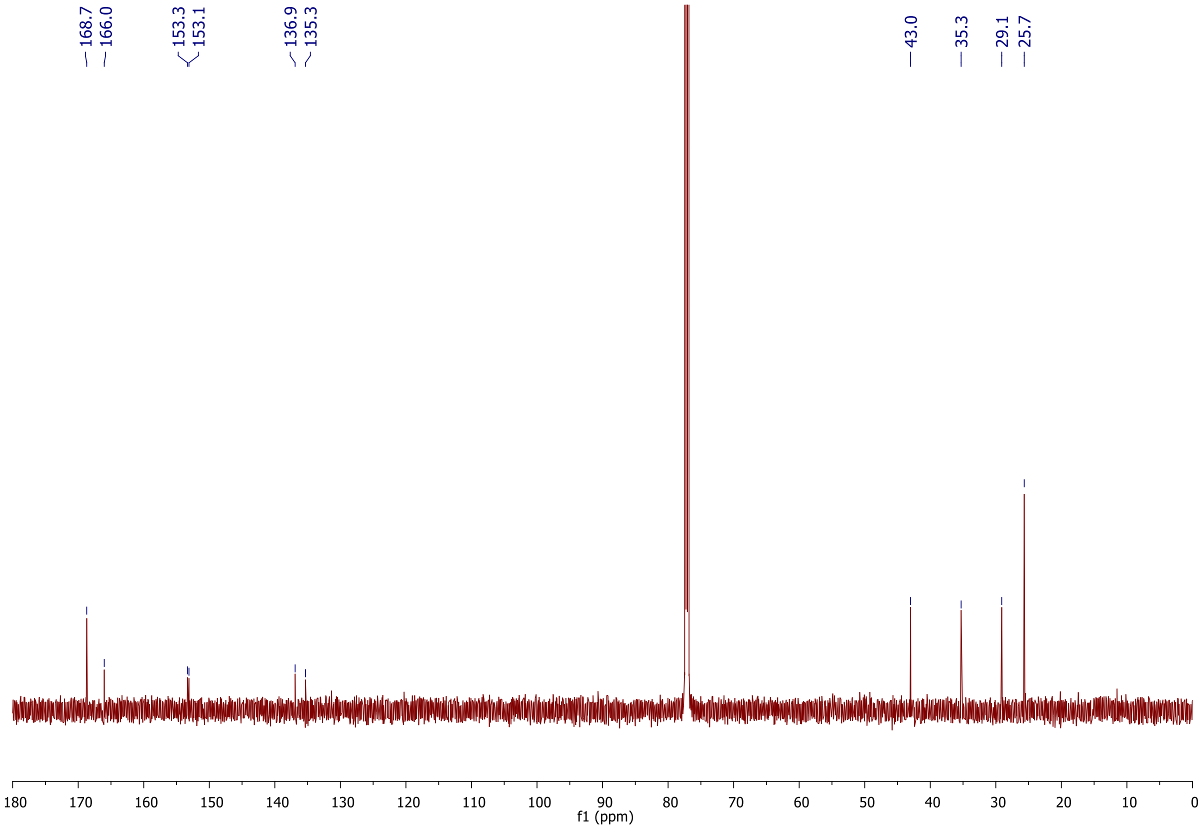
168.7
166.0
153.3
153.1
136.9
135.3
43.0
35.3
29.1
25.7
180 170 160 150 140 130 120 110 100 90 80 70 60 50 40 30 20 10 0
f1 (ppm)

((1*R*,8*S*,9*S*)-Bicyclo[6.1.0]non-4-yn-9-yl)methyl (2-(2-(2-(3-(4,5-dibromo-2-methyl-3,6-dioxo-3,6-dihydropyridazin-1(2*H*)-yl)propanamido)ethoxy)ethoxy)ethyl) carbamate S6[2]

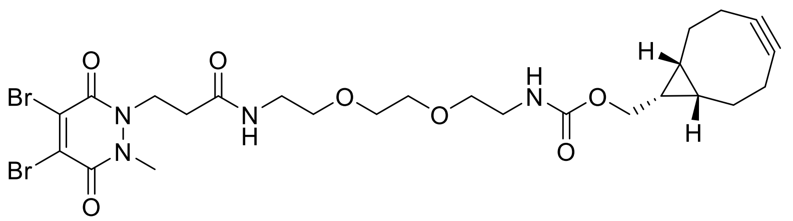

S6

To a solution of 2,5-dioxopyrrolidin-1-yl 3-(4,5-dibromo-2-methyl-3,6-dioxo-3,6-dihydropyridazin-1(2*H*)-yl) propanoate **S5** (100 mg, 0.221 mmol, pre-dissolved in MeCN (10 mL)), was added BCN amine (78.7 mg, 0.243 mmol) and the reaction mixture was stirred at 21 °C for 16 h. After this time, the reaction was concentrated *in vacuo* and the crude residue dissolved in $CHCl_3$ (50 mL) and washed with water (2 × 30 mL) and saturated aq. K_2CO_3 (30 mL). The organic layer was then dried ($MgSO_4$) and concentrated *in vacuo.* Purification of the crude residue by flash column chromatography (0% to 10% MeOH/EtOAc) afforded ((1*R*,8*S*,9*S*)-Bicyclo[6.1.0] non-4-yn-9-yl)methyl (2-(2-(2-(3-(4,5-dibromo-2-methyl-3,6-dioxo-3,6-dihydropyridazin-1(2*H*)-yl)propanamido)ethoxy)ethoxy)ethyl) carbamate **S6** (105 mg, 0.168 mmol, 72%) as a yellow oil: ^{1}H NMR (600 MHz, $CDCl_3$, rotamers) δ 7.84 (s, 0.3H), 6.38 (s, 0.7H), 5.78 (s, 0.3H), 5.24 (s, 0.7H), 4.44 (t, *J* = 6.6 Hz, 2H), 4.14–4.12 (m, 2H), 3.73–3.71 (m, 3H), 3.60–3.57 (m, 6H), 3.53–3.52 (m, 2H), 3.45–3.43 (m, 2H), 3.39–3.35 (m, 2H), 2.62 (t, *J* = 6.6 Hz, 2H), 2.29–2.20 (m, 6H), 1.61–1.57 (m, 2H), 1.35–1.32 (m, 1H), 0.96–0.94 (m, 2H); ^{13}C NMR (150 MHz, $CDCl_3$, rotamers) δ 169.1 (C), 156.9 (C), 153.1 (C), 153.0 (C), 136.4 (C), 135.5 (C), 98.9 (C), 70.4 (CH_2), 70.3 (CH_2), 69.7 (CH_2), 63.0 (CH_2), 44.6 (CH_2), 40.8 (CH_2), 39.5 (CH_2), 35.1 (CH_3), 34.1 (CH_2), 29.3 (CH_2), 29.2 (CH_2), 21.6 (CH_2), 20.2 (CH_2), 17.9 (CH), 14.3 (CH); IR (thin film) 3329, 2920, 2858, 1708, 1630, 1572, 1534 cm^{-1}; LRMS (ESI), 687 (50, [M^{81}Br^{81}Br+Na]$^+$) 685 (100, [M^{79}Br^{81}Br+Na]$^+$), 683 (50, [M^{79}Br^{79}Br+Na]$^+$), 663 (60, [M^{79}Br^{81}Br+H]$^+$); HRMS (ESI) calcd for $C_{25}H_{35}Br_2N_4O_7$ [M^{79}Br^{81}Br+H]$^+$ 663.0847; observed 663.0846.

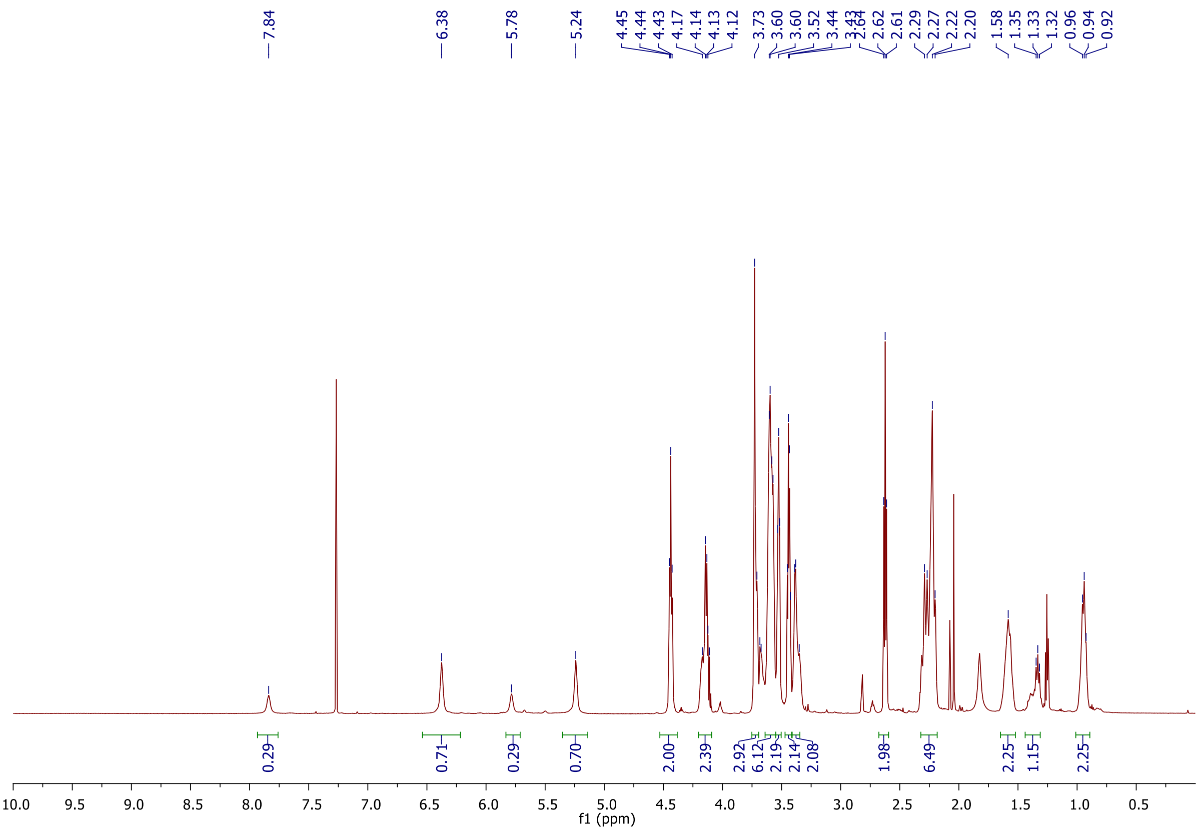
7.84
6.38
5.78
5.24
4.45
4.44
4.43
4.17
4.14
4.13
4.12
3.73
3.60
3.60
3.52
3.44
3.43
2.64
2.62
2.61
2.29
2.27
2.22
2.20
1.58
1.35
1.33
1.32
0.96
0.94
0.92
0.29
0.71
0.29
0.70
2.00
2.39
2.92
6.12
2.19
2.14
2.08
1.98
6.49
2.25
1.15
2.25
f1 (ppm)

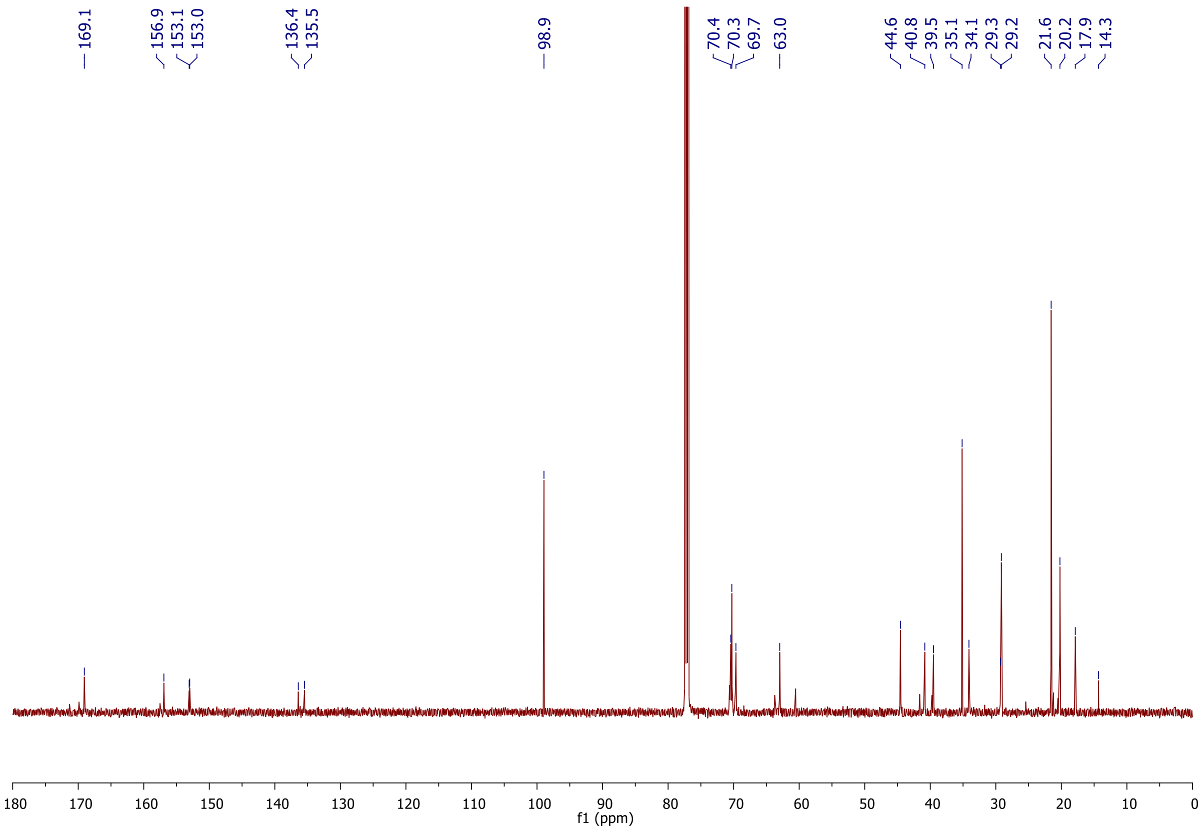
169.1
156.9
153.1
153.0
136.4
135.5
98.9
70.4
70.3
69.7
63.0
44.6
40.8
39.5
35.1
34.1
29.3
29.2
21.6
20.2
17.9
14.3
f1 (ppm)

tert-Butyl (4-cyanobenzyl)carbamate S7[3]

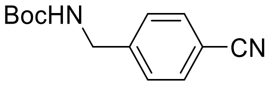

S7

To a solution of 4-(aminomethyl)benzonitrile (5.0 g, 29.7 mmol) in water (30 mL) was added a pre-dissolved solution of NaOH (3.6 g, 89.1 mmol) and di-*tert*-butyl dicarbonate (7.1 g, 32.6 mmol) in water (30 mL). The solution was stirred for 16 h at 21 °C, to form a heterogenous solution. After this time, the resulting solid was isolated, washed with water (100 mL), and dried solid to afford *tert*-butyl (4-cyanobenzyl)carbamate **S7** as a white solid (6.09 g, 26.2 mmol, 88%). ^{1}H NMR (400 MHz, $CDCl_3$) δ 7.62 (d, *J* = 8.3 Hz, 2H), 7.38 (d, *J* = 8.3 Hz, 2H), 4.96 (br s, 1H), 4.37 (d, *J* = 5.9 Hz, 2H), 1.46 (s, 9H); ^{13}C NMR (100 MHz, $CDCl_3$) δ 144.7 (C), 132.5 (C), 127.9 (C), 118.8 (C), 111.2 (C), 80.8 (C), 44.3 (CH_2), 28.4 (CH_3); IR (solid) 3350, 2974, 2927, 2226, 1692 cm^{-1}.

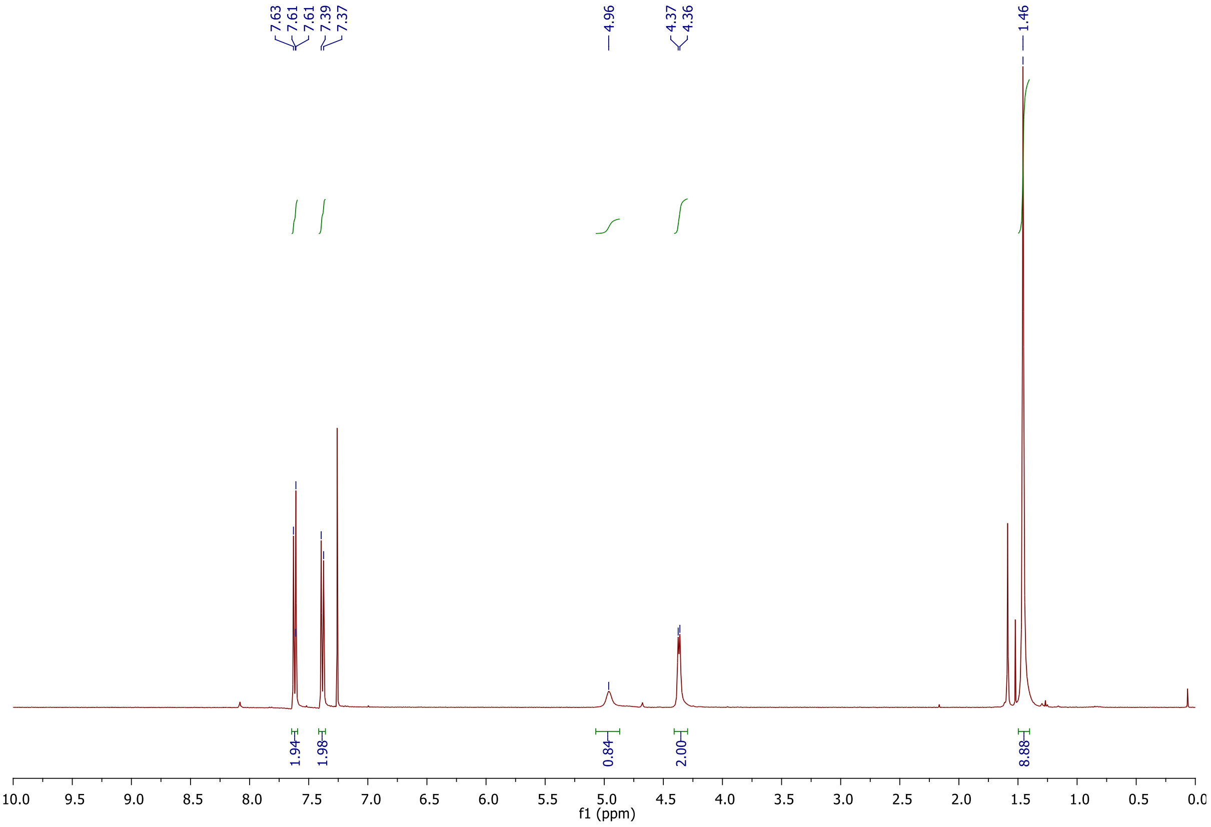

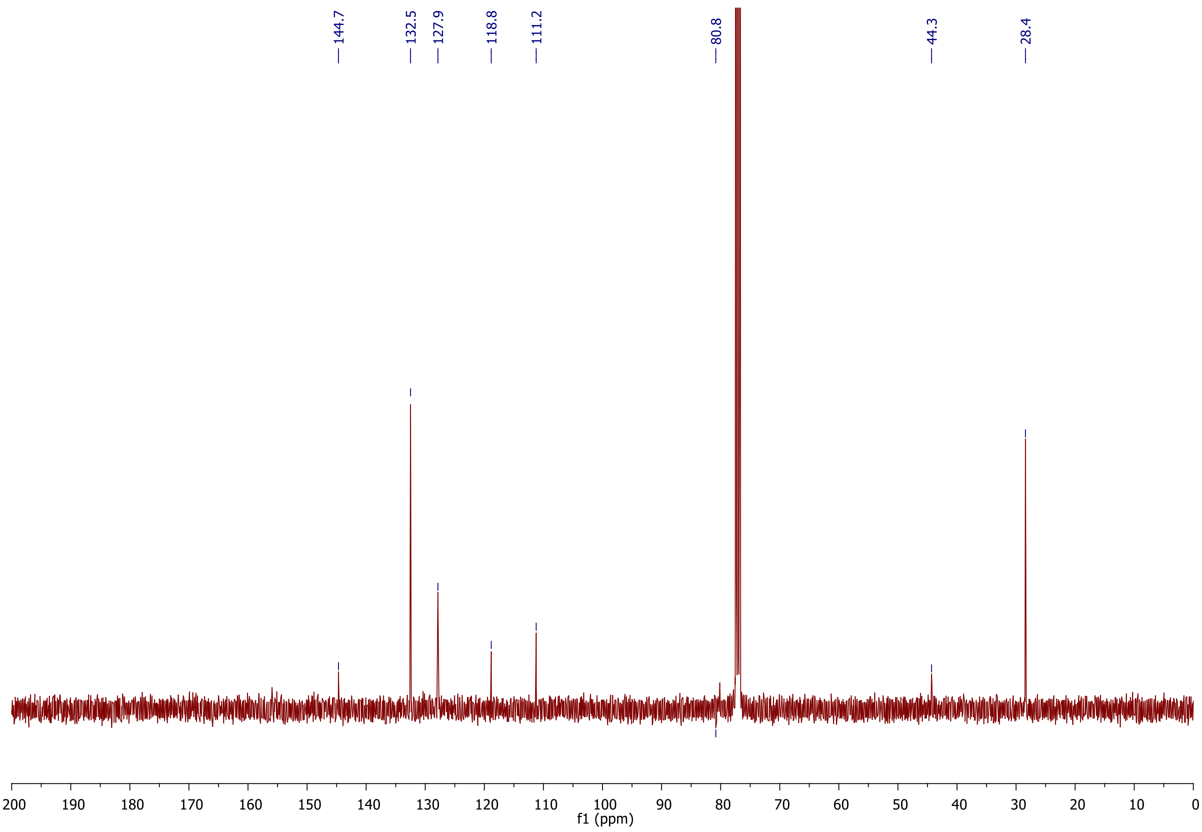
144.7
132.5
127.9
118.8
111.2
80.8
44.3
28.4
200 190 180 170 160 150 140 130 120 110 100 90 80 70 60 50 40 30 20 10 0
f1 (ppm)

tert-Butyl (4-(6-methyl-1,2,4,5-tetrazin-3-yl)benzyl)carbamate S8[3]

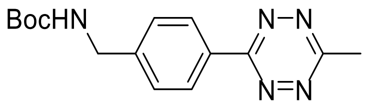

S8

To a solution of *tert*-butyl (4-cyanobenzyl)carbamate **S7** (3.0 g, 12.9 mmol), acetonitrile (6.72 mL, 12.9 mmol), and $Zn(OTf)_2$ (2.34 g, 6.46 mmol) in 1,4-dioxane (6 mL), was added hydrazine hydrate (80% w/w, 39.5 mL, 646 mmol) and the reaction was stirred at 65 °C for 72 h. After this time the reaction was diluted with EtOAc (50 mL), washed with 1 M HCl (50 mL) and the aqueous phase extracted with EtOAc (2 × 30 mL). The organic phase was dried ($MgSO_4$), and EtOAc was removed *in vacuo*. The resulting crude residue was dissolved in CH_2Cl_2 and AcOH (1:1, 200 mL), and $NaNO_2$ (17.8 g, 258 mmol) was added portion-wise over 15 min. The reaction was then diluted in CH_2Cl_2 (200 mL) and washed with sat. aqueous $NaHCO_3$ (200 mL). The product was extracted with CH_2Cl_2 (2 × 100 mL), dried ($MgSO_4$), and CH_2Cl_2 was removed *in vacuo*. The crude residue was purified by silica gel chromatography (20% EtOAc/petrol) to afford *tert*-butyl (4-(6-methyl-1,2,4,5-tetrazin-3-yl)benzyl)carbamate **S8** as a pink solid (1.07 g, 3.55 mmol, 28%). ^{1}H NMR (400 MHz, $CDCl_3$) δ 8.55 (d, *J* = 8.4 Hz, 2H), 7.50 (d, *J* = 8.3 Hz, 2H), 4.97 (br s, 1H), 4.44 (d, *J* = 5.8 Hz, 2H), 3.09 (s, 3H), 1.48 (s, 9H); ^{13}C NMR (100 MHz, $CDCl_3$) δ 167.3 (C), 164.0 (C), 144.0 (C), 130.9 (C), 128.3 (C), 128.1 (C), 80.1 (C), 28.5 (CH_3), 21.2 (CH_3); IR (solid) 3339, 2974, 2928, 1696, 1516 cm^{-1}.

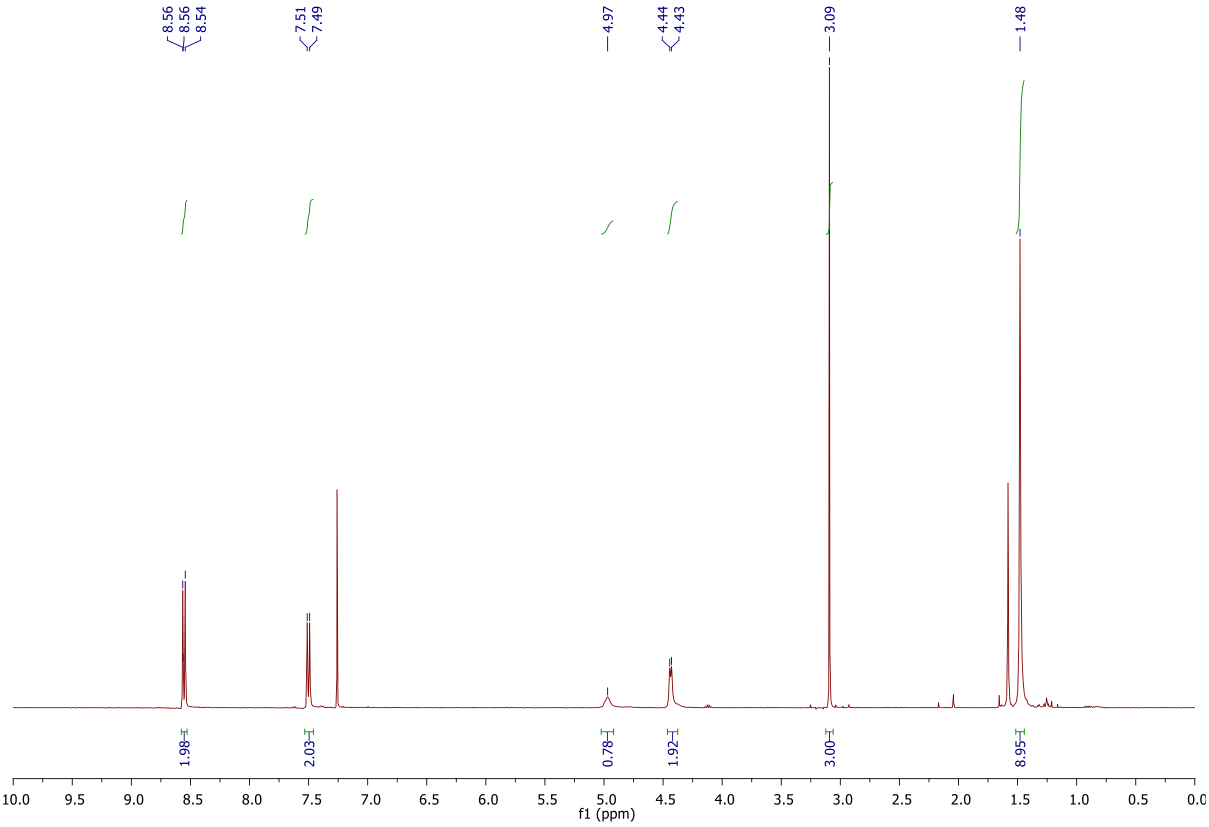
8.56
8.56
8.54
7.51
7.49
4.97
4.44
4.43
3.09
1.48
1.98
2.03
0.78
1.92
3.00
8.95
10.0 9.5 9.0 8.5 8.0 7.5 7.0 6.5 6.0 5.5 5.0 4.5 4.0 3.5 3.0 2.5 2.0 1.5 1.0 0.5 0.0
f1 (ppm)

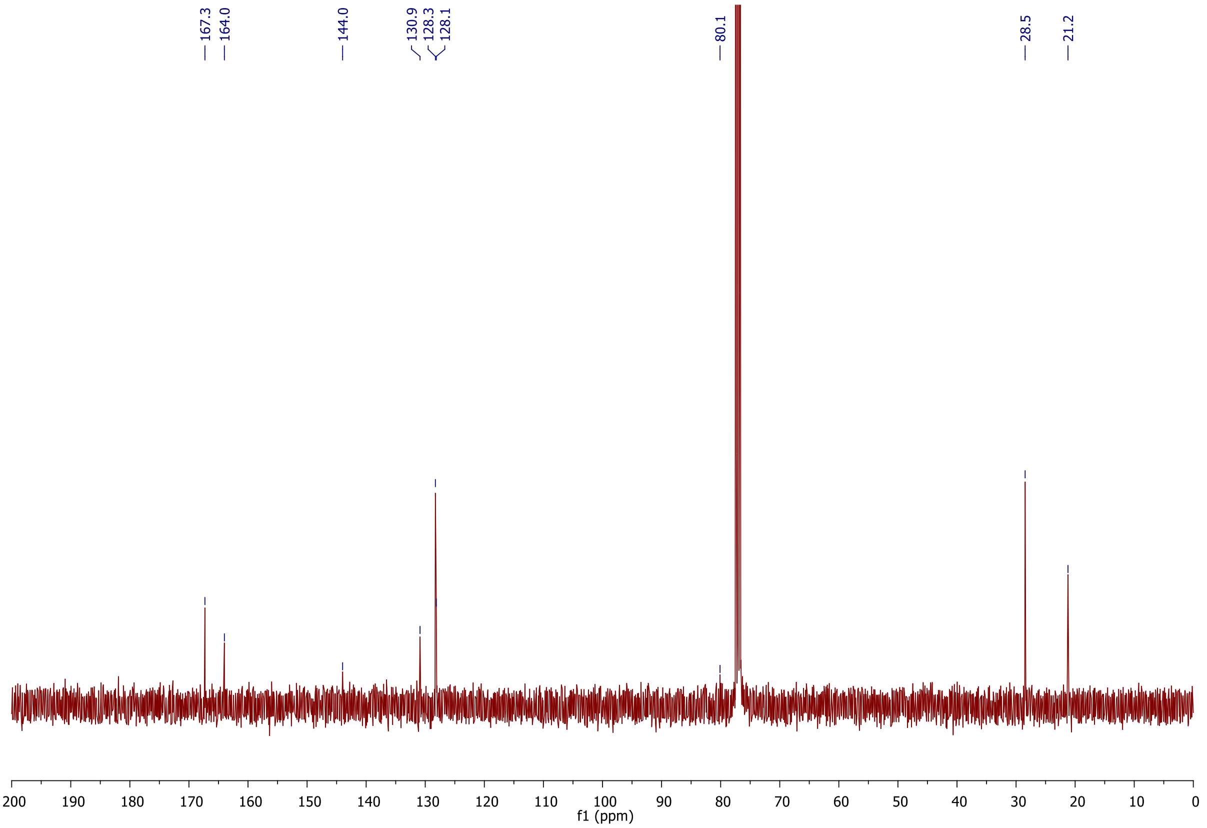
167.3
164.0
144.0
130.9
128.3
128.1
80.1
28.5
21.2
200 190 180 170 160 150 140 130 120 110 100 90 80 70 60 50 40 30 20 10 0
f1 (ppm)

5-((4-(6-Methyl-1,2,4,5-tetrazin-3-yl)benzyl)amino)-5-oxopentanoic acid S9[4]

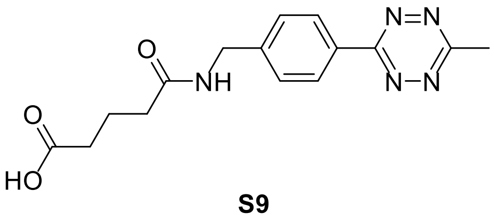

S9

To a solution of TFA and CH_2Cl_2 (1:4, 20 mL), was added *tert*-butyl (4-(6-methyl-1,2,4,5-tetrazin-3-yl)benzyl)carbamate **S8** (800 mg, 2.65 mmol) and the reaction was stirred at 21 °C for 2 h. After this time the TFA and CH_2Cl_2 was removed *in vacuo.* The crude residue was dissolved in THF (50 mL). To this, was added glutaric anhydride (605 mg, 5.31 mmol) and the reaction was stirred at 55 °C for a further 16 h. The THF was removed *in vacuo* and the crude residue was dissolved in sat. aqueous K_2CO_3 solution (100 mL). The product was then acidified with 15% HCl (20 mL) and extracted with EtOAc (3 × 50 mL). The product was then washed with water (4 × 30 mL) and brine (30 mL), dried ($MgSO_4$) and the EtOAc removed *in vacuo* to afford 5-((4-(6-methyl-1,2,4,5-tetrazin-3-yl)benzyl)amino)-5-oxopentanoic acid **S9** as a purple solid (691 mg, 2.2 mmol, 83%). ^{1}H NMR (400 MHz, $CDCl_3$) δ 8.41 (d, *J* = 8.4, 2H), 7.51 (d, *J* = 8.5 Hz, 2H), 4.38 (d, *J* = 6.0 Hz, 2H), 2.98 (s, 3H), 2.22 (q, *J* = 7.4 Hz, 4H), 1.76 (p, *J* = 7.4 Hz, 2 H); ^{13}C NMR (100 MHz, $CDCl_3$) δ 174.2 (C), 171.9 (C), 167.1 (C), 163.2 (C), 144.5 (C), 130.4 (C), 128.1 (C), 127.5 (C), 41.9 (CH_2), 34.4 (CH_2), 33.1 (CH_2), 20.8 (CH_3), 20.7 (CH_2); IR (solid) 3271, 3025, 2973, 2923, 2880, 1694, 1630, 1523 cm^{-1}.

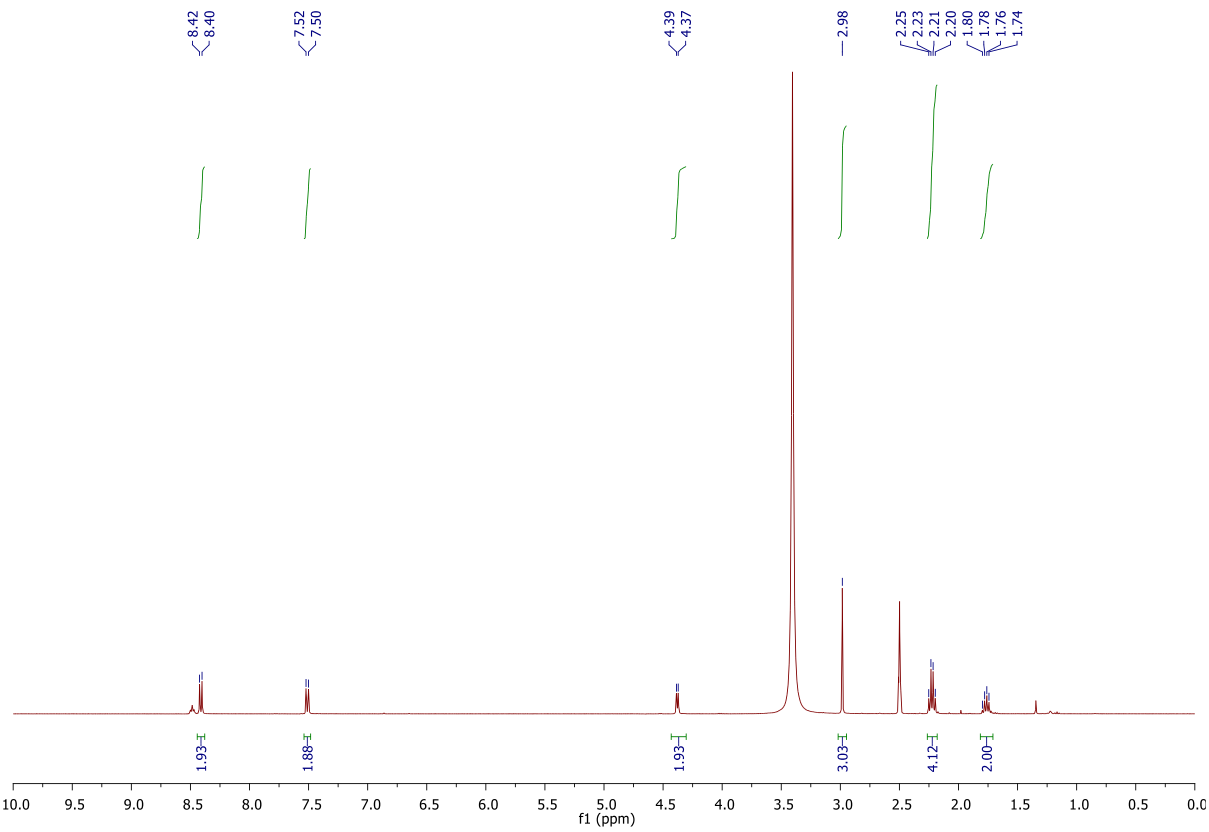
8.42
8.40
7.52
7.50
4.39
4.37
2.98
2.25
2.23
2.21
2.20
1.80
1.78
1.76
1.74
1.93
1.88
1.93
3.03
4.12
2.00
f1 (ppm)

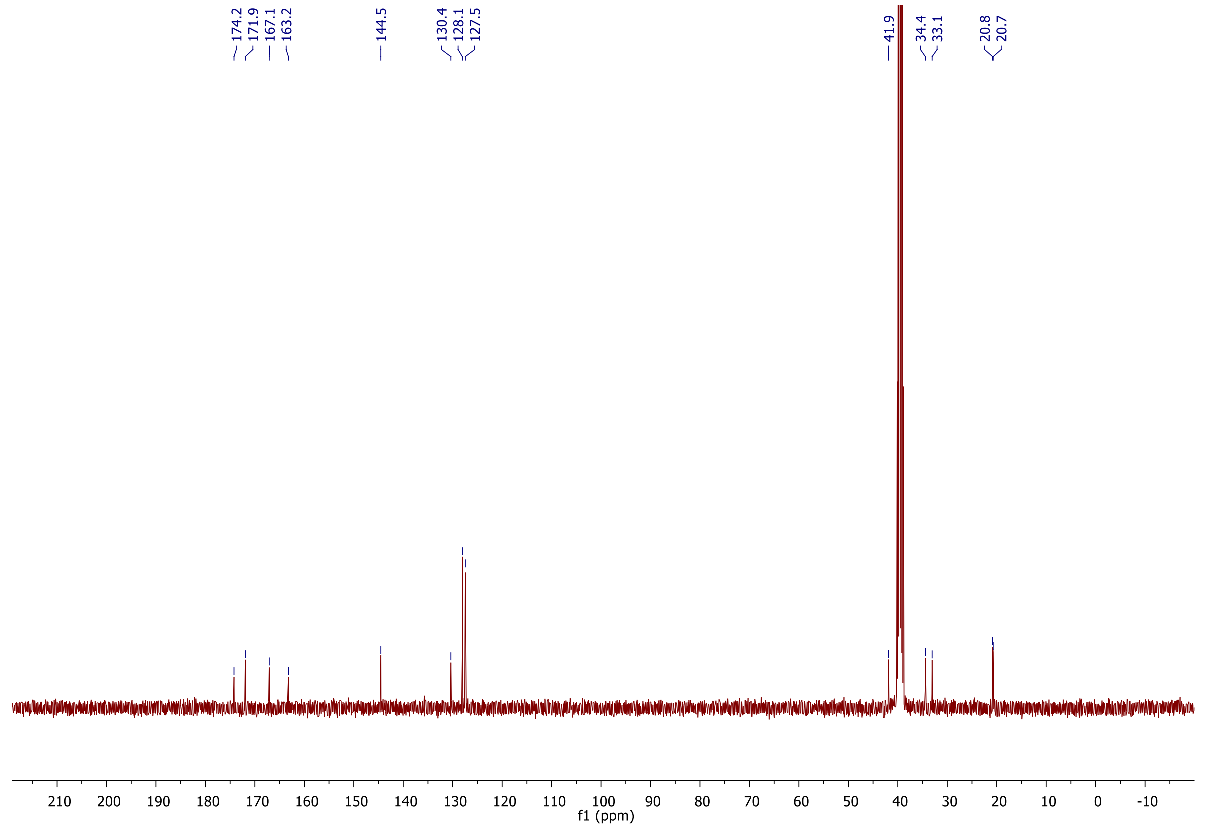
174.2
171.9
167.1
163.2
144.5
130.4
128.1
127.5
41.9
34.4
33.1
20.8
20.7
f1 (ppm)

Di-*tert*-butyl 1-(prop-2-yn-1-yl)hydrazine-1,2-dicarboxylate S10[5]

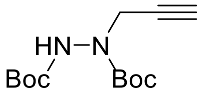

S10

To a solution of di-*tert*-butyl hydrazine-1,2-dicarboxylate (3.00 g, 12.9 mmol) in a mixture of toluene (15 mL) and 5% aq. NaOH (15mL) were added tetra-*n*-butylammonium bromide (104 mg, 0.32 mmol) and propargyl bromide (5.76 g, 38.7 mmol). The reaction mixture was stirred at 21 °C for 16 h. After this time, water (20 mL) was added and the mixture was extracted with EtOAc (3 × 30 mL). The combined organic layers were washed with brine (30 mL), dried ($MgSO_4$), and concentrated *in vacuo*. Purification by flash column chromatography (20 % EtOAc/petrol) afforded di-*tert*-butyl 1-(prop-2-yn-1-yl)hydrazine-1,2-dicarboxylate **S10** (2.04 g, 7.56 mmol, 59%) as a white solid. ^{1}H NMR (400 MHz, $CDCl_3$, rotamers) δ 6.48-6.17 (br s, 1H), 4.27 (s, 2H), 2.24 (t, *J* = 2.4 Hz, 1H), 1.47 (s, 18H); ^{13}C NMR (125 MHz, $CDCl_3$, rotamers) δ 154.9 (C), 154.7 (C), 82.1 (C), 81.6 (C), 78.8 (C), 72.1 (CH), 39.3 (CH_2), 28.3 (CH_3), 28.2 (CH_3); IR (solid) 3310, 3290, 2982, 1729, 1688, 1512 cm^{-1}.

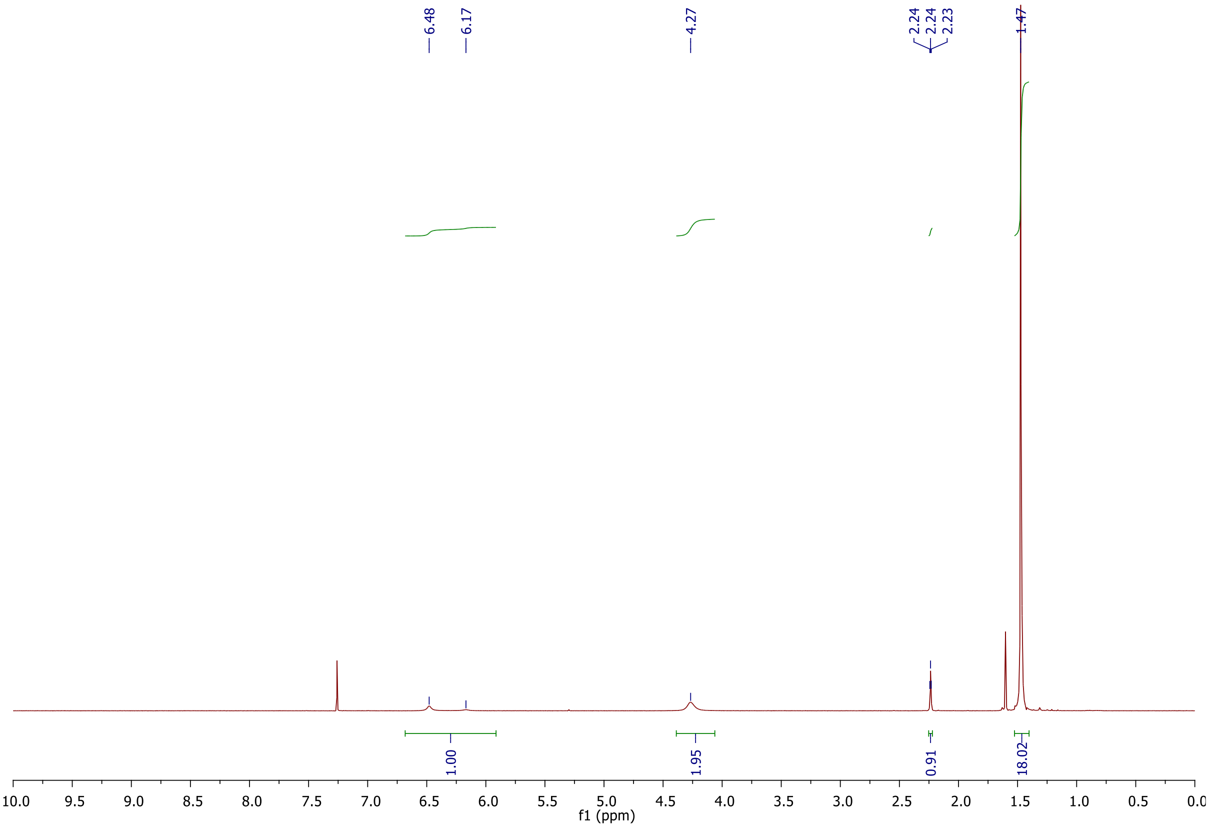

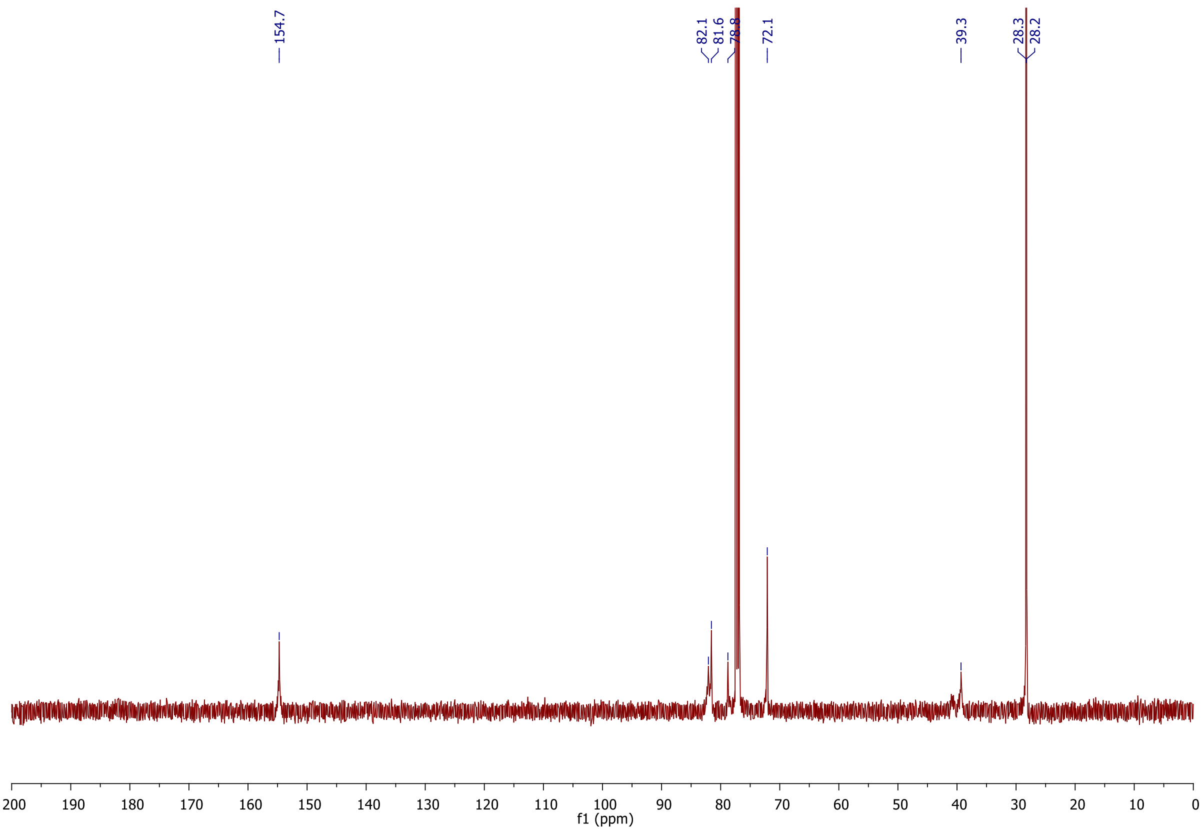
154.7
82.1
81.6
78.8
72.1
39.3
28.3
28.2
200 190 180 170 160 150 140 130 120 110 100 90 80 70 60 50 40 30 20 10 0
f1 (ppm)

Di-*tert*-butyl 1-(3-(tert-butoxy)-3-oxopropyl)-2-(prop-2-yn-1-yl)hydrazine-1,2-dicarboxylate S11

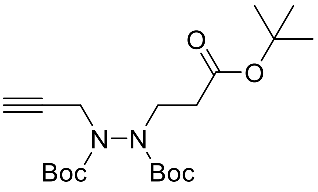

S11

To a solution of di-*tert*-butyl 1-(prop-2-yn-1-yl)hydrazine-1,2-dicarboxylate **S10** (2.05 g, 7.60 mmol) in *t*-BuOH (15 mL) was added 10% NaOH (0.17 mL), and the reaction mixture stirred at 21 °C for 10 min. After this, *tert*-butyl acrylate (1.47 mL, 15.2 mmol) was added to the solution and the reaction mixture was heated at 60 °C for 24 h. Following this, the solvent was removed *in vacuo* and the crude residue was dissolved in EtOAc (150 mL) and washed with water (3 × 50 mL). The organic layer was then dried ($MgSO_4$) and concentrated *in vacuo.* Purification of the crude residue by flash column chromatography (0% to 20% EtOAc/petrol) afforded di-*tert*-butyl 1-(3-(tert-butoxy)-3-oxopropyl)-2-(prop-2-yn-1-yl)hydrazine-1,2-dicarboxylate **S11** (2.20 g, 5.52 mmol, 73%) as a clear oil: ^{1}H NMR (600 MHz, $CDCl_3$, rotamers) δ 4.61–4.00 (m, 2H), 3.83–3.68 (m, 2H), 2.64 (t, *J* = 7.6 Hz, 2H), 2.27 (t, *J* = 2.5 Hz, 1H), 1.50–1.43 (m, 27H); ^{13}C NMR (150 MHz, $CDCl_3$, rotamers) δ 171.1 (C), 155.5 (C), 154.4 (C), 82.0 (C), 81.5 (C), 80.7 (C), 78.5 (C), 73.0 (CH), 46.2 (CH_2), 39.5 (CH_2), 34.2 (CH_2), 28.3 (CH_3), 28.2 (CH_3), 28.2 (CH_3); IR (thin film) 3265, 2978, 2934, 1710 cm^{-1}; LRMS (ESI) 343 (60, $[M-C_4H_9+2H]^+$, 399 (100, $[M+H]^+$). HRMS (ESI) calcd for $C_{20}H_{34}N_2O_6$ $[M+H]^+$ 399.2490; observed 399.2487.

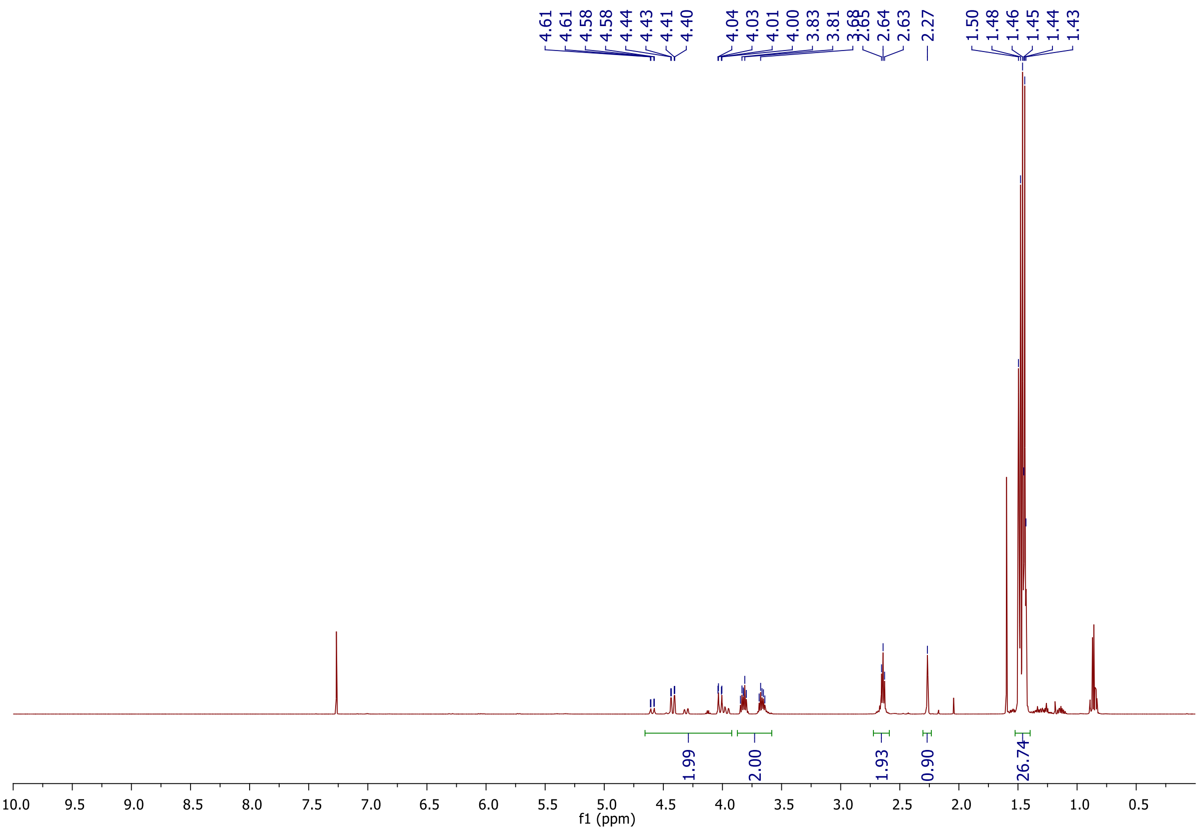
4.61 4.61 4.58 4.58 4.44 4.43 4.41 4.40 4.04 4.03 4.01 4.00 3.83 3.81 3.68 3.65 2.64 2.63 2.27 1.50 1.48 1.46 1.45 1.44 1.43
1.99 2.00 1.93 0.90 26.74
10.0 9.5 9.0 8.5 8.0 7.5 7.0 6.5 6.0 5.5 5.0 4.5 4.0 3.5 3.0 2.5 2.0 1.5 1.0 0.5
f1 (ppm)

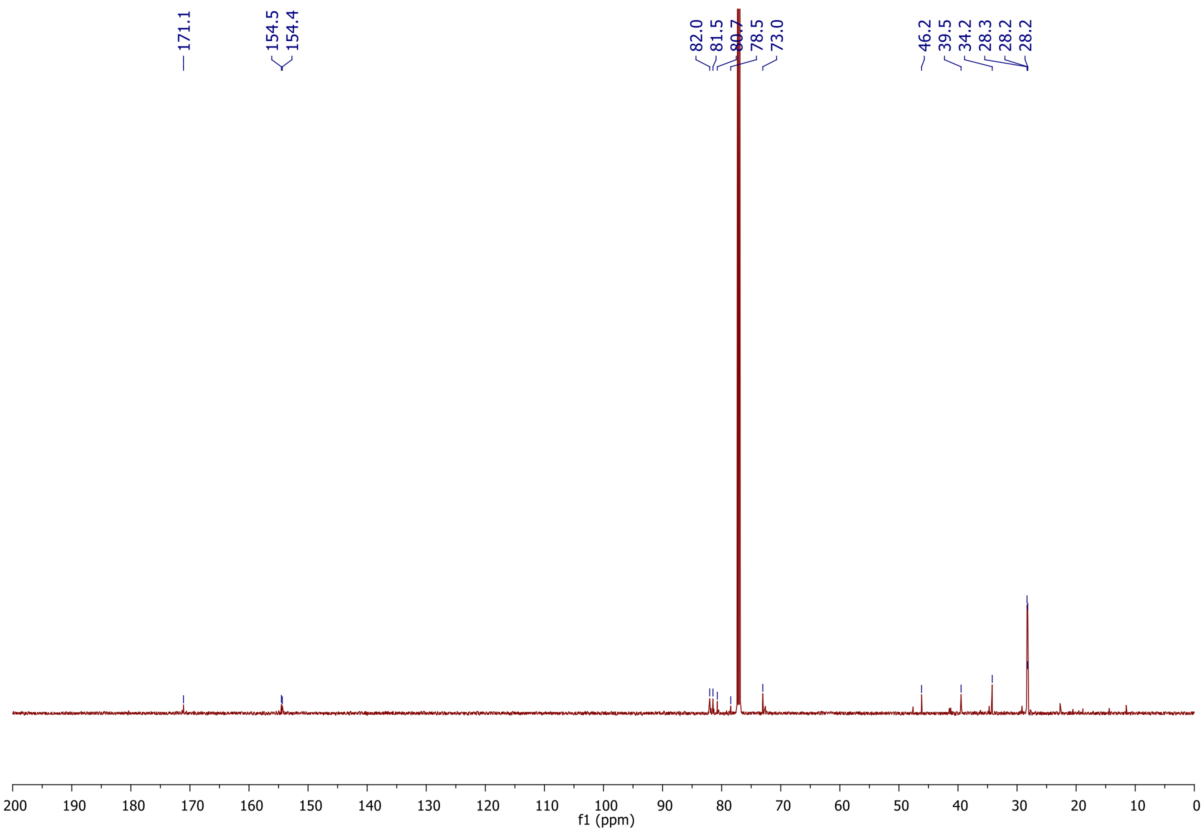
171.1 154.5 154.4 82.0 81.5 80.7 78.5 73.0 46.2 39.5 34.2 28.3 28.2 28.2
200 190 180 170 160 150 140 130 120 110 100 90 80 70 60 50 40 30 20 10 0
f1 (ppm)

3-(4,5-Dibromo-3,6-dioxo-2-(prop-2-yn-1-yl)-2,3-dihydropyridazin-1(6*H*)-yl)propanoic acid S12

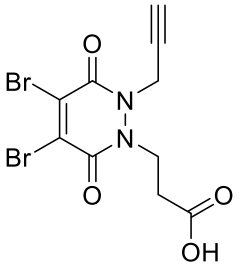

S12

Dibromomaleic acid (1.66 g, 6.06 mmol) was dissolved in AcOH (25 mL) and heated under reflux for 30 min. To this solution, was added di-*tert*-butyl 1-(3-(*tert*-butoxy)-3-oxopropyl)-2-(prop-2-yn-1-yl)hydrazine-1,2-dicarboxylate **S11** (2.20 g, 5.51 mmol) and the reaction heated under reflux for a further 4 h. After this time, the reaction mixture was concentrated *in vacuo* with toluene co-evaporation (3 × 30 mL, as an azeotrope) and the crude residue purified by flash column chromatography (50% to 100% EtOAc/petrol (1% AcOH)) to afford 3-(4,5-dibromo-3,6-dioxo-2-(prop-2-yn-1-yl)-2,3-dihydropyridazin-1(6*H*)-yl)propanoic acid **S12** (801 mg, 2.25 mmol, 77%) as a yellow solid: m.p. 136–140 °C; ^{1}H NMR (600 MHz, DMSO-D$_6$) δ 4.91 (d, *J* = 2.4 Hz, 2H), 4.27 (t, *J* = 7.5 Hz, 2H), 3.77 (s, 1H), 3.52 (t, *J* = 2.4 Hz, 1H), 2.66 (t, *J* = 7.5 Hz, 2H); ^{13}C NMR (150 MHz, DMSO-d$_6$) δ 171.9 (C), 153.4 (C), 153.0 (C), 136.2 (C), 135.1 (C), 77.2 (C), 76.7 (CH), 43.5 (CH$_2$), 37.6 (CH$_2$), 31.5 (CH$_2$); IR (solid) 3216, 2979, 1723 cm^{-1} LRMS (ESI). 379 (5k0, [M^{81}Br^{81}Br+H]$^+$), 381 (100, [M^{79}Br^{81}Br+H]$^+$), 383 (50, [M^{79}Br^{79}Br+H]$^+$). HRMS (ESI) calcd for $C_{10}H_8Br_2N_2O_4$ [M^{79}Br^{81}Br+H]$^+$ 380.8904; observed 380.8906.

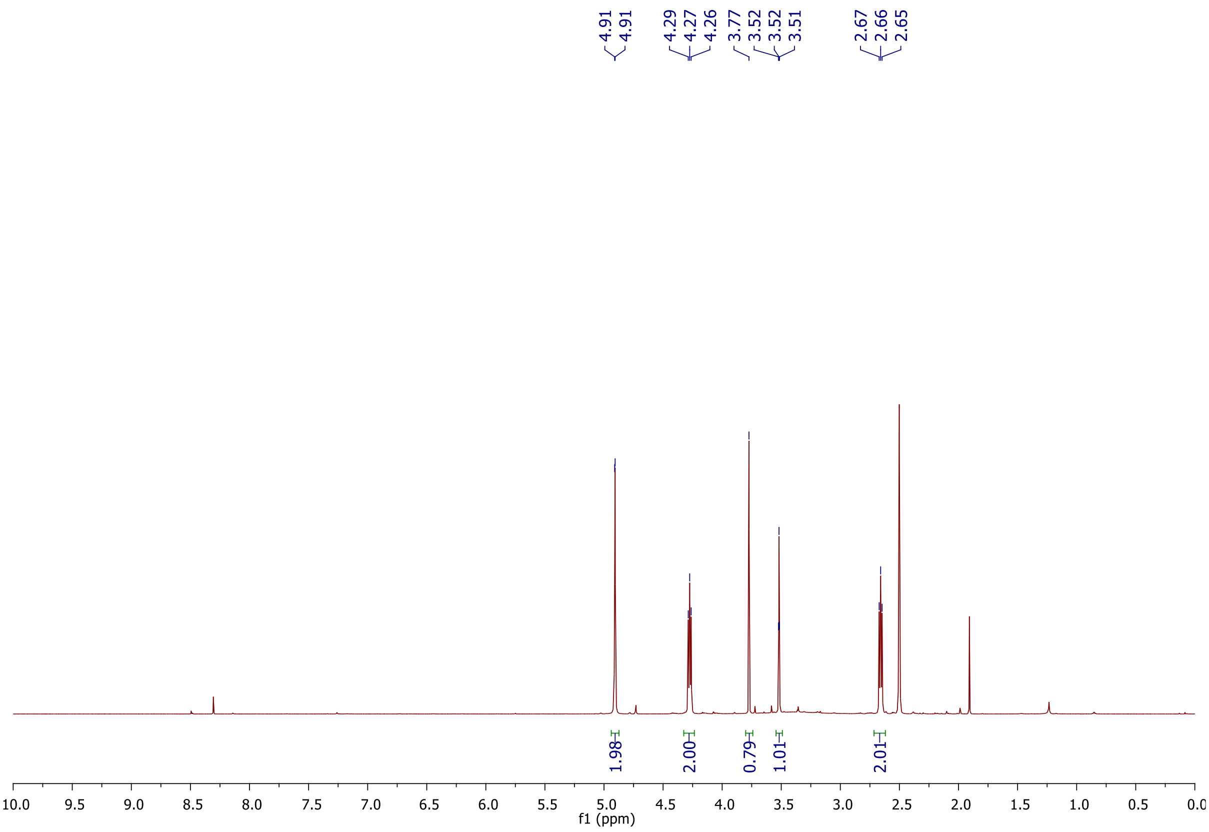

4.91
4.91
4.29
4.27
4.26
3.77
3.52
3.52
3.51
2.67
2.66
2.65
1.98
2.00
0.79
1.01
2.01
f1 (ppm)

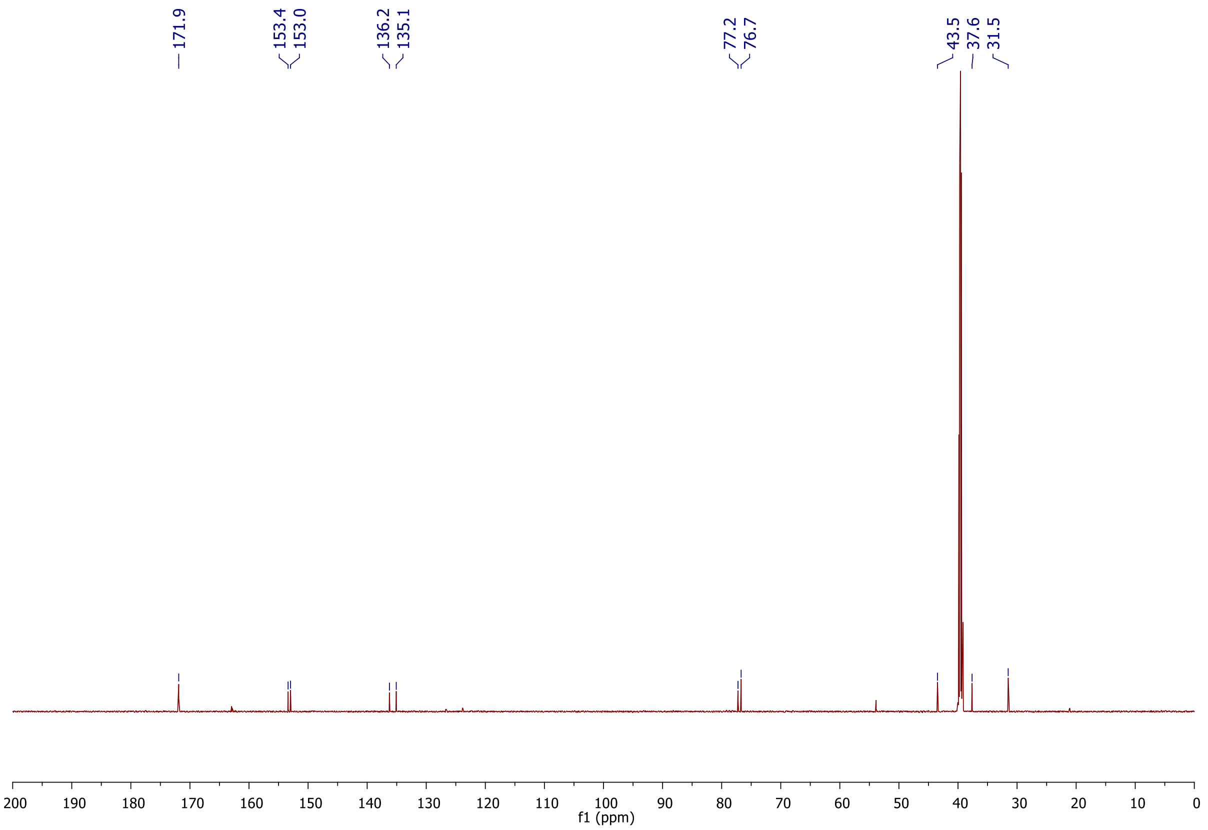

171.9
153.4
153.0
136.2
135.1
77.2
76.7
43.5
37.6
31.5
f1 (ppm)

N^1-(2-(2-(2-(2-Azidoethoxy)ethoxy)ethoxy)ethyl)-N^5-(4-(6-methyl-1,2,4,5-tetrazin-3-yl)benzyl)glutaramide S13

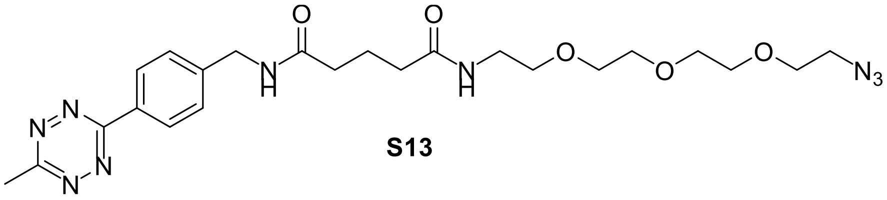

S13

To a solution of 5-((4-(6-methyl-1,2,4,5-tetrazin-3-yl)benzyl)amino)-5-oxopentanoic acid **S9** (200 mg, 0.63 mmol) in CH_2Cl_2 (5 mL) was added HATU (240 mg, 0.63 mmol), and NEt_3 (87.8 µL, 0.63 mmol) and the reaction stirred for 5 min at 21 °C. To this solution was added a solution of 2-(2-(2-(2-azidoethoxy)ethoxy)ethoxy)ethan-1-amine (387.5 µL, 1.96 mmol) in CH_2Cl_2 (5 mL), and the resulting solution was stirred at 21 °C for 16 h. The reaction was diluted with EtOAc (25 mL) and water (25 mL) and the phases separated. The aqueous phase was extracted with EtOAc (3 × 25 mL) and the combined organic phases were washed with water (3 × 25 mL), brine (20 mL), dried ($MgSO_4$) and the solvent removed *in vacuo*. The crude residue was purified by flash column chromatography (0-10% MeOH in CH_2Cl_2) to afford N^1-(2-(2-(2-(2-azidoethoxy)ethoxy)ethoxy)ethyl)-N^5-(4-(6-methyl-1,2,4,5-tetrazin-3-yl)benzyl)glutaramide **S13** (209.5 mg, 0.51 mmol, 64%) as a purple solid: ^{1}H NMR (400 MHz, $CDCl_3$) δ 8.55 (d, *J* = 8.4 Hz, 2H), 7.51 (d, *J* = 8.5 Hz, 2H), 6.61 (br s, 1H), 6.16 (br s, 1H), 4.55 (d, *J* = 6.0 Hz, 2H), 3.67–3.60 (m, 10 H), 3.55–3.53 (m, 2H), 3.44–3.36 (m, 4H), 3.09 (s, 3H), 2.35 (t, *J* = 7.1 Hz, 2H), 2.26 (t, *J* = 6.9 Hz, 2H), 2.01 (p, *J* = 6.9 Hz, 2H); ^{13}C NMR (100 MHz, $CDCl_3$) δ 172.7 (C), 172.7 (C), 167.3 (C), 163.9 (C), 143.6 (C), 131.0 (C), 128.5 (CH), 128.3 (CH), 70.8 (CH_2), 70.7 (CH_2), 70.6 (CH_2), 70.3 (CH_2), 70.1(CH_2), 69.8 (CH_2), 50.7 (CH_2), 43.2 (CH_2), 39.3 (CH_2), 35.3 (CH_2), 35.2 (CH_2), 22.0 (CH_2), 21.2 (CH_3). IR (solid) 3298, 3076, 2868, 2101, 1637, 1541 cm^{-1}. HRMS (ESI) calcd for $C_{23}H_{34}N_9O_5$ [M+H]$^+$ 516.2677; observed 516.2677.

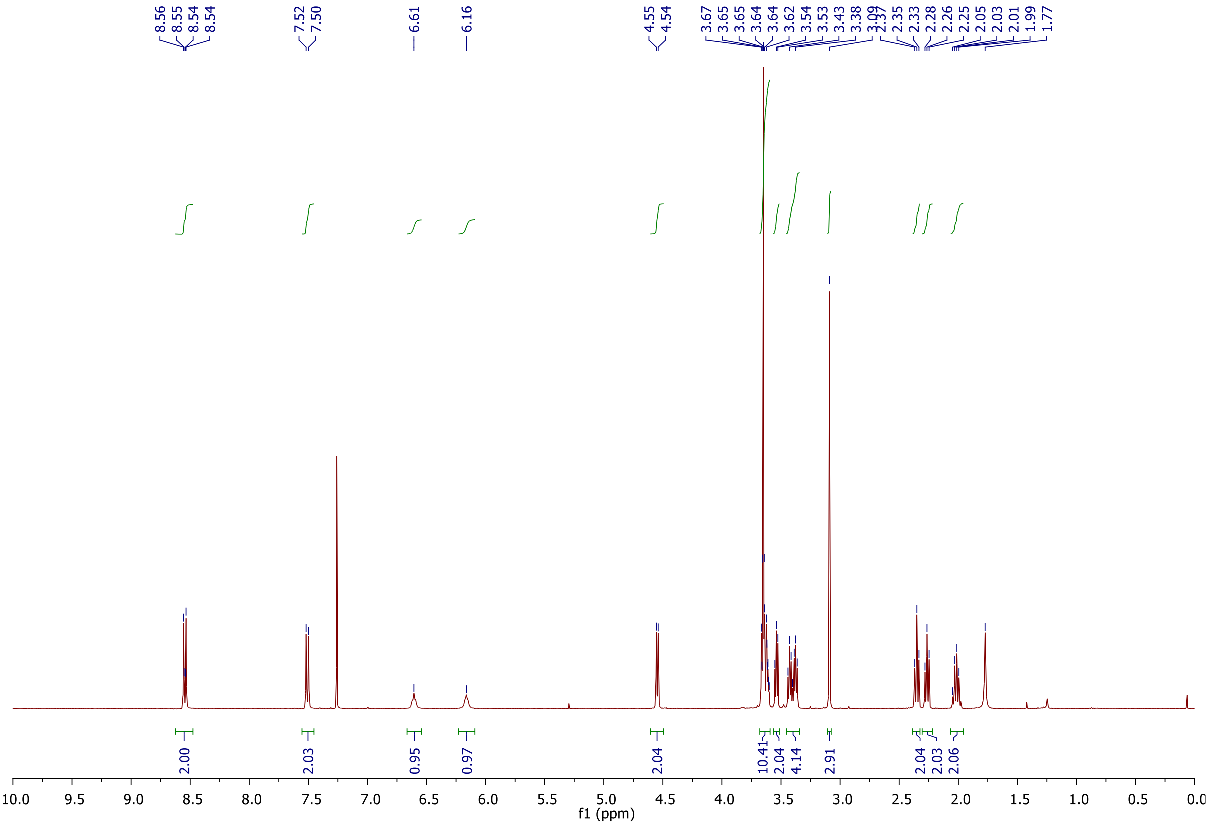
8.56
8.55
8.54
8.54
7.52
7.50
6.61
6.16
4.55
4.54
3.67
3.65
3.65
3.64
3.64
3.62
3.54
3.53
3.43
3.38
3.09
2.37
2.35
2.33
2.28
2.26
2.25
2.05
2.03
2.01
1.99
1.77
2.00
2.03
0.95
0.97
2.04
10.41
2.04
4.14
2.91
2.04
2.03
2.06
f1 (ppm)

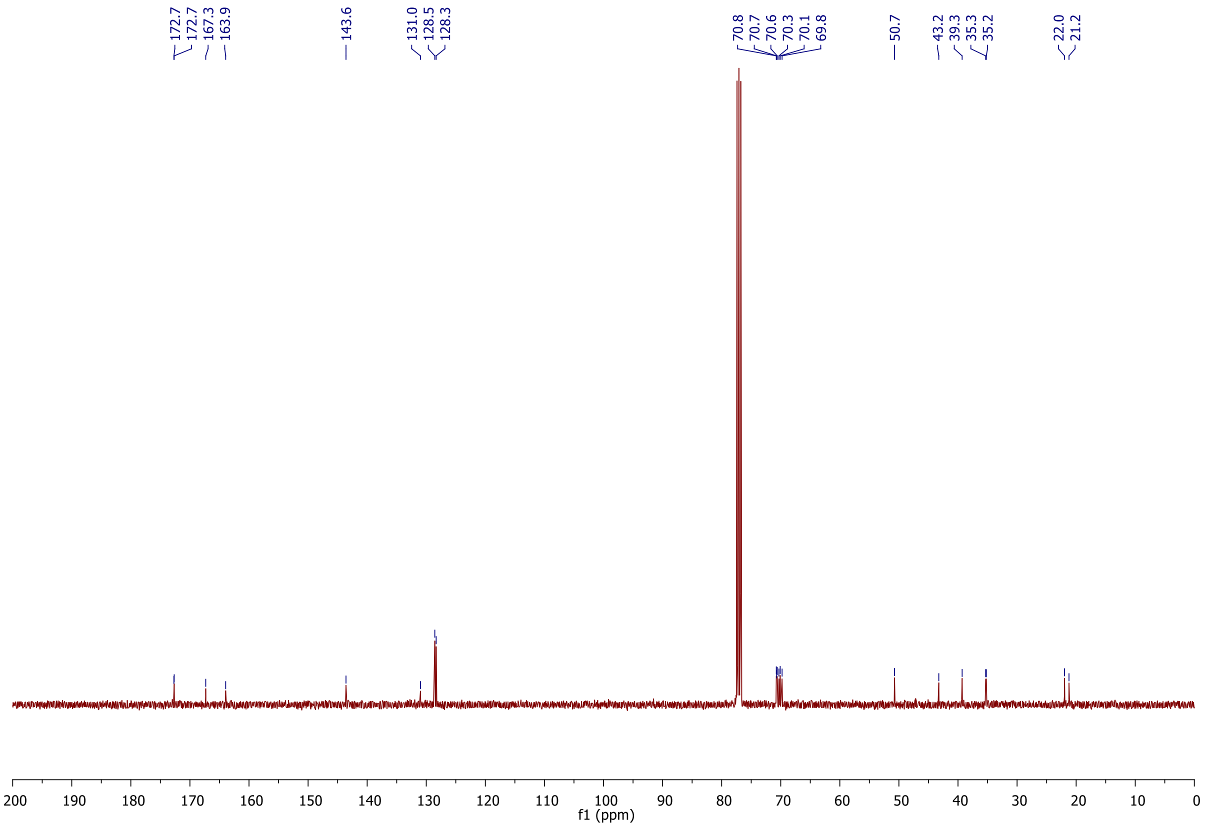
172.7
172.7
167.3
163.9
143.6
131.0
128.5
128.3
70.8
70.7
70.6
70.3
70.1
69.8
50.7
43.2
39.3
35.3
35.2
22.0
21.2
f1 (ppm)

3-(4,5-Dibromo-2-((1-(1-(4-(6-methyl-1,2,4,5-tetrazin-3-yl)phenyl)-3,7-dioxo-11,14,17-trioxa-2,8-diazanonadecan-19-yl)-1*H*-1,2,3-triazol-4-yl)methyl)-3,6-dioxo-3,6-dihydropyridazin-1(2*H*)-yl)propanoic acid S14

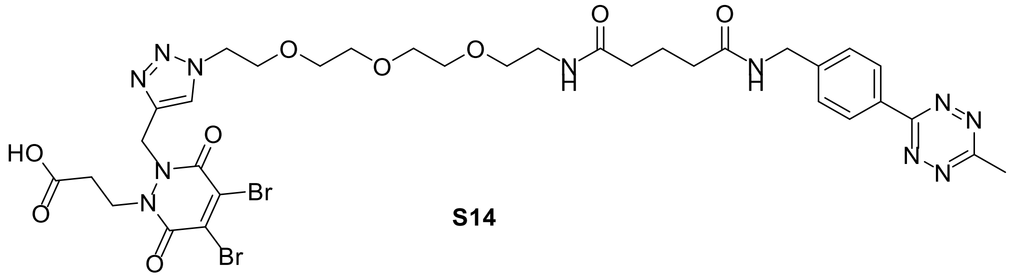

S14

To a solution of *N*[1]-(2-(2-(2-(2-azidoethoxy)ethoxy)ethoxy)ethyl)-*N*[5]-(4-(6-methyl-1,2,4,5-tetrazin-3-yl)benzyl)glutaramide **S13** (50 mg, 97.0 µmol) and 3-(4,5-dibromo-3,6-dioxo-2-(prop-2-yn-1-yl)-3,6-dihydropyridazin-1(2*H*)-yl)propanoic acid **S12** (44.3 mg, 116.6 µmol) in THF (10 mL) was added DIPEA (16.8 µL, 97.0 µmol) and CuI (9.35 mg, 48.5 µmol) and the mixture stirred at 21 °C for 5 h. The mixture was then filtered, and the solvent removed *in vacuo*. The mixture was dissolved in water (10 mL), basified with sat. aq. $NaHCO_3$, and washed with CH_2Cl_2 (3 × 10 mL). CH_2Cl_2 (10 mL) was then added to the aqueous phase. The aqueous phase was then acidified with 4 M HCl until the purple product visibly transferred to the organic phase. The aqueous phase was then extracted with further CH_2Cl_2 (3 × 10 mL). The combined organic phases were washed with brine, dried ($MgSO_4$), filtered and the solvent removed *in vacuo*. The crude residue was purified by column chromatography (0% to 20% MeOH/CH_2Cl_2, 1% AcOH) to yield 3-(4,5-dibromo-2-((1-(1-(4-(6-methyl-1,2,4,5-tetrazin-3-yl)phenyl)-3,7-dioxo-11,14,17-trioxa-2,8-diazanonadecan-19-yl)-1*H*-1,2,3-triazol-4-yl)methyl)-3,6-dioxo-3,6-dihydropyridazin-1(2*H*)-yl)propanoic acid **S14** (67.2 mg, 75.0 µmol, 77%) as a purple solid: ^{1}H NMR (400 MHz, DMSO-d$_6$) δ 8.42 (d, *J* = 8.3 Hz, 2H), 8.10 (s, 1H), 7.88 (s, 1H), 7.51 (d, *J* = 8.3 Hz, 2H), 5.35 (br s, 2H), 4.5 (t, *J* = 5.1 Hz, 2H), 4.38 (d, *J* = 6.0 Hz, 2H), 4.30 (s 2H), 3.78 (t, *J* = 5.3 Hz, 2H), 3.48–3.44 (m, 9 H), 3.39 (t, *J* = 6.0 Hz, 3H), 3.18 (q, *J* = 5.9 Hz, 2H), 2.99 (s, 3H), 2.18 (t, *J* = 7.5 Hz, 2H), 2.10 (t, *J* = 7.4 Hz, 2H), 1.76 (p, *J* = 7.7 Hz, 2 H); ^{13}C NMR (125 MHz, DMSO-d$_6$) δ 172.0 (C), 171.8 (C), 167.1 (C), 163.2 (C), 153.5 (C), 152.9 (C), 144.6 (C), 135.9 (C), 135.2 (C), 130.3 (C), 128.1 (CH), 127.5 (CH), 124.5 (CH), 69.7 (CH_2), 69.6 (CH_2), 69.6 (CH_2), 69.2 (CH_2), 68.6 (CH_2), 54.9 (CH_2), 49.6 (CH_2), 41.8 (CH_2), 34.8 (CH_2), 34.7 (CH_2), 21.5 (CH_2), 20.8 (CH_3). IR (thin film) 3335, 2924,

1721, 1630, 1545 cm^{-1}. HRMS (ESI) calcd for $C_{33}H_{42}Br_2N_{11}O_9$ $[M^{79}Br^{81}Br+H]^+$ 896.1435; observed 896.1503.

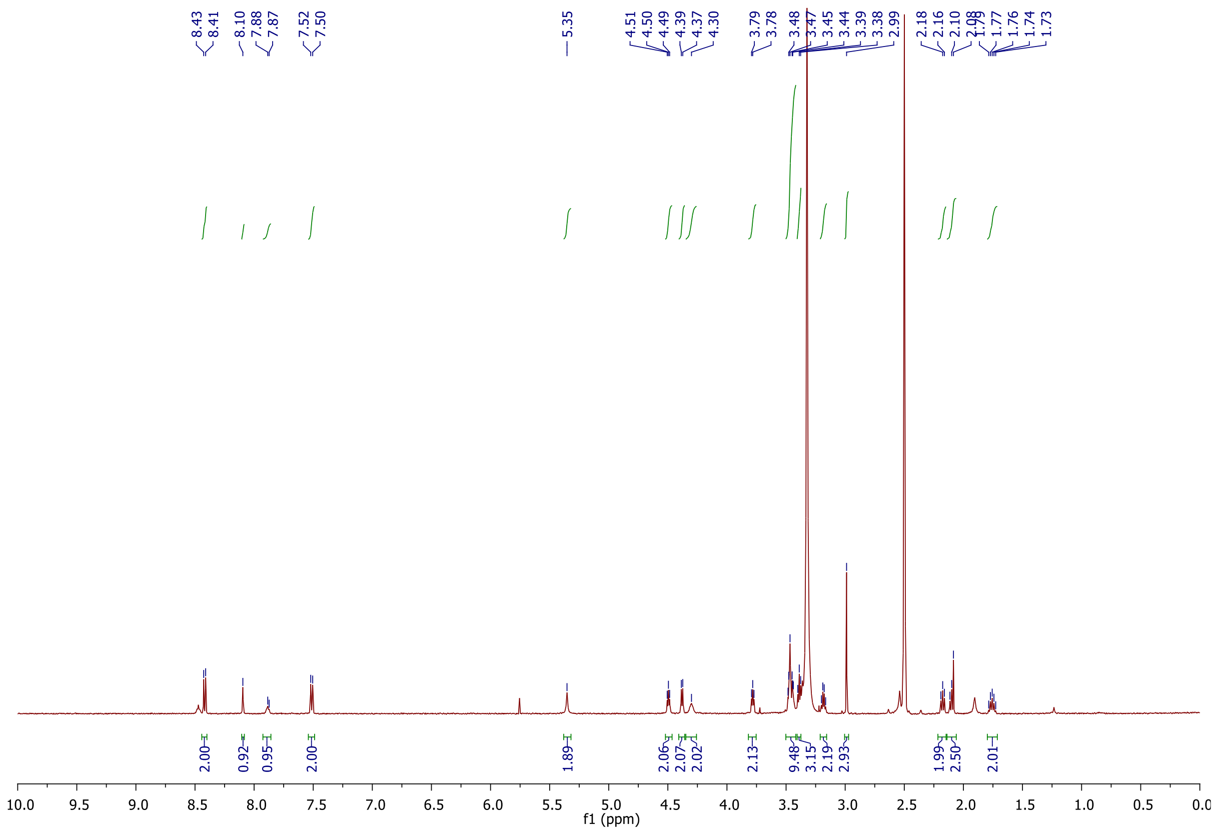

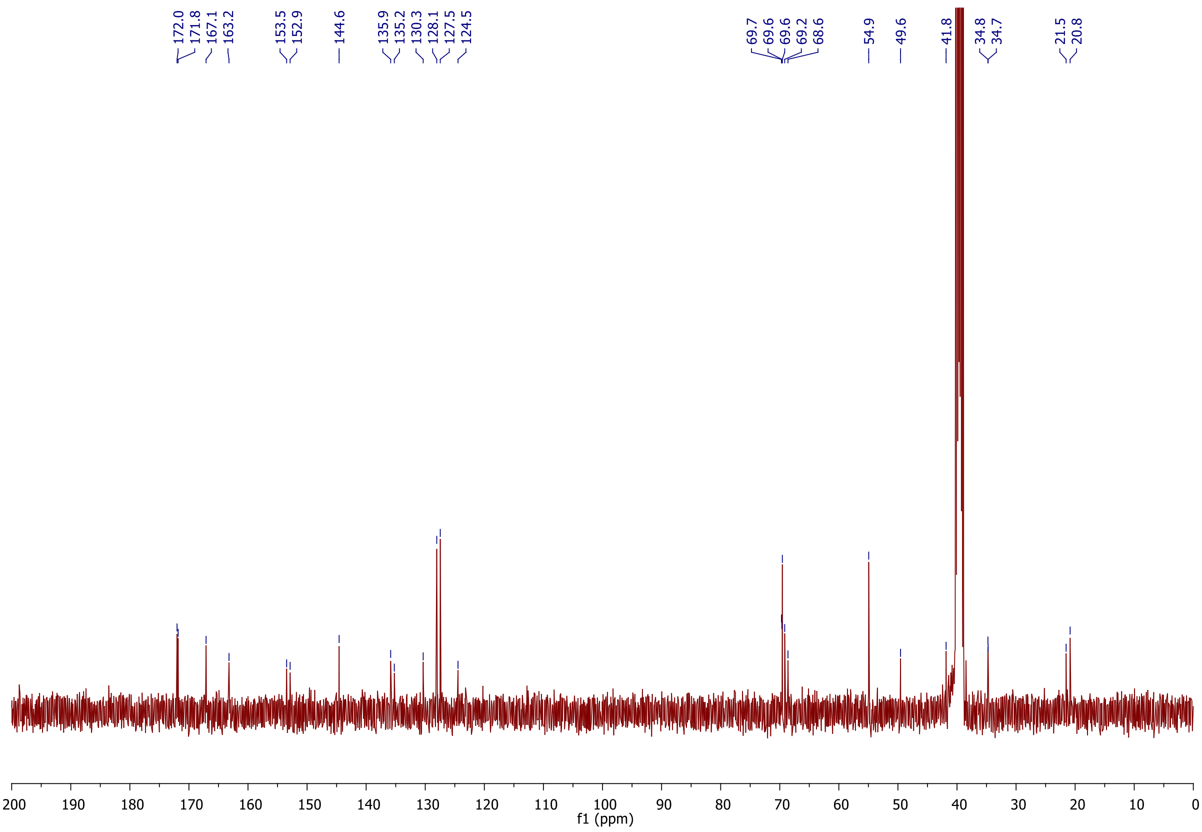
172.0
171.8
167.1
163.2
153.5
152.9
144.6
135.9
135.2
130.3
128.1
127.5
124.5
69.7
69.6
69.6
69.2
68.6
54.9
49.6
41.8
34.8
34.7
21.5
20.8
200 190 180 170 160 150 140 130 120 110 100 90 80 70 60 50 40 30 20 10 0
f1 (ppm)

N^1-(2-(2-(2-(2-(4-((2-(1-Azido-13-oxo-3,6,9-trioxa-12-azapentadecan-15-yl)-4,5-dibromo-3,6-dioxo-3,6-dihydropyridazin-1(2*H*)-yl)methyl)-1*H*-1,2,3-triazol-1-yl)ethoxy)ethoxy)ethoxy)ethyl)-N^5-(4-(6-methyl-1,2,4,5-tetrazin-3-yl)benzyl)glutaramide 22

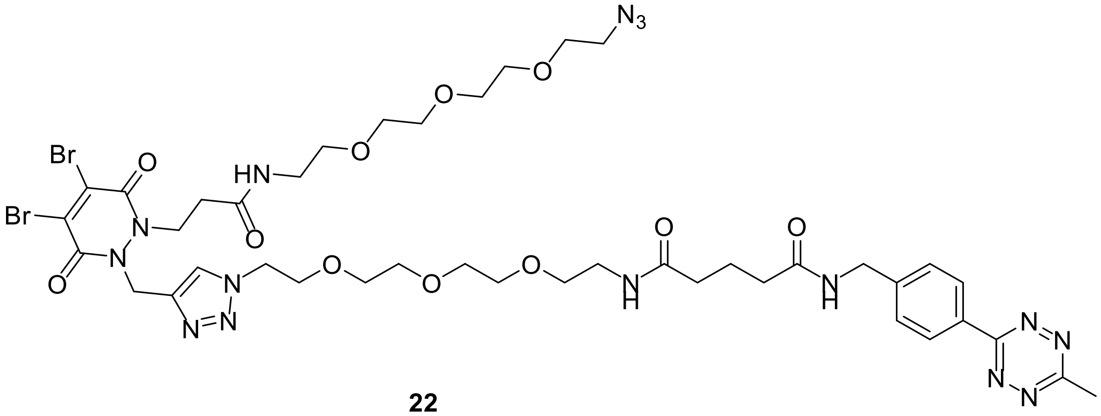

22

=

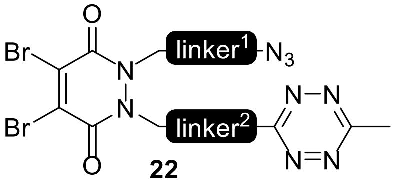

22

To a solution of 3-(4,5-dibromo-2-((1-(1-(4-(6-methyl-1,2,4,5-tetrazin-3-yl)phenyl)-3,7-dioxo-11,14,17-trioxa-2,8-diazanonadecan-19-yl)-1*H*-1,2,3-triazol-4-yl)methyl)-3,6-dioxo-3,6-dihydropyridazin-1(2*H*)-yl)propanoic acid **S14** (40 mg, 45 µmol) in CH_2Cl_2 (2.5 mL) was added HATU (28.1 mg, 74 µmol), and DIPEA (5.78 mg, 45 µmol) and the reaction stirred for 5 min at 21 °C. To this solution was added 2-(2-(2-(2-azidoethoxy)ethoxy)ethoxy)ethan-1-amine (14.6 mg, 67 µmol) in CH_2Cl_2 (2.5 mL), and the resulting mixture was stirred at 21 °C for a further 16 h. The reaction was diluted with EtOAc (10 mL) and washed with sat. aq. $NaHCO_3$ (3 × 10 mL), 1 M HCl (3 × 10 mL), water (10 mL), brine (10 mL), dried ($MgSO_4$), filtered and the solvent removed *in vacuo*. The crude residue was purified by flash column chromatography (0% to 10% MeOH/CH_2Cl_2) to afford N^1-(2-(2-(2-(2-(4-((2-(1-azido-13-oxo-3,6,9-trioxa-12-azapentadecan-15-yl)-4,5-dibromo-3,6-dioxo-3,6-dihydropyridazin-1(2*H*)-yl)methyl)-1*H*-1,2,3-triazol-1-yl)ethoxy)ethoxy)ethoxy)ethyl)-N^5-(4-(6-methyl-1,2,4,5-tetrazin-3-yl)benzyl)glutaramide **22** (16.7 mg, 15 µmol, 34%) as a purple solid: ^{1}H NMR (500 MHz, $CDCl_3$) δ 8.52 (d, *J* = 8.4 Hz, 2H), 7.84 (s, 1H), 7.50 (d, *J* = 8.5 Hz, 2H), 6.74 (br s, 1H), 6.65 (br s, 1H), 6.43 (br s, 1H), 4.65 (t, *J* = 6.8 Hz, 2H), 4.54 (d, *J* = 5.9 Hz, 2H), 4.50 (t, *J* =

4.9 Hz, 2H), 3.85 (t, J = 5.1 Hz, 2H), 3.68–3.51 (m, 24 H), 3.43–3.38 (m, 6H), 3.09 (s, 3H), 2.66 (t, J = 6.8 Hz, 2H), 2.35 (t, J = 7.1 Hz, 2H), 2.38 (t, J = 7.0 Hz, 2H), 2.01 (p, J = 7.0 Hz, 2 H); ^{13}C NMR (125 MHz, $CDCl_3$) δ 172.9 (C), 172.8 (C), 169.5 (C), 167.4 (C), 164.0 (C), 153.4 (C), 153.1 (C), 143.7 (C), 141.0 (C), 136.5 (C), 135.7 (C), 131.0 (C), 128.6 (CH), 128.3 (CH), 125.1 (CH), 70.8 (CH_2), 70.7 (CH_2), 70.7 (CH_2), 70.6 (CH_2), 70.6 (CH_2), 70.6 (CH_2), 70.4 (CH_2), 70.3 (CH_2), 70.1(CH_2), 69.9 (CH_2), 69.6 (CH_2), 69.3 (CH_2), 50.8 (CH_2), 50.5 (CH_2), 45.2 (CH_2), 43.3 (CH_2), 42.7 (CH_2), 39.5 (CH_2), 39.3 (CH_2), 35.5 (CH_2), 35.3 (CH_2), 34.0 (CH_2), 29.8 (CH_2), 22.0 (CH_2), 21.3 (CH_3). IR (thin film) 3306, 2919, 2101, 1722, 1634, 1543 cm^{-1}. HRMS (ESI) calcd for $C_{41}H_{58}Br_2N_{15}O_{11}$ [$M^{79}Br^{81}Br+H$]$^+$ 1096.2708; observed 1096.2782.

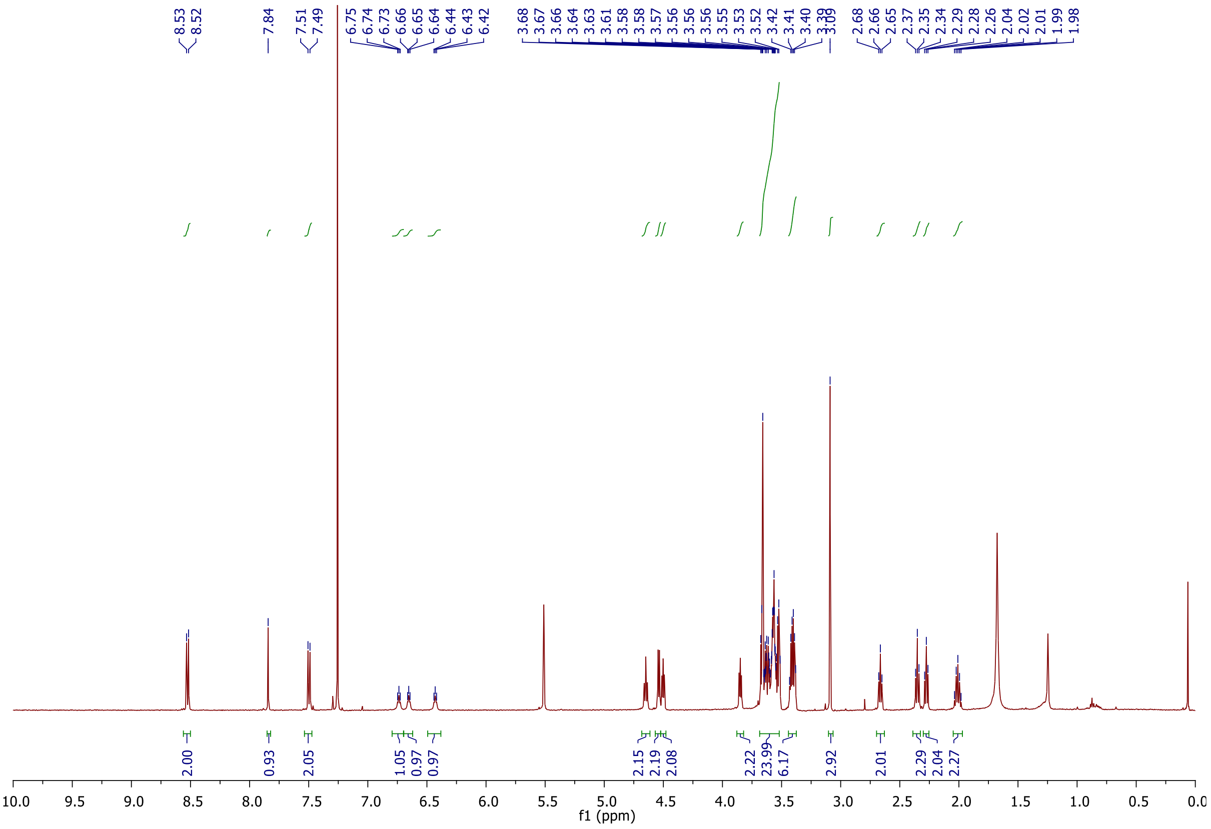

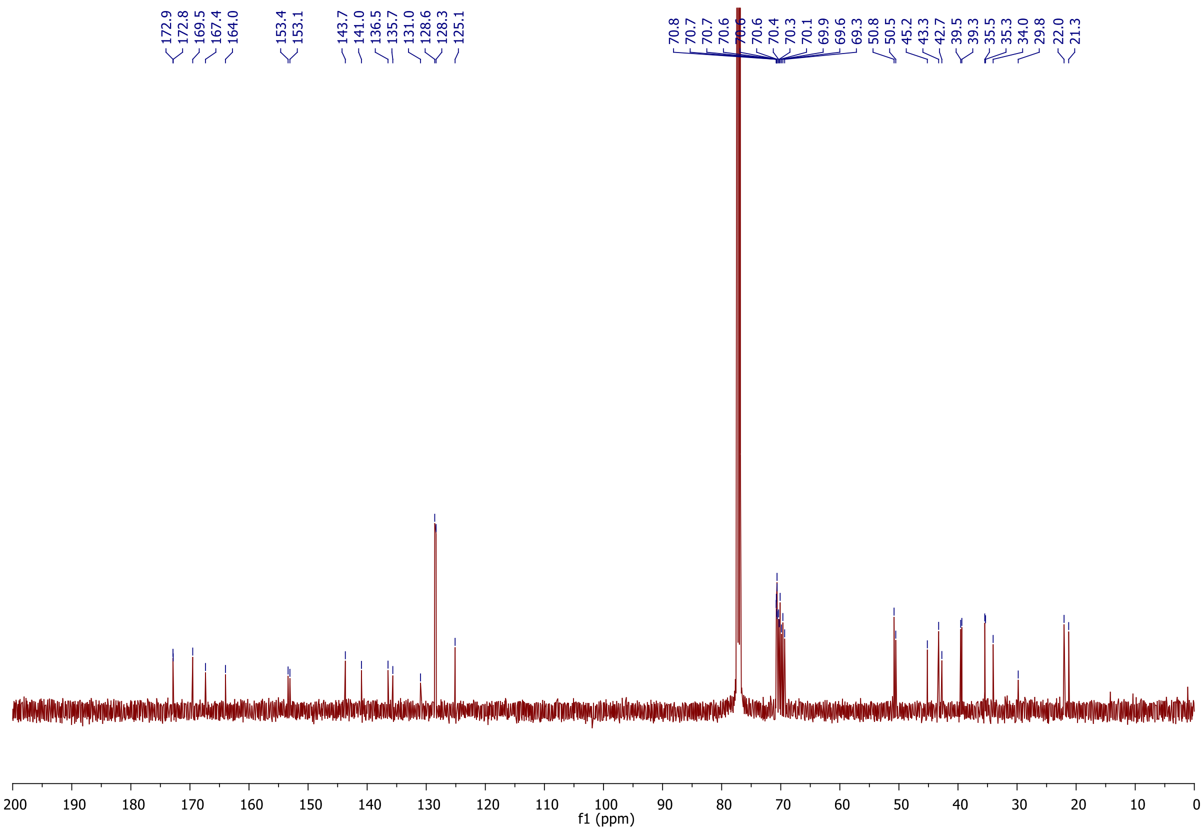
172.9
172.8
169.5
167.4
164.0
153.4
153.1
143.7
141.0
136.5
135.7
131.0
128.6
128.3
125.1
70.8
70.7
70.7
70.6
70.6
70.6
70.4
70.3
70.1
69.9
69.6
69.3
50.8
50.5
45.2
43.3
42.7
39.5
39.3
35.5
35.3
34.0
29.8
22.0
21.3
f1 (ppm)

2-(4-Hydroxyphenyl)isoindoline-1,3-dione S15[6]

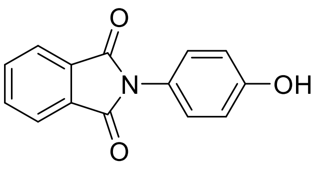

S15

p-Aminophenol (275 mg, 2.52 mmol) was added to phthalic anhydride (0.373 g, 2.52 mmol) dissolved AcOH (10 mL). The mixture was heated under reflux for 16 h. After cooling to room temperature, the precipitate was filtered and washed with MeOH (10 mL). The solvent was removed *in vacuo* to afford 2-(4-hydroxyphenyl)isoindoline-1,3-dione **S15** as a white solid (0.391 g, 1.64 mmol, 65%). ^{1}H NMR (600 MHz, DMSO-D$_6$); δ 9.75 (s, 1H), 7.95–7.93 (m, 2H), 7.90–7.88 (m, 2H), 7.21–7.20 (m, 2H), 6.88–6.86 (m, 2H); ^{13}C NMR (150 MHz, DMSO-D$_6$); δ 167.4 (C), 157.3 (C), 134.6 (CH), 131.6 (C), 128.8 (CH), 123.3 (CH), 122.9 (C), 115.4 (CH). IR (solid) 3412, 1787, 1712, cm^{-1}.

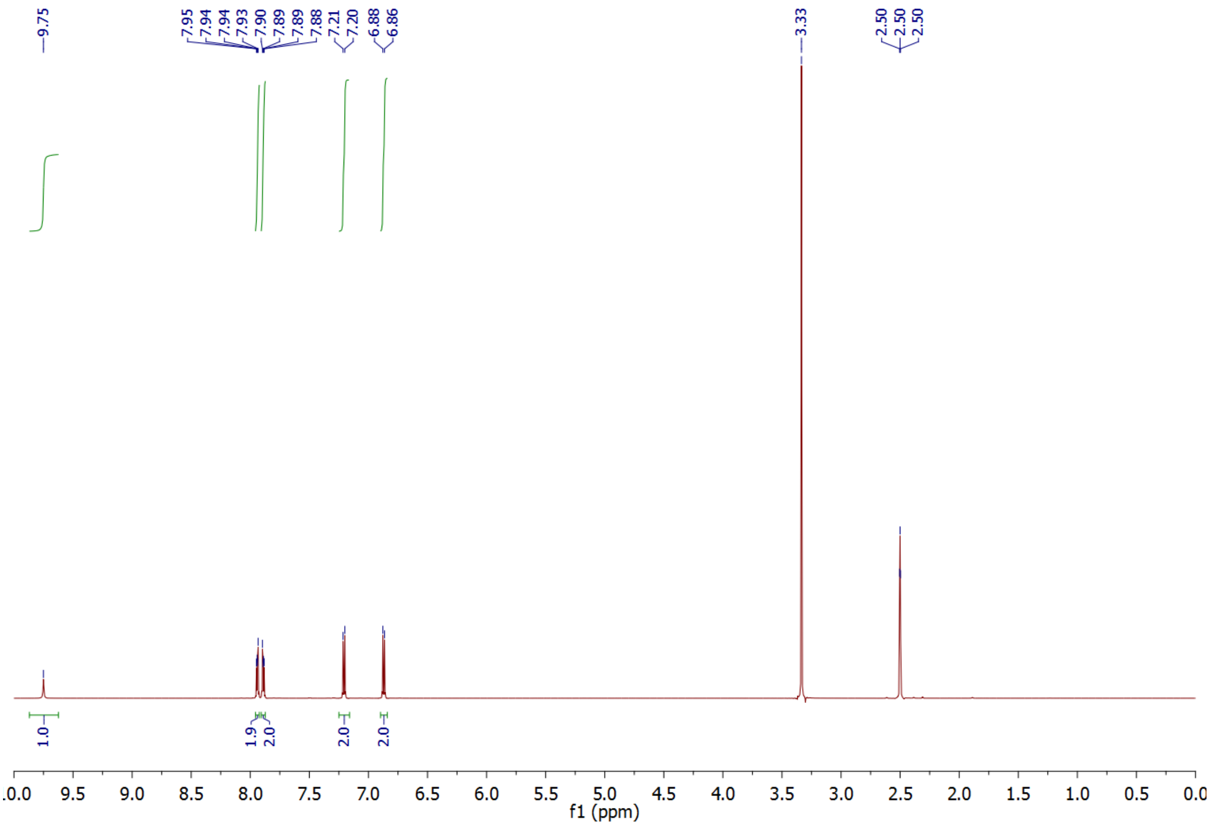

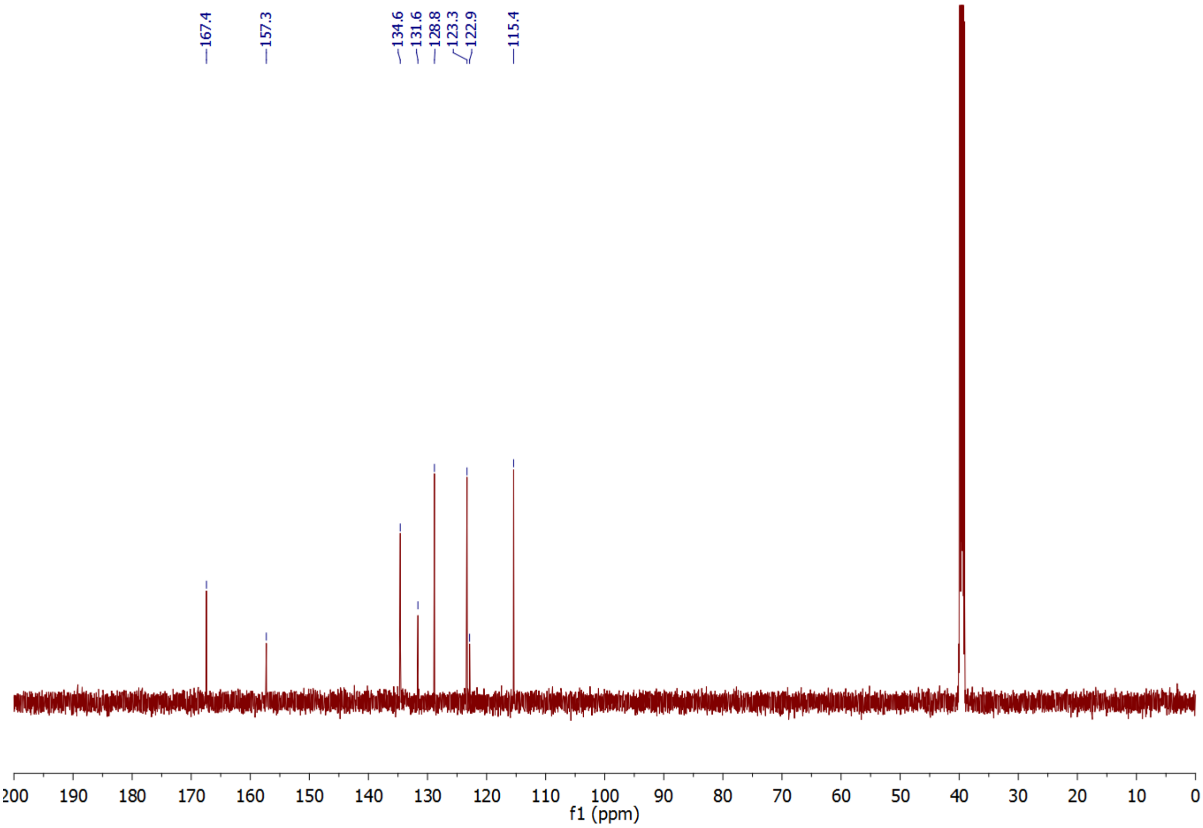
167.4
157.3
134.6
131.6
128.8
123.3
122.9
115.4
200 190 180 170 160 150 140 130 120 110 100 90 80 70 60 50 40 30 20 10 0
f1 (ppm)

((Oxybis(ethane-2,1-diyl))bis(oxy))bis(ethane-2,1-diyl) bis(4-methylbenzenesulfonate) S16[7]

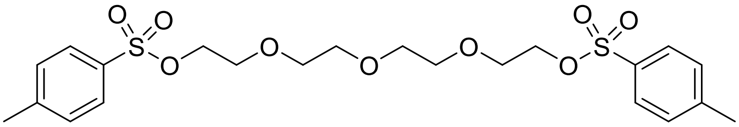

S16

To a stirred solution of tetra ethylene glycol (1.08 g, 5.56 mmol) in pyridine (3.6 mL) at 0 °C was added dropwise over 30 min tosyl chloride (2.33 g, 12.23 mmol, pre-dissolved in CH_2Cl_2 (5 mL)). The mixture was stirred for 6 h at 0 °C. Ice water (30 mL) was added to the reaction mixture and crude product extracted with EtOAc (3 x 30 mL). The organic layer was washed with HCl (2 M, 2 × 13 mL). The organic layer was then dried ($MgSO_4$), filtered and the solvent removed *in vacuo* to afford ((oxybis(ethane-2,1-diyl))bis(oxy))bis(ethane-2,1-diyl) bis(4-methylbenzenesulfonate) **S16** as a colourless oil (1.74 g, 3.45 mmol, 62%). ^{1}H NMR (600 MHz, $CDCl_3$) δ 7.80–7.78 (m, 4H), 7.34–7.33 (m, 4H), 4.16–4.14 (m, 4H), 3.68–3.67 (m, 4H), 3.58–3.54 (m, 8H), 2.44 (s, 6H); ^{13}C NMR (150 MHz, $CDCl_3$) δ 145.0 (C), 133.0 (C), 130.0 (CH), 128.1 (CH), 70.9 (CH_2), 70.7 (CH_2), 69.4 (CH_2), 68.8 (CH_2), 21.8 (CH_3). IR (thin film); 2913, 2870, 1550.

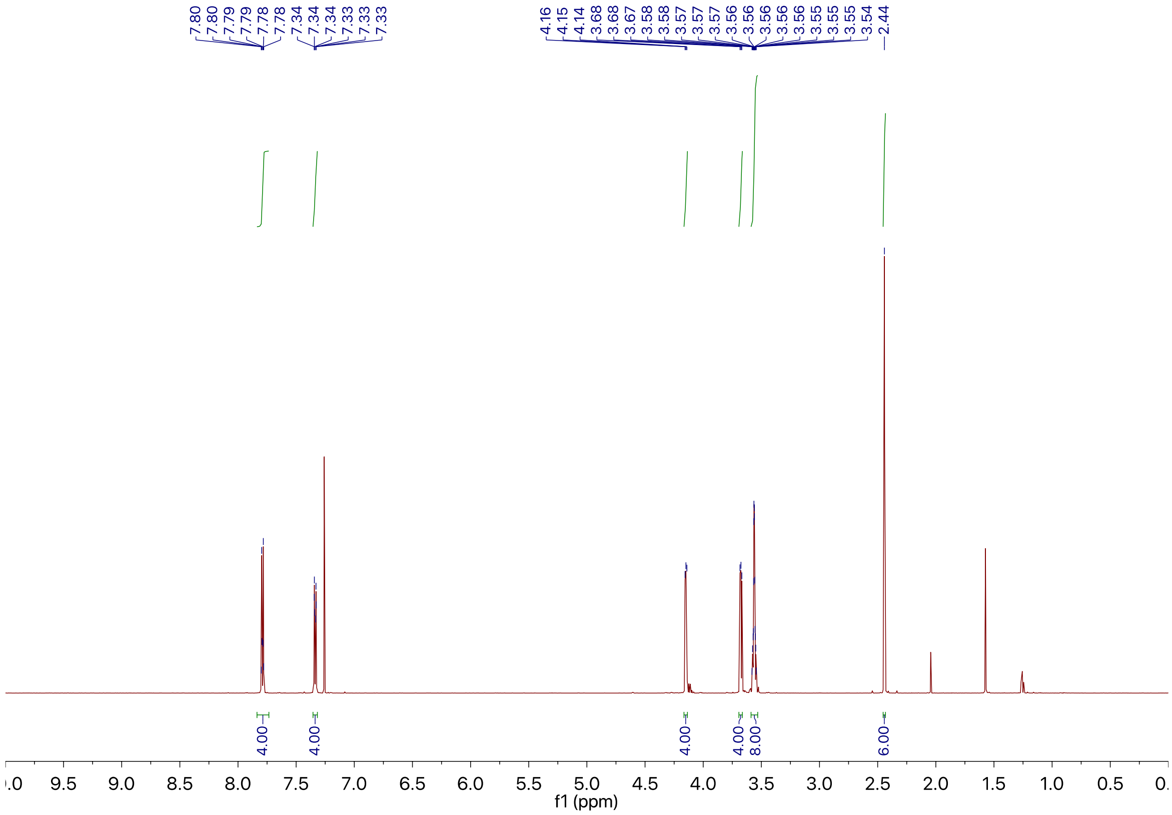

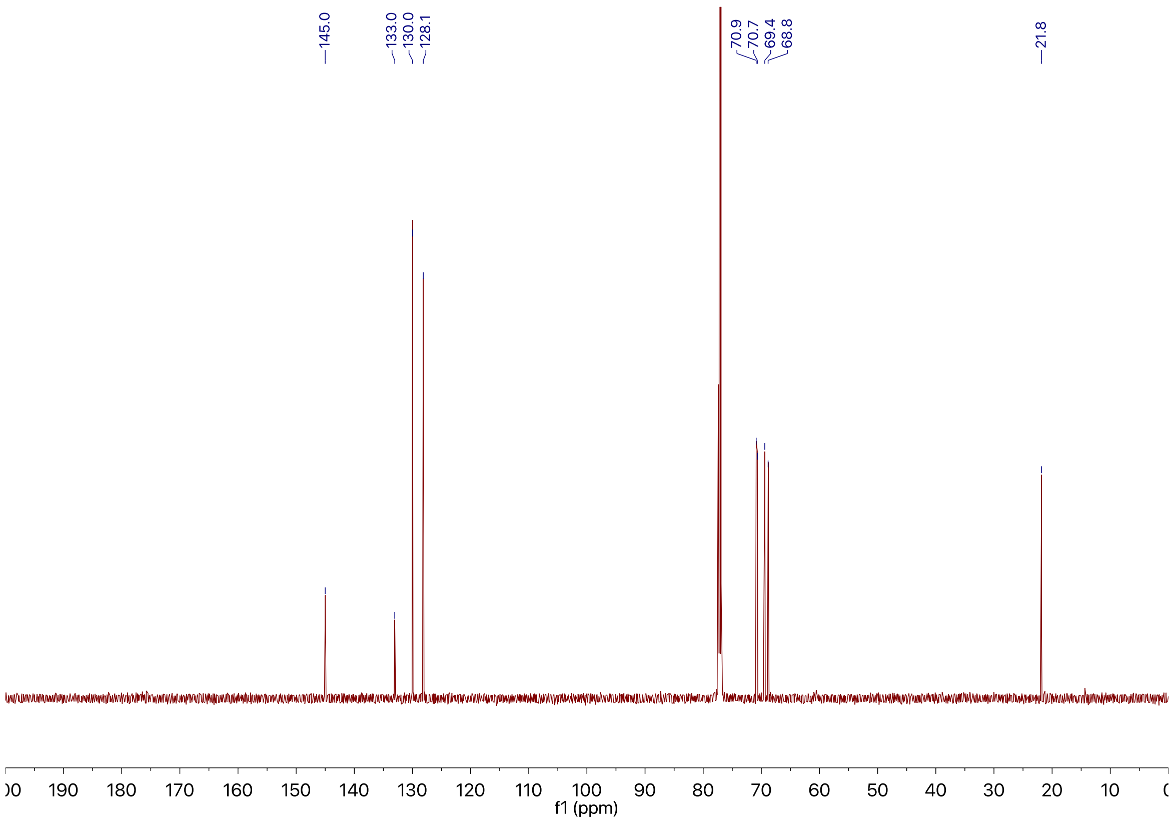

145.0
133.0
130.0
128.1
70.9
70.7
69.4
68.8
21.8
190
180
170
160
150
140
130
120
110
100
90
80
70
60
50
40
30
20
10
f1 (ppm)

2-(2-(2-(2-Azidoethoxy)ethoxy)ethoxy)ethyl 4-methylbenzenesulfonate S17[8]

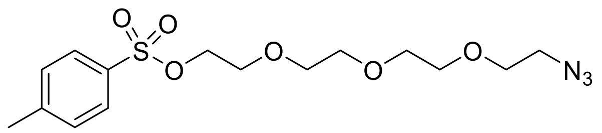

S17

To a solution of ((oxybis(ethane-2,1-diyl))bis(oxy))bis(ethane-2,1-diyl) bis(4-methylbenzenesulfonate) **S16** (0.24 g, 0.48 mmol) in ethanol (5 mL) was added sodium azide (0.033 g, 0.51 mmol). The resulting solution was heated at 80 °C for 16 h. The reaction was then poured into ice water (15 mL) and the product was extracted with ethyl acetate (3 x 15 mL). The organic extracts were washed with water (15 mL), brine (15 mL) and dried ($MgSO_4$), the resulting mixture was filtered and the solvent removed *in vacuo.* The crude residue was purified by flash column chromatography (25% to 80% EtOAc/petrol) to afford 2-(2-(2-(2-azidoethoxy)ethoxy)ethoxy)ethyl 4-methylbenzenesulfonate **S17** as a light brown oil (0.062 g, 0.168 mmol, 35%). ^{1}H NMR (600 MHz, $CDCl_3$) δ 7.79–7.77 (m, 2H), 7.33–7.32 (m, 2H), 4.15– 4.13 (m, 2H), 3.66–3.57 (m, 12H), 3.34 (t, *J* = 5.3 Hz, 2H), 2.41 (s, 3H, CH_3); ^{13}C NMR (150 MHz, $CDCl_3$) δ 144.9 (C), 133.1 (C), 129.9 (CH), 128.1 (CH), 70.9 (CH_2), 70.8 (CH_2), 70.8 (CH_2), 70.7 (CH_2), 70.1 (CH_2), 69.4 (CH_2), 68.8 (CH_2), 50.8 (CH_2), 21.8 (CH_3); IR (thin film); 2100, 2869 cm^{-1}.

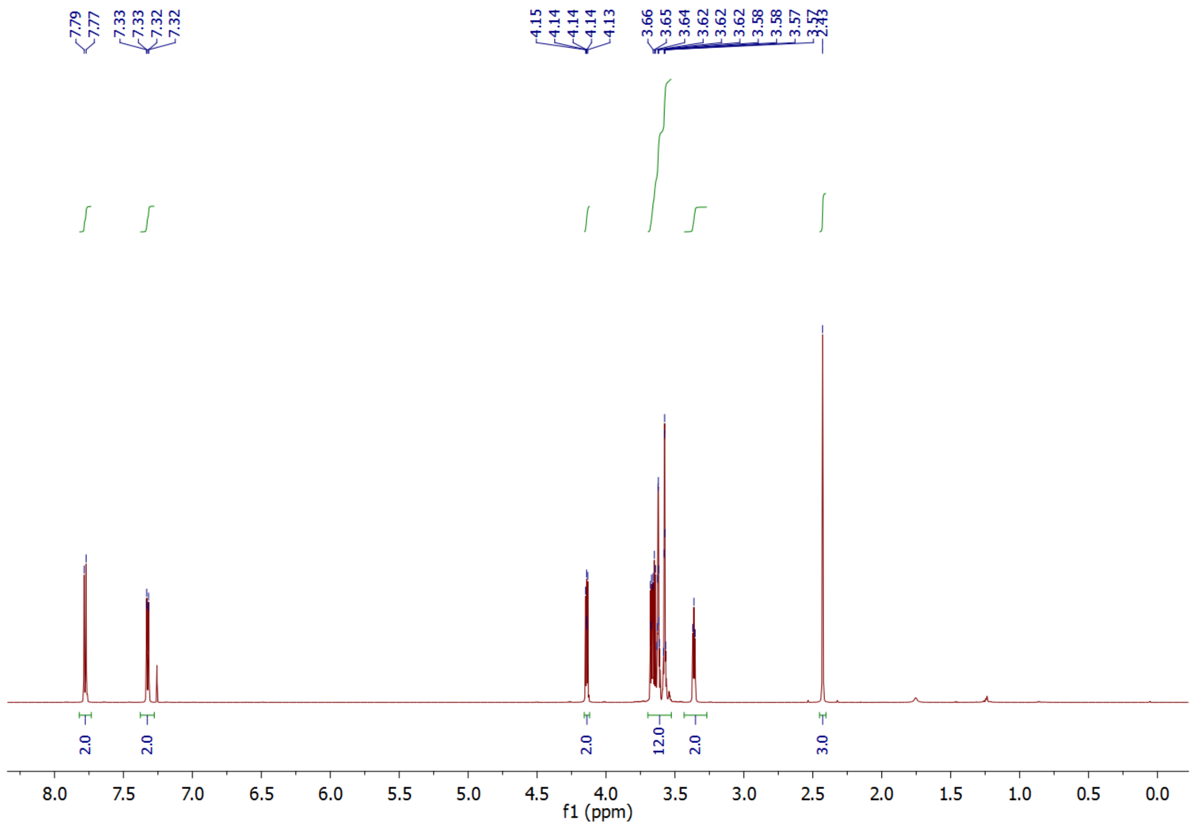
7.79
7.77
7.33
7.33
7.32
7.32
4.15
4.14
4.14
4.14
4.13
3.66
3.65
3.64
3.62
3.62
3.62
3.58
3.58
3.57
3.57
2.43
2.0
2.0
2.0
12.0
2.0
3.0
8.0 7.5 7.0 6.5 6.0 5.5 5.0 4.5 4.0 3.5 3.0 2.5 2.0 1.5 1.0 0.5 0.0
f1 (ppm)

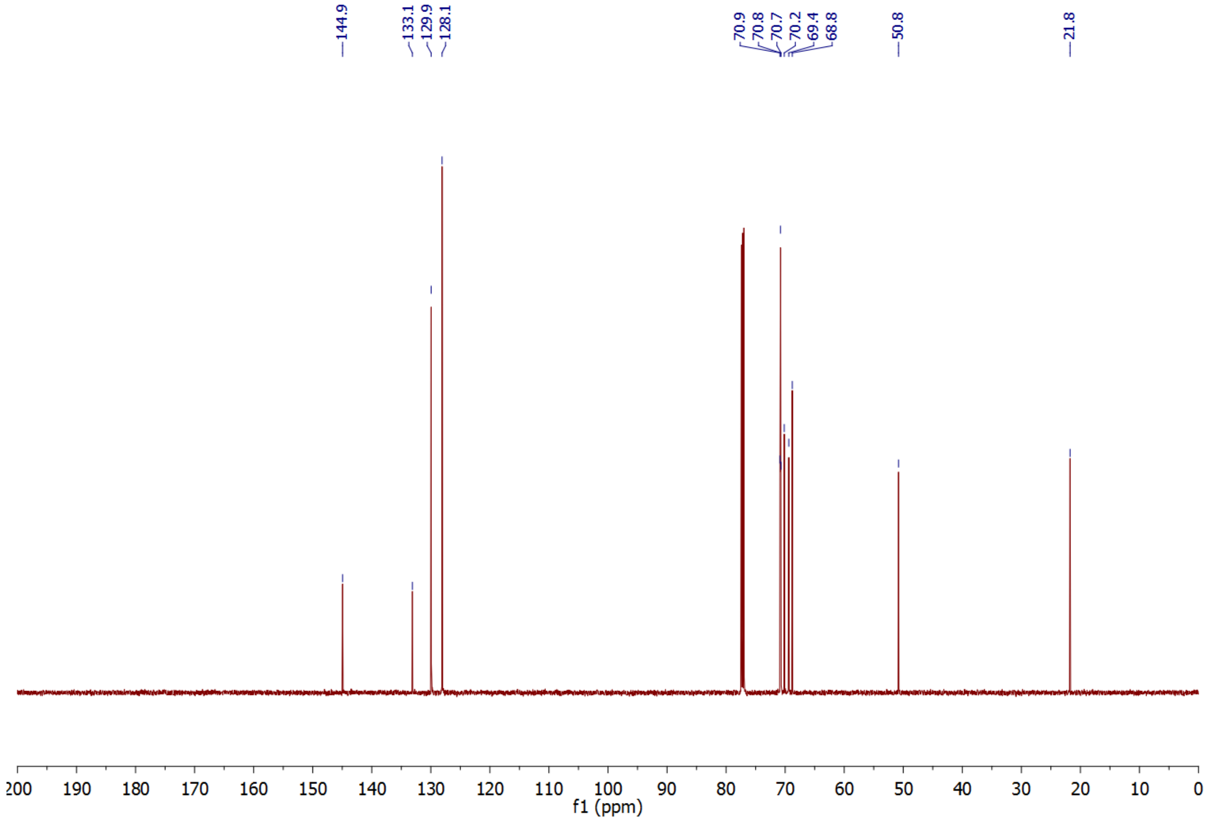
144.9
133.1
129.9
128.1
70.9
70.8
70.7
70.2
69.4
68.8
50.8
21.8
200 190 180 170 160 150 140 130 120 110 100 90 80 70 60 50 40 30 20 10 0
f1 (ppm)

2-(4-(2-(2-(2-(2-Azidoethoxy)ethoxy)ethoxy)ethoxy)phenyl)isoindoline-1,3-dione S18[9]

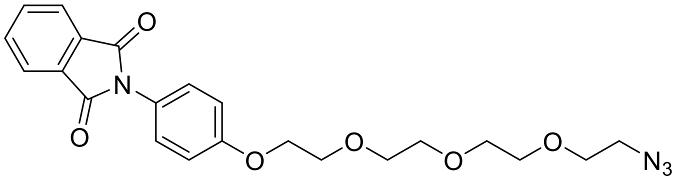

S18

To solution of potassium *tert*-butoxide (725 µL, 1M in THF) in DMF (20 mL), was added 2-(4-hydroxyphenyl)isoindoline-1,3-dione **S15** (189 mg, 0.79 mmol) and the reaction was stirred at 21 °C for 5 min. 2-(2-(2-(2-Azidoethoxy)ethoxy)ethoxy)ethyl 4-methylbenzenesulfonate **S17** (245 mg, 0.66 mmol) was then added, and the reaction mixture was stirred at 90 °C for 16 h. The reaction was diluted with water (100 mL), and the crude product was extracted with EtOAc (3 × 30 mL). The organic extracts were combined and washed with saturated lithium chloride solution (2 × 30 mL) and then dried ($MgSO_4$). The solvent was removed *in vacuo* and the crude residue was purified by flash column chromatography (25% to 80% EtOAc/petrol) to afford 2-(4-(2-(2-(2-(2-azidoethoxy)ethoxy)ethoxy)ethoxy)phenyl)isoindoline-1,3-dione **S18** as a clear yellow oil (202 mg, 0.46 mmol, 70%). ^{1}H NMR (600 MHz, $CDCl_3$) δ 7.89–7.88 (m, 2H), 7.74–7.69 (m, 2H), 7.30–7.28 (m, 2H), 7.00–6.99 (m, 2H), 4.14–4.13 (m, 2H), 3.86–3.82 (m, 2H), 3.72–3.69 (m, 2H), 3.64–3.61 (m, 8H), 3.35–3.33 (m, 2H); ^{13}C NMR (150 MHz, $CDCl_3$) δ 167.6 (C), 158.6 (C), 134.4 (CH), 131.9 (C), 128.0 (CH), 124.6 (C), 123.7 (CH), 115.2 (CH), 71.0 (CH_2), 70.8 (CH_2), 70.8 (CH_2), 70.8 (CH_2), 70.1 (CH_2), 69.7 (CH_2), 67.8 (CH_2), 50.8 (CH_2); IR (thin film): 2864, 2112, 1704 cm^{-1}.

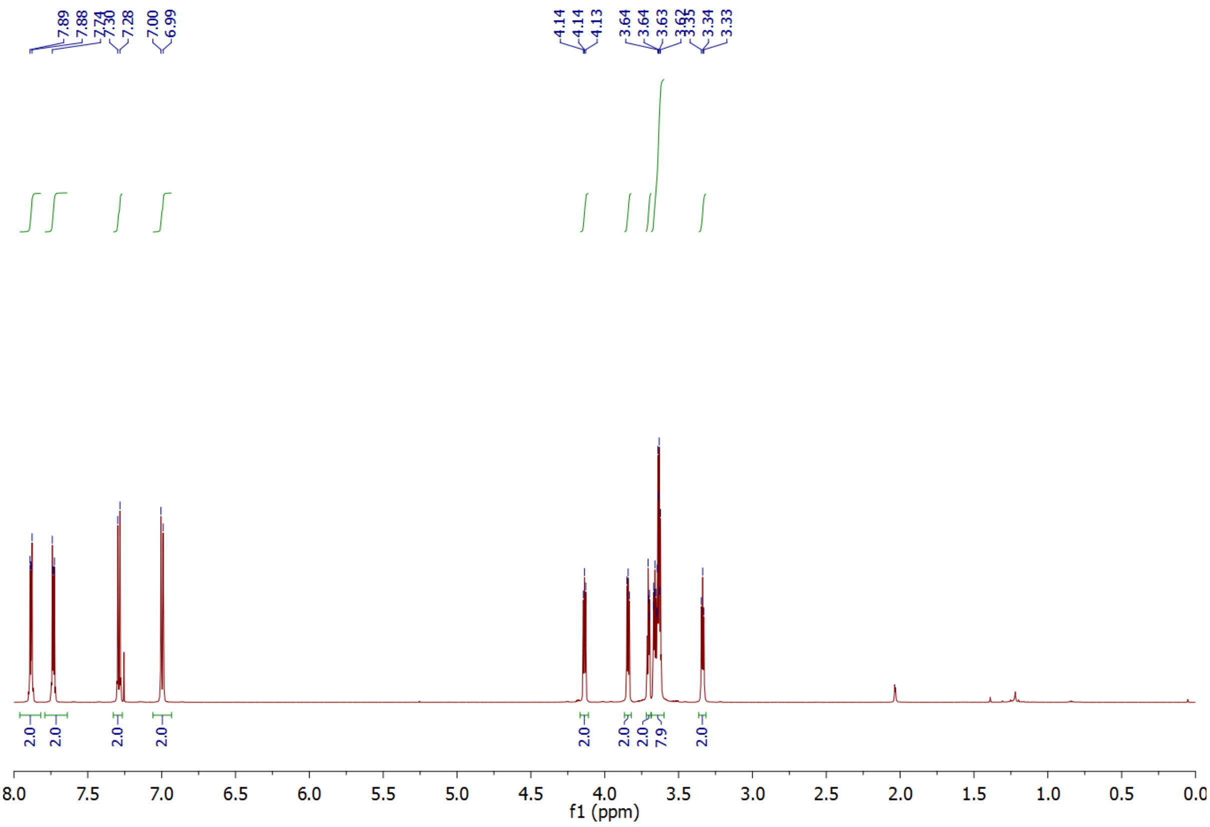
7.89
7.88
7.74
7.30
7.28
7.00
6.99
4.14
4.14
4.13
3.64
3.64
3.63
3.62
3.35
3.34
3.33
2.0
2.0
2.0
2.0
2.0
2.0
2.0
7.9
2.0
f1 (ppm)

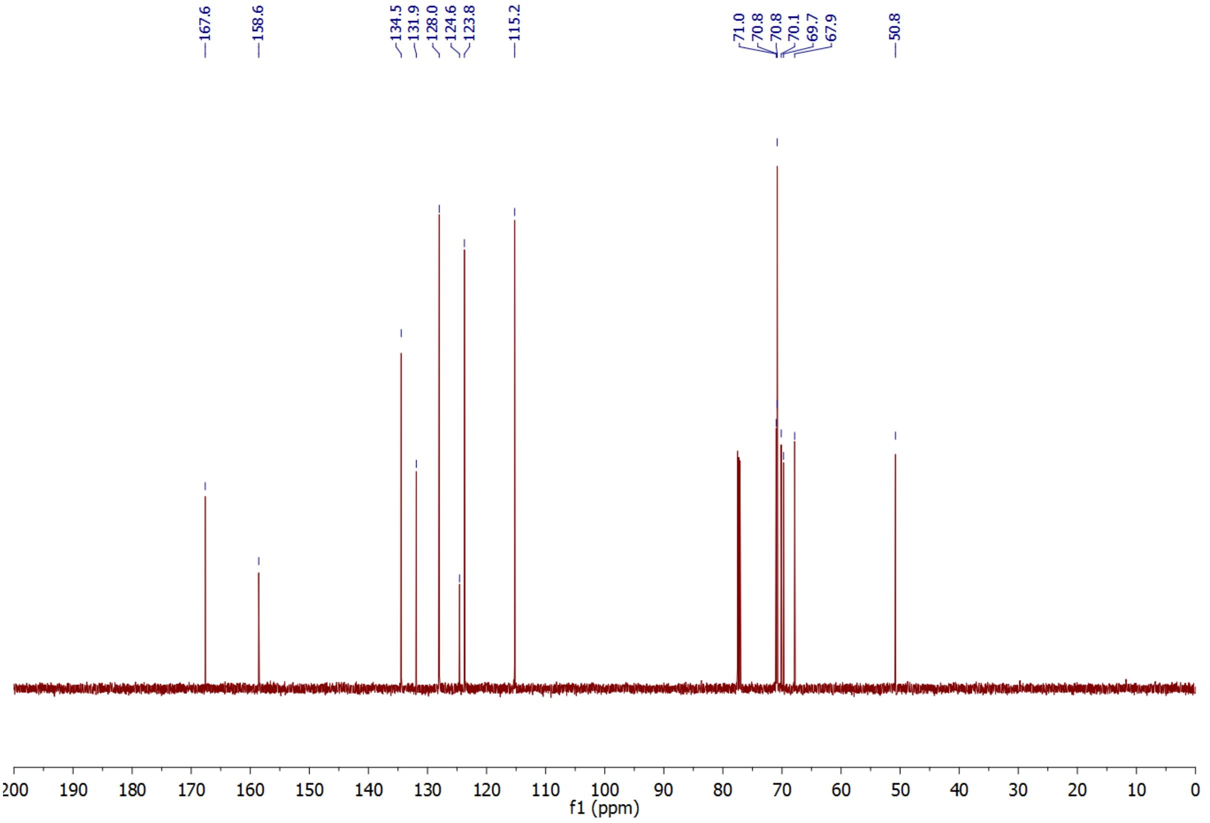
167.6
158.6
134.5
131.9
128.0
124.6
123.8
115.2
71.0
70.8
70.8
70.1
69.7
67.9
50.8
f1 (ppm)

4-(2-(2-(2-(2-Azidoethoxy)ethoxy)ethoxy)ethoxy)aniline 16[9]

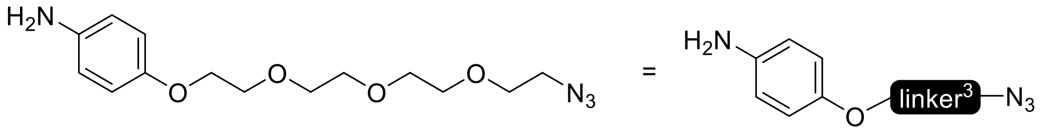

16 **16**

To 2-(4-(2-(2-(2-(2-azidoethoxy)ethoxy)ethoxy)ethoxy)phenyl)isoindoline-1,3-dione **S18** (141 mg, 0.32 mmol) in THF (10 mL) was added hydrazine monohydrate (330 μL, 60% solution). The reaction was then stirred for 2 h at 21 °C. The reaction mixture was diluted with water (30 mL) and the product was extracted with EtOAc (3 × 30 mL), washed with brine (20 mL), and dried ($MgSO_4$). The solvent was removed *in vacuo* and the crude residue was purified by flash column chromatography (20% to 100% EtOAc/petrol) to afford 4-(2-(2-(2-(2-azidoethoxy)ethoxy)ethoxy)ethoxy)aniline **16** as a brown oil (73 mg, 0.23 mmol, 73%).^{1}H NMR (600 MHz, $CDCl_3$) δ 6.74–6.73 (m, 2H), 6.62–6.60 (m, 2H), 4.06– 4.03 (m, 2H), 3.80–3.79 (m, 2H), 3.70–3.69 (m, 2H), 3.69–3.64 (m, 8H), 3.43 (s, 2H), 3.36 (t, *J* = 5.1 Hz, 2H); ^{13}C NMR (150 MHz, $CDCl_3$) δ 152.0 (C), 140.4 (C), 116.4 (CH), 116.0 (CH), 70.9 (CH_2), 70.8 (CH_2), 70.8 (CH_2), 70.8 (CH_2), 70.1 (CH_2), 70.0 (CH_2), 50.8 (CH_2); IR (thin film); 3430, 3356, 2869, 2098 cm^{-1}; LRMS (ESI) 311 (100, $[M+H]^+$); HRMS (ES$^+$) calcd for $C_{14}H_{22}N_4O_4$ $[M+H]^+$ 311.1733, observed 311.1719.

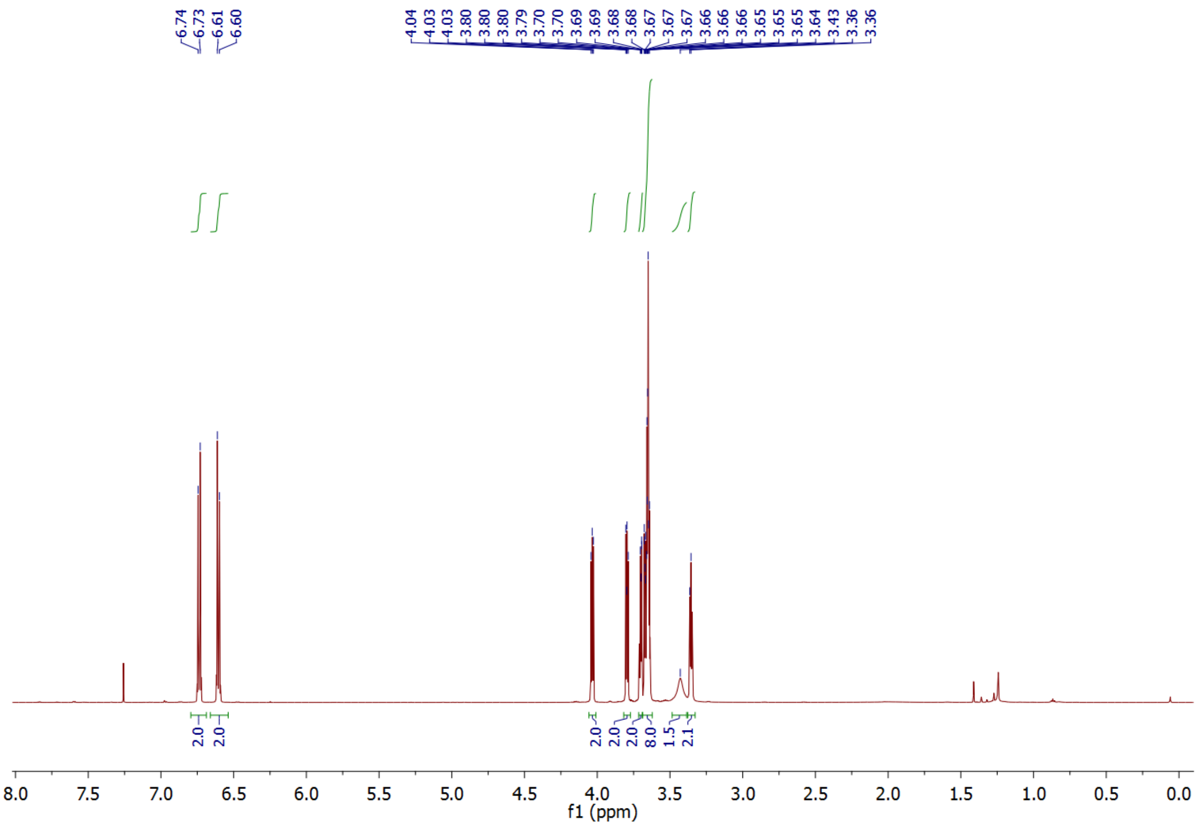
6.74
6.73
6.61
6.60
4.04
4.03
4.03
3.80
3.80
3.79
3.70
3.69
3.69
3.68
3.68
3.67
3.67
3.67
3.66
3.66
3.66
3.65
3.65
3.65
3.64
3.43
3.36
3.36
2.0
2.0
2.0
2.0
2.0
8.0
1.5
2.1
f1 (ppm)

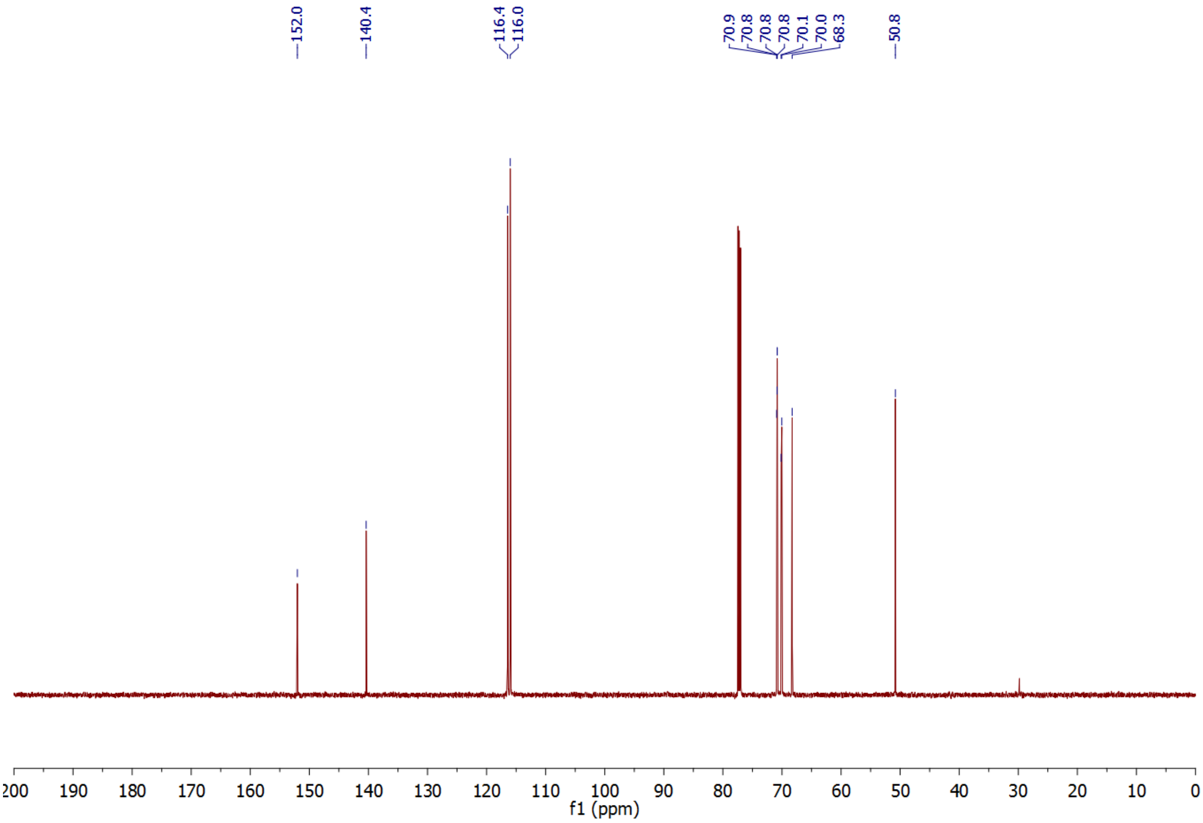
152.0
140.4
116.4
116.0
70.9
70.8
70.8
70.8
70.1
70.0
68.3
50.8
f1 (ppm)

Chemical Biology

Solid Phase Peptide Synthesis (SPPS) (FEKGC peptide 12)[10]

Peptides were synthesised *via* manual solid phase peptide synthesis (SPPS) using an *in situ* neutralisation/HCTU activation procedure for Fmoc chemistry on an H-Cys(Trt)-2-ClTrt resin (Sigma) using Fmoc protected amino acids as described below:

Preloaded resin preparation: The preloaded 2-chlorotrityl resin was weighed out into a 5 mL SPPS cartridge fitted with a Polytetrafluoroethylene (PTFE) stopcock, swollen in DMF for 30 min and then filtered.

Amino acid coupling: DIPEA (11.0 eq.) was added to a solution of amino acid (5.0 eq.) and HCTU (5.0 eq.) dissolved in the minimum volume of DMF and the solution added to the resin. The reaction mixture was gently agitated by rotation for 1 h, and the resin filtered off and washed with DMF (3 × 2 min with rotation).

Fmoc deprotection: A solution of 20% piperidine in DMF was added to the resin and gently agitated by rotation for 2 minutes. The resin was filtered off and repeated four more times, followed by washes with DMF (5 × 2 min with rotation).

Cleavage and Isolation: Resins containing full synthesised peptides were washed with DCM (3 × 2 min with rotation) and MeOH (3 × 2 min with rotation). The resin was dried on a vacuum manifold and further dried on a high vacuum line overnight. A solution of cleavage cocktail 95:2.5:2.5 (v/v) TFA:H_2O:triisopropylsilane was then added to the resin, and the resulting mixture was gently agitated by rotation for 60 min. The reaction mixture was drained into ice-cold Et_2O and centrifuged at 6000 rpm at 4 °C until pelleted (*ca*. 5-10 min). The supernatant was carefully decanted and subsequently resuspended, centrifuged and supernatant decanted three more times. The precipitated peptide pellet was then either dissolved 10% MeCN or in 10% aq. AcOH and lyophilised. Lyophilised peptides were then stored at -20 ^{o}C until required.

FEKGC 12

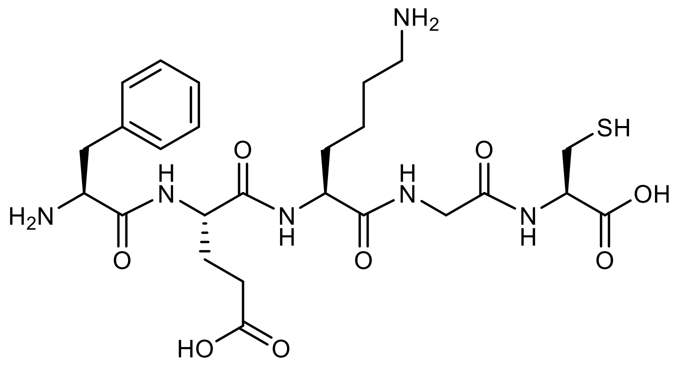

Synthesised using 200 mg resin (0.19 mmol g^{-1} loading).

Yield = 18 mg (82 %).

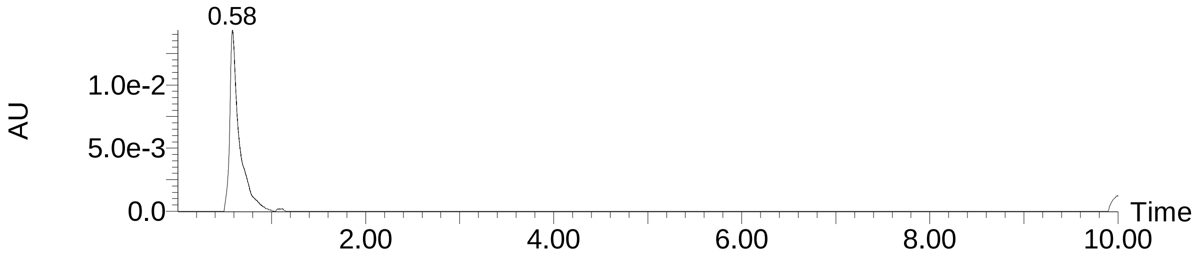

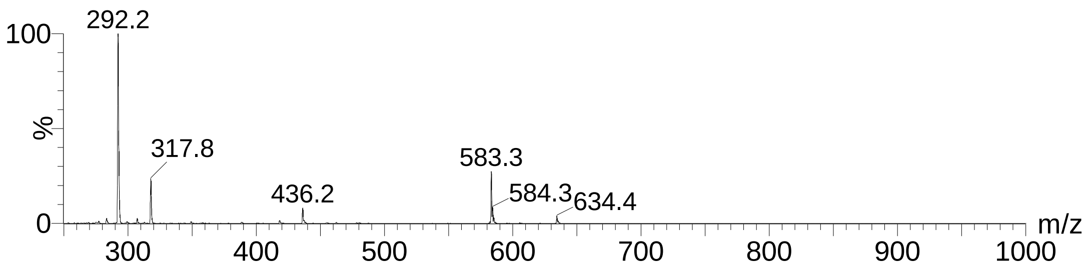

LRMS: Found $[M+H]^+$ 583.3, $C_{25}H_{39}N_6O_8S$ requires 583.7. **HPLC**: t_R 0.58 min

Cysteine Mutant Green Fluorescent Protein (GFPS147C) 1[11]

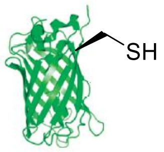

1

GFPS147C **1** was expressed in competent *E. coli* BL21(DE3) cells as described previously.[11] A two step purification (His-Tag and SEC) was carried out as follows:

The cell pellets were re-suspended in 30 mL binding buffer (100mM Sodium Phosphate, 25mM imidazole, pH 8) and stored overnight at -20 °C. Cells were lysed in the presence of two tablets of a cocktail of EDTA-free Complete Protease Inhibitors (Roche) by sonication on ice, for 6 x 30 sec bursts with 1 min cooling intervals using a stud probe on the Sonicator 400 at amplitude 10. The resultant cell lysate was centrifuged at 35,000 g for 30min at 4 °C and the supernatant 0.22 µm syringe filtered before loading onto a 5 mL HisTrap HP column (GE Healthcare) equilibrated in binding buffer. Bound material was eluted with a 25-500 mM imidazole gradient and fractions analysed by SDS-PAGE for pooling.

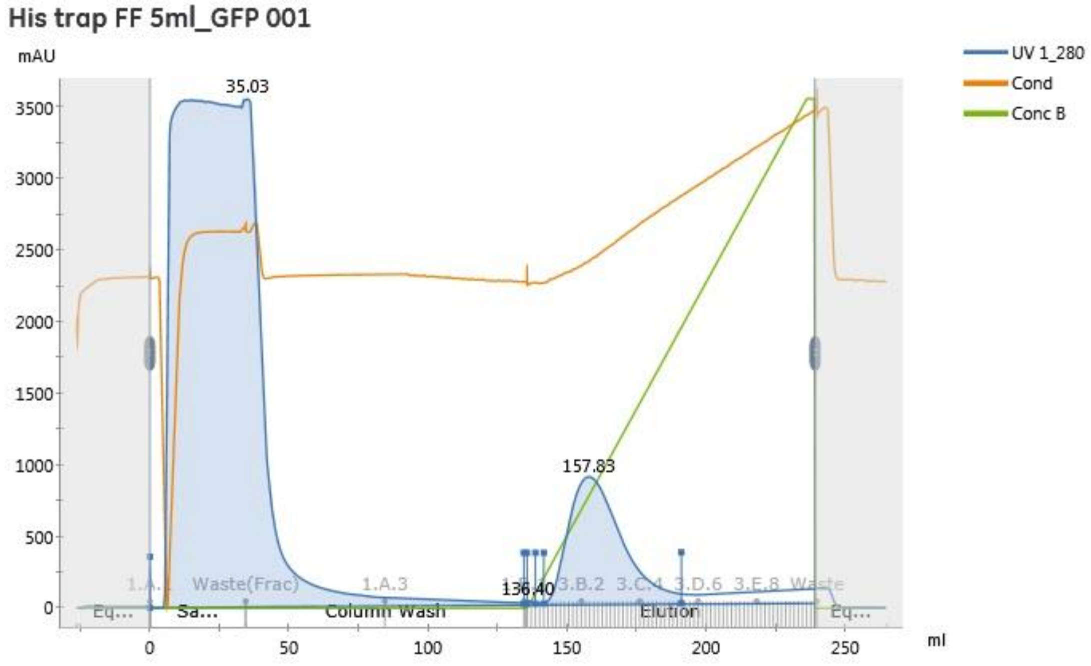

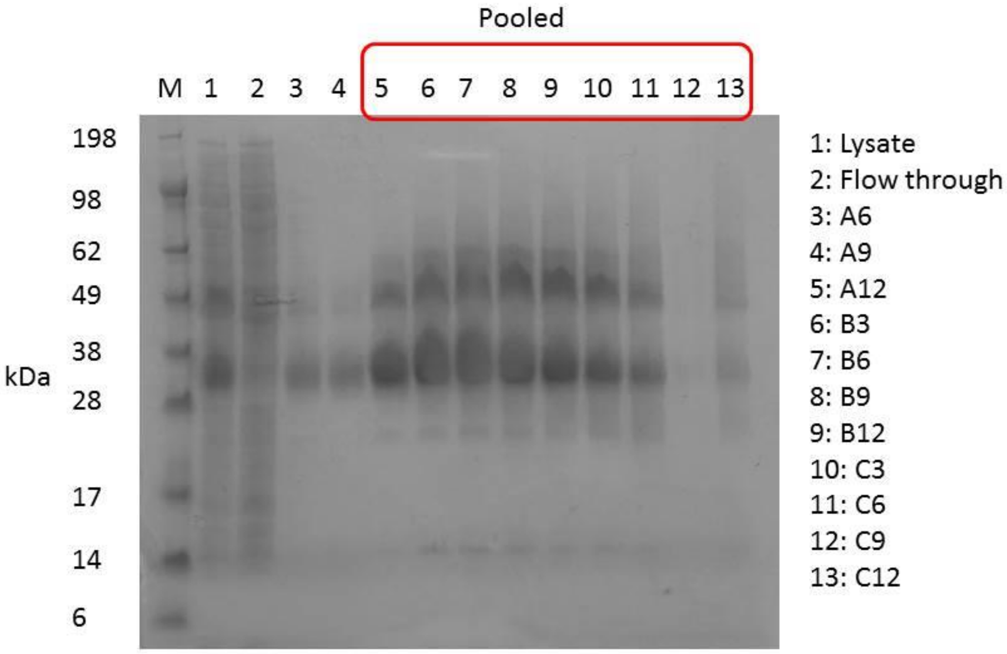

Sample was loaded onto a Superdex 75 10/300 column (GE Healthcare) equilibrated in 100mM Sodium Phosphate, pH 7.4 and eluted fractions pooled appropriately as indicated by SDS-PAGE. Formation of the correct product was confirmed by electrospray mass spectrometry.

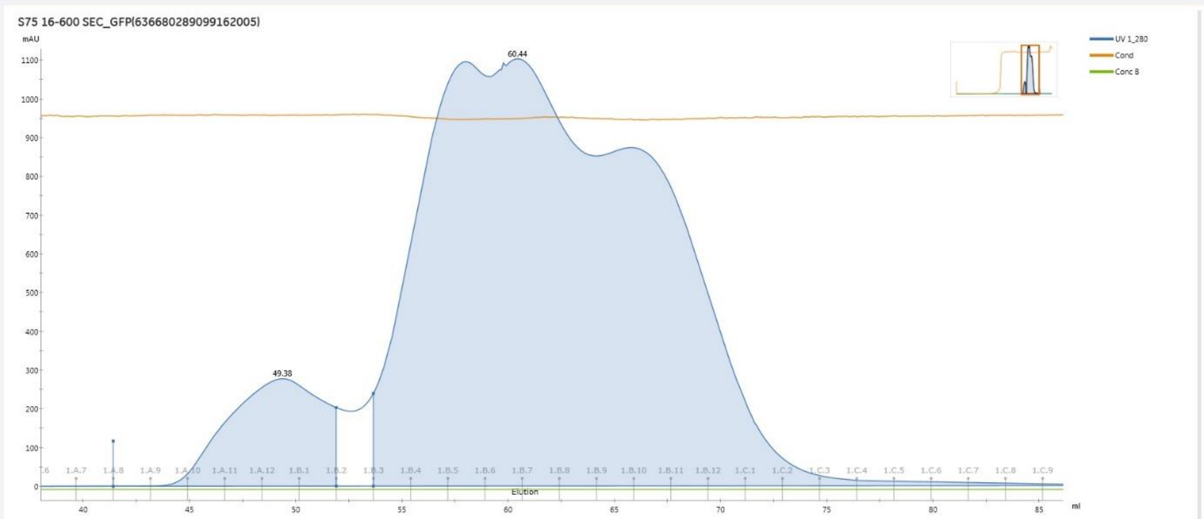

Peak Table - UV 1_280

Peak	Retention ml	Area ml*mAU	Area %	Ext coeff. mg ml⁻¹ cm⁻¹	Fraction(s)	Volume ml	Conductivity mS/cm
Peak A	49.375	1431	9.01		1.A.8 - 1.B.1	10.500	10.52
Peak B	60.442	14448	90.99		1.B.3 - 1.C.9	33.257	10.47

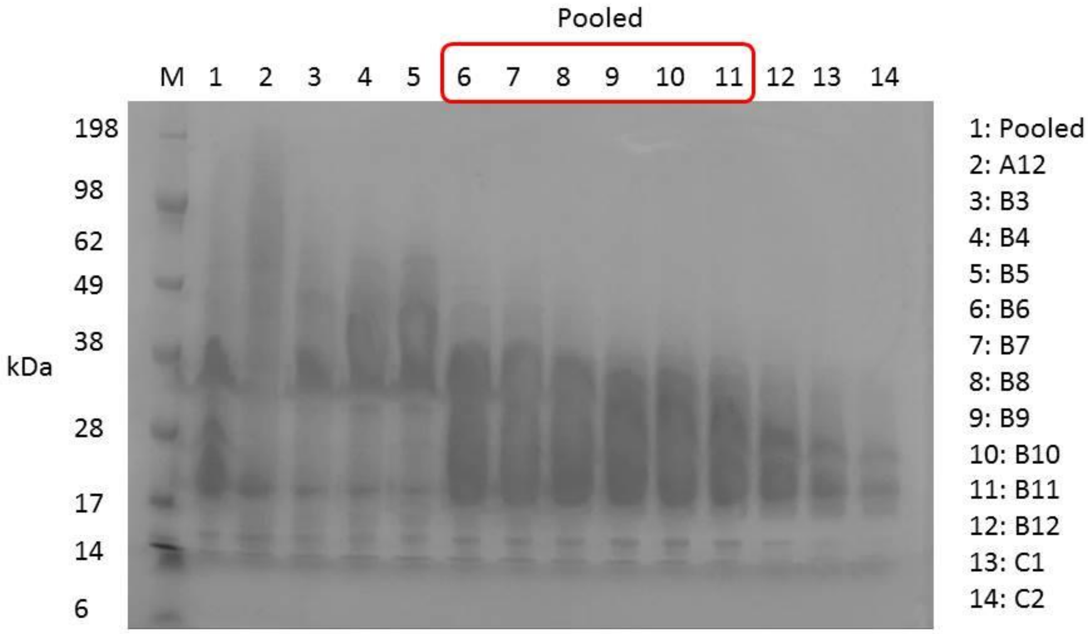

Due to the presence of a small amount of dimer species, GFPS147C **1** was then reduced prior to conjugation and characterised as below:

Reduction of GFPS147C

TCEP (31.3 µL, 20 mM in deionised water, 25 eq.) was added to a solution of GFPS147C **1** (500 µL, 50 µM) in PBS (pH 7.4, 5 mM EDTA) and the solution was incubated at 37 °C for 90 min. Excess reagents were removed by ultrafiltration (6 × 10000 MWCO, VivaSpin®, GE Healthcare) into PBS (pH 7.4, 5 mM EDTA) for further experiments. Samples were desalted (7000 MWCO, ZebaSpin®, Thermo Scientific) prior to LCMS analysis. Concentration was determined photometrically using $\varepsilon_{280} = 20{,}500\ M^{-1}\,cm^{-1}$. Observed masses (LCMS Method 1): 29355 Da, 29538 Da.

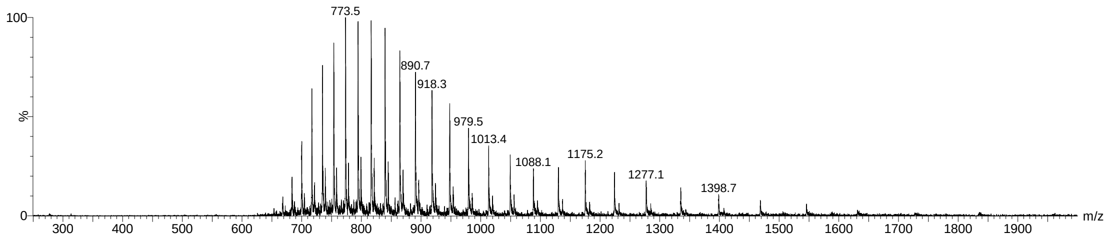

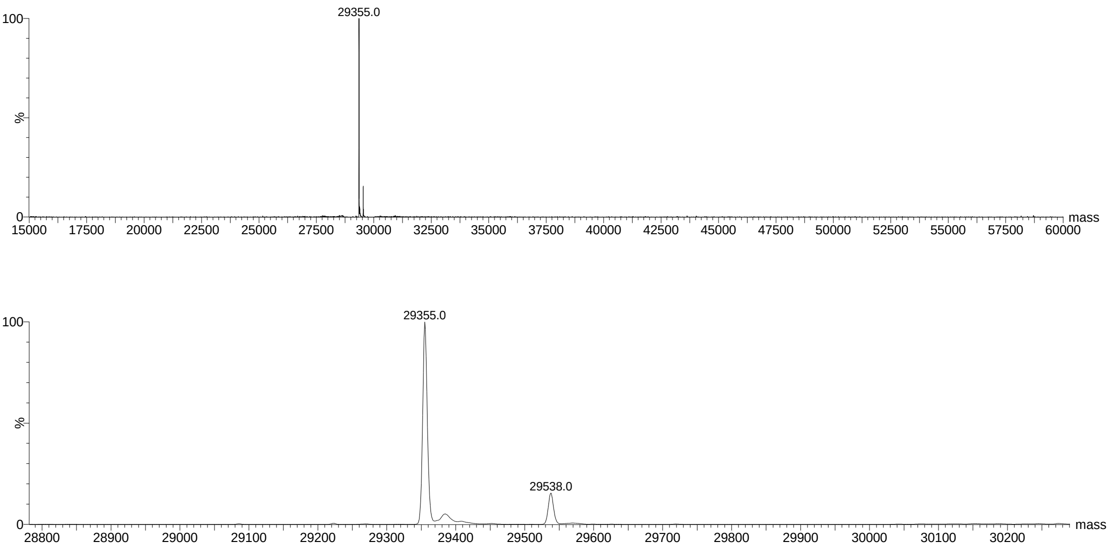

29355.0
100
%
0
mass
15000
17500
20000
22500
25000
27500
30000
32500
35000
37500
40000
42500
45000
47500
50000
52500
55000
57500
60000
29355.0
29538.0
100
%
0
mass
28800
28900
29000
29100
29200
29300
29400
29500
29600
29700
29800
29900
30000
30100
30200

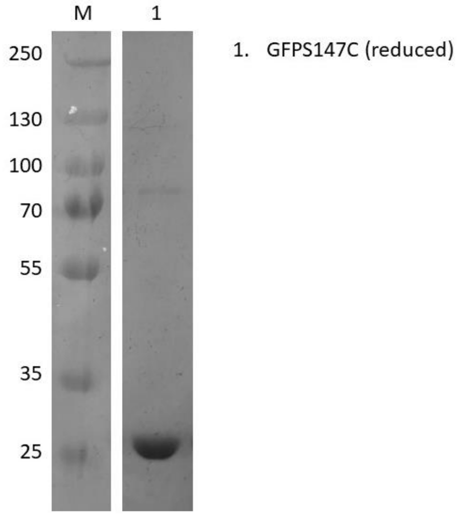

M
1
250
130
100
70
55
35
25
1. GFPS147C (reduced)

GFPS147C–PD (2-4, 9) Bioconjugations

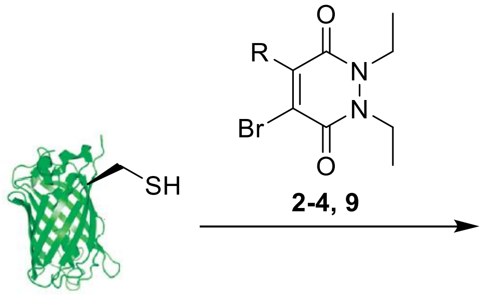

1

To a solution of reduced GFPS147C **1** (100 µL, 50 µM in BBS pH 8.0, 5 mM EDTA) was added Pyridazinedione species **2-4** and **9** (5 µL, 20 mM in DMSO, 20 eq., 1 mM final concentration) and the solution was incubated at 37 °C for 4 h. Excess reagents were removed using desalting columns (7000 MWCO, ZebaSpin®, Thermo Scientific) prior to LCMS analysis.

N,N'-Diethyl-Br-Hexylamine PD **9** Expected masses: 29605 Da. Observed masses (LCMS Method 3): 29340, 58680 Da.

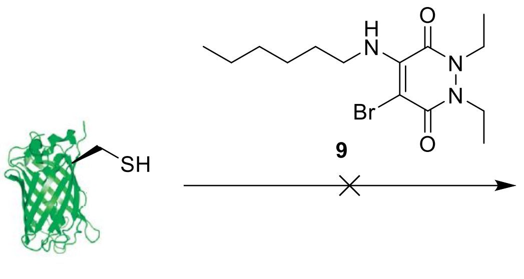

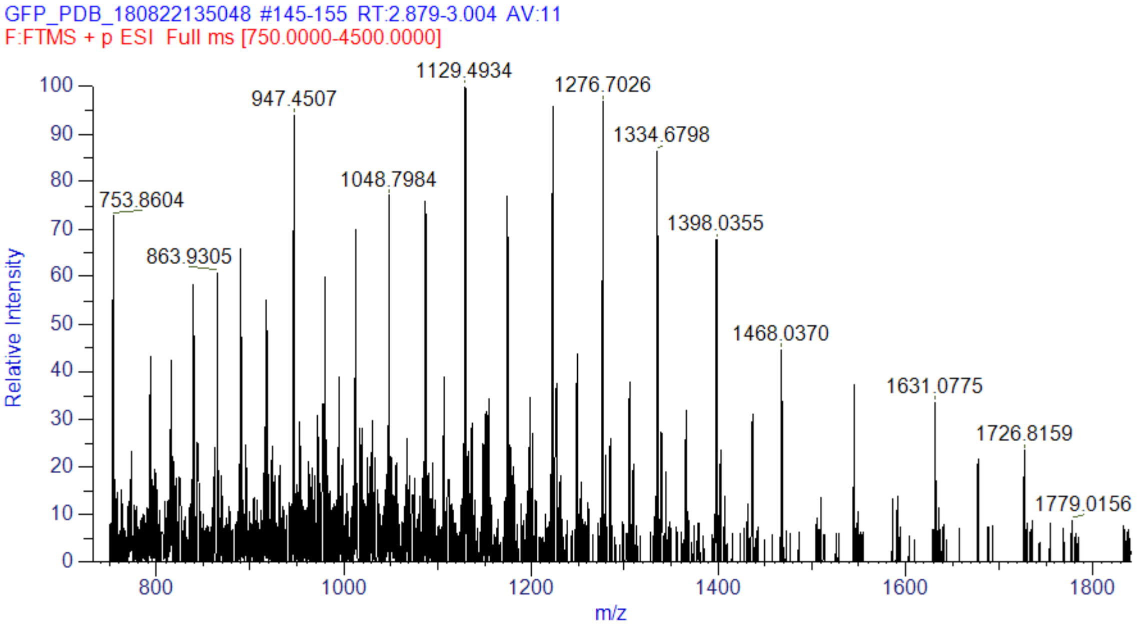

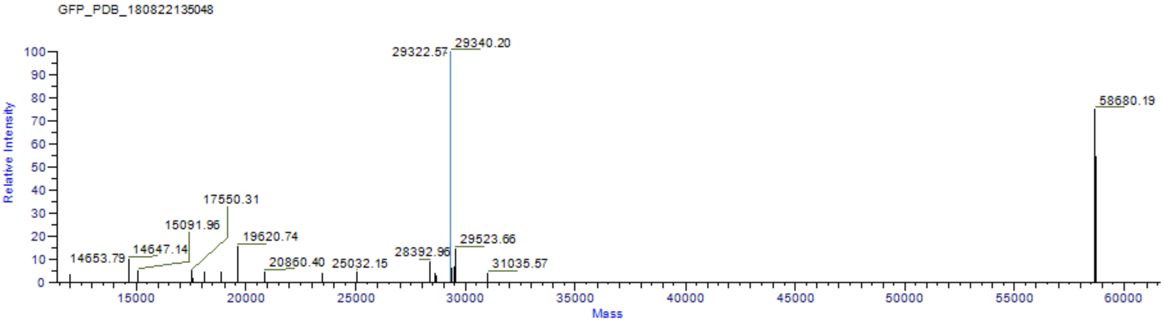

N,N′-Diethyl-Br-Hexanethiol PD **2** Expected masses: 29622 Da. Observed masses (LCMS Method 3): 29622 Da.

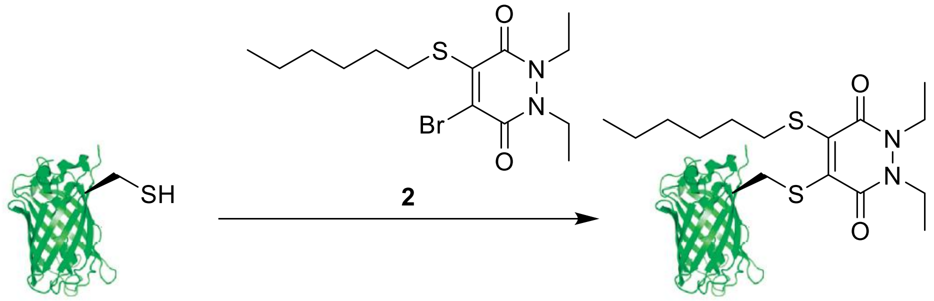

1 **5**

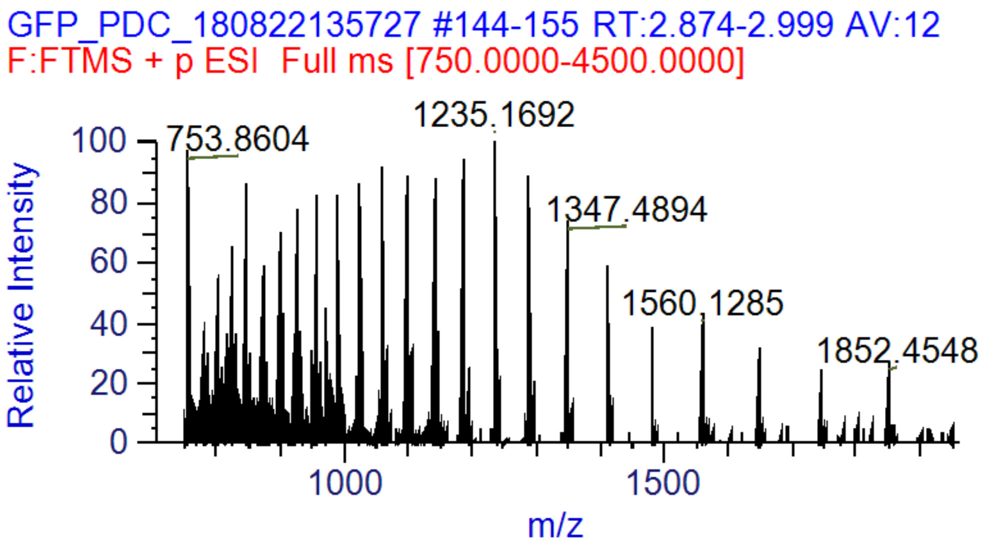

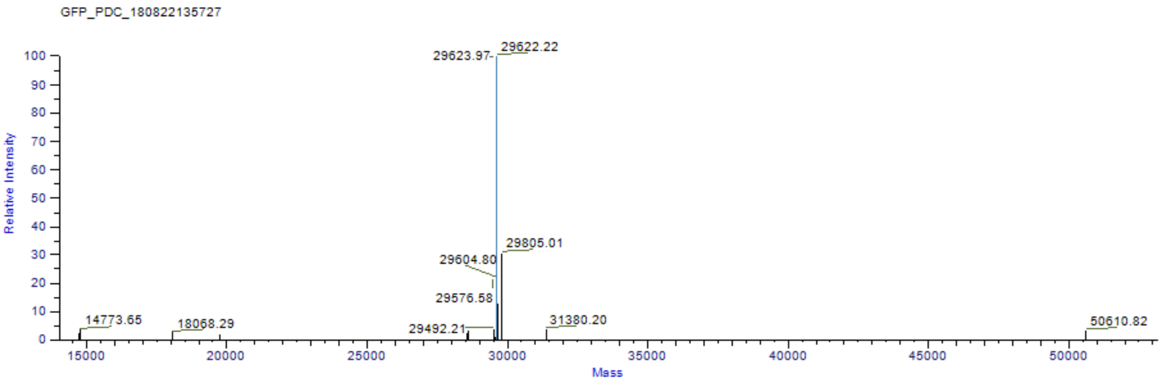

N,N'-Diethyl-Br-2-Mercaptoethanol PD **3** Expected masses: 29582 Da. Observed masses (LCMS Method 3): 29582 Da.

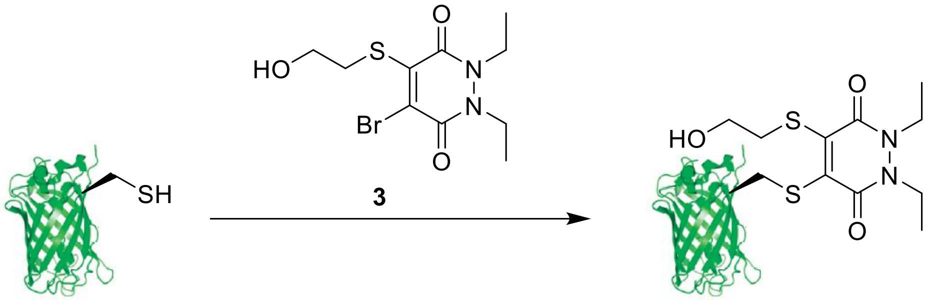

1 **6**

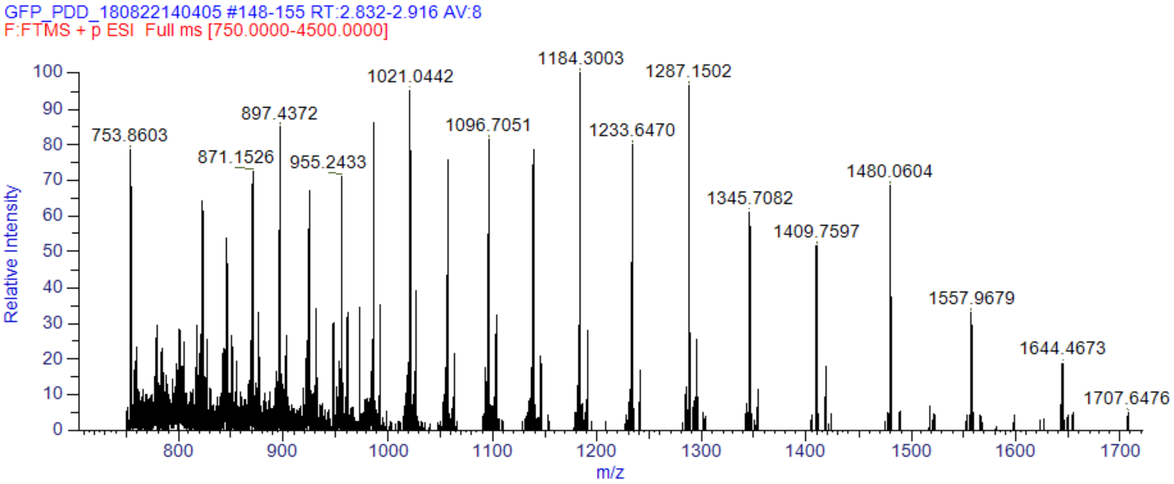

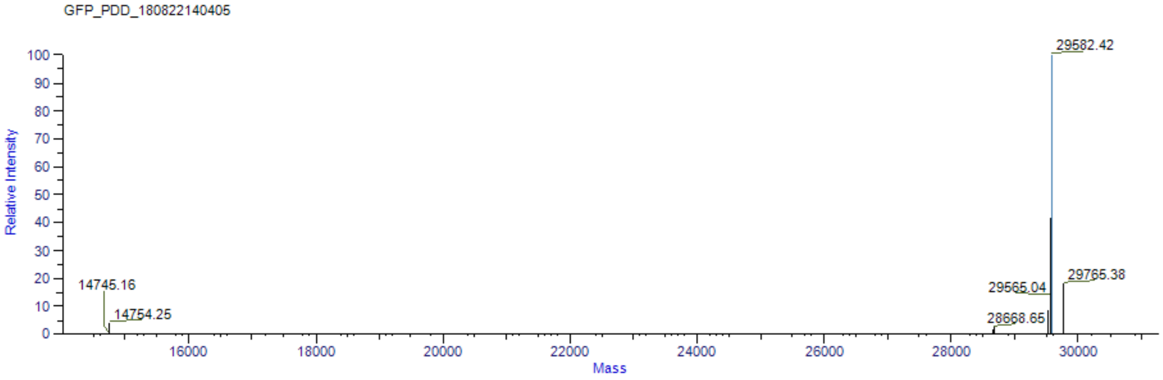

N,N′-Diethyl-Br-3-Mercaptopropanoic acid PD **4** Expected masses: 29610 Da. Observed masses (LCMS Method 3): 29610 Da.

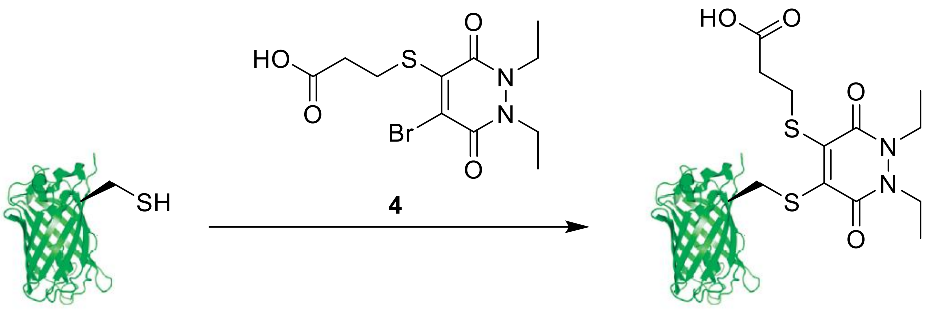

1 **7**

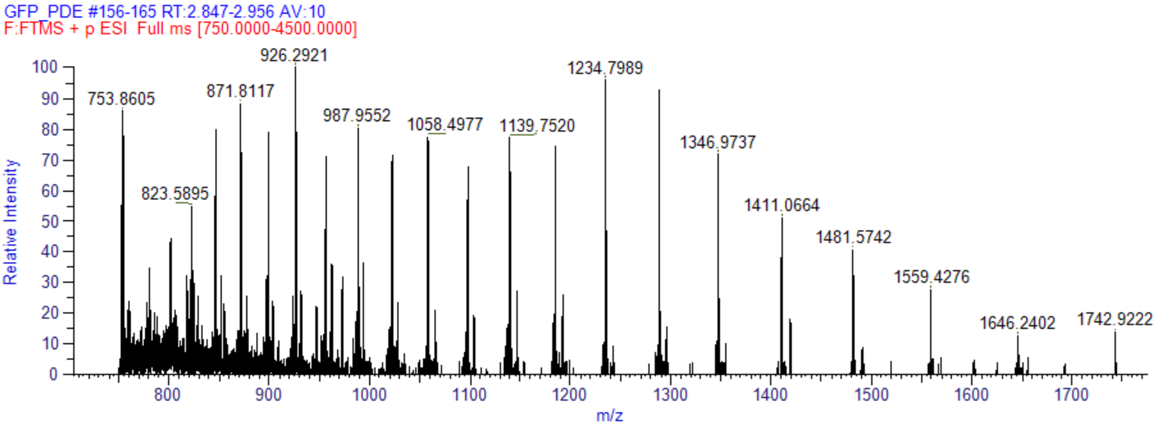

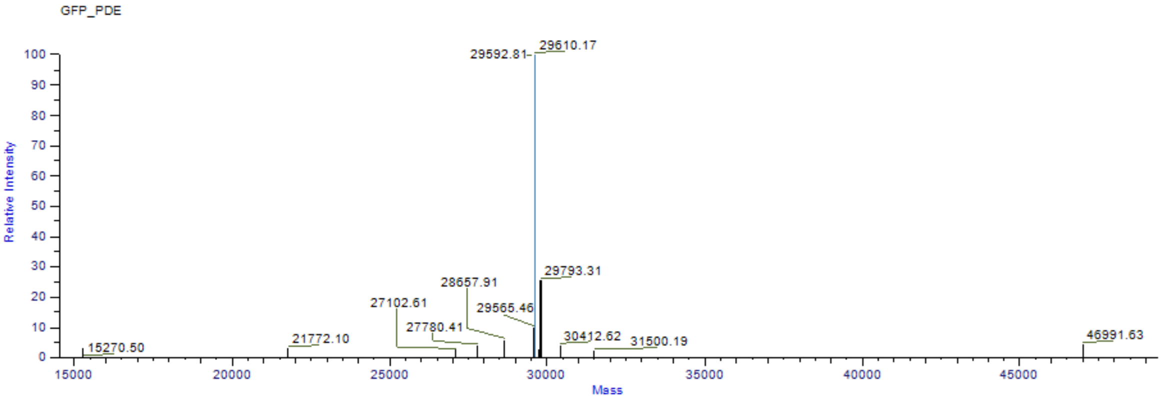

GFPS147C–PD (*N,N'*-diethyl)–Br 8

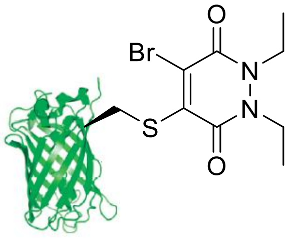

8

To a solution of reduced GFPS147C **1** (500 µL, 50 µM in BBS pH 8.0, 5 mM EDTA) was added *N,N*-diethyl dibromo pyridazinedione **10** (25 µL, 20 mM in DMSO, 20 eq., 1 mM final concentration) and the solution was incubated at 37 °C for 4 h. Excess reagents were removed using desalting columns (7000 MWCO, ZebaSpin®, Thermo Scientific) prior to LCMS analysis. Expected masses: 29586 Da. Observed masses (LCMS Method 2): 29586 Da.

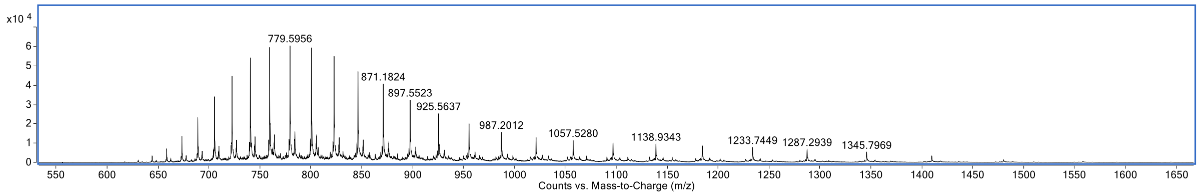

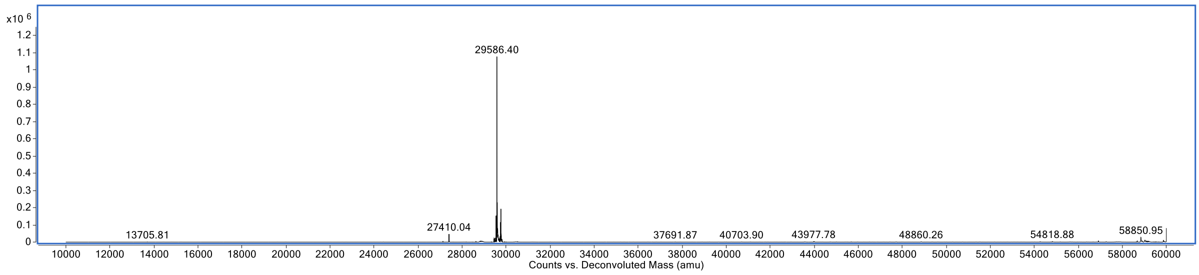

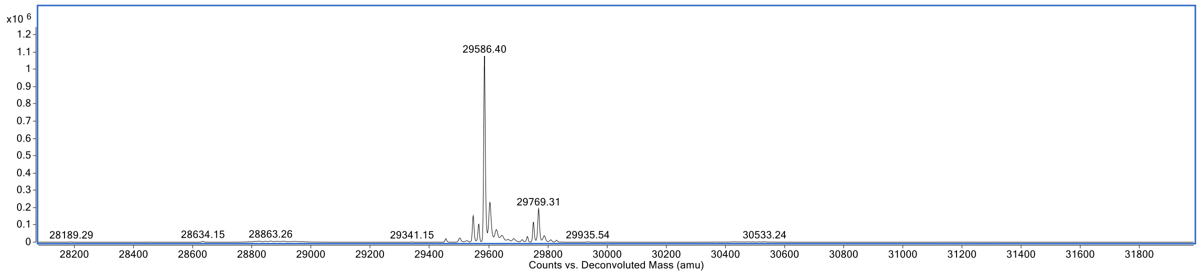

GFPS147C–PD (*N,N’*-diethyl)–Hexane thiol 13

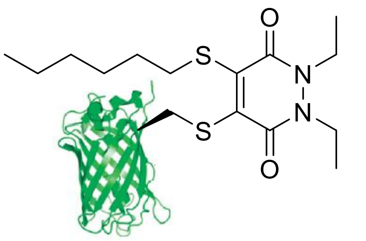

13

To a solution of GFPS147C–PD (N,N’-diethyl)–Br **8** (50 µL, 50 µM in BBS pH 8.0, 5 mM EDTA) was added *n*-hexane thiol **11** (2.5 µL, 20 mM in DMSO, 20 eq., 1 mM final concentration) and the solution was incubated at 4 °C for 16 h. Excess reagents were removed using desalting columns (7000 MWCO, ZebaSpin®, Thermo Scientific) prior to LCMS analysis. Expected masses: 29624 Da. Observed masses (LCMS Method 2): 29624 Da.

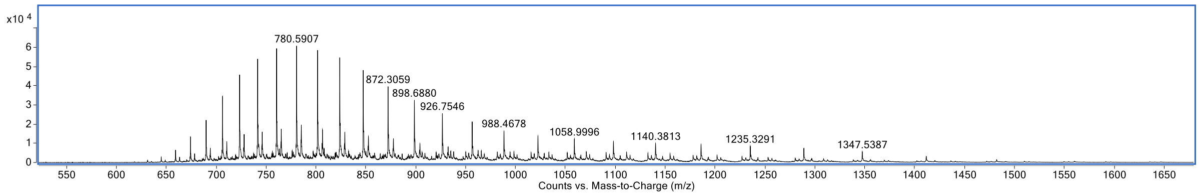

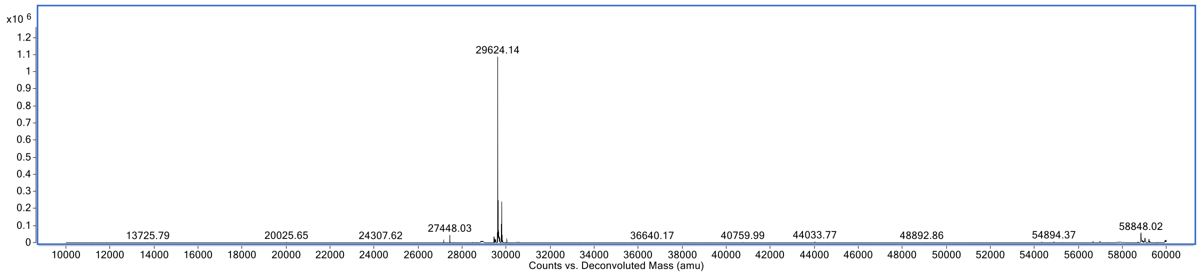

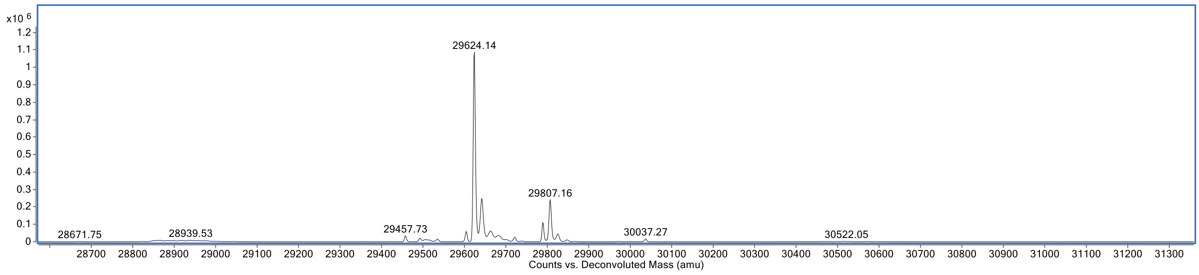

GFPS147C–PD (*N,N'*-diethyl)–FEKGC 14

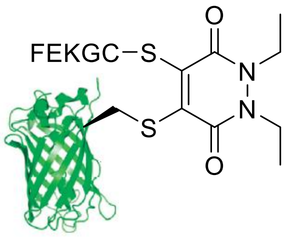

14

To a solution of GFPS147C–PD (N,N'-diethyl)–Br **8** (50 µL, 50 µM in BBS pH 8.0, 5 mM EDTA) was added peptide FEKGC **12** (2.5 µL, 20 mM in DMSO, 20 eq., 1 mM final concentration) and the solution was incubated at 4 °C for 16 h. Excess reagents were removed using desalting columns (7000 MWCO, ZebaSpin®, Thermo Scientific) prior to LCMS analysis. Expected masses: 30088 Da. Observed masses (LCMS Method 2): 30088 Da.

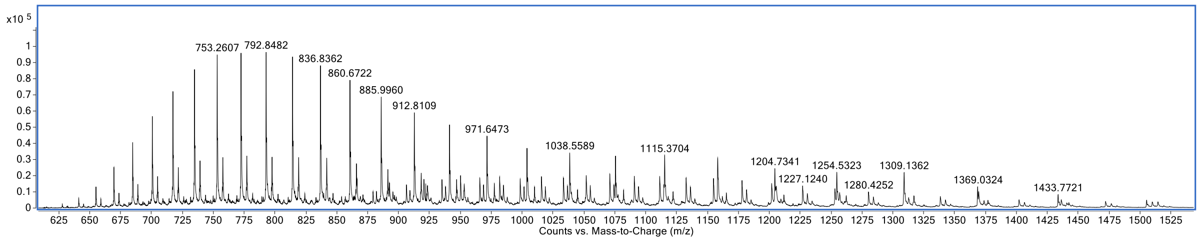

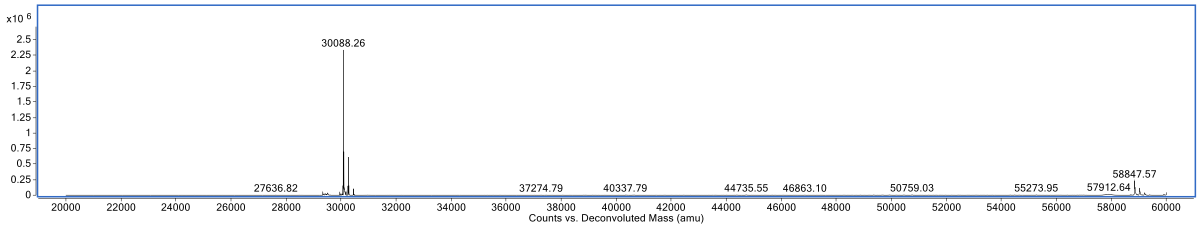

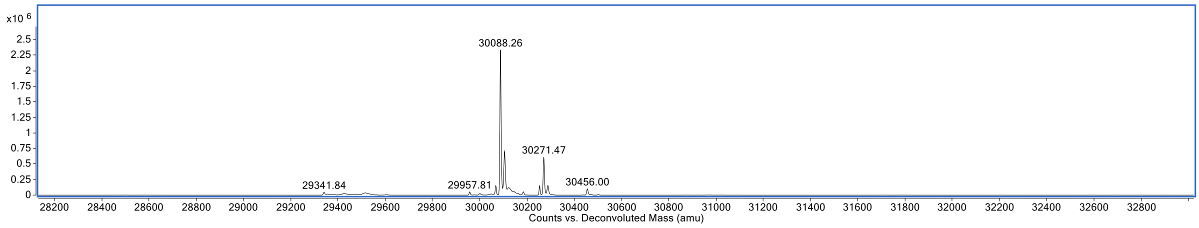

GFPS147C–PD (*N,N'*-diethyl)–*p*-Anisidine 17

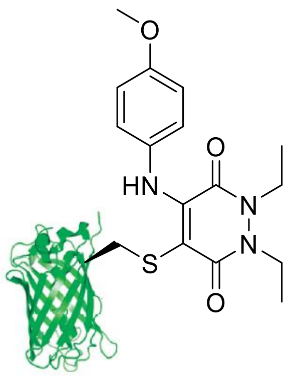

17

To a solution of GFPS147C–PD (N,N'-diethyl)–Br **8** (50 µL, 50 µM in BBS pH 8.0, 5 mM EDTA) was added *p*-anisidine **15** (2.5 µL, 1 M in DMSO, 1000 eq., 50 mM final concentration) and the solution was incubated at 37 °C for 16 h. Excess reagents were removed using desalting columns (7000 MWCO, ZebaSpin®, Thermo Scientific) prior to LCMS analysis. Expected masses: 29629 Da. Observed masses (LCMS Method 2): 29628 Da.

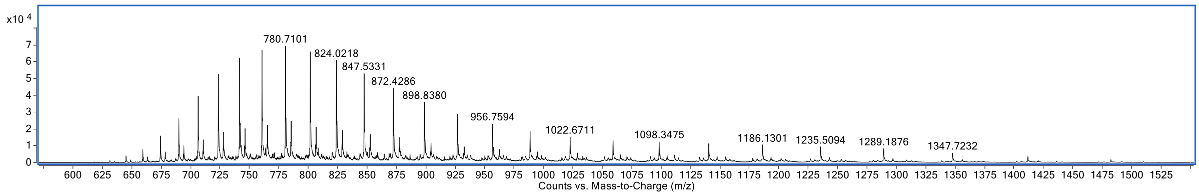

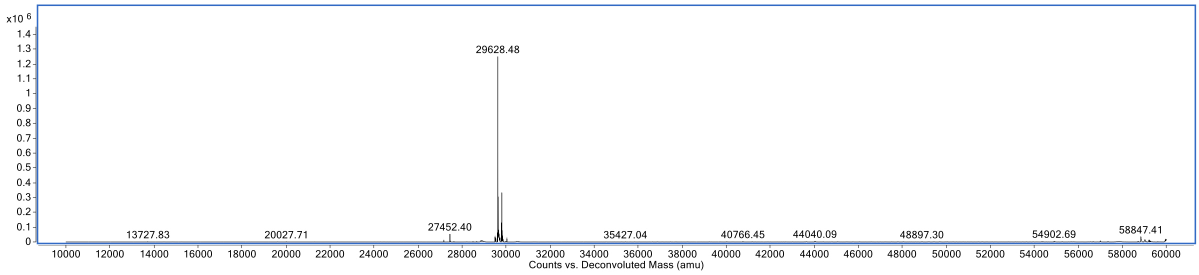

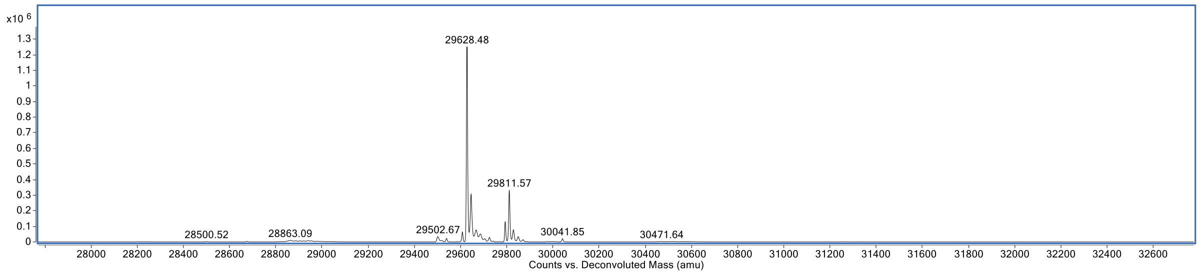

GFPS147C–PD (*N,N’*-diethyl)–Aniline Azide 18

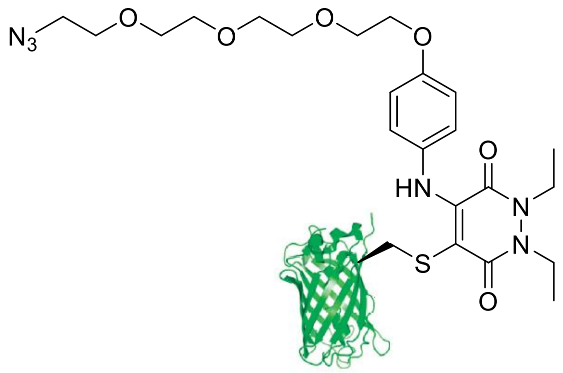

18

To a solution of GFPS147C–PD (N,N’-diethyl)–Br **8** (50 µL, 50 µM in BBS pH 8.0, 5 mM EDTA) was added aniline azide **16** (2.5 µL, 1 M in DMSO, 1000 eq., 50 mM final concentration) and the solution was incubated at 37 °C for 16 h. Excess reagents were removed using desalting columns (7000 MWCO, ZebaSpin®, Thermo Scientific) prior to LCMS analysis. Expected masses: 29816 Da. Observed masses (LCMS Method 2): 29816 Da.

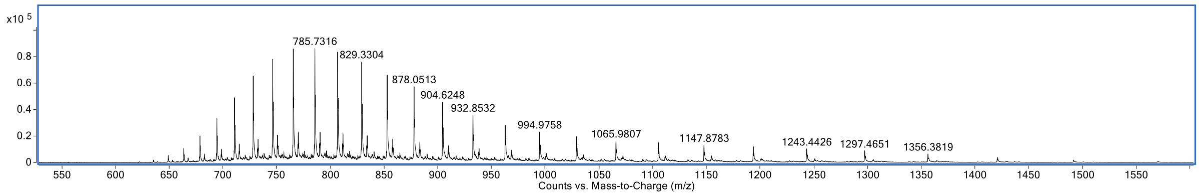

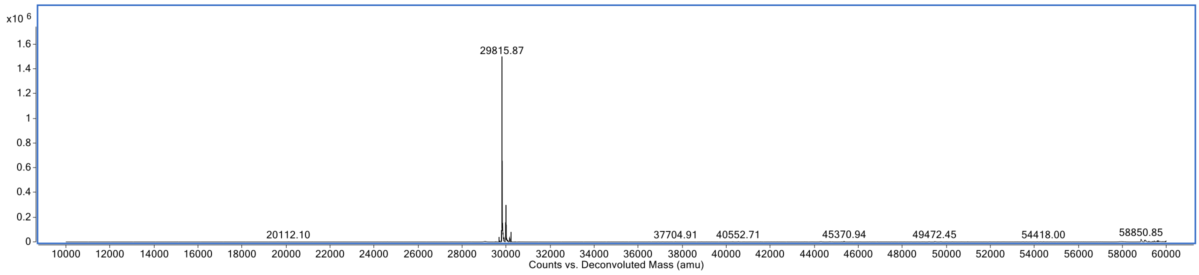

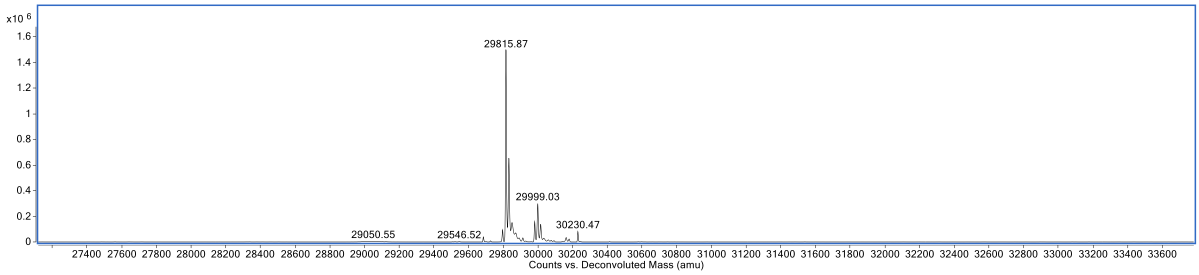

GFPS147C–PD (*N*-methyl, *N'*-BCN (AF-488))–Hexane thiol 20

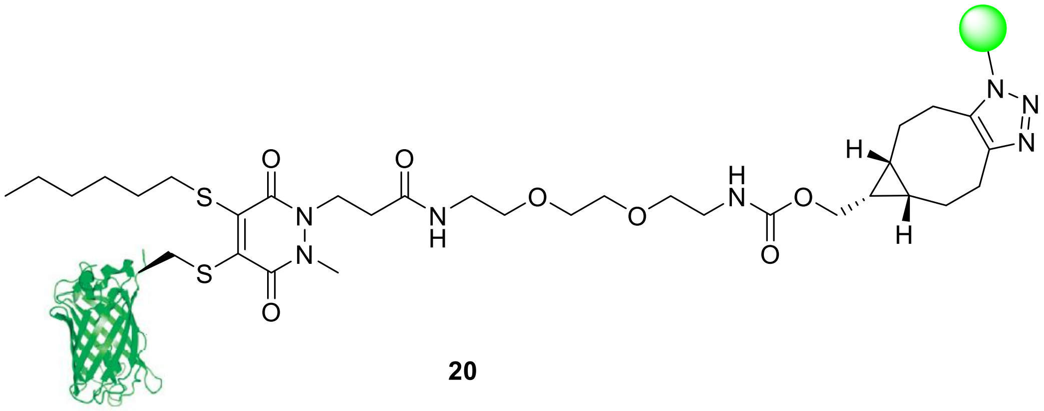

20

To a solution of reduced GFPS147C **1** (250 µL, 50 µM in BBS pH 8.0, 5 mM EDTA), was added *N*-methyl, *N'*-BCN dibromo PD **S6** (12.5 µL, 20 mM in DMSO, 20 eq., 1 mM final concentration) and the solution was incubated at 37 °C for 4 h. After this time excess reagents were removed using desalting columns (7000 MWCO, ZebaSpin®, Thermo Scientific) to elute the conjugate into PBS (pH 7.4, 5 mM EDTA). Alexa fluor-488 azide (6.25 µL, 10 mM in DMSO, 5 eq., 250 µM final concentration) was added and the reaction was incubated for 4 h at 37 °C. Excess reagents were removed using desalting columns (7000 MWCO, ZebaSpin®, Thermo Scientific) to elute the conjugate into BBS (pH 8.0, 5 mM EDTA). To this solution, was added *n*-hexane thiol **11** (12.5 µL, 20 mM in DMSO, 20 eq., 1 mM final concentration) and the reaction was incubated for a further 16 h at 4 °C. Excess reagents and buffer were removed using desalting columns (7000 MWCO, ZebaSpin®, Thermo Scientific) prior to LCMS analysis. Expected masses: 30619 Da, Observed masses (LCMS Method 2): 30621 Da.

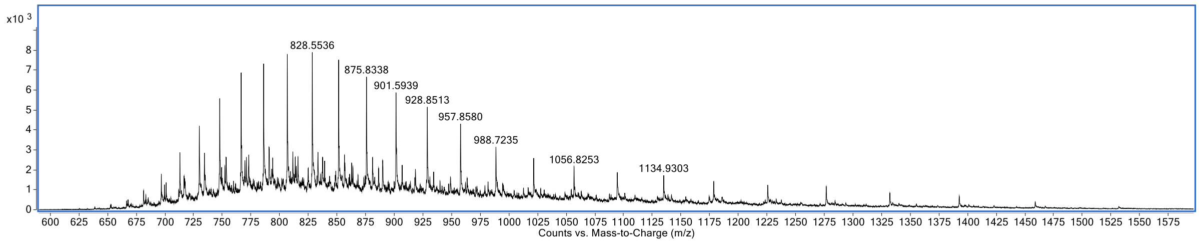
x10 3
828.5536
875.8338
901.5939
928.8513
957.8580
988.7235
1056.8253
1134.9303
Counts vs. Mass-to-Charge (m/z)

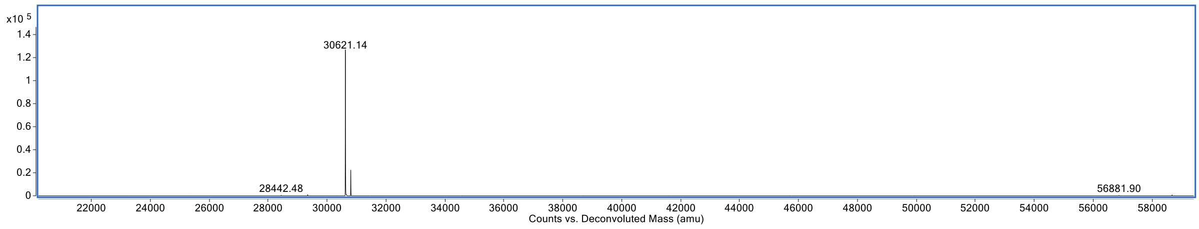
x10 5
30621.14
28442.48
56881.90
Counts vs. Deconvoluted Mass (amu)

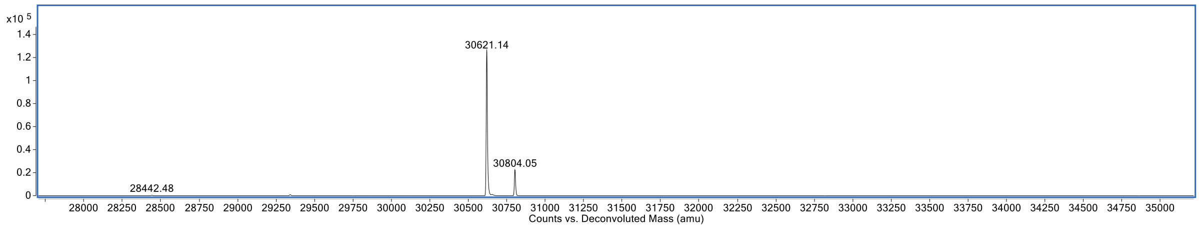
x10 5
30621.14
30804.05
28442.48
Counts vs. Deconvoluted Mass (amu)

GFPS147C–PD (*N*-methyl, *N'*-BCN (AF-488)–*p*-anisidine 21

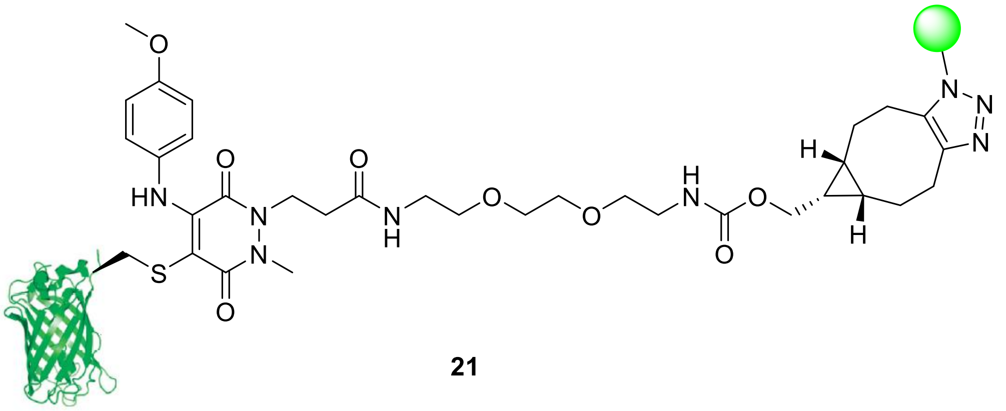

21

To a solution of reduced GFPS147C **1** (250 µL, 50 µM in BBS pH 8.0, 5 mM EDTA), was added *N*-methyl, *N'*-BCN dibromo PD **S6** (12.5 µL, 20 mM in DMSO, 20 eq., 1 mM final concentration) and the solution was incubated at 37 °C for 4 h. After this time excess reagents were removed using desalting columns (7000 MWCO, ZebaSpin®, Thermo Scientific) to elute the conjugate into PBS (pH 7.4, 5 mM EDTA). Alexa fluor-488 azide (6.25 µL, 10 mM in DMSO, 5 eq., 250 µM final concentration) was added and the reaction was incubated for 4 h at 37 °C. Excess reagents were removed using desalting columns (7000 MWCO, ZebaSpin®, Thermo Scientific) to elute the conjugate into BBS (pH 8.0, 5 mM EDTA). To this solution, was added *p*-anisidine **15** (12.5 µL, 1 M in DMSO, 1000 eq., 50 mM final concentration) and the reaction was incubated for a further 16 h at 37 °C. Excess reagents and buffer were removed using desalting columns (7000 MWCO, ZebaSpin®, Thermo Scientific) prior to LCMS analysis. Expected masses: 30624 Da. Observed masses (LCMS Method 2): 30626 Da.

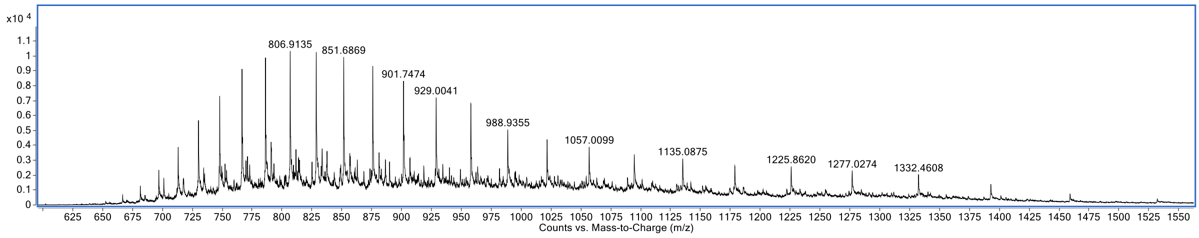
x10 4
806.9135
851.6869
901.7474
929.0041
988.9355
1057.0099
1135.0875
1225.8620
1277.0274
1332.4608
Counts vs. Mass-to-Charge (m/z)

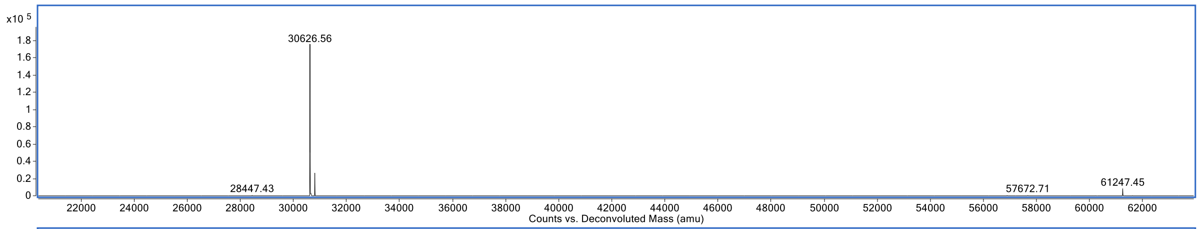
x10 5
30626.56
28447.43
57672.71
61247.45
Counts vs. Deconvoluted Mass (amu)

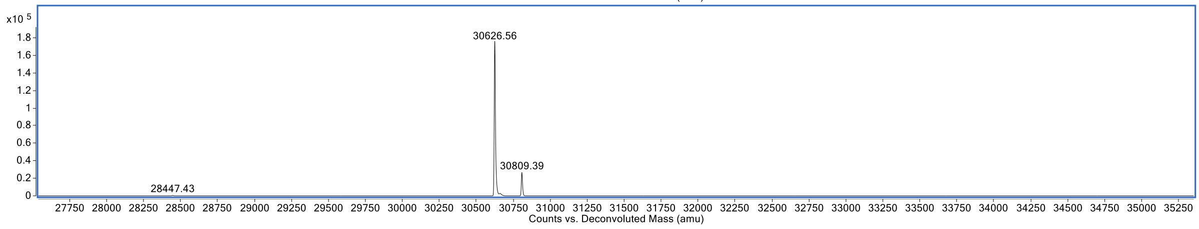
x10 5
30626.56
30809.39
28447.43
Counts vs. Deconvoluted Mass (amu)

GFPS147C–PD (*N*-tetrazine, *N′*-azide)–Br 23

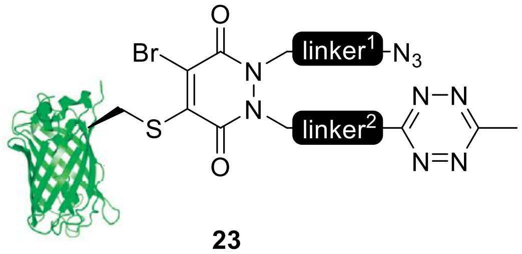

23

To a solution of reduced GFPS147C **1** (500 µL, 50 µM in BBS pH 8.0, 5 mM EDTA) was added *N*-tetrazine, *N′*-azide dibromo pyridazinedione **22** (25 µL, 20 mM in DMSO, 20 eq., 1 mM final concentration) and the solution was incubated at 37 °C for 4 h. Excess reagents were removed using desalting columns (7000 MWCO, ZebaSpin®, Thermo Scientific) prior to LCMS analysis. Expected masses: 30371 Da. Observed masses (LCMS Method 1): 30376 Da.

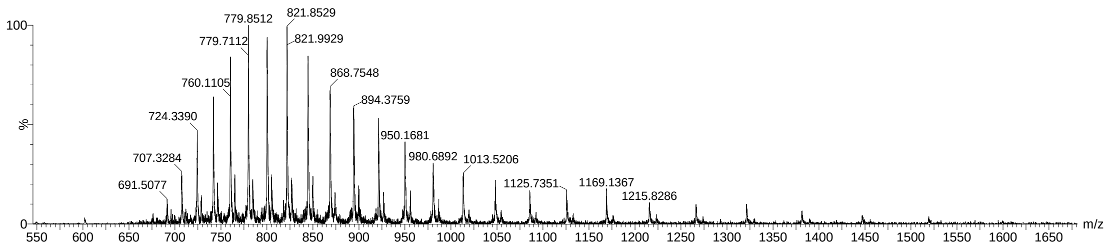

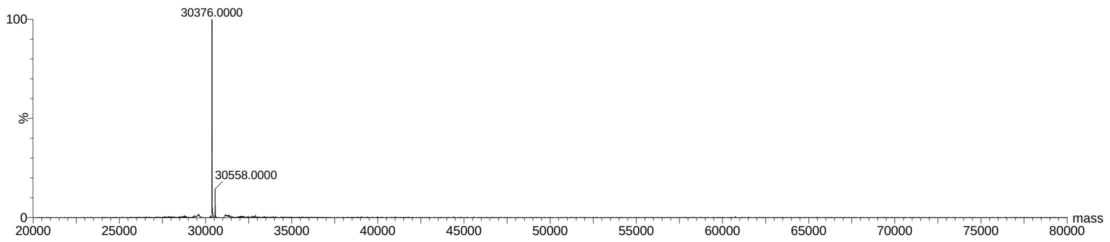

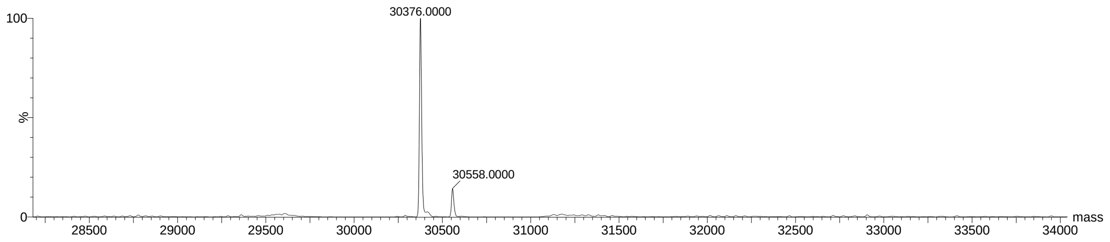

GFPS147C–PD (*N*-tetrazine (BCN fluorescein), *N'*-azide (DBCO biotin))–Br 26

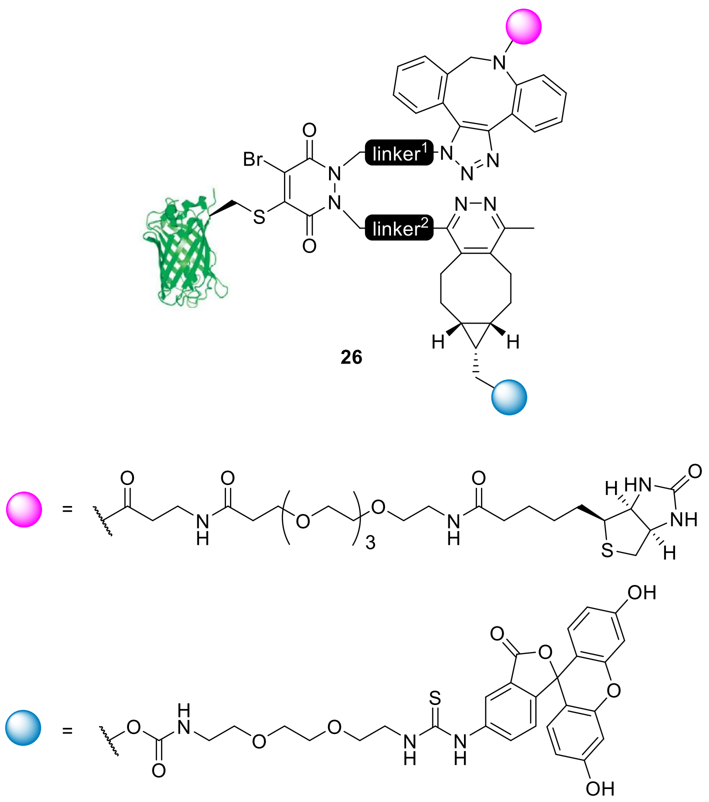

To a solution of GFPS147C–PD (*N*-tetrazine, *N'*-azide)–Br **23** (200 µL, 50 µM in PBS pH 7.4, 5 mM EDTA) were added BCN-Fluorescein **24** (4 µL, 5 mM in DMSO, 2 eq., 100 µM final concentration) and DBCO-biotin **25** (5 µL, 20 mM in DMSO, 10 eq., 500 µM), and the solution was incubated at 37 °C for 4 h. Excess reagents were removed using desalting columns (7000 MWCO, ZebaSpin®, Thermo Scientific) prior to LCMS analysis. Expected masses: 31806 Da. Observed masses (LCMS Method 1): 31806 Da.

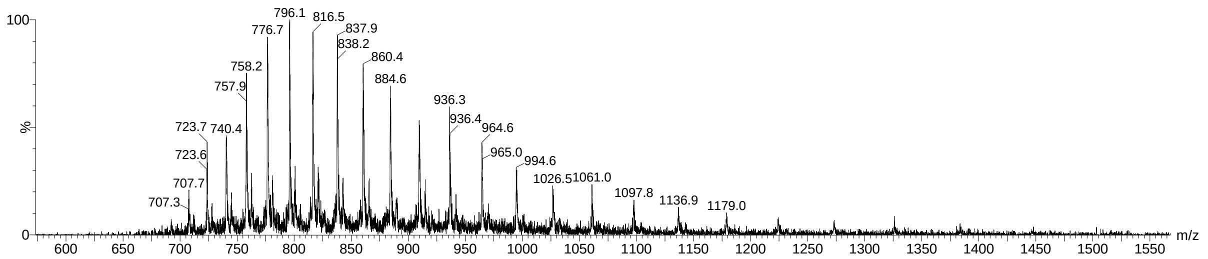
100
%
0
796.1
776.7
816.5
837.9
838.2
860.4
758.2
757.9
884.6
936.3
936.4
964.6
723.7
740.4
723.6
965.0
994.6
707.7
1026.5
1061.0
707.3
1097.8
1136.9
1179.0
m/z
600
650
700
750
800
850
900
950
1000
1050
1100
1150
1200
1250
1300
1350
1400
1450
1500
1550

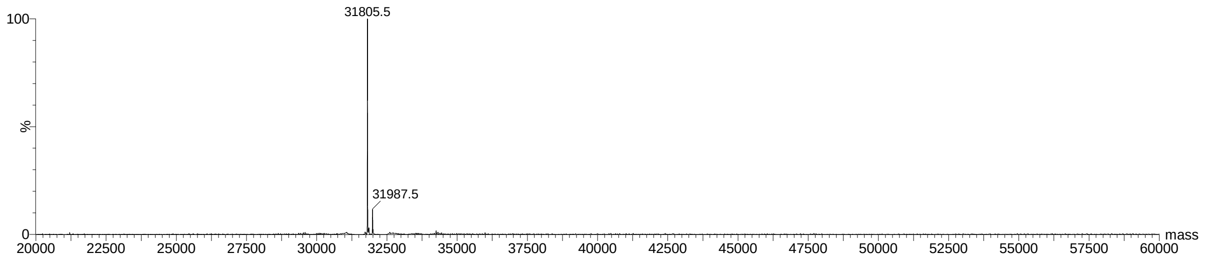
100
%
0
31805.5
31987.5
mass
20000
22500
25000
27500
30000
32500
35000
37500
40000
42500
45000
47500
50000
52500
55000
57500
60000

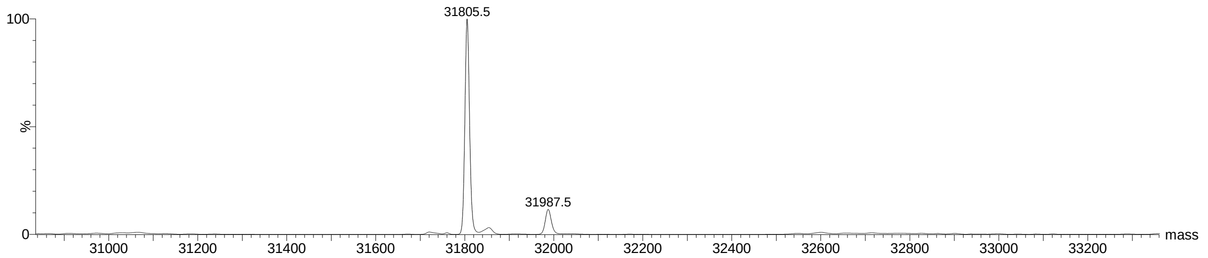
100
%
0
31805.5
31987.5
mass
31000
31200
31400
31600
31800
32000
32200
32400
32600
32800
33000
33200

GFPS147C–PD (*N*-tetrazine(BCN fluorescein), *N'*-azide(DBCO biotin))–Peptide (FEKGC) 27

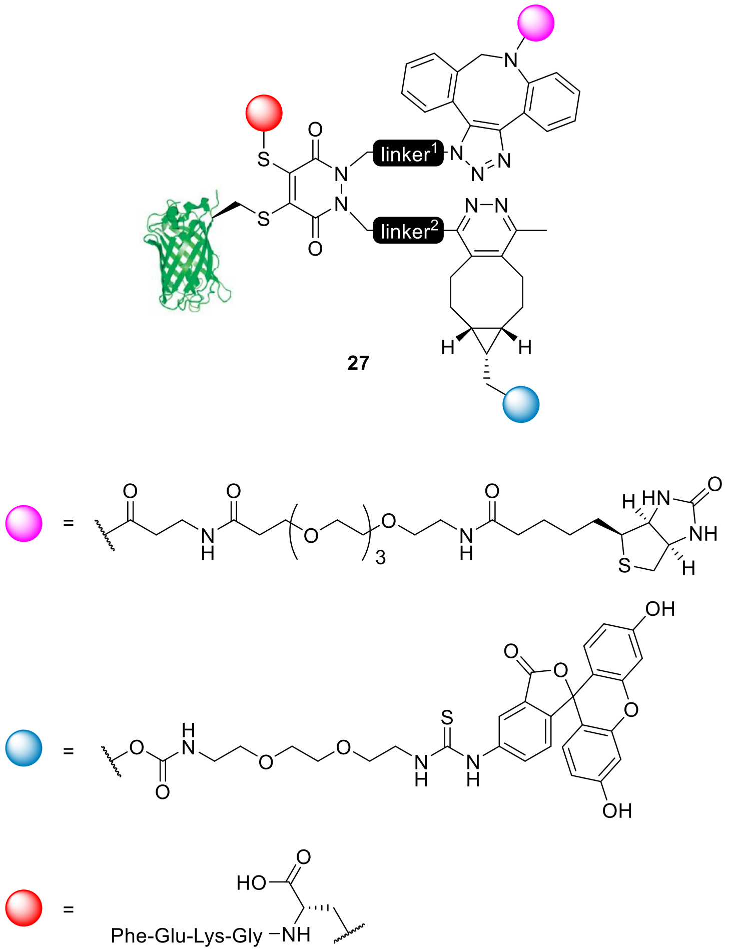

To a solution of GFPS147C–PD (*N*-tetrazine (BCN fluorescein), *N'*-azide (DBCO biotin))–Br **26** (50 µL, 50 µM in BBS pH 8.0, 5 mM EDTA), was added peptide FEKGC **12** (2.5 µL, 20 mM in DMSO, 20 eq., 1 mM final concentration) and the reaction was incubated at 4 °C for 16 h. Excess reagents were removed using desalting columns (7000 MWCO, ZebaSpin®, Thermo Scientific) prior to LCMS analysis. Expected masses: 32309 Da. Observed masses (LCMS Method 1): 32308 Da.

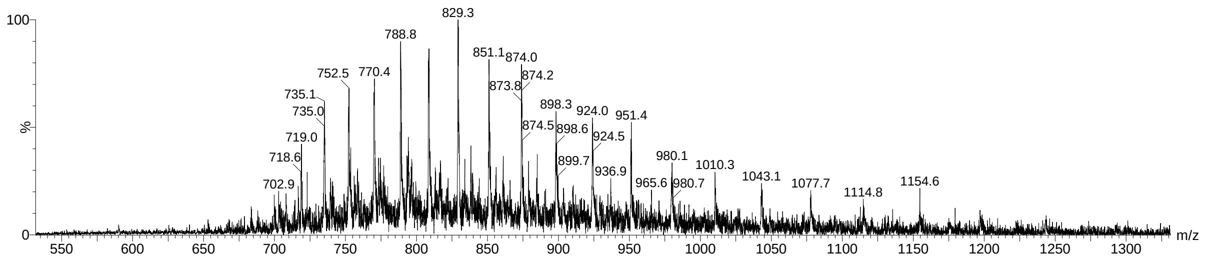

829.3
788.8
851.1
874.0
874.2
873.8
752.5
770.4
735.1
735.0
719.0
718.6
702.9
898.3
898.6
874.5
924.0
924.5
951.4
899.7
936.9
980.1
965.6
980.7
1010.3
1043.1
1077.7
1114.8
1154.6
100
%
0
m/z
550
600
650
700
750
800
850
900
950
1000
1050
1100
1150
1200
1250
1300

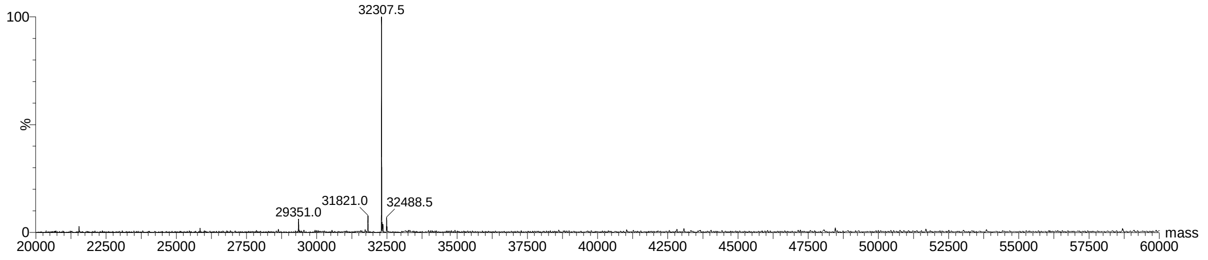

32307.5
31821.0
29351.0
32488.5
100
%
0
mass
20000
22500
25000
27500
30000
32500
35000
37500
40000
42500
45000
47500
50000
52500
55000
57500
60000

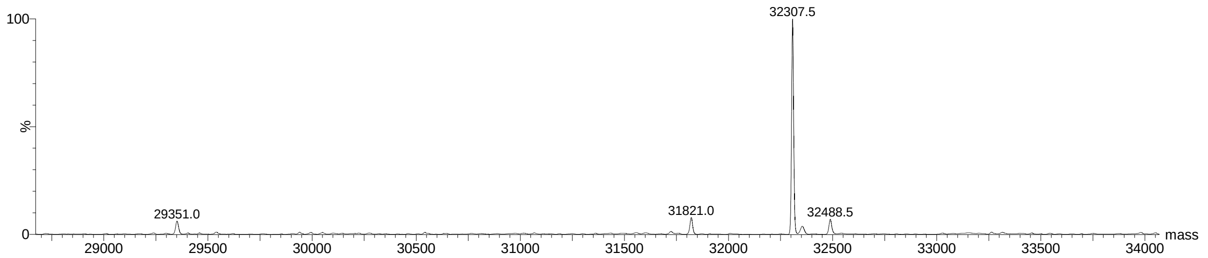

32307.5
29351.0
31821.0
32488.5
100
%
0
mass
29000
29500
30000
30500
31000
31500
32000
32500
33000
33500
34000

GFPS147C–PD (*N*-tetrazine(BCN fluorescein), *N'*-azide(DBCO biotin))–*p*-azido(PEG$_4$)aniline 28

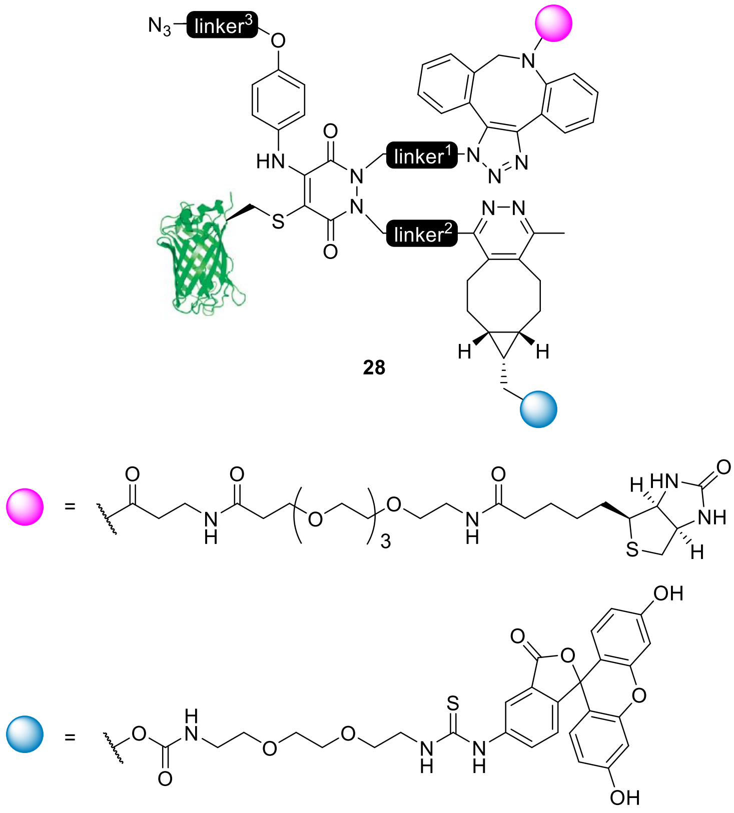

28

To a solution of GFPS147C–PD (*N*-tetrazine (BCN fluorescein), *N'*-azide (DBCO biotin))–Br **26** (50 µL, 50 µM in BBS pH 8.0, 5 mM EDTA) was added aniline azide **16** (2.5 µL, 1 M in DMSO, 1000 eq., final concentration 50 mM) and the reaction was incubated at 37 °C for 16 h. Excess reagents were removed using desalting columns (7000 MWCO, ZebaSpin®, Thermo Scientific) prior to LCMS analysis. Expected masses: 32036 Da. Observed masses (LCMS Method 1): 32035 Da

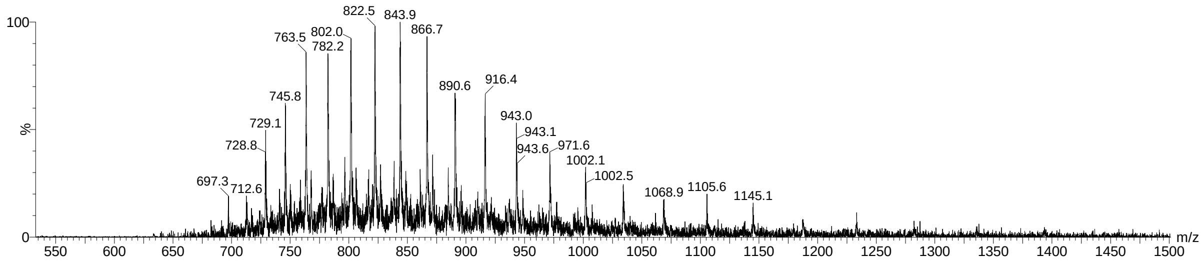
100
%
0
697.3
712.6
728.8
729.1
745.8
763.5
782.2
802.0
822.5
843.9
866.7
890.6
916.4
943.0
943.1
943.6
971.6
1002.1
1002.5
1068.9
1105.6
1145.1
m/z
550 600 650 700 750 800 850 900 950 1000 1050 1100 1150 1200 1250 1300 1350 1400 1450 1500

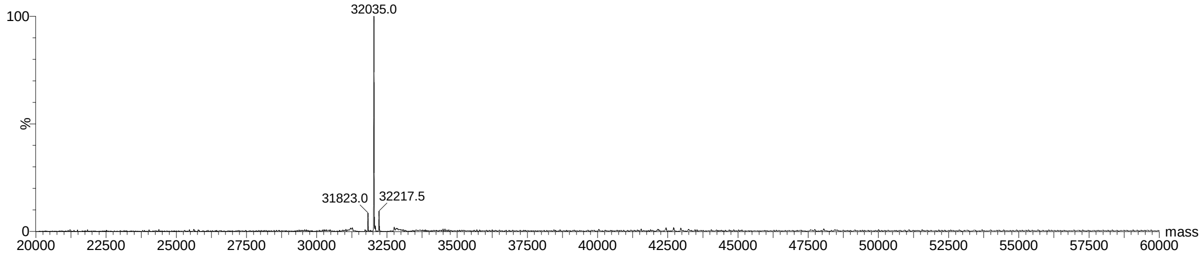
100
%
0
32035.0
31823.0
32217.5
mass
20000 22500 25000 27500 30000 32500 35000 37500 40000 42500 45000 47500 50000 52500 55000 57500 60000

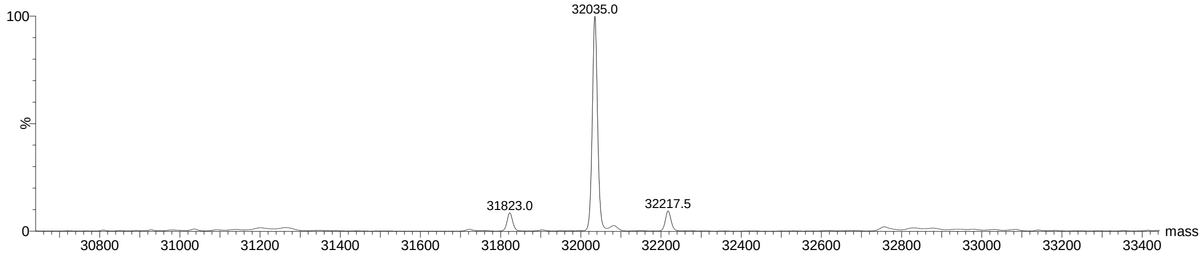
100
%
0
32035.0
31823.0
32217.5
mass
30800 31000 31200 31400 31600 31800 32000 32200 32400 32600 32800 33000 33200 33400

GFPS147C–PD (*N*-tetrazine(BCN fluorescein), *N'*-azide(DBCO biotin))–*p*-PEG$_{12}$(PEG$_4$)aniline 30

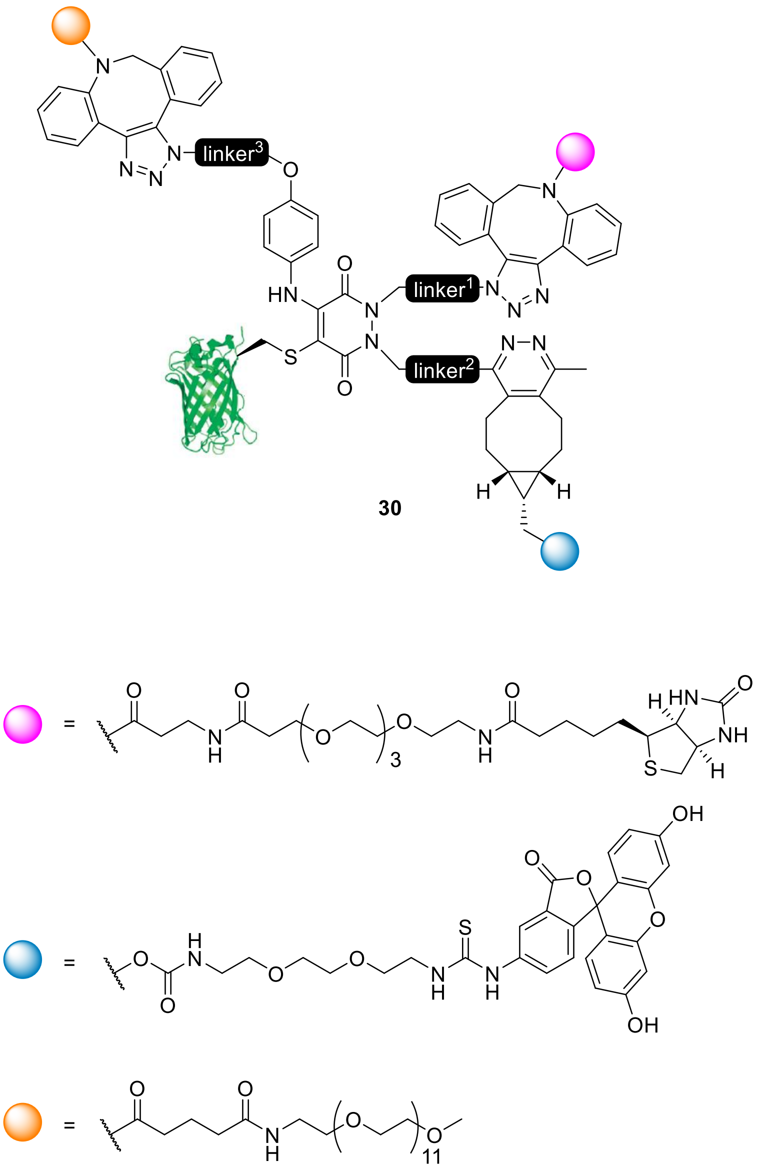

30

To a solution of GFPS147C–PD (N-tetrazine(BCN fluorescein), N'-azide(DBCO biotin))–*p*-azido(PEG$_4$)aniline **28** (50 µL, 50 µM in BBS pH 8.0, 5 mM EDTA) was added DBCO-PEG$_{12}$ **29** (2.5 µL, 20 mM in DMSO, 20 eq., 1 mM final concentration) and the reaction was incubated at 37 °C for 4 h. Excess reagents were removed using desalting columns (7000 MWCO, ZebaSpin®, Thermo Scientific) prior to LCMS analysis. Expected masses: 32897 Da. Observed masses (LCMS Method 1): 32895 Da

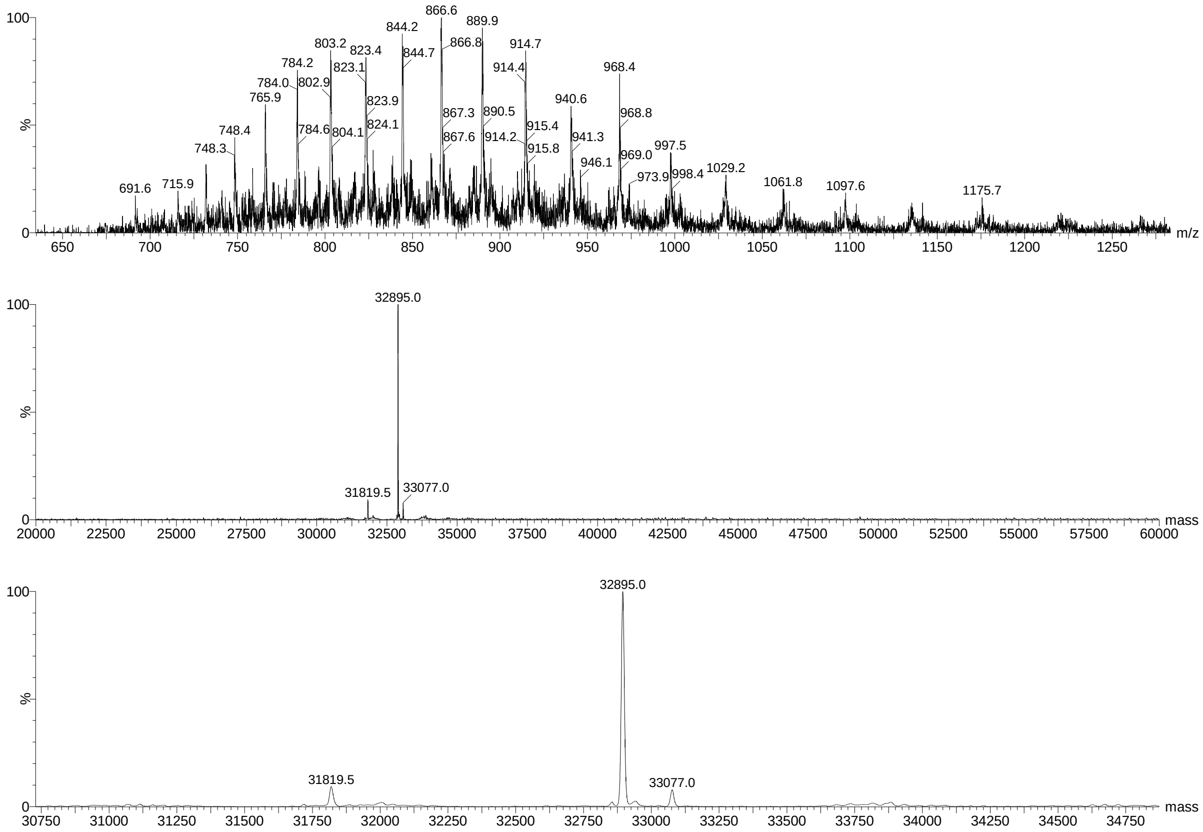
100
%
0
866.6
889.9
844.2
866.8
914.7
803.2
823.4
844.7
784.2
823.1
914.4
968.4
784.0
802.9
765.9
823.9
940.6
867.3
890.5
968.8
748.4
784.6
804.1
824.1
915.4
867.6
914.2
941.3
748.3
915.8
969.0
997.5
946.1
1029.2
973.9
998.4
1061.8
715.9
1097.6
691.6
1175.7
m/z
650
700
750
800
850
900
950
1000
1050
1100
1150
1200
1250
32895.0
31819.5
33077.0
mass
20000
22500
25000
27500
30000
32500
35000
37500
40000
42500
45000
47500
50000
52500
55000
57500
60000
30750
31000
31250
31500
31750
32000
32250
32500
32750
33000
33250
33500
33750
34000
34250
34500
34750

Blood-Like Glutathione (GSH) Cleavage Study

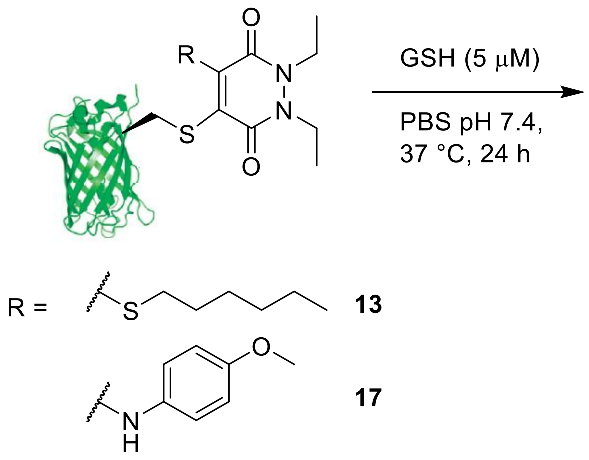

GFPS147C conjugates **13** and **17** (50 µL, 6.8 µM) were buffer exchanged using desalting columns (7000 MWCO, ZebaSpin®, Thermo Scientific) into GSH containing buffer (5 µM GSH, in PBS pH 7.4) and maintained at 37 °C for 24 h. Excess reagents were removed using desalting columns (7000 MWCO, ZebaSpin®, Thermo Scientific) prior to LCMS analysis.

GFPS147C–PD (*N*,*N'*-diethyl)–*n*-Hexane thiol **13**: Expected masses: 29342 Da (reduced GFPS147C **1**), 29624 Da (GFP-PD-*n*-hexane thiol **13**)

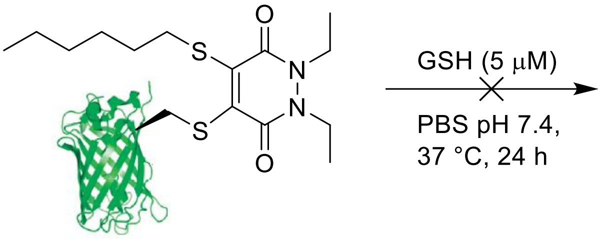

13

0 h: Observed masses (LCMS Method 2): 29624 Da (**13**).

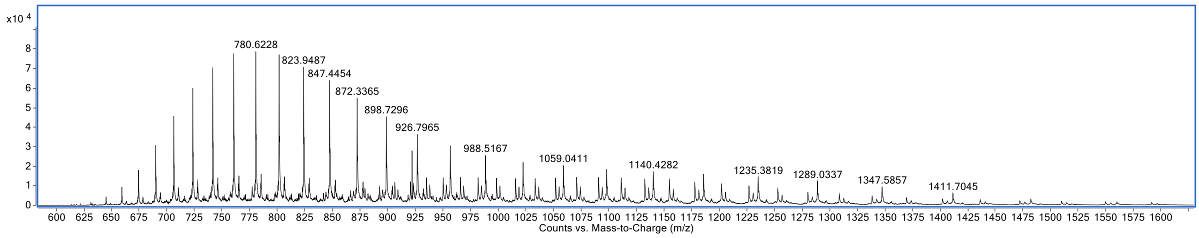

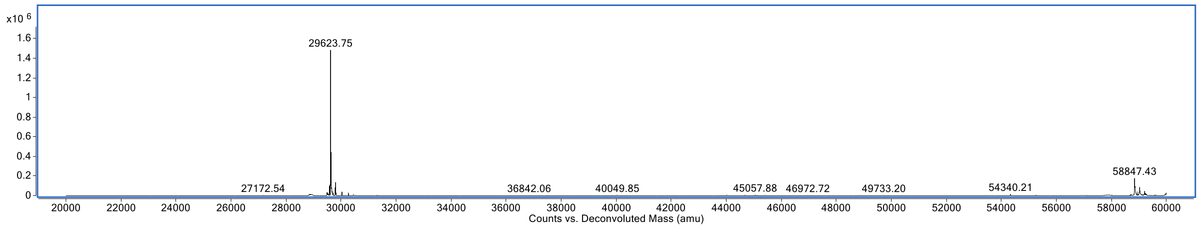

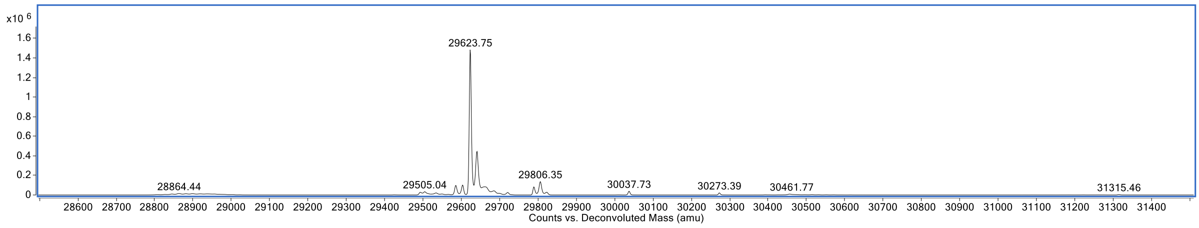

24 h: Observed mass (LCMS Method 2): 29624 Da (**13**)

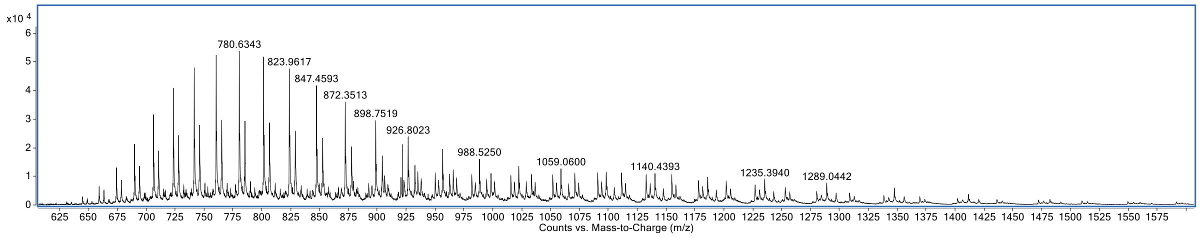

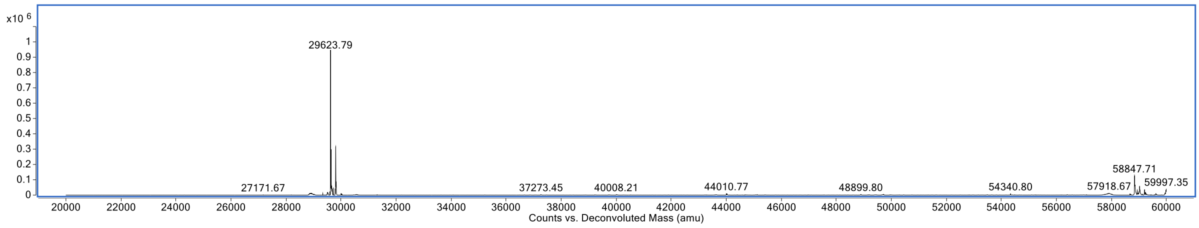

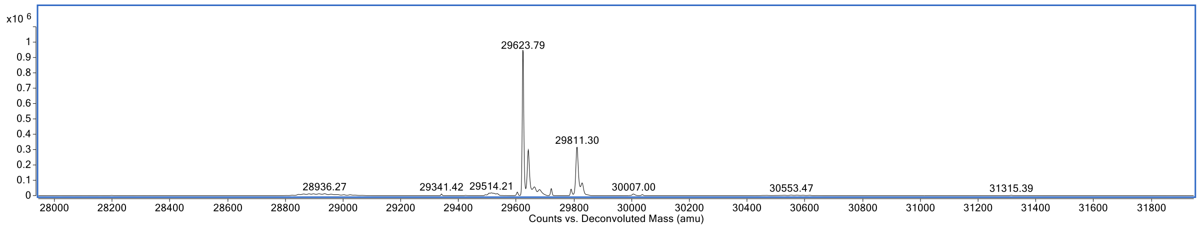

GFPS147C–PD (*N*,*N*′-diethyl)–p-Anisidine **17** – GSH cleavage: Expected masses: 29341 Da (reduced GFPS147C **1**), 29629 Da (GFP-PD-p-Anisidine **17**).

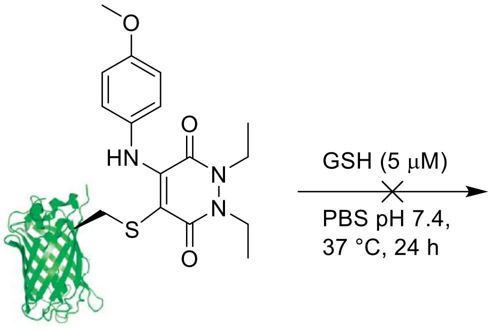

17

0 h: Observed masses (LCMS Method 2): 29629 Da (**17**)

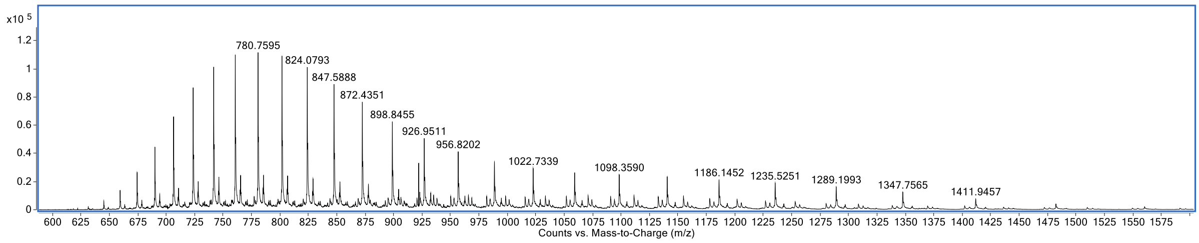

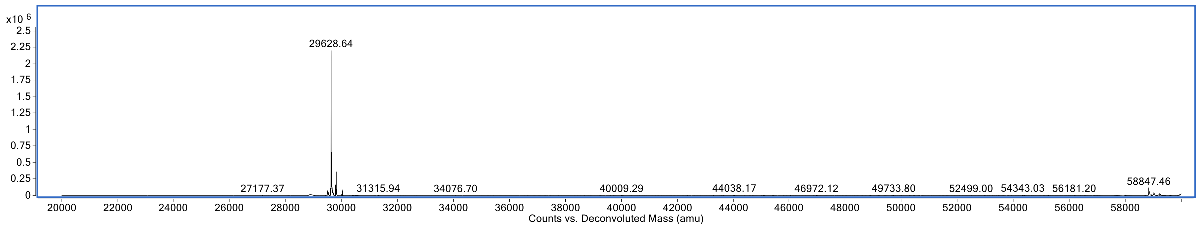

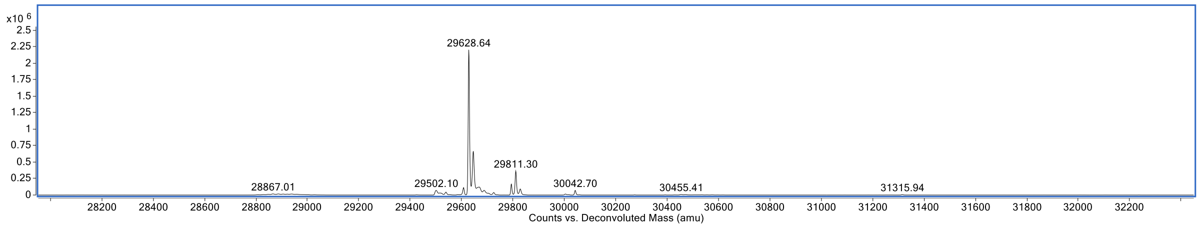

24 h: Observed masses (LCMS Method 2): 29629 Da (**17**)

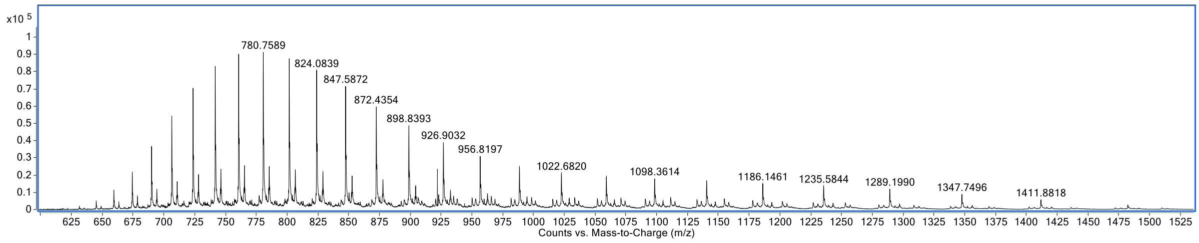

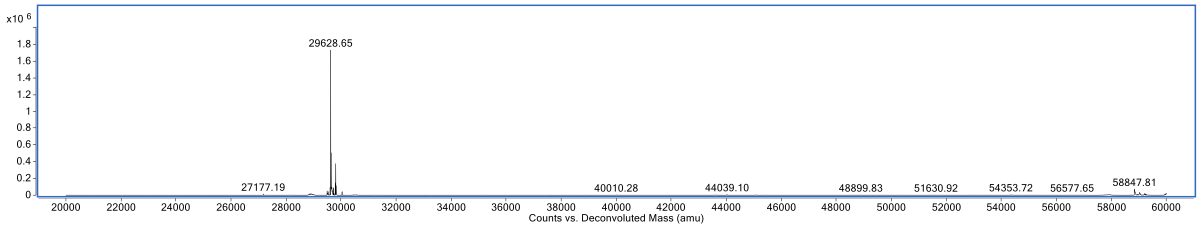

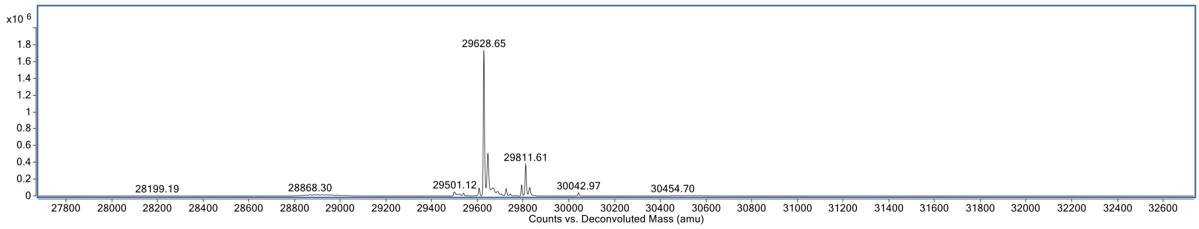

Cell-Like Glutathione (GSH) Cleavage Study

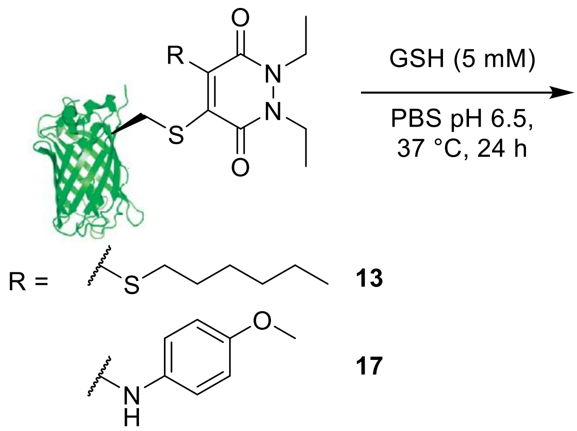

GFPS147C conjugates **13** and **17** (50 µL, 6.8 µM) were buffer exchanged using desalting columns (7000 MWCO, ZebaSpin®, Thermo Scientific) into GSH containing buffer (5 mM GSH, in PBS pH 6.5) and maintained at 37 °C for 24 h. Samples were taken at 2, 4, 8 and 24 h timepoints where excess reagents were removed using desalting columns (7000 MWCO, ZebaSpin®, Thermo Scientific) prior to LCMS analysis.

GFPS147C–PD (*N*,*N*'-diethyl)–*n*-Hexane thiol **13**: Expected masses: 29342 Da (reduced GFPS147C **1**), 29624 Da (GFP-PD-*n*-hexane thiol **13**), 29646 (GFP-GSH heterodimer **19**)

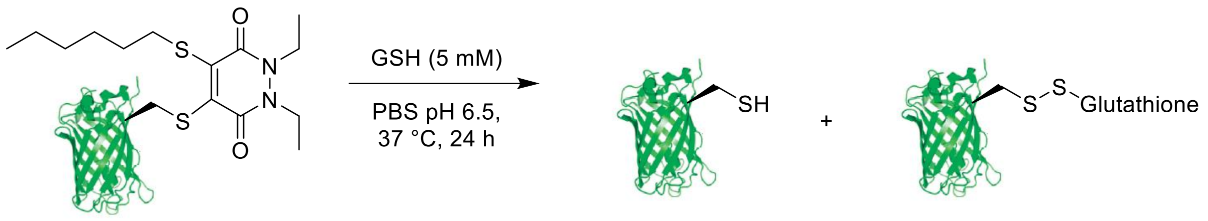

13 **1** **19**

0 h: Expected masses: 29624 Da. Observed masses (LCMS Method 2): 29624 Da (**13**)

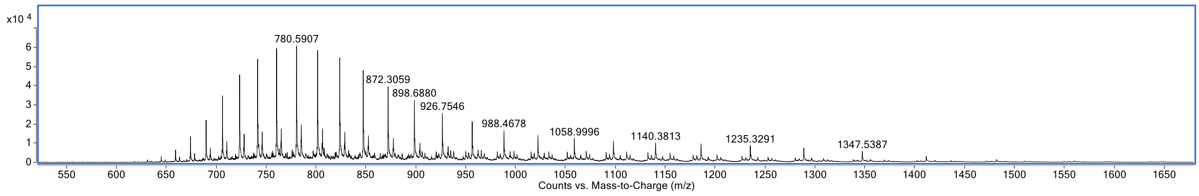

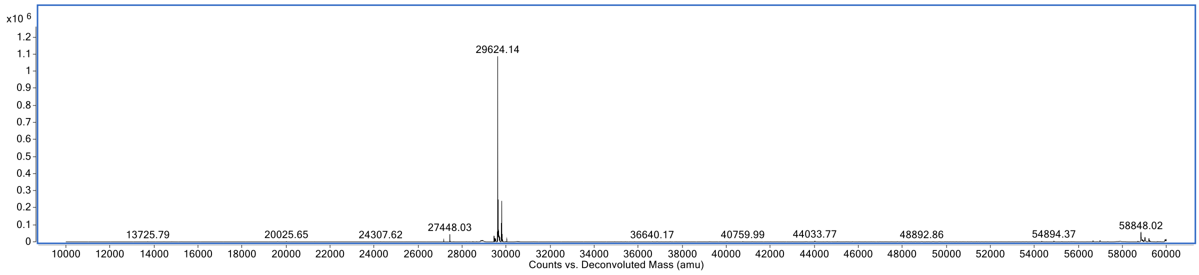

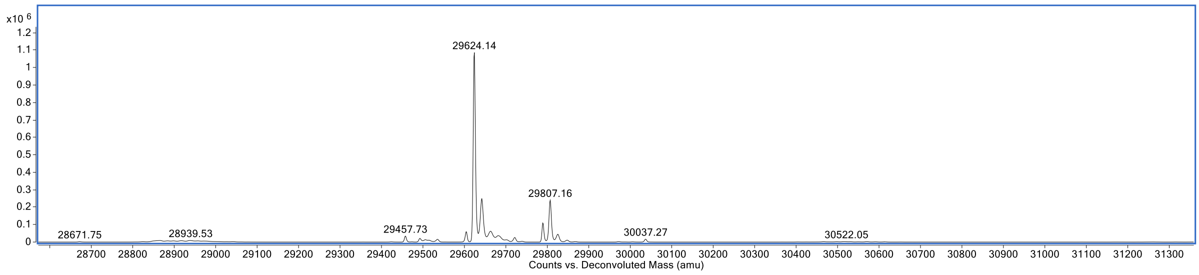

2 h: Observed masses (LCMS Method 2): 29341 Da (**1**), 29646 Da (**19**).

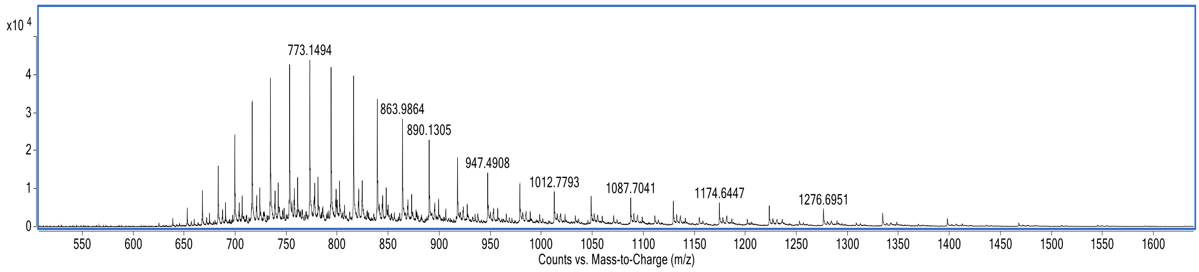

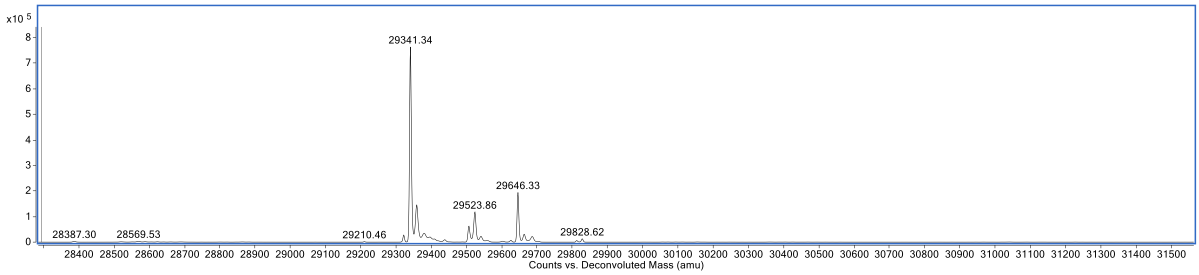

4 h: Observed masses (LCMS Method 2): 29341 Da (**1**), 29646 Da (**19**).

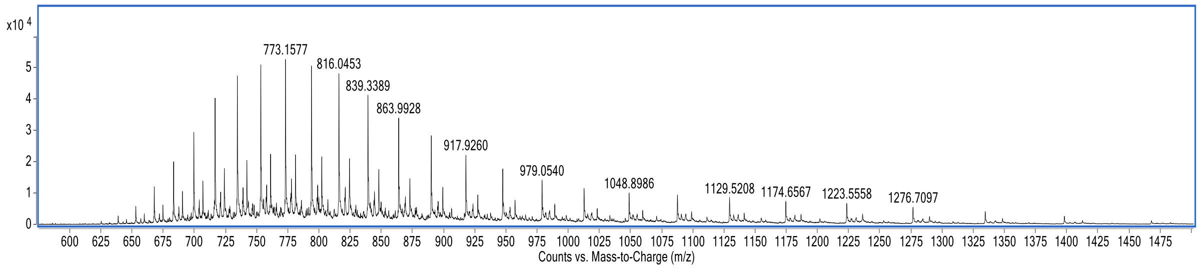

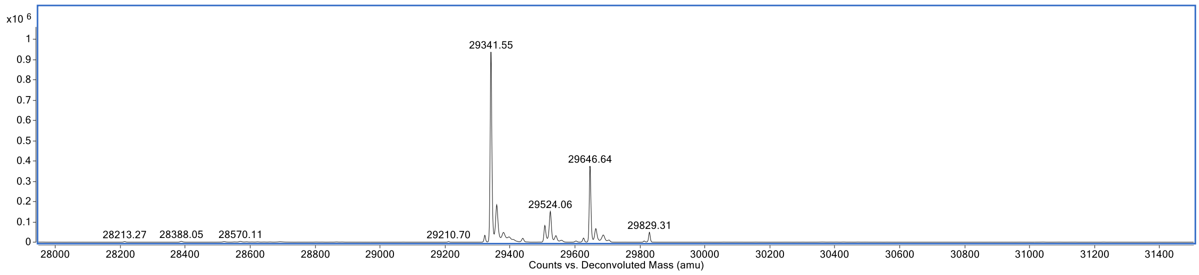

8 h: Observed masses (LCMS Method 2): 29341 Da (**1**), 29646 Da (**19**).

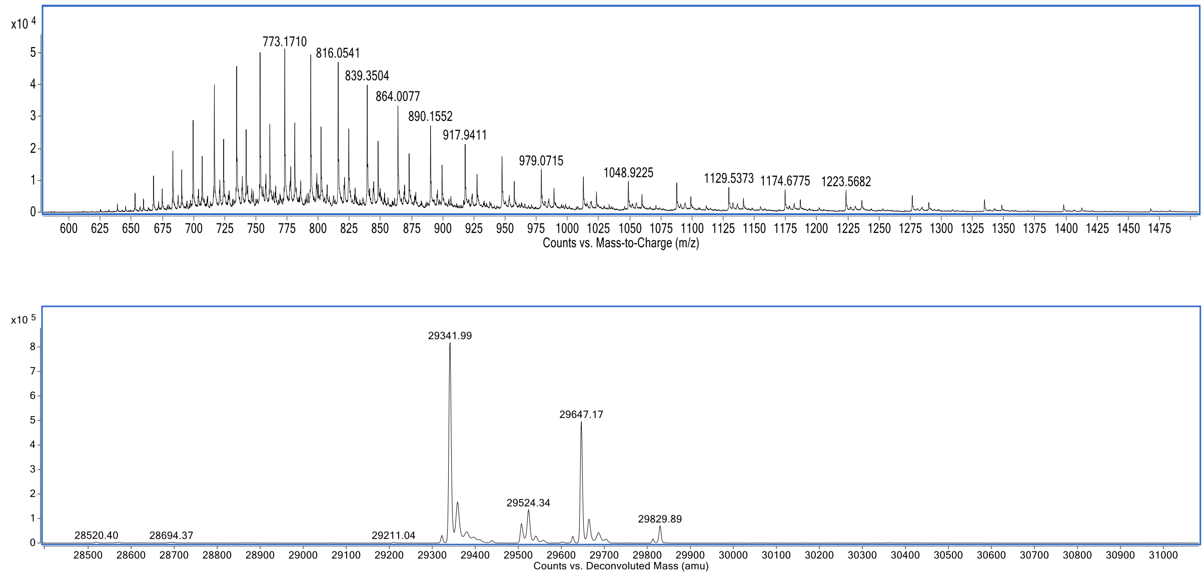

24 h: Observed masses (LCMS Method 2): 29646 Da (**19**).

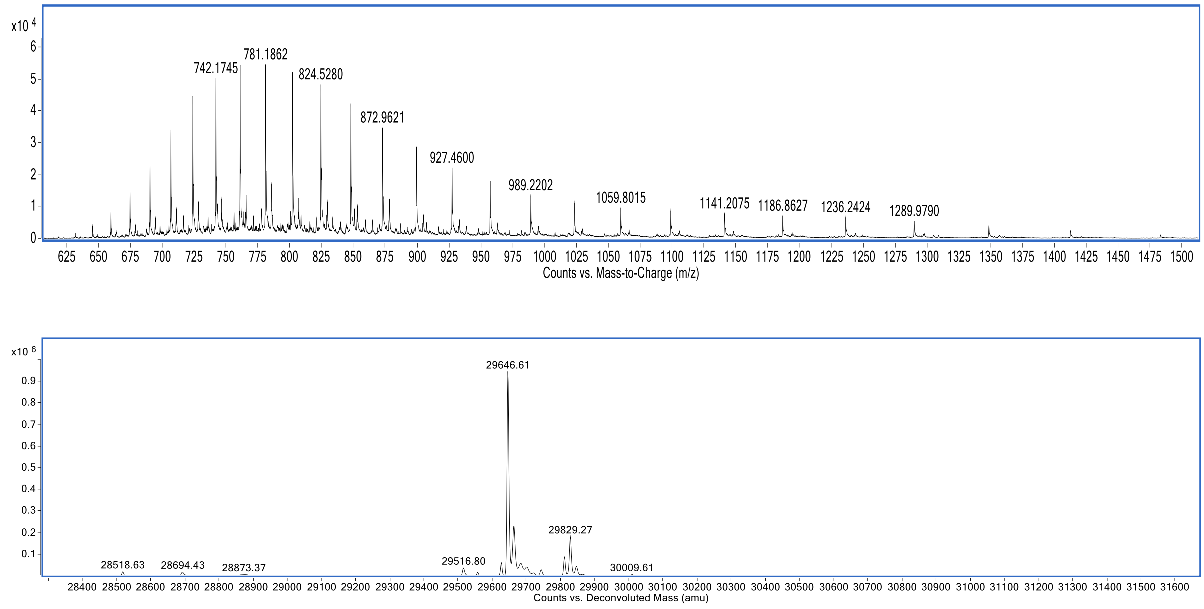

GFPS147C–PD (*N*,*N*'-diethyl)–p-Anisidine **17** – GSH cleavage: Expected masses: 29629 Da (GFP-PD-p-Anisidine **17**)

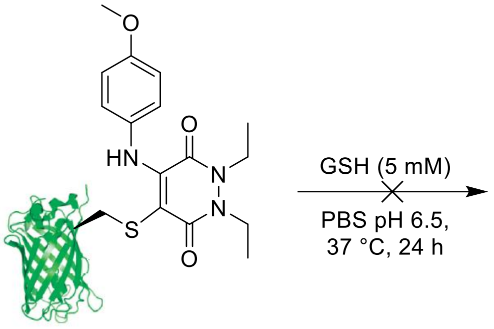

17

0 h: Expected masses: 29629 Da. Observed masses (LCMS Method 2): 29628 Da (**17**).

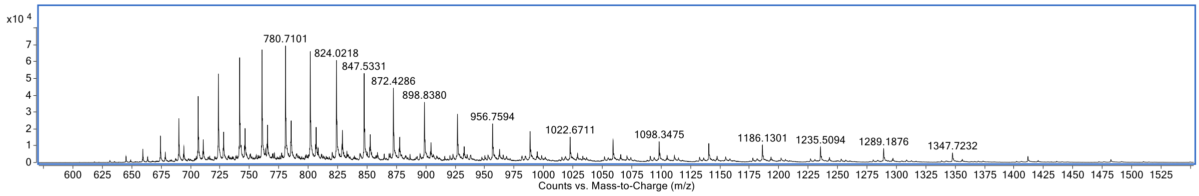

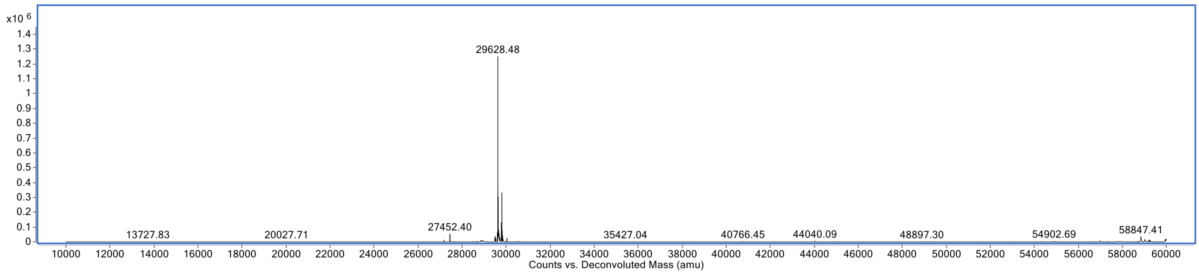

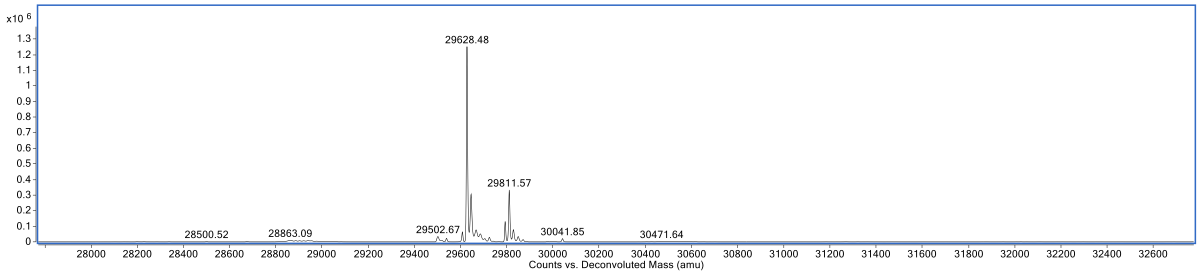

2 h: Observed masses (LCMS Method 2): 29628 Da (**17**), 29645 (**17** + [O])

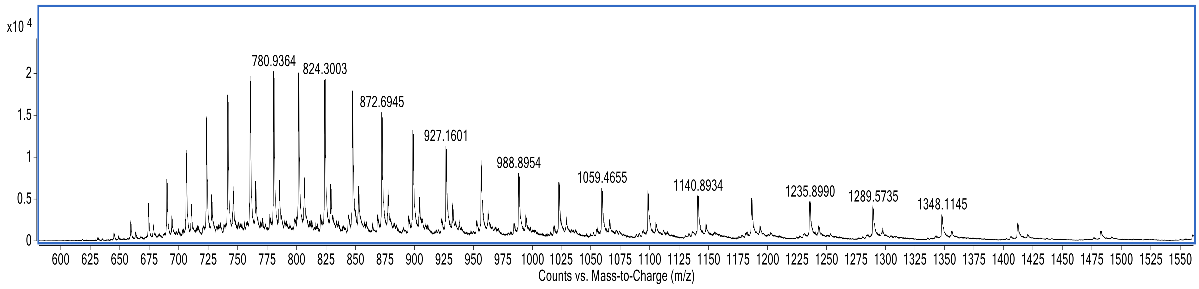

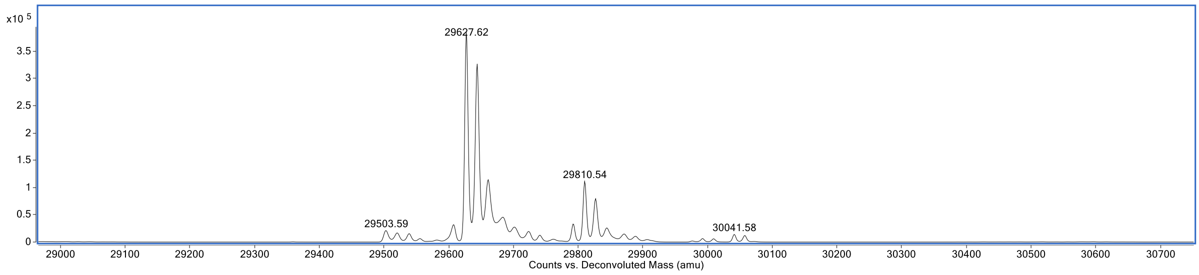

4 h: Observed masses (LCMS Method 2): 29628 Da (**17**), 29645 (**17** + [O])

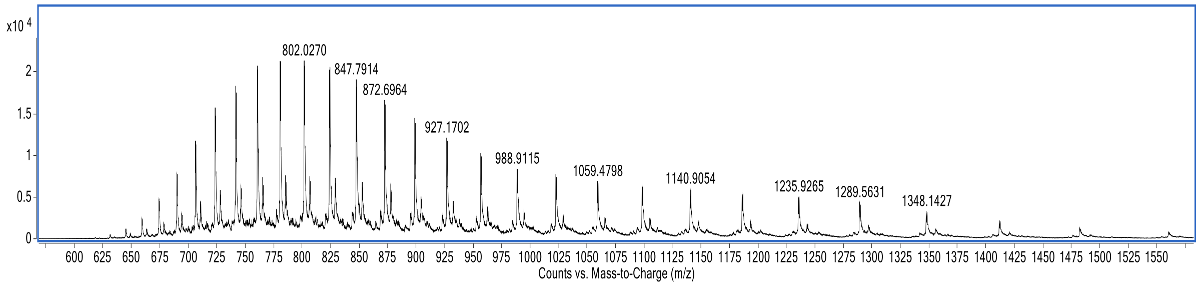

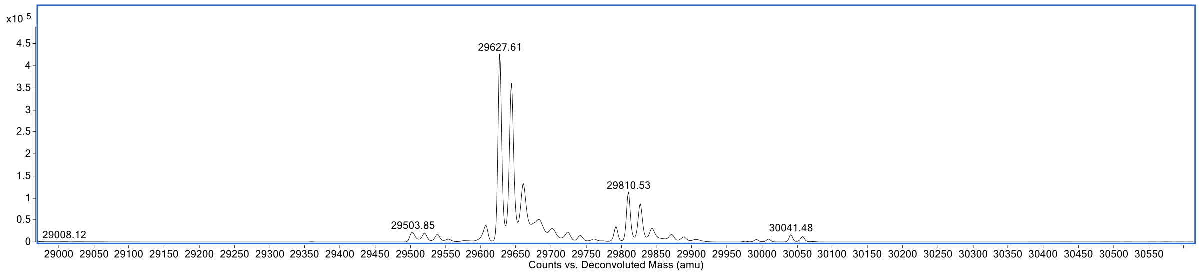

8 h: Observed masses (LCMS Method 2): 29628 Da (**17**), 29645 (**17** + [O])

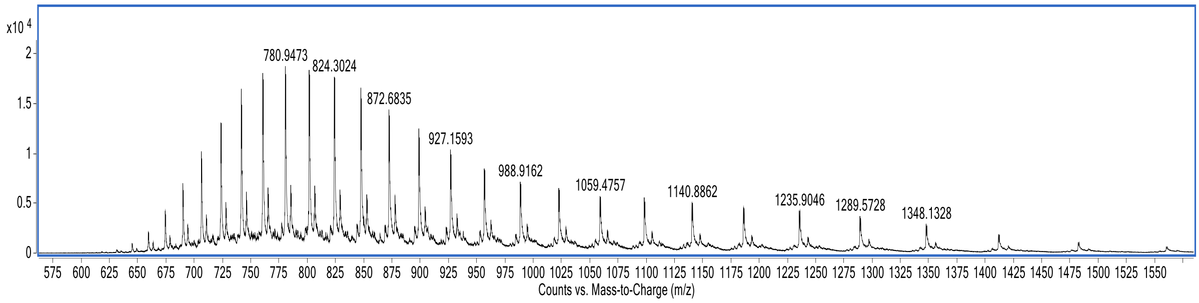

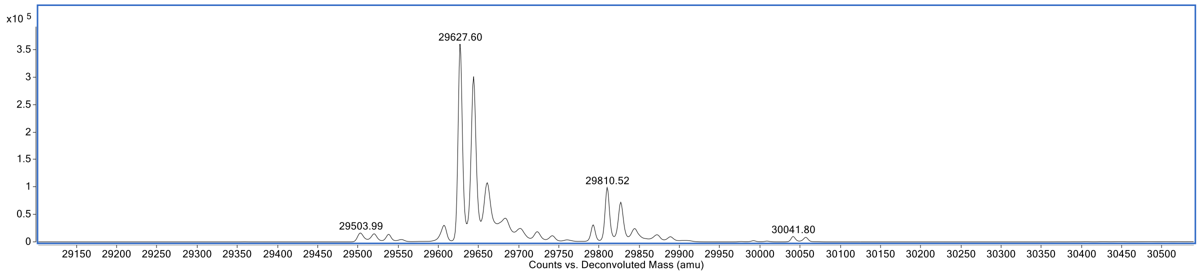

24 h: Observed masses (LCMS Method 2): 29628 Da (**17**), 29645 (**17** + [O])

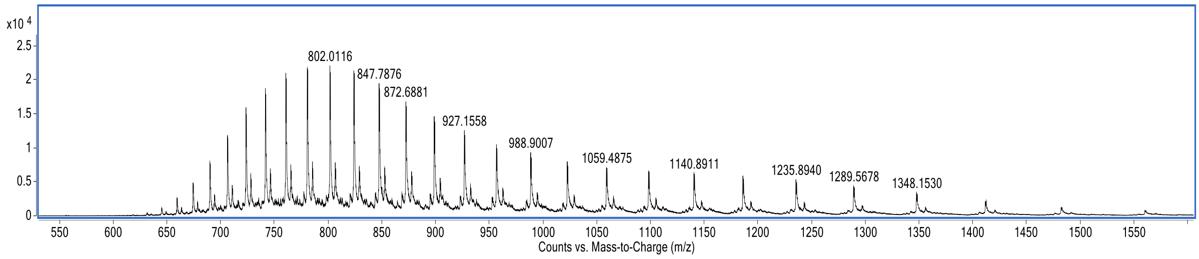

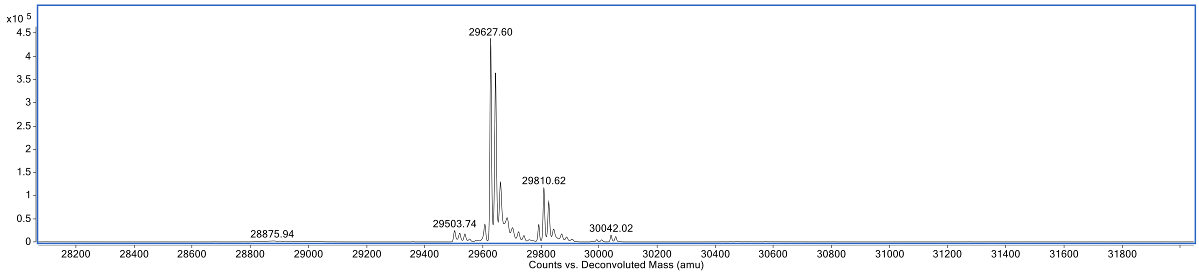

Serum Stability Study[12]

GFPS147C–PD (*N*-methyl, *N'*-BCN (AF-488)–conjugates **20** and **21** were prepared as 0.2 mg/mL solutions in PBS 140 mM sodium chloride 12 mM sodium phosphates and 2 mM sodium azide at pH 7.4. The conjugates were diluted with 50 % of human blood serum to give a final a concentration of 0.1 mg/mL of 5 or 12 and 1 mM of sodium azide. One aliquot (50 µL) for each conjugate was taken, flash frozen and stored at −20 °C. The remaining solution was incubated at 37 °C under mild shaking (300 rpm) and covered from light. Aliquots (50 µL) were taken at 1, 3, 5 and 7 days, flash frozen and stored at −20 °C. Aliquots were thawed, spin-filtered (0.22 µm filter) and diluted 100× with elution buffer. Samples (20 µL) of diluted aliquots were analysed by SEC-HPLC on a TSK gel G3000SWXL (7.8 mm x 30 cm) column connected to an Agilent 1200 HPLC system equipped with a 1200 series diode array detector and a fluorescence detector. Samples were eluted using PBS 140 mM NaCl, 100 mM sodium phosphates and 0.02 % sodium azide at pH 7.0 as mobile phase at a flow rate of 0.5 mL/min. over 30 min. Fluorescence was detected with an excitation wavelength of 495 nm and emission wavelength of 525 nm.

GFPS147C–PD (*N*-methyl, *N'*-BCN (AF-488)–*n*-Hexane thiol **20**:

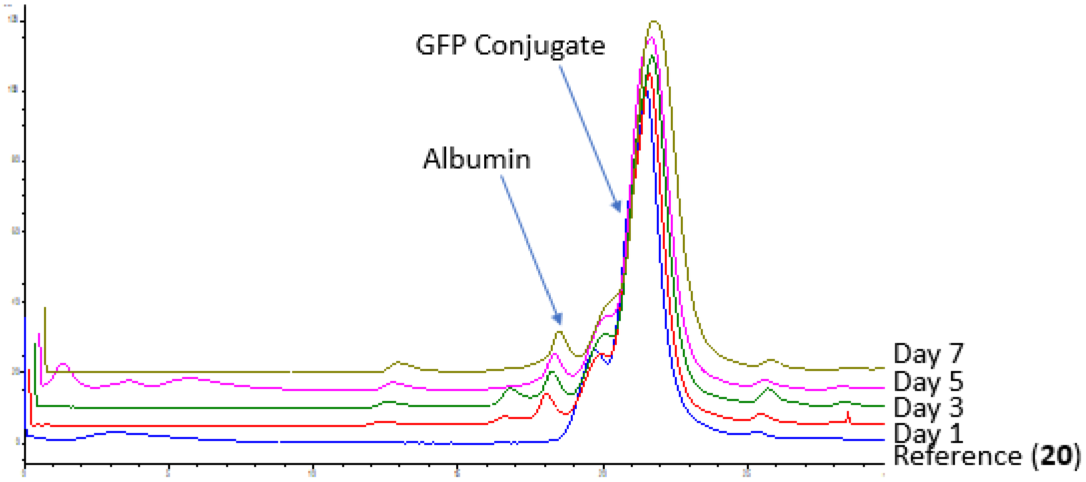

Note: Reference for sample **20** (confirmed to be a single species by LCMS) is observed as two peaks by native SEC-HPLC

Overlay with HSA reference (normalised):

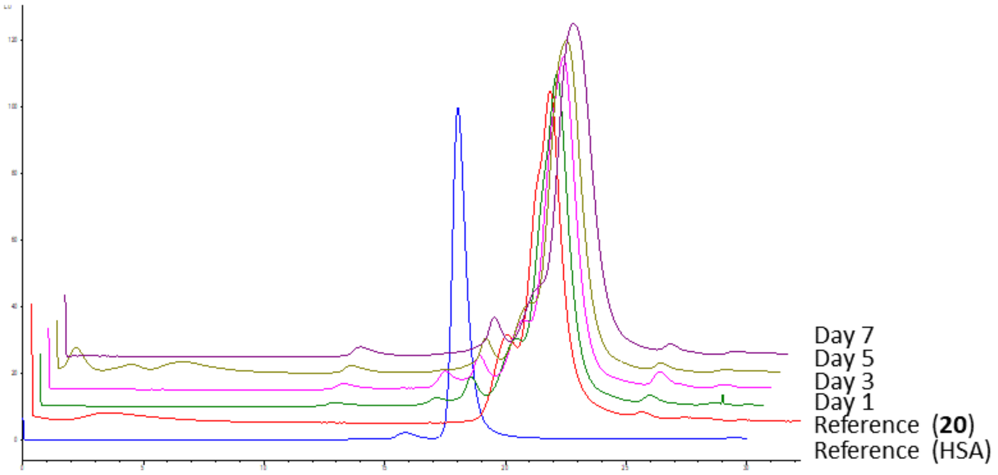

Overlay with HSA reference (uncorrected):

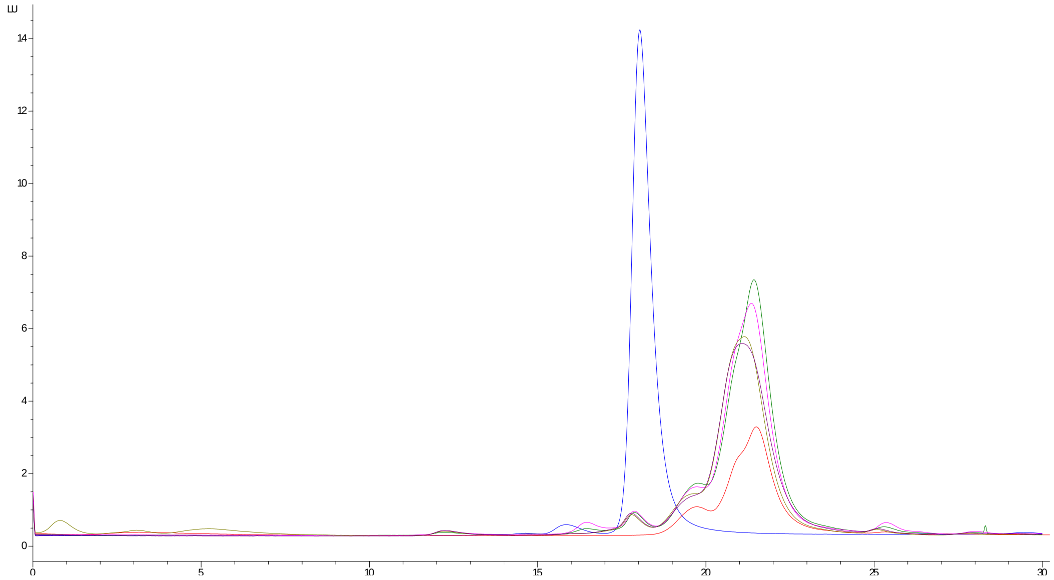

GFPS147C–PD (*N*-methyl, *N*'-BCN (AF-488)–*p*-Anisidine **21**:

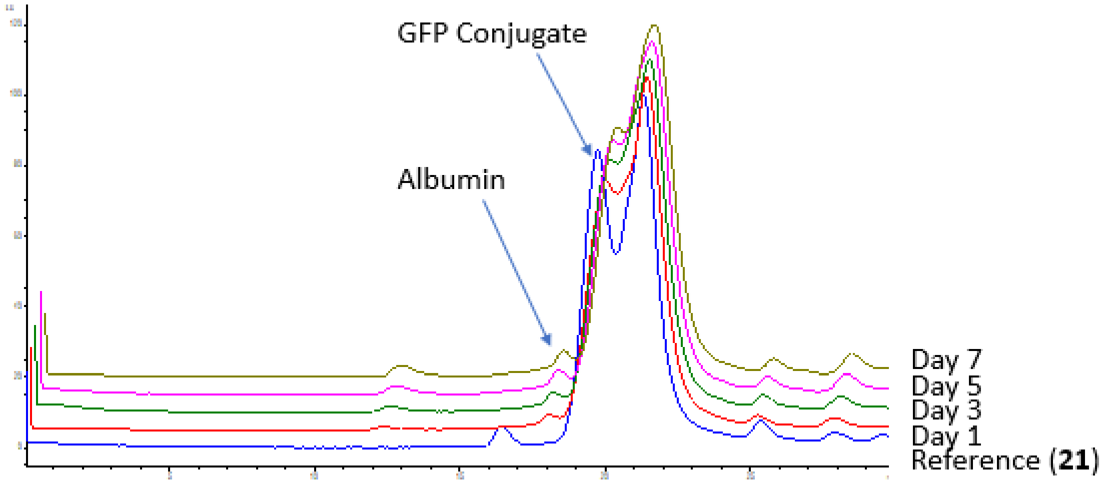

Note: Reference for sample **21** (confirmed to be a single species by LCMS) is observed as two peaks by native SEC-HPLC

Overlay with HSA reference (normalised):

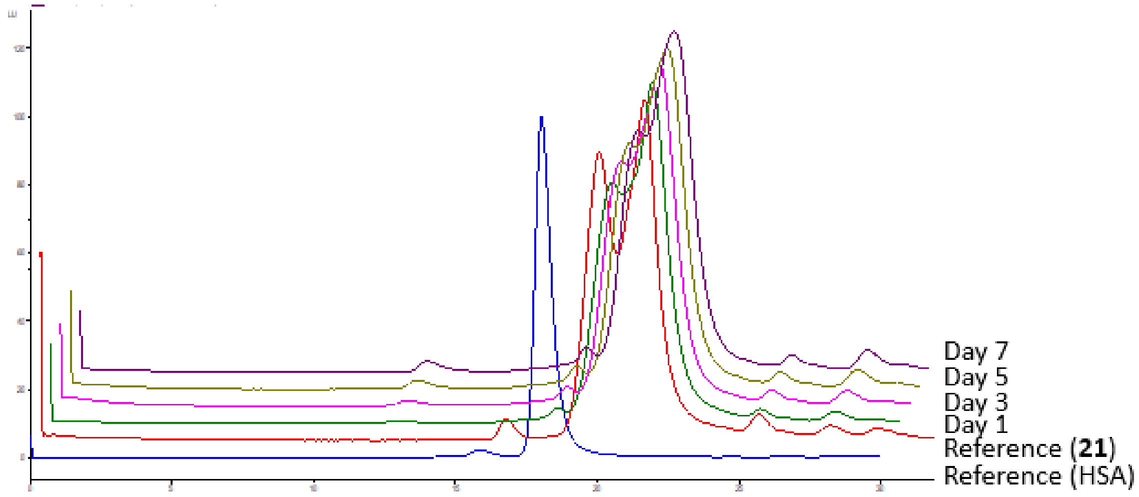

Overlay with HSA reference (uncorrected):

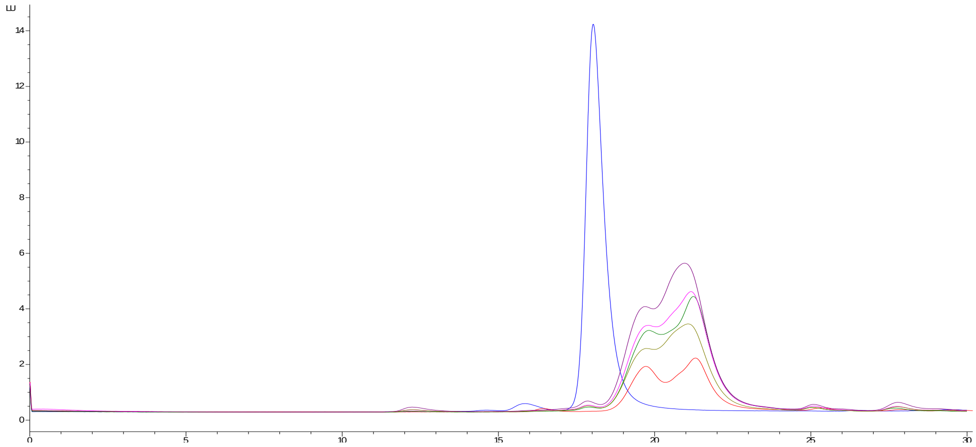
EU
14
12
10
8
6
4
2
0
0
5
10
15
20
25
30

References:

(1) Chudasama, V., Smith, M. E. B., Schumacher, F. F., Papaioannou, D., Waksman, G., Baker, J. R., Caddick, S. (2011) Bromopyridazinedione-Mediated Protein and Peptide Bioconjugation. *Chem. Commun. 47*, 8781.

(2) Bahou, C., Richards, D. A., Maruani, A., Love, E. A., Javaid, F., Caddick, S., Baker, J. R., Chudasama, V. (2018) Highly Homogeneous Antibody Modification through Optimisation of the Synthesis and Conjugation of Functionalised Dibromopyridazinediones. *Org. Biomol. Chem. 16*, 1359–1366.

(3) Lang, K., Davis, L., Wallace, S., Mahesh, M., Cox, D. J., Blackman, M. L., Fox, J. M., Chin, J. W. (2012) Genetic Encoding of Bicyclononynes and Trans -Cyclooctenes for Site-Specific Protein Labeling in Vitro and in Live Mammalian Cells via Rapid Fluorogenic Diels–Alder Reactions. *J. Am. Chem. Soc. 134*, 10317–10320.

(4) Hernández-Gil, J., Braga, M., Harriss, B. I., Carroll, L. S., Leow, C. H., Tang, M.-X., Aboagye, E. O., Long, N. J. (2019) Development of 68 Ga-Labelled Ultrasound Microbubbles for Whole-Body PET Imaging. *Chem. Sci. 10*, 5603–5615.

(5) Rasmussen, L. K. (2006) Facile Synthesis of Mono-, Di-, and Trisubstituted Alpha-Unbranched Hydrazines. *J. Org. Chem. 71*, 3627–3629.

(6) Zhang, K., Liu, J., Ohashi, S., Liu, X., Han, Z., Ishida, H. (2015) Synthesis of High Thermal Stability Polybenzoxazoles via Ortho -Imide-Functional Benzoxazine Monomers. *J. Polym. Sci. Part A Polym. Chem. 53*, 1330–1338.

(7) Bonger, K. M., van den Berg, R. J. B. H. N., Heitman, L. H., IJzerman, A. P., Oosterom, J., Timmers, C. M., Overkleeft, H. S., van der Marel, G. A. (2007) Synthesis and Evaluation of Homo-Bivalent GnRHR Ligands. *Bioorg. Med. Chem. 15*, 4841–4856.

(8) Yue, X., Feng, Y., Yu, Y. B. (2013) Synthesis and Characterization of Fluorinated Conjugates of Albumin. *J. Fluor. Chem. 152*, 173–181.

(9) Wall, A., Wills, A. G., Forte, N., Bahou, C., Bonin, L., Nicholls, K., Ma, M. T., Chudasama, V., Baker, J. R. (2020) One-Pot Thiol–Amine Bioconjugation to Maleimides: Simultaneous Stabilisation and Dual Functionalisation. *Chem. Sci.*

(10) Spears, R. J., Brabham, R. L., Budhadev, D., Keenan, T., McKenna, S., Walton, J., Brannigan, J. A., Brzozowski, A. M., Wilkinson, A. J., Plevin, M., Fascione, M. A. (2018) Site-Selective C-C Modification of Proteins at Neutral PH Using Organocatalyst-Mediated Cross Aldol Ligations. *Chem. Sci.*

(11) Moody, P., Smith, M. E. B., Ryan, C. P., Chudasama, V., Baker, J. R., Molloy, J., Caddick, S. (2012) Bromomaleimide-Linked Bioconjugates Are Cleavable in Mammalian Cells. *ChemBioChem 13*, 39–41.

(12) Robinson, E., Nunes, J. P. M., Vassileva, V., Maruani, A., Nogueira, J. C. F., Smith, M. E. B., Pedley, R. B., Caddick, S., Baker, J. R., Chudasama, V. (2017) Pyridazinediones Deliver Potent, Stable, Targeted and Efficacious Antibody–Drug Conjugates (ADCs) with a Controlled Loading of 4 Drugs per Antibody. *RSC Adv. 7*, 9073–9077.
